# Transition-metal-free electrochemical cross-electrophile coupling of activated benzyl alcohols and primary alkyl bromides

**DOI:** 10.1126/sciadv.aeb3720

**Published:** 2026-01-02

**Authors:** Guang Chen, Changhao Huang, Like Luo, Dayu Tian, Xiaocheng Wang, Hai-Jun Zhang

**Affiliations:** ^1^ State Key Laboratory of Precision and Intelligent Chemistry, Department of Chemistry, University of Science and Technology of China, Hefei, Anhui 230026, China.; ^2^ Department of Rheumatology and Immunology, The First Affiliated Hospital of Anhui Medical University, No. 218 Jixi Road, Hefei, Anhui 230022, China.

## Abstract

Cross-electrophile coupling (XEC) has emerged as a powerful strategy for constructing carbon─carbon bonds. Here, we report the first transition-metal-free electrochemical XEC between readily available benzyl alcohol derivatives and primary alkyl bromides, enabling the efficient construction of C(sp^3^)─C(sp^3^) bonds to access valuable benzylic quaternary and tertiary carbon centers. This method expands the reaction profiles of both benzyl alcohols and electrochemistry. A key to unlocking this transformation is the identification of 4-*tert*-butylbenzoyl (TBBz) as an effective activating group for benzyl alcohols. This modular protocol features broad substrate scope, compatibility with CO_2_ and chlorosilanes as coupling partners, and is amenable to gram-scale synthesis. The synthetic utility of this method is exemplified by the simplified synthesis of high-value commercial building blocks and advanced intermediates for bioactive compounds, as well as the facile access to bioisosteric analogs of drug molecules.

## Teaser

A transition-metal-free electrochemical method enables C(sp^3^)─C(sp^3^) bond construction from benzyl alcohols.

## INTRODUCTION

**Fig. 1.**
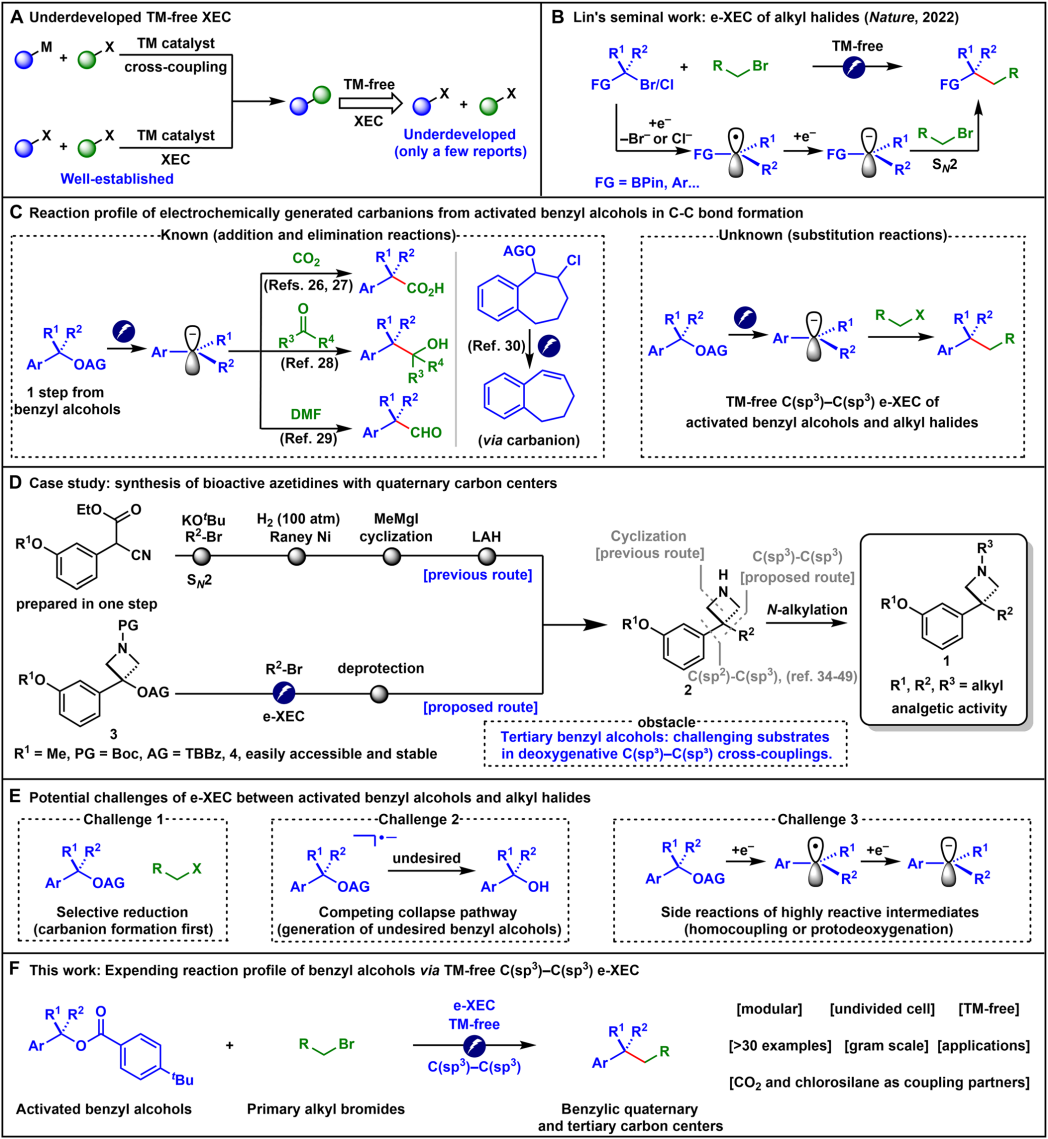
Background and this work. (**A**) Underdeveloped TM-free XEC strategy. (**B**) e-XEC of alkyl halides via the S*_N_*2 pathway to forge new C(sp^3^)─C(sp^3^) bonds. (**C**) Electrochemical deoxygenative generation of carbanions from activated benzyl alcohols for C─C bond formation via addition or elimination, as well as unexplored substitution pathways. (**D**) A case study comparing the simplified e-XEC strategy with traditional multistep routes for accessing bioactive azetidines bearing all-carbon quaternary centers. (**E**) Potential challenges of e-XEC between activated benzyl alcohols and alkyl halides. (**F**) This work: TM-free e-XEC of activated benzyl alcohols and primary alkyl bromides. FG, functional group; TBBz, 4-*tert*-butylbenzoyl; AG, activating group; PG, protecting group.

A fundamental objective in organic synthesis is the efficient construction of carbon─carbon bonds (*1*). Transition metal (TM)–catalyzed cross-coupling reactions have become one of the most reliable methods for achieving this goal (Fig. 1A) (*2*). However, conventional approaches often require the prior preparation of organometallic nucleophiles, adding synthetic complexity and requiring the handling of sensitive intermediates. In recent years, TM-catalyzed XEC reactions have emerged as a powerful alternative, enabling the direct coupling of two electrophiles and eliminating the need for organometallic reagents (*3*–*5*). Despite their advantages, the reliance on TM catalysts often introduces inherent economic and environmental challenges, as well as potential contamination risks in downstream applications. Therefore, the development of alternative, TM-free strategies is highly desirable. In this context, several groundbreaking studies have recently emerged, leveraging electrochemistry (*6*, *7*), enzyme catalysis (*8*), and frustrated ion pair chemistry (*9*) to achieve the highly challenging yet valuable TM-free C(sp^3^)─C(sp^3^) XECs. Among these, Lin and co-workers (*6*) reported an elegant electrochemical XEC (e-XEC) (*10*) of alkyl halides (*11*), offering a sustainable and tunable platform (Fig. 1B). In their proposed electrochemical-chemical-electrochemical-chemical (ECEC) mechanism (*12*), alkyl halides bearing anion-stabilizing substituents (e.g., α-halo pinacol boronate esters and benzyl chlorides) were selectively reduced to generate radical intermediates. These radicals subsequently underwent a rapid second reduction at the cathode to afford stable carbanions, which then reacted with primary alkyl halides via bimolecular nucleophilic substitution (S*_N_*2), forging new C(sp^3^)─C(sp^3^) bonds. Encouraged by this unique electrochemically enabled reactivity, we sought to explore the feasibility of using more stable and readily available benzyl alcohol derivatives as carbanion precursors in C(sp^3^)─C(sp^3^) e-XECs via electrochemical cleavage of the strong C─O bond (*3*–*5*, *13*–*19*).

Under electroreductive conditions, benzyl alcohols can generate carbanions via two modes of activation. Direct in situ reduction has been demonstrated but remains limited to the formation of C─H, C─B, C─Si, C─Ge, C─Sn, and C─CO_2_H bonds (*20*–*25*). In contrast, the preactivation strategy enables carbon─carbon bond formation with electrophiles such as CO_2_, carbonyl compounds, or *N*,*N*′-dimethylformamide (DMF) via addition reactions, or undergoes elimination to form styrenes (Fig. 1C) (*26*–*30*). However, the cross-coupling of these carbanions with alkyl halides via substitution reactions to forge C(sp^3^)─C(sp^3^) bonds remains an unmet challenge (*14*, *15*, *19*, *31*, *32*). If such a transformation could be realized, it would offer an alternative and powerful route to C(sp^3^)─C(sp^3^) bond construction.

The case study presented in Fig. 1D vividly illustrates both the potential advantages and key obstacles of pursuing this strategy. Azetidines **1**, featuring all-carbon quaternary centers, were previously prepared to investigate their analgetic activities (*33*). In the reported synthetic route, each analog was synthesized de novo to incorporate the desired alkyl groups via a lengthy linear sequence, including a high-pressure Raney Ni hydrogenation. Retrosynthetically, compound **2** could be constructed via C(sp^2^)─C(sp^3^) coupling. However, the corresponding tertiary alkyl coupling partner bearing an azetidine ring is generally challenging to access, and such transformations predominantly rely on TM catalysis (*34*–*49*). We propose that the synthesis of common intermediates **2** could be simplified via e-XECs between activated tertiary benzyl alcohols **3** and alkyl bromides, followed by removal of the protecting group. Notably, activated tertiary benzyl alcohols, such as compound **4**, are readily accessible in a single step through a consecutive one-pot sequence and exhibit excellent bench-top stability (see the Supplementary Materials for details). However, despite the broad availability of tertiary benzyl alcohols and their simple derivatives, the application of these substrates in deoxygenative C(sp^3^)─C(sp^3^) cross-coupling reactions to directly form all-carbon quaternary centers, one of the most synthetically challenging yet highly desirable motifs in medicinal chemistry (*50*), remains underdeveloped (*51*), with such substrates frequently reported to afford low yields or show poor compatibility (*52*, *53*). Therefore, establishing a viable e-XEC protocol for tertiary benzyl alcohols would substantially expand their synthetic utility. Moreover, this proposed TM-free approach offers a compelling advantage by completely eliminating the risk of metal contamination in the final targets.

Three potential challenges could hinder the successful realization of this transformation (Fig. 1E). First, selective reduction between activated benzyl alcohols and alkyl halides is essential: activated benzyl alcohols must be deeply reduced to carbanions before alkyl halides can be reduced (*6*, *10*). Second, previous studies suggest that a competing collapse pathway of in-situ generated radical anions could lead to the undesired formation of benzyl alcohols instead of benzylic carbanions (*34*, *54*–*56*). Third, the highly reactive intermediates, benzyl radicals and carbanions, are prone to side reactions, such as homocoupling or protodeoxygenation (*29*). It is worth noting that although the latter two challenges have been partially addressed in the context of addition and elimination reactions, they may still affect the selectivity or efficiency of substitution reactions.

Here, we report the successful development of a TM-free e-XEC between activated benzyl alcohols and primary alkyl bromides, enabling efficient construction of C(sp^3^)─C(sp^3^) bonds (Fig. 1F). This strategy expands the synthetic utility of these readily available alcohols and provides a valuable platform to access benzylic quaternary and tertiary carbon centers. Notably, this type of benzylic quaternary carbon center, featuring an aromatic ring attached to three alkyl groups, has attracted considerable research attention; however, its formation has predominantly relied on TM-catalyzed C(sp^2^)─C(sp^3^) coupling (*34*–*49*). Central to the success of our transformation is the identification of 4-*tert*-butylbenzoyl (TBBz) as an effective activating group, which substantially enhances the coupling efficiency. This modular protocol exhibits broad substrate scope and is readily amenable to gram-scale synthesis. Furthermore, CO_2_ and chlorosilanes have been demonstrated as viable coupling partners, further extending the utility of the method. Last, the power of this approach is showcased through the streamlined synthesis of advanced intermediates for bioactive compounds, high-value commercial building blocks, and bioisosteric analogs of drug molecules.

## RESULTS

### Reaction development

**Fig. 2.**
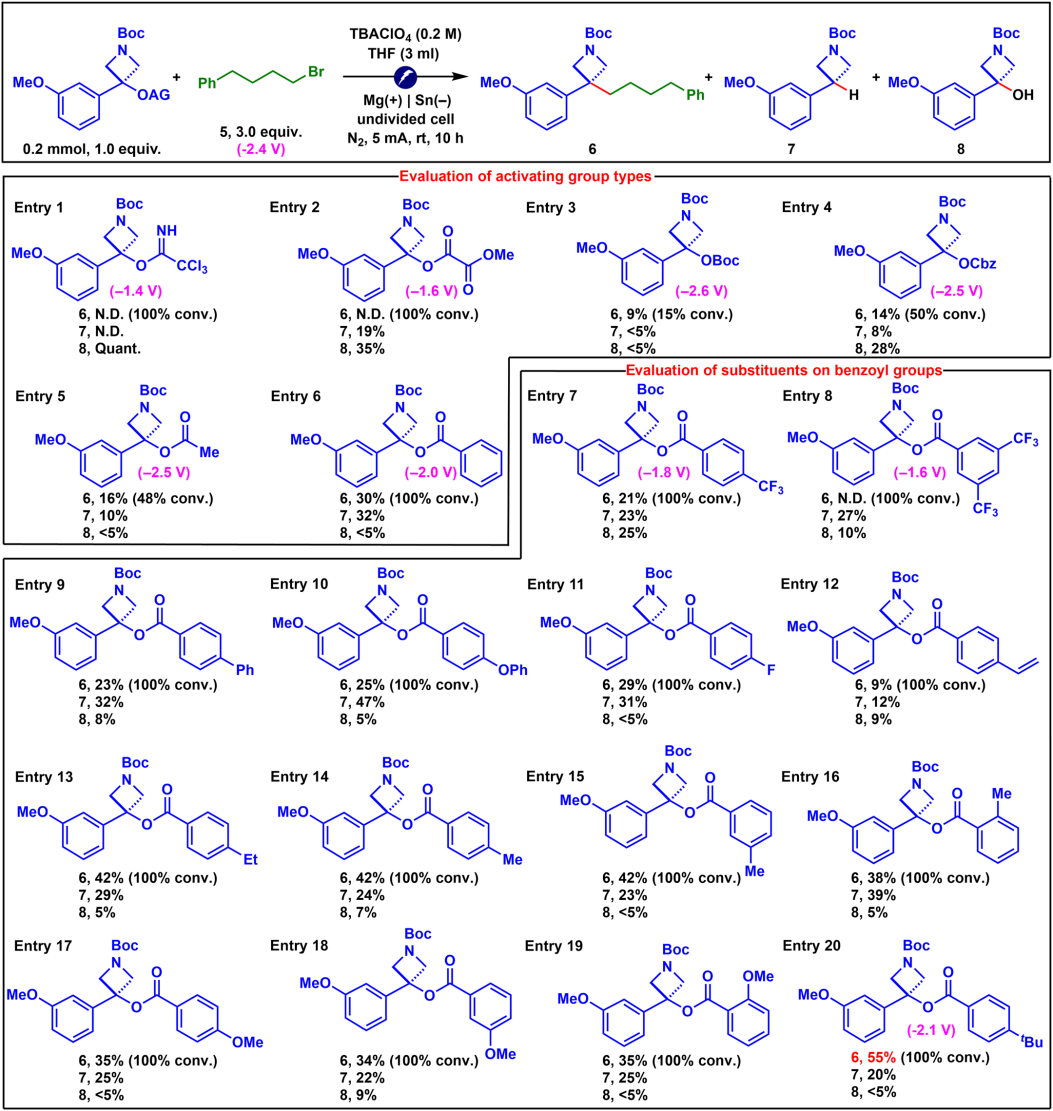
Evaluation of activating groups. Yields of **6** and **7** were determined by ^1^H-NMR, while the yields of **8** were determined by GC-MS, both using 1,3,5-trimethoxybenzene as an internal standard. All potentials were measured by cyclic voltammetry using a glassy carbon working electrode, a platinum wire counter electrode, and an Ag/AgCl reference electrode in THF containing 0.1 M TBAClO_4_ at room temperature. Values correspond to the onset potentials of the reduction waves (see the Supplementary Materials for details). AG, activating group; N.D., not detected; Conv., conversion; Quant., quantitative.

We initiated our study by evaluating a variety of activating groups that are readily installable on tertiary benzyl alcohols (Fig. 2, entries 1 to 6). Among them, the benzoyl group delivered the most promising result, affording the desired coupling product **6** in 30% yield (entry 6). As expected, the major detectable side products were the protodeoxygenation product **7** and the benzyl alcohol **8**. Product **7** likely arose from a Hofmann elimination process, as proposed by Lin and co-workers, wherein the in situ–formed carbanion intermediate acted as a strong base to deprotonate the tetrabutylammonium cation from the electrolyte (*6*, *57*). Product **8** was presumably formed via an undesired collapse of in situ–generated radical anions, as outlined in Fig. 1E. We next systematically evaluated a series of benzoyl groups bearing various substituents on the aromatic ring to assess the influence of electronic effects and substitution patterns (entries 6 to 20). Gratifyingly, TBBz emerged as the optimal activating group, delivering the desired coupling product in 55% yield (entry 20). Notably, when activated benzyl alcohols exhibited more negative reduction potentials than alkyl bromide **5**, the reaction proceeded with low conversion (entries 3 to 5). In contrast, those with less negative reduction potentials than **5** consistently led to full conversion, although the yields of the desired product varied (entries 1 to 2, 6 to 8, and 20). These observations suggest that reduction potential alone is not the sole determining factor in this transformation. Instead, as suggested by previous studies from Lam, Markó, and Gong (*34*, *54*–*56*), we propose that the collapse pathway and the collapse rate of the in situ–generated radical anions may also play a crucial role.

**Fig. 3.**
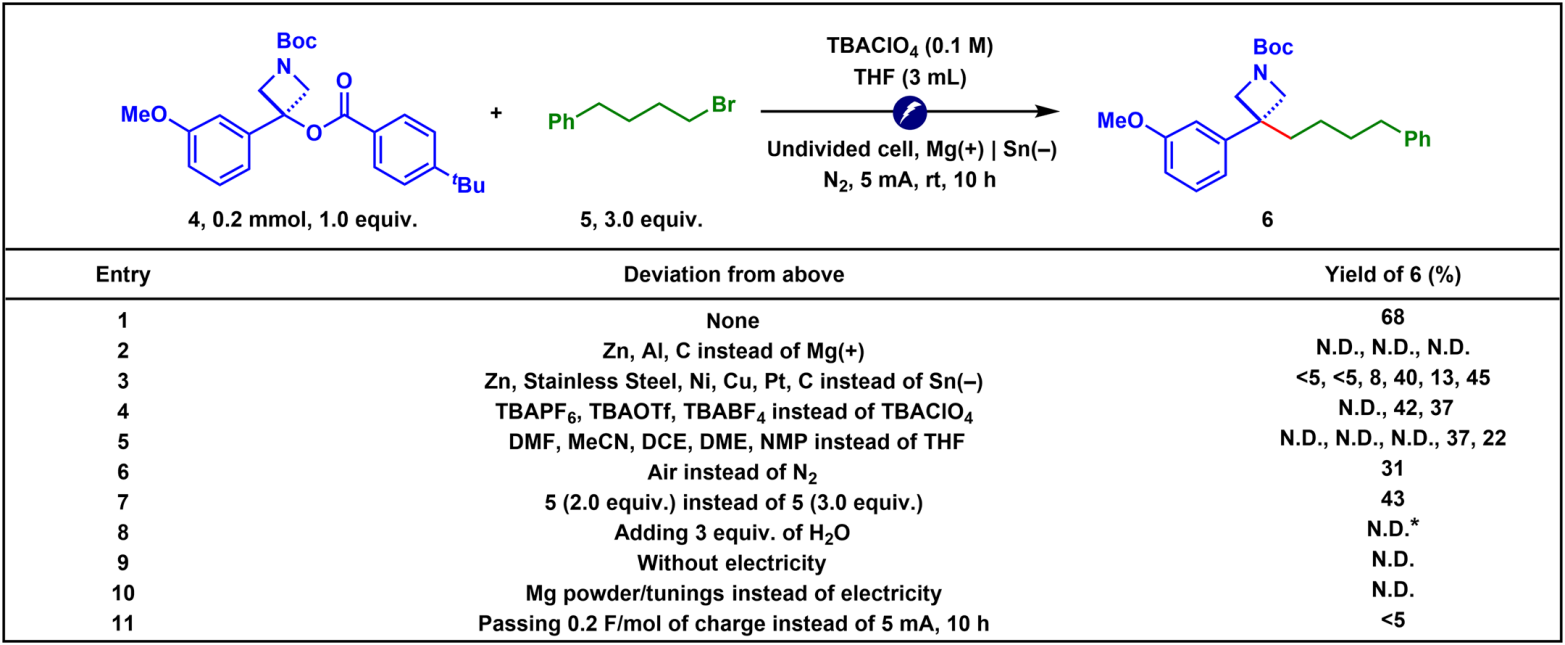
Optimization of reaction conditions. Yields were determined by ^1^H-NMR using 1,3,5-trimethoxybenzene as an internal standard. *The protodeoxygenation product **7** was isolated in 48% yield, corresponding to 62% conversion of **4**. N.D., not detected.

Having identified TBBz as the optimal activating group, we proceeded to optimize the reaction conditions, ultimately achieving a 68% yield under fully optimized parameters (Fig. 3, entry 1). Any variation in electrode materials (entries 2 to 3), electrolytes (entry 4), or solvents (entry 5) resulted in inferior outcomes. Replacing the nitrogen atmosphere with air also led to a substantial decrease in product formation (entry 6). Decreasing the equivalents of alkyl bromide under the standard conditions led to a lower yield, affording 43% of the desired product (entry 7). The addition of 3 equivalents of H_2_O suppressed the desired coupling reaction and instead led to the formation of the protodeoxygenation product **7** in 48% yield (62% conversion of **4**), likely due to interception of the carbanion intermediate by H_2_O (entry 8). Control experiments highlighted the indispensable role of electricity in this transformation: no product formation was observed either in the absence of electricity (entry 9) or when magnesium powder or turnings were used as chemical reductants (entry 10). Passing 0.2 F/mol of charge, followed by stirring for 10 hours without electricity, resulted in minimal conversion, indicating that continuous electrolysis is required for product formation (entry 11).

### Evaluation of the substrate scope

**Fig. 4.**
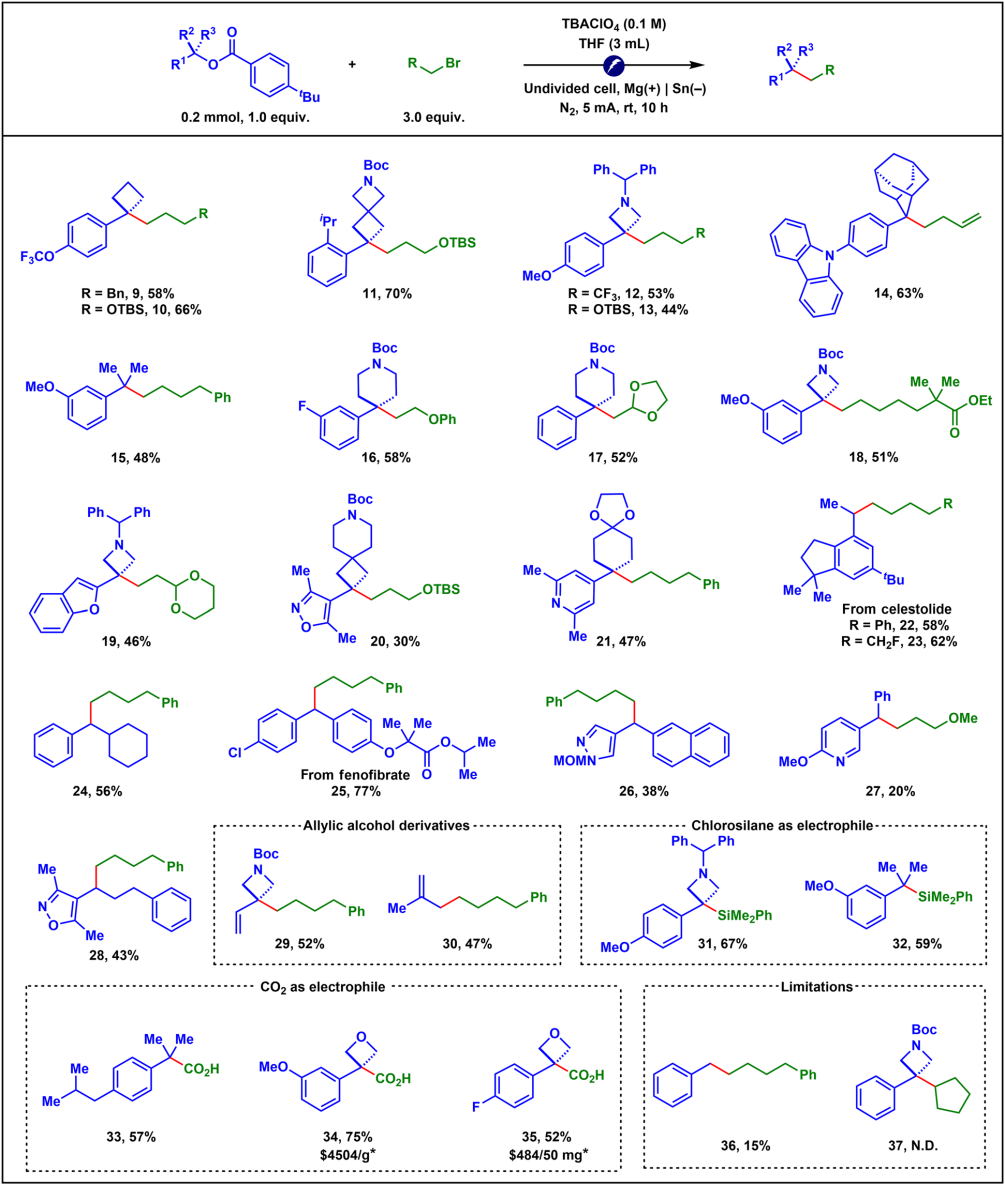
Substrate scope. Isolated yields were reported. *The price is from ChemScene LLC, search date April 2025.

With the optimized conditions in hand, we next turned our attention to exploring the substrate scope (Fig. 4). Given recent research in medicinal chemistry linking an increased fraction of sp^3^ carbons in drug candidates to improved clinical trial success (*58*–*60*), we incorporated a variety of C(sp^3^)-rich saturated cyclic structures into our scope, such as cyclobutane, azetidine, adamantane, piperidine, spirocyclic cyclobutane, cyclohexane, and oxetane. A variety of activated tertiary and secondary benzyl alcohols bearing diverse substituents on the aromatic ring, such as trifluoromethoxy (**9**, **10**), isopropyl (**11**), methoxy (**12**, **13**, **15**, **18**), *N*-carbazolyl (**14**), fluoro (**16**), and chloro (**25**) groups, proved to be effective substrates. Notably, the e-XEC of activated heterobenzyl alcohols, including those derived from benzofuran, isoxazole, pyridine, and pyrazole scaffolds, also delivered synthetically useful yields (**19** to **21** and **26** to **28**). In addition, activated allylic alcohols participated smoothly in the transformation (**29** to **30**). As for the scope of alkyl bromides, a broad range of primary alkyl bromides bearing various functional groups, including TBS ethers (**10**, **11**, **13**, and **20**), fluoroalkyl groups (**12** and **23**), ethers (**16** and **27**), olefins (**14**), esters (**18** and **25**), and acetals (**17** and **19**), were well tolerated. Furthermore, chlorosilanes (**31** and **32**) (*20*–*23*) and CO_2_ (**33** to **35**) (*26*, *27*, *61*) were successfully used as electrophilic coupling partners. It is noteworthy that carboxylic acids **34** and **35** are commercially expensive, and their synthetic procedures have not been reported according to a search of the Reaxys database, despite numerous reports on benzylic carboxylation methods. Nonetheless, activated primary benzyl alcohols delivered only low coupling yields, presumably due to their reduced nucleophilicity (**36**). Moreover, secondary alkyl bromides failed to afford any product, likely because of steric hindrance (**37**; see the Supplementary Materials for a complete list of ineffective substrates).

**Fig. 5.**
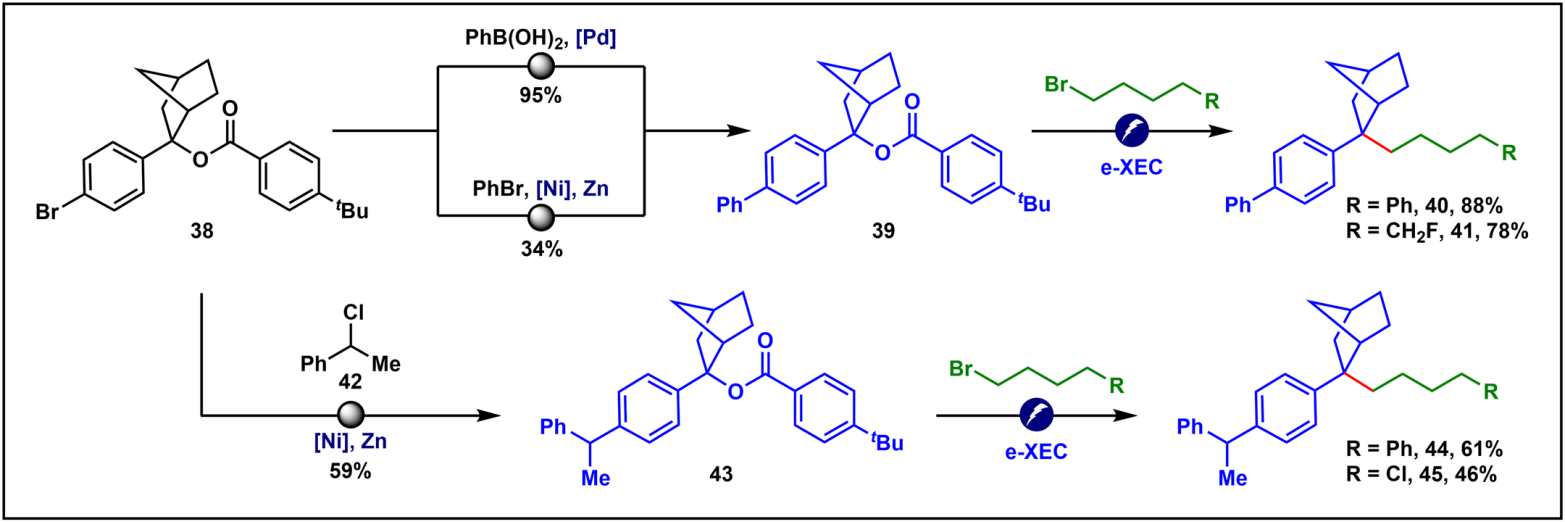
Orthogonal reactivity between TM-catalyzed cross-coupling and TM-free e-XEC. Isolated yields were reported. See details in the Supplementary Materials.

A notable advantage of using benzyl benzoate as a carbanion precursor in the e-XEC process lies in its considerable stability, which permits its survival through diverse synthetic transformations (Fig. 5). For instance, benzoate **38** underwent both a redox-neutral palladium-catalyzed Suzuki coupling (*62*) and a nickel-catalyzed reductive C(sp^2^)─C(sp^2^) coupling (*63*) to furnish product **39**, with the benzoate moiety remaining intact. The resultant benzyl benzoate **39** served as an effective precursor for our e-XEC methodology, facilitating the efficient construction of benzylic quaternary carbon centers in excellent yields (**40** to **41**). Likewise, benzyl benzoate **38** participated in a nickel-catalyzed reductive C(sp^2^)─C(sp^3^) coupling with benzyl chloride **42** (*64*), again leaving the benzoate group unaltered to afford compound **43**. Subsequent application of our e-XEC reaction to the untouched benzoate further underscored the orthogonal reactivity between the benzyl benzoate and benzyl chloride functionalities (**44** to **45**).

### Synthetic utility

**Fig. 6.**
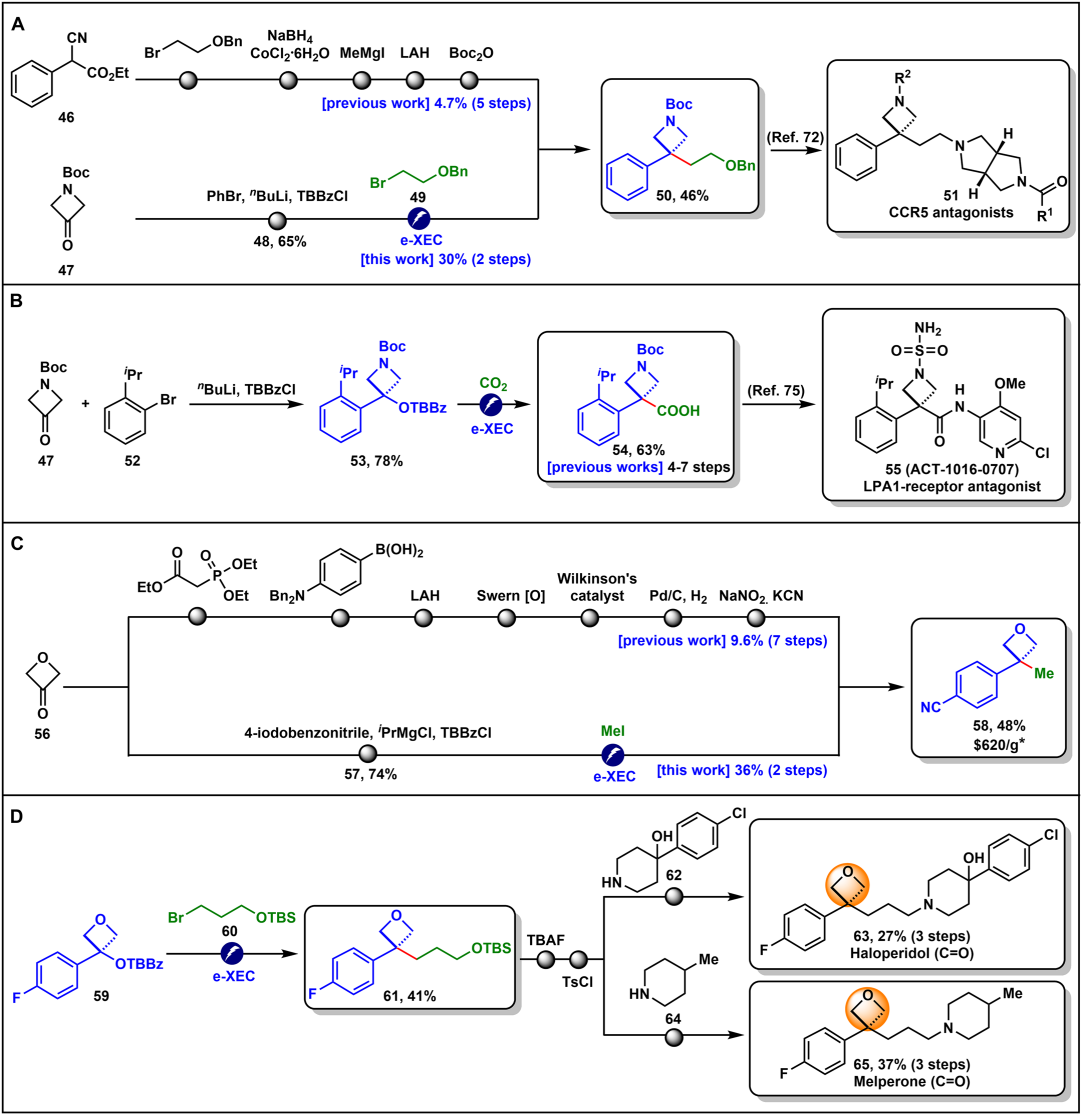
Synthetic utility. (**A**) Streamlined synthesis of the intermediate of CCR5 antagonists via the e-XEC strategy. (**B**) Synthesis of the intermediate of LPA1-receptor antagonists via the e-XEC strategy. (**C**) Simplified synthesis of compound **58** via the e-XEC strategy. (**D**) Synthesis of oxetane-containing analogs as bioisosteres of haloperidol and melperone. See details in the Supplementary Materials. *The price is from ChemScene LLC, search date April 2025.

Encouraged by the broad substrate scope, we further applied the e-XEC strategy to the synthesis of structurally important motifs. Azetidines and oxetanes are privileged scaffolds in drug discovery, offering advantages such as enhanced metabolic stability, improved pharmacokinetics, bioisosteric potential, and conformational rigidity (*65*, *66*). However, efficient synthetic methods for these strained small rings, especially those bearing quaternary carbon centers, remain scarce (*40*, *42*, *45*, *46*, *67*–*69*). In line with our group’s continued interest in developing concise approaches to access these structures (*70*, *71*), we demonstrate here how the e-XEC strategy can substantially simplify the synthesis of related compounds (Fig. 6, A to C). For example, azetidine **50**, an advanced intermediate toward CCR5 antagonists **51**, was previously synthesized in five steps with a 4.7% overall yield (*72*). Now, starting from 1-Boc-3-azetidinone **47** to form benzyl benzoate **48**, followed by e-XEC with alkyl bromide **49**, compound **50** can be prepared in just two steps (Fig. 6A). Similarly, azetidinyl carboxylic acid **54**, a key intermediate for the LPA1-receptor antagonist **55**, which was previously synthesized in 4 to 7 steps (*71*, *73*–*75*), can now be directly obtained by coupling benzyl benzoate **53** with CO_2_ (Fig. 6B). In addition, oxetane **58**, despite its simple structure, is commercially expensive and was previously synthesized in seven steps with a 9.6% overall yield from 3-oxetanone **56** (*76*). Now, starting from the same starting material, it can be synthesized in just two steps using our e-XEC method (Fig. 6C).

Bioisosteric replacement is a valuable strategy in drug discovery for fine-tuning the physicochemical properties of bioactive compounds (*77*). Here, we further demonstrate the power of our e-XEC method by efficiently synthesizing bioisosteric analogs of the drug molecules haloperidol and melperone through the replacement of the carbonyl group with an oxetane ring (Fig. 6D) (*66*, *78*–*80*). Specifically, oxetanyl benzoate **59**, prepared in a single step from **56**, underwent e-XEC with alkyl bromide **60** to afford compound **61** in 41% yield. Subsequent TBAF deprotection, *O*-tosylation, and an S*_N_*2 reaction with free piperidines furnished compounds **63** and **65** in reasonable overall yields. These oxetane-containing analogs serve as bioisosteres for haloperidol and melperone, respectively.

**Fig. 7.**
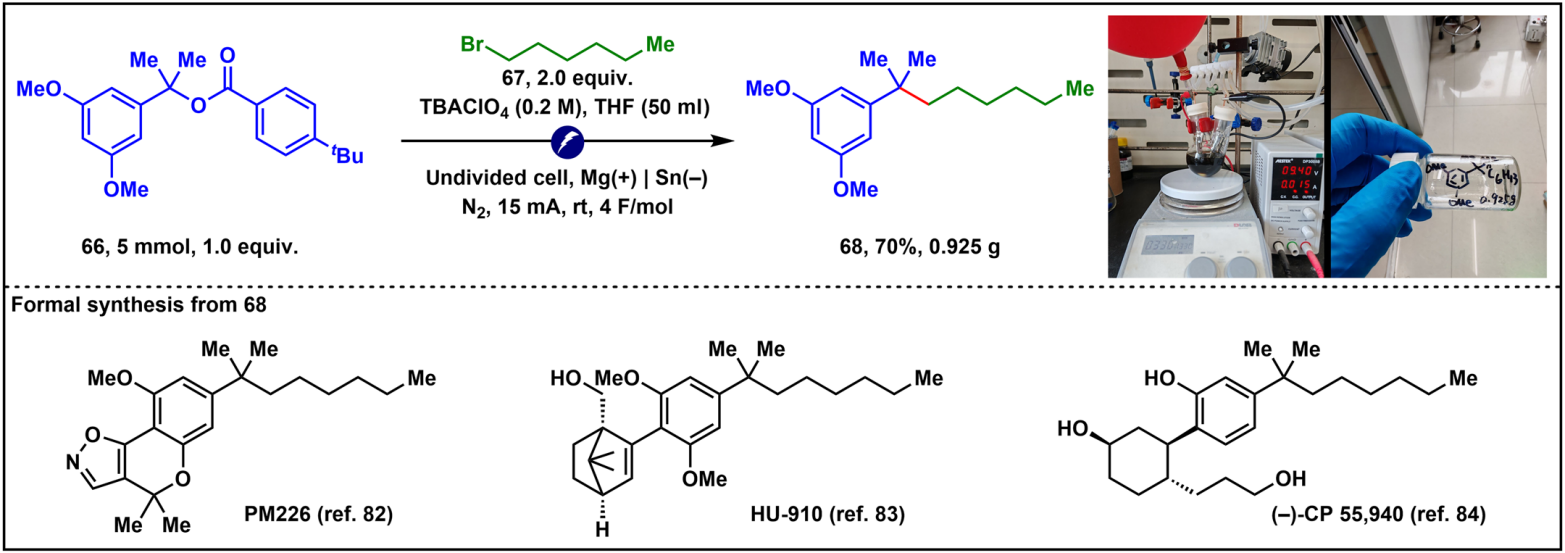
Scale-up synthesis.

Last, we demonstrated that this e-XEC reaction is amenable to scale-up with a reduced loading of the alkyl bromide (form 3 to 2 equiv.). As illustrated in Fig. 7, a 5-mmol-scale batch reaction delivered 0.925 g of compound **68**, a valuable intermediate for the formal synthesis of several cannabinoid receptor 2 modulators (*81*), such as PM226 (*82*), HU-910 (*83*), and (−)-CP 55,940 (*84*).

### Mechanistic considerations

**Fig. 8.**
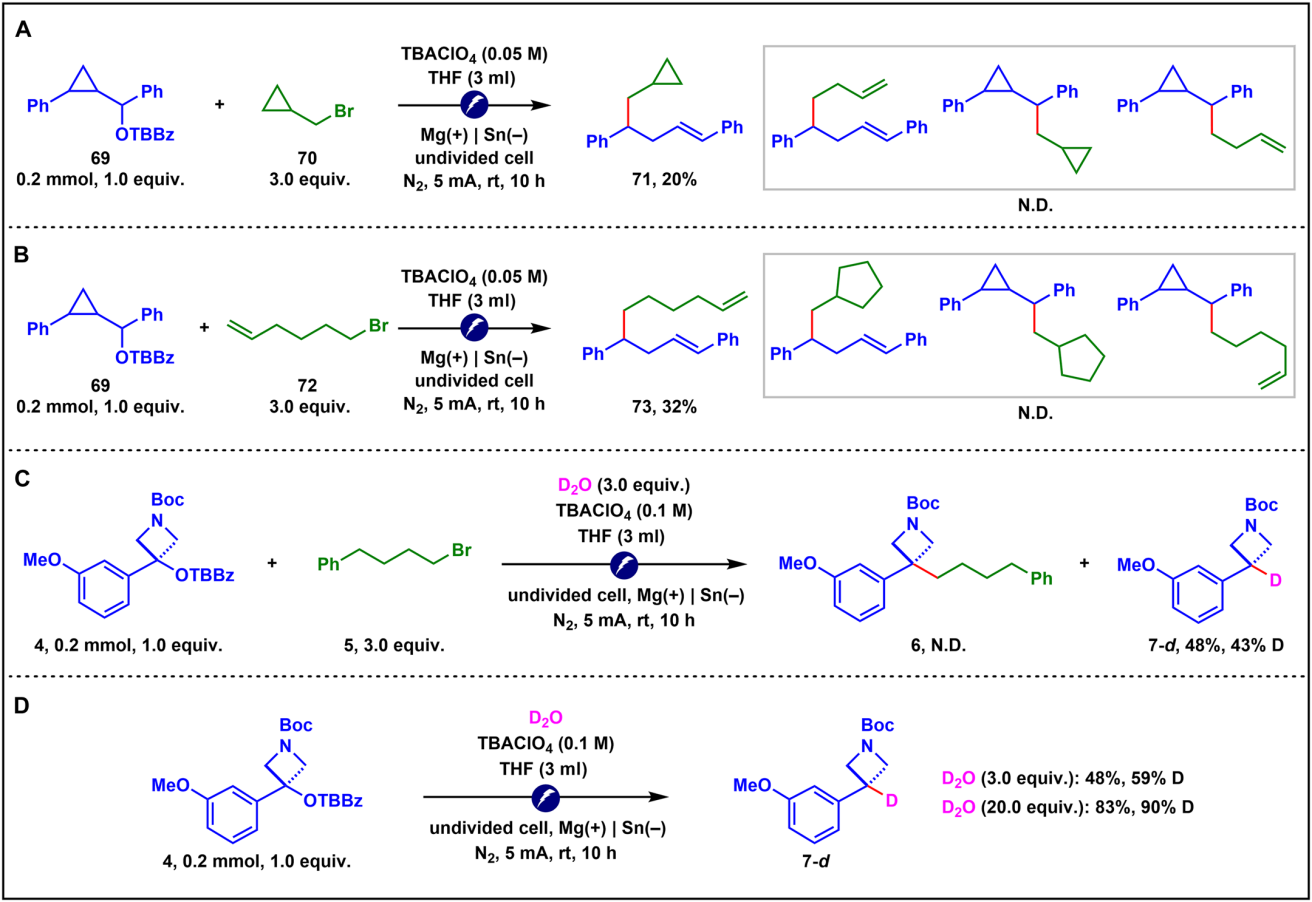
Mechanistic studies. (**A** and **B**) Radical clock experiments. (**C** and **D**) Deuterium-labeling experiments.

Several mechanistic studies were conducted to gain insight into the reaction mechanism (Fig. 8). First, radical clock experiments indicated that the activated benzyl alcohol derivatives likely underwent radical pathways, whereas the primary alkyl halides were not involved in radical processes (Fig. 8, A and B). Next, deuterium-labeling experiments were performed by introducing three equivalents of D_2_O into the model reaction (Fig. 8C). This led to two notable observations: (i) complete suppression of the desired coupling pathway, and (ii) isolation of the deoxygenative deuteration product **7-*d*** in 48% yield with 43% deuterium incorporation. Notably, in the absence of alkyl bromide **5**, the yield of the deuterated product **7-*d*** remained unchanged, while deuterium incorporation increased to 59% (Fig. 8D). Increasing the D_2_O loading to 20 equivalents further improved the product yield to 83%, with 90% deuterium incorporation (Fig. 8D). These results suggested the involvement of a carbanion intermediate (*28*, *85*, *86*). On the basis of the above observations and Lin’s study (*6*, *12*), we proposed that our reaction also proceeded via a similar ECEC mechanism. Activated benzyl alcohol derivatives were selectively reduced to generate radical intermediates, which then underwent rapid second reduction at the cathode to afford stable carbanions. These carbanions subsequently reacted with primary alkyl bromides via an S*_N_*2 pathway, forging new C(sp^3^)─C(sp^3^) bonds.

## DISCUSSION

In conclusion, we have developed the first TM-free e-XEC reaction of readily available activated benzyl alcohols and alkyl bromides, providing a modular and sustainable approach for constructing C(sp^3^)─C(sp^3^) bonds to access valuable benzylic quaternary and tertiary carbon centers. The key to unlocking this reactivity is the identification of TBBz as an effective activating group, coupled with the use of electroreductive conditions. This method offers broad substrate compatibility and can be readily scaled to gram quantities. The power of this method is showcased by synthesizing commercially expensive or bioactive azetidines and oxetanes bearing quaternary carbon centers. By expanding the synthetic potential of both benzyl alcohols and electrochemistry, this strategy holds considerable promise for future applications in synthetic and medicinal chemistry. Looking forward, addressing the current limitations of this TM-free approach, namely, its reliance on the substrate’s intrinsic reduction potential and the lack of stereocontrol, represents a promising avenue to bring its utility closer to that of TM-catalyzed reactions.

## MATERIALS AND METHODS

### General information

All reactions were conducted under a nitrogen atmosphere unless otherwise noted. Tetrahydrofuran (THF) was purchased from Energy Chemical and dried by refluxing with sodium metal and benzophenone. The dryness of the solvent was confirmed by the appearance of a deep purple color. The electrolyte tetrabutylammonium perchlorate (TBAClO_4_) was purchased from Energy Chemical and purified by recrystallization before use. All the other reagents were purchased at the highest commercial quality and used without further purification, unless otherwise specified. All electrolysis reactions were performed on oven-dried vial (5 ml) unless otherwise noted. The Mg electrode was cut into 5.3 × 0.8 × 0.2 cm^3^ plate. The Mg plate should be polished with spatula to remove the stains and insoluble salts in the surface before use. Sn plate was cut into 5.3 × 0.8 × 0.2 cm^3^ plate and could recyclable use. The typical interelectrode spacing was approximately 4.5 mm, and the electrodes were immersed in the reaction solution to a depth of about 18 mm. Cyclic voltammetry data were measured with a Signal 1000E instrument. Nuclear magnetic resonance (NMR) spectra were recorded on Bruker Avance-600, Bruker Avance-500, and Avance-400 instruments, calibrated with residual undeuterated solvent (CHCl_3_ at 7.26 ppm ^1^H NMR, 77.16 ppm ^13^C NMR). High-resolution mass spectra (HRMS) were recorded on Waters Xevo G2QTOF/UPLC (Waters Corporation). Gas chromatography–mass spectrometry (EI) was recorded on Agilent 7820A GC systems and 5975 Series MSD.

### General procedure for the e-XEC reaction of activated benzyl alcohol with primary alkyl bromides or chlorosilanes

An oven-dried 5-ml electrochemical cell equipped with a stir bar, a magnesium plate anode, and a tin plate cathode was charged with activated benzyl alcohol (1.0 equiv., 0.2 mmol) and TBAClO_4_ (0.1 M). The cell was evacuated and back-filled with N_2_ for three times. A solution of alkyl bromide or chlorosilane (3.0 equiv., 0.6 mmol) in dry THF (3.0 ml) was then added via syringe under nitrogen. After prestirring for 5 min, the mixture was electrolyzed at a constant current of 5 mA (current density = 1.18 mA/cm^2^) for 10 hours at room temperature. Upon completion, the reaction mixture was diluted with EtOAc and filtered through a short plug of silica gel. The filtrate was concentrated under reduced pressure, and the residue was purified by silica gel column chromatography or preparative thin layer chromatography (PTLC) to afford the desired product.

### General procedure for the e-XEC reaction of activated benzyl alcohol with CO_2_

An oven-dried 5-ml electrochemical cell equipped with a stir bar, a magnesium plate anode, and a tin plate cathode was charged with activated benzyl alcohol (1.0 equiv., 0.2 mmol) and TBAClO_4_ (0.1 M). The cell was evacuated and back-filled with CO_2_ for three times, and dry DMF (3.0 ml) was added by syringe. After prestirring for 5 min, the mixture was electrolyzed at a constant current of 10 mA (current density = 1.18 mA/cm^2^) for 10 hours at room temperature. Upon completion, the reaction mixture was acidified with 1 M HCl and extracted with EtOAc (3×). The combined organic layers were washed with saturated brine (3×), dried over Na_2_SO_4_, filtered, and concentrated under reduced pressure. The crude product was purified by reverse-phase column chromatography or PTLC to afford the desired product.

## Supplementary Materials

### This PDF file includes:

Supplementary Text Tables S1 to S6 Figs. S1 to S4 NMR Spectra References
