# Supplementary material for: Transition-metal-free electrochemical cross-electrophile coupling of activated benzyl alcohols and primary alkyl bromides
Source: Sci Adv. 2026 Jan 2;12(1):eaeb3720. doi: 10.1126/sciadv.aeb3720 (PMC13162218; doi:10.1126/sciadv.aeb3720)
Supplement: Supplementary file 1 — Supplementary Text Tables S1 to S6 Figs. S1 to S4 NMR Spectra References [file sciadv.aeb3720_sm.pdf]

Science Advances
AAAS

Supplementary Materials for

Transition-metal-free electrochemical cross-electrophile coupling of activated benzyl alcohols and primary alkyl bromides

Guang Chen *et al.*

Corresponding author: Xiaocheng Wang, wangxiaocheng@ucas.ac.cn; Hai-Jun Zhang, haijunzhang@ustc.edu.cn

Sci. Adv. **12**, eaeb3720 (2026)
DOI: 10.1126/sciadv.aeb3720

This PDF file includes:

Supplementary Text
Tables S1 to S6
Figs. S1 to S4
NMR Spectra
References

General Experimental

All reactions were conducted under a nitrogen atmosphere unless otherwise noted. Tetrahydrofuran (THF) was purchased from Energy Chemical and dried by refluxing with sodium metal and benzophenone. The dryness of the solvent was confirmed by the appearance of a deep purple color. The electrolyte tetrabutylammonium perchlorate ($TBAClO_4$) was purchased from Energy Chemical and purified by recrystallization before use. All the other reagents were purchased at the highest commercial quality and used without further purification, unless otherwise specified. Thin layer chromatography (TLC) was utilized for product detection, with visualization achieved using short-wave UV light, iodine (I_2), or potassium permanganate ($KMnO_4$). All electrolysis reactions were performed on oven-dried vial (5 mL) unless otherwise noted. The Mg electrode was cut into 5.3 x 0.8 x 0.2 cm^3 plate. The Mg plate should be polished with spatula to remove the stains and insoluble salts in the surface before use. Sn plate was cut into 5.3 x 0.8 x 0.2 cm^3 plate and could recyclable use. General procedure to recycle the Sn plate: after reaction, the Sn plate was sequentially washed with water and dichloromethane (DCM) (If necessary, dilute aqueous HCl was used to remove the precipitated Mg). The Sn plate was then immersed in DCM and subjected to ultrasonic cleaning for 5 minutes. Afterward, the plate was rinsed with DCM, polished with sandpaper, and finally dried in an oven before reuse. The typical interelectrode spacing was approximately 4.5 mm, and the electrodes were immersed in the reaction solution to a depth of about 18 mm. Cyclic voltammetry data were measured with a Signal 1000E instrument. NMR spectra were recorded on Bruker Avance-600, Bruker Avance-500, and Avance-400 instruments, calibrated with residual undeuterated solvent ($CHCl_3$ at 7.26 ppm ^1H NMR, 77.16 ppm ^{13}C NMR). The following abbreviations indicate multiplicities: s = singlet, d = doublet, t = triplet, q = quartet, p = pentet, m = multiplet, br = broad. High-resolution mass spectra (HRMS) were recorded on Waters Xevo G2QTOF/UPLC (Waters Corporation). GCMS (EI) was recorded on Agilent 7820A GC systems and 5975 Series MSD.

General Procedure for the Synthesis of Activated Benzyl Alcohols

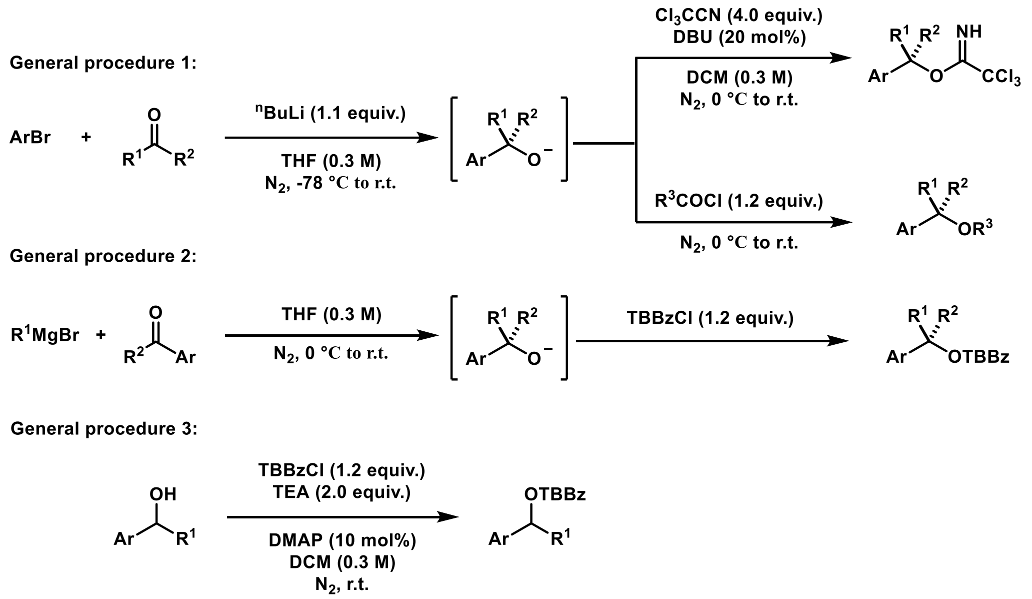

The trichloroacetimidate derivatives were prepared following the reported literature procedures (*71*).

General procedure 1: Under nitrogen atmosphere, to a solution of aryl halides (1.1 equiv.) in dry THF (0.3 M) was treated dropwise a solution of *n*-BuLi (2.5 M in hexanes, 1.1 equiv.) at –78 °C. After stirring for 1 h, ketones or aldehydes (1.0 equiv.) in dry THF was added dropwise to the reaction mixture. The reaction mixture was stirred at –78 °C for 10 minutes and allowed to warm to room temperature overnight. The reaction mixture was then returned to the ice bath, and R^3COCl (1.2 equiv.) was added dropwise. The mixture was allowed to warm to room temperature and stirred for 3 h. After concentration in vacuo, the crude product was directly purified by silica gel column chromatography to give the desired compounds.

General procedure 2: Under nitrogen atmosphere, to a solution of ketones or aldehydes (1.0 equiv.) in dry THF (0.3 M) was treated dropwise a solution of MeMgBr (3.0 M in Et_2O, 1.1 equiv.) at 0 °C. The reaction mixture was stirred at 0 °C for 5 minutes and allowed to warm to room temperature overnight. The reaction mixture was then returned to the ice bath, and 4-(tert-butyl)benzoyl chloride (TBBzCl, 1.2 equiv.) was added dropwise. The mixture was allowed to warm to room temperature and stirred for 3 h. After concentration in vacuo, the crude product was directly purified by silica gel column chromatography to afford the desired compounds.

General procedure 3: Under nitrogen atmosphere, to a solution of benzyl alcohol (1.0 equiv.), DMAP (10 mol%) and TEA (2.0 equiv.) in dry DCM (0.3 M) was treated dropwise with TBBzCl (1.2 equiv.) at 0 °C. The mixture was allowed to warm to room temperature and stirred for 3 h. After concentration in vacuo, the crude product was directly purified by silica gel column chromatography to afford the desired compounds.

Table S1 Activated Benzyl Alcohols Used in This Work

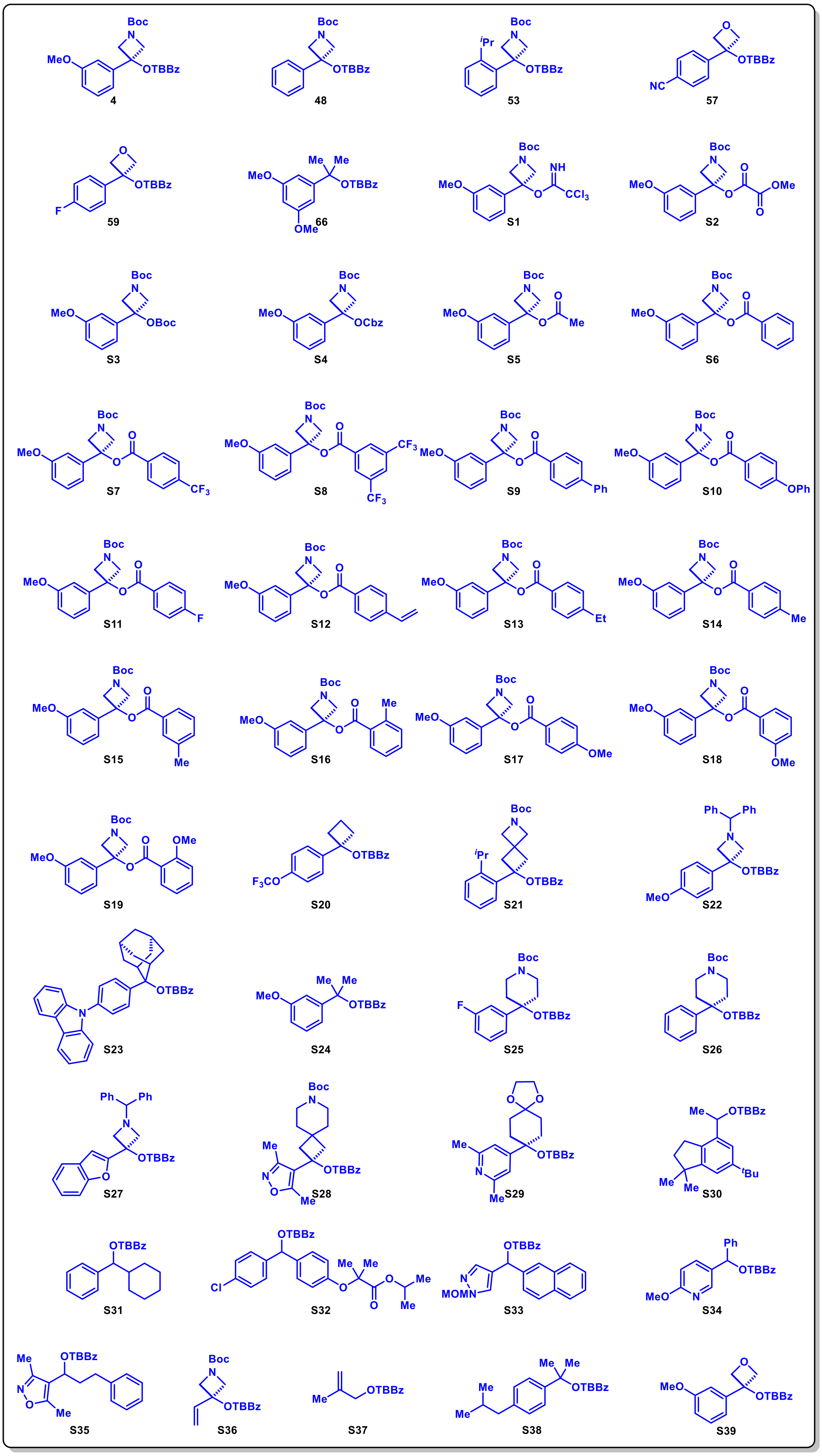

OTBBz: 4-tert-butylbenzoyl

Characterization Data of Activated Benzyl Alcohols

Compound 4

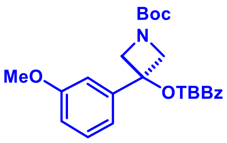

Prepared following the **General procedure 1** using 1-bromo-3-methoxybenzene (1.1 equiv., 5.5 mmol), *n*-BuLi (2.5 M in hexanes, 1.1 equiv., 5.5 mmol), tert-butyl 3-oxoazetidine-1-carboxylate (1.0 equiv., 5 mmol), TBBzCl (1.2 equiv., 6 mmol) and purified by silica gel column chromatography to afford the desired **Compound 4** (1.78 g, 77%) as a white solid.

^{1}H NMR (400 MHz, CDCl$_3$) δ 8.01 (d, *J* = 7.0 Hz, 2H), 7.48 (dd, *J* = 8.5, 1.5 Hz, 2H), 7.31 – 7.26 (m, 1H), 7.08 – 7.03 (m, 1H), 7.01 – 6.98 (m, 1H), 6.87 – 6.79 (m, 1H), 4.45 (q, *J* = 10.5, 10.1 Hz, 4H), 3.78 (s, 3H), 1.46 (s, 9H), 1.35 (s, 9H).

^{13}C NMR (101 MHz, CDCl$_3$) δ 165.12, 159.95, 157.46, 156.46, 141.98, 129.92, 129.83, 126.91, 125.67, 117.05, 112.99, 111.15, 80.25, 76.25, 61.74, 55.41, 35.31, 31.23, 28.48.

HRMS (ESI-TOF) m/z: calc'd for $C_{26}H_{33}NNaO_5$ [M+Na]$^+$: 462.2251; found 462.2254.

Compound S1

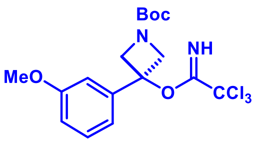

Prepared following the reported literature procedures (*71*) using 1-bromo-3-methoxybenzene (1.1 equiv., 5.5 mmol), *n*-BuLi (2.5 M in hexanes, 1.1 equiv., 5.5 mmol), tert-butyl 3-oxoazetidine-1-carboxylate (1.0 equiv., 5 mmol), Trichloroacetonitrile (4.0 equiv., 20 mmol) and purified by silica gel column chromatography to afford the desired **Compound S1** (1.7 g, 81%) as a white solid.

^{1}H NMR (400 MHz, CDCl$_3$) δ 8.40 (s, 1H), 7.31 (t, *J* = 8.0 Hz, 1H), 7.12 – 7.07 (m, 1H), 7.03 (t, *J* = 2.2 Hz, 1H), 6.86 (dd, *J* = 8.3, 2.5 Hz, 1H), 4.42 (d, *J* = 10.1 Hz, 2H), 4.34 (d, *J* = 10.2 Hz, 2H), 3.79 (s, 3H), 1.47 (s, 9H).

^{13}C NMR (101 MHz, CDCl$_3$) δ 159.87, 159.01, 156.33, 140.96, 129.84, 116.79, 113.46, 110.45, 91.05, 80.18, 78.95, 61.06, 55.28, 28.40.

HRMS (ESI-TOF) m/z: calc'd for $C_{17}H_{22}Cl_3N_2O_4$ [M+H]$^+$: 423.0640; found 423.0638.

Compound S2

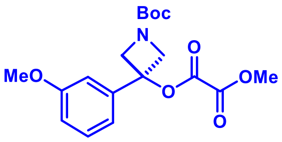

Prepared following the **General procedure 1** using 1-bromo-3-methoxybenzene (1.1 equiv., 5.5 mmol), *n*-BuLi (2.5 M in hexanes, 1.1 equiv., 5.5 mmol), tert-butyl 3-oxoazetidine-1-carboxylate (1.0 equiv., 5 mmol), methyl 2-chloro-2-oxoacetate (1.2 equiv., 6 mmol) and purified by silica gel column chromatography to afford the desired **Compound S2** (0.82 g, 45%) as a pale yellow oil.

^{1}H NMR (400 MHz, CDCl$_3$) δ 7.31 (t, *J* = 8.0 Hz, 1H), 7.05 – 7.00 (m, 1H), 6.96 (t, *J* = 2.2 Hz,

1H), 6.87 (ddd, J = 8.3, 2.6, 0.9 Hz, 1H), 4.43 (s, 4H), 3.90 (s, 3H), 3.81 (s, 3H), 1.45 (s, 9H).
^{13}C NMR (101 MHz, CDCl$_3$) δ 160.01, 157.72, 156.18, 155.98, 139.62, 130.06, 117.69, 114.01, 111.62, 80.50, 78.88, 60.74, 55.47, 28.44.
HRMS (ESI-TOF) m/z: calc'd for $C_{18}H_{23}NNaO_7$ [M+Na]$^+$: 388.1367; found 388.1360.

Compound S3

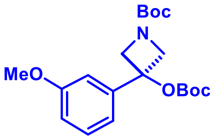

Prepared following the **General procedure 1** using 1-bromo-3-methoxybenzene (1.1 equiv., 5.5 mmol), *n*-BuLi (2.5 M in hexanes, 1.1 equiv., 5.5 mmol), tert-butyl 3-oxoazetidine-1-carboxylate (1.0 equiv., 5 mmol), Di-tert-butyl dicarbonate (1.2 equiv., 6 mmol) and purified by silica gel column chromatography to afford the desired **Compound S3** (1.21 g, 64%) as a colorless oil.
^{1}H NMR (400 MHz, CDCl$_3$) δ 7.31 (t, J = 8.0 Hz, 1H), 7.01 (ddd, J = 7.7, 1.8, 0.9 Hz, 1H), 6.96 – 6.93 (m, 1H), 6.85 (ddd, J = 8.3, 2.6, 0.9 Hz, 1H), 4.37 – 4.33 (m, 2H), 4.27 (d, J = 10.0 Hz, 2H), 3.81 (s, 3H), 1.46 (s, 9H), 1.43 (s, 9H).
^{13}C NMR (101 MHz, CDCl$_3$) δ 160.01, 157.72, 156.18, 155.98, 139.62, 130.06, 117.69, 114.01, 111.62, 80.50, 78.88, 61.26, 60.95, 55.47, 53.86, 28.44.
HRMS (ESI-TOF) m/z: calc'd for $C_{20}H_{29}NNaO_6$ [M+Na]$^+$: 402.1888; found 402.1892.

Compound S4

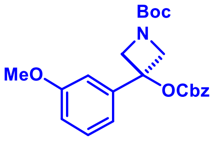

Prepared following the **General procedure 1** using 1-bromo-3-methoxybenzene (1.1 equiv., 5.5 mmol), *n*-BuLi (2.5 M in hexanes, 1.1 equiv., 5.5 mmol), tert-butyl 3-oxoazetidine-1-carboxylate (1.0 equiv., 5 mmol), benzyl carbonochloridate (1.2 equiv., 6 mmol) and purified by silica gel column chromatography to afford the desired **Compound S4** (1.24 g, 60%) as a colorless oil.
^{1}H NMR (400 MHz, CDCl$_3$) δ 7.38 – 7.32 (m, 6H), 7.04 – 7.00 (m, 1H), 6.94 (t, J = 2.2 Hz, 1H), 6.87 (dd, J = 8.2, 2.6 Hz, 1H), 5.11 (s, 2H), 4.37 (t, J = 9.1 Hz, 4H), 3.77 (s, 3H), 1.46 (s, 9H).
^{13}C NMR (101 MHz, CDCl$_3$) δ 159.94, 156.26, 153.07, 141.04, 134.80, 129.95, 128.79, 128.73, 128.47, 117.16, 113.73, 110.85, 80.25, 77.52, 69.99, 61.27, 55.32, 28.40.
HRMS (ESI-TOF) m/z: calc'd for $C_{23}H_{27}NNaO_6$ [M+Na]$^+$: 436.1731; found 436.1725.

Compound S5

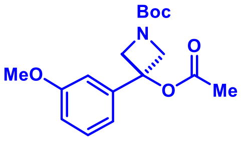

Prepared following the **General procedure 1** using 1-bromo-3-methoxybenzene (1.1 equiv., 5.5 mmol), *n*-BuLi (2.5 M in hexanes, 1.1 equiv., 5.5 mmol), tert-butyl 3-oxoazetidine-1-carboxylate (1.0 equiv., 5 mmol), Acetyl chloride (1.2 equiv., 6 mmol) and purified by silica gel column

chromatography to afford the desired **Compound S5** (1.15 g, 72%) as a colorless oil.

^{1}H NMR (400 MHz, CDCl$_3$) δ 7.29 (t, *J* = 8.0 Hz, 1H), 6.97 (ddd, *J* = 7.8, 1.8, 0.9 Hz, 1H), 6.91 (dd, *J* = 2.5, 1.7 Hz, 1H), 6.84 (ddd, *J* = 8.3, 2.6, 0.9 Hz, 1H), 4.32 (d, *J* = 1.6 Hz, 4H), 3.81 (s, 3H), 2.11 (s, 3H), 1.45 (s, 9H).

^{13}C NMR (101 MHz, CDCl$_3$) δ 169.50, 159.91, 156.37, 141.76, 129.87, 117.21, 113.07, 111.28, 80.20, 75.99, 61.85, 55.41, 28.46, 21.41.

HRMS (ESI-TOF) m/z: calc'd for $C_{17}H_{23}NNaO_5$ [M+Na]$^+$: 344.1469; found 344.1468.

Compound S6

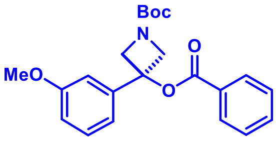

Prepared following the **General procedure 1** using 1-bromo-3-methoxybenzene (1.1 equiv., 5.5 mmol), *n*-BuLi (2.5 M in hexanes, 1.1 equiv., 5.5 mmol), tert-butyl 3-oxoazetidine-1-carboxylate (1.0 equiv., 5 mmol), Benzoyl chloride (1.2 equiv., 6 mmol) and purified by silica gel column chromatography to afford the desired **Compound S6** (1.27 g, 66%) as a white solid.

^{1}H NMR (400 MHz, CDCl$_3$) δ 8.10 – 8.03 (m, 2H), 7.62 – 7.55 (m, 1H), 7.46 (td, *J* = 7.8, 2.7 Hz, 2H), 7.28 (td, *J* = 8.0, 2.8 Hz, 1H), 7.04 (dd, *J* = 7.8, 2.2 Hz, 1H), 6.99 (d, *J* = 2.3 Hz, 1H), 6.82 (dd, *J* = 8.3, 2.7 Hz, 1H), 4.52 – 4.39 (m, 4H), 3.77 (s, 3H), 1.46 (s, 9H).

^{13}C NMR (101 MHz, CDCl$_3$) δ 165.05, 159.92, 156.38, 141.76, 133.63, 129.92, 129.87, 129.64, 128.64, 117.04, 113.02, 111.14, 80.23, 76.42, 61.55, 55.34, 28.43.

HRMS (ESI-TOF) m/z: calc'd for $C_{22}H_{25}NNaO_5$ [M+Na]$^+$: 406.1625; found 406.1619.

Compound S7

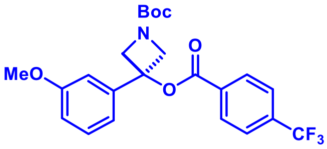

Prepared following the **General procedure 1** using 1-bromo-3-methoxybenzene (1.1 equiv., 5.5 mmol), *n*-BuLi (2.5 M in hexanes, 1.1 equiv., 5.5 mmol), tert-butyl 3-oxoazetidine-1-carboxylate (1.0 equiv., 5 mmol), 4-(trifluoromethyl)benzoyl chloride (1.2 equiv., 6 mmol) and purified by silica gel column chromatography to afford the desired **Compound S7** (1.63 g, 72%) as a white solid.

^{1}H NMR (400 MHz, CDCl$_3$) δ 8.19 (dt, *J* = 8.0, 0.8 Hz, 2H), 7.79 – 7.70 (m, 2H), 7.31 (t, *J* = 8.0 Hz, 1H), 7.04 (ddd, *J* = 7.8, 1.8, 0.9 Hz, 1H), 6.98 (dd, *J* = 2.5, 1.7 Hz, 1H), 6.85 (ddd, *J* = 8.3, 2.5, 0.9 Hz, 1H), 4.48 (d, *J* = 1.9 Hz, 4H), 3.79 (s, 3H), 1.47 (s, 9H).

^{13}C NMR (101 MHz, CDCl$_3$) δ 163.91, 160.03, 156.38, 141.27, 135.12 (q, *J* = 32.9 Hz), 132.94, 130.34, 130.06, 125.75 (q, *J* = 3.8 Hz), 123.66 (q, *J* = 272.8 Hz), 117.18, 113.24, 111.41, 80.42, 61.64, 55.44, 28.48.

^{19}F NMR (376 MHz, CDCl$_3$) δ -63.17.

HRMS (ESI-TOF) m/z: calc'd for $C_{23}H_{24}F_3NNaO_5$ [M+Na]$^+$: 474.1499; found 474.1499.

Compound S8

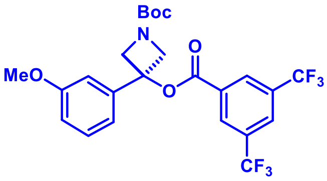

Prepared following the **General procedure 1** using 1-bromo-3-methoxybenzene (1.1 equiv., 5.5 mmol), *n*-BuLi (2.5 M in hexanes, 1.1 equiv., 5.5 mmol), tert-butyl 3-oxoazetidine-1-carboxylate (1.0 equiv., 5 mmol), 3,5-bis(trifluoromethyl)benzoyl chloride (1.2 equiv., 6 mmol) and purified by silica gel column chromatography to afford the desired **Compound S8** (1.38 g, 53%) as a white solid.

^{1}H NMR (400 MHz, $CDCl_3$) δ 8.49 (s, 2H), 8.10 (s, 1H), 7.32 (t, *J* = 8.0 Hz, 1H), 7.07 – 7.01 (m, 1H), 6.99 (t, *J* = 2.2 Hz, 1H), 6.87 (dd, *J* = 8.3, 2.5 Hz, 1H), 4.51 (s, 4H), 3.81 (s, 3H), 1.47 (s, 9H).

^{13}C NMR (101 MHz, $CDCl_3$) δ 163.03, 160.02, 156.32, 140.63, 132.54 (q, *J* = 34.1 Hz), 131.87, 130.11, 130.01 (q, *J* = 3.6 Hz), 126.98 (q, *J* = 3.5 Hz), 122.89 (q, *J* = 272.8 Hz), 117.44, 113.38, 111.75, 80.53, 77.96, 61.34, 55.47, 28.46.

^{19}F NMR (376 MHz, $CDCl_3$) δ -62.95.

HRMS (ESI-TOF) m/z: calc'd for $C_{24}H_{23}F_6NNaO_5$ $[M+Na]^+$: 542.1373; found 542.1377.

Compound S9

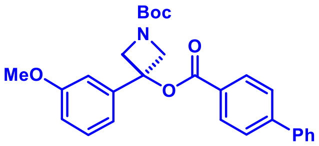

Prepared following the **General procedure 1** using 1-bromo-3-methoxybenzene (1.1 equiv., 5.5 mmol), *n*-BuLi (2.5 M in hexanes, 1.1 equiv., 5.5 mmol), tert-butyl 3-oxoazetidine-1-carboxylate (1.0 equiv., 5 mmol), [1,1'-biphenyl]-4-carbonyl chloride (1.2 equiv., 6 mmol) and purified by silica gel column chromatography to afford the desired **Compound S9** (1.33 g, 58%) as a white solid.

^{1}H NMR (500 MHz, $CDCl_3$) δ 8.15 (d, *J* = 8.4 Hz, 2H), 7.70 (d, *J* = 8.5 Hz, 2H), 7.66 – 7.60 (m, 2H), 7.49 (t, *J* = 7.5 Hz, 2H), 7.44 – 7.39 (m, 1H), 7.31 (t, *J* = 8.0 Hz, 1H), 7.08 (ddd, *J* = 7.8, 1.9, 0.9 Hz, 1H), 7.03 (t, *J* = 2.1 Hz, 1H), 6.85 (ddd, *J* = 8.3, 2.6, 0.9 Hz, 1H), 4.59 – 4.44 (m, 4H), 3.80 (s, 3H), 1.49 (s, 9H).

^{13}C NMR (126 MHz, $CDCl_3$) δ 164.97, 159.98, 156.42, 146.40, 141.85, 139.94, 130.44, 129.95, 129.10, 128.43, 128.40, 127.41, 127.32, 117.09, 113.08, 111.20, 80.25, 76.50, 61.60, 55.39, 28.47.

HRMS (ESI-TOF) m/z: calc'd for $C_{28}H_{29}NNaO_5$ $[M+Na]^+$: 482.1938; found 482.1933.

Compound S10

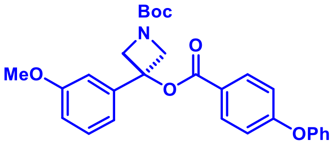

Prepared following the **General procedure 1** using 1-bromo-3-methoxybenzene (1.1 equiv., 5.5 mmol), *n*-BuLi (2.5 M in hexanes, 1.1 equiv., 5.5 mmol), tert-butyl 3-oxoazetidine-1-carboxylate (1.0 equiv., 5 mmol), 4-phenoxybenzoyl chloride (1.2 equiv., 6 mmol) and purified by silica gel

column chromatography to afford the desired **Compound S10** (1.31 g, 55%) as a colorless oil.
^{1}H NMR (400 MHz, CDCl$_3$) δ 8.06 – 8.00 (m, 2H), 7.43 – 7.38 (m, 2H), 7.30 (d, *J* = 8.0 Hz, 1H), 7.24 – 7.18 (m, 1H), 7.10 – 7.06 (m, 2H), 7.04 (ddd, *J* = 7.8, 1.8, 0.9 Hz, 1H), 7.02 – 6.97 (m, 3H), 6.84 (ddd, *J* = 8.3, 2.5, 0.9 Hz, 1H), 4.56 – 4.40 (m, 4H), 3.79 (s, 3H), 1.47 (s, 9H).
^{13}C NMR (101 MHz, CDCl$_3$) δ 164.60, 162.54, 159.98, 156.44, 155.56, 141.96, 132.10, 130.23, 129.96, 124.83, 123.89, 120.35, 117.48, 117.07, 113.02, 111.20, 80.26, 76.35, 61.79, 55.43, 28.49.
HRMS (ESI-TOF) m/z: calc'd for $C_{28}H_{29}NNaO_6$ $[M+Na]^+$: 498.1888; found 498.1891.

Compound S11

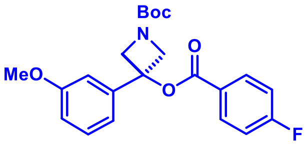

Prepared following the **General procedure 1** using 1-bromo-3-methoxybenzene (1.1 equiv., 5.5 mmol), *n*-BuLi (2.5 M in hexanes, 1.1 equiv., 5.5 mmol), tert-butyl 3-oxoazetidine-1-carboxylate (1.0 equiv., 5 mmol), 4-fluorobenzoyl chloride (1.2 equiv., 6 mmol) and purified by silica gel column chromatography to afford the desired **Compound S11** (1.20 g, 60%) as a white solid.
^{1}H NMR (400 MHz, CDCl$_3$) δ 8.12 – 8.04 (m, 2H), 7.30 (t, *J* = 8.0 Hz, 1H), 7.14 (t, *J* = 8.5 Hz, 2H), 7.06 – 7.01 (m, 1H), 6.97 (t, *J* = 2.2 Hz, 1H), 6.84 (dd, *J* = 8.3, 2.4 Hz, 1H), 4.46 (m, 4H), 3.79 (s, 3H), 1.46 (s, 9H).
^{13}C NMR (101 MHz, CDCl$_3$) δ 166.23 (d, *J* = 254.9 Hz), 164.13, 159.97, 156.40, 141.65, 132.54 (d, *J* = 9.5 Hz), 130.00, 125.95 (d, *J* = 2.9 Hz), 117.09, 115.90 (d, *J* = 22.0 Hz), 113.08, 111.26, 80.33, 76.64, 61.71, 55.42, 28.47.
^{19}F NMR (376 MHz, CDCl$_3$) δ -104.42.
HRMS (ESI-TOF) m/z: calc'd for $C_{22}H_{24}FNNaO_5$ $[M+Na]^+$: 424.1531; found 424.1534.

Compound S12

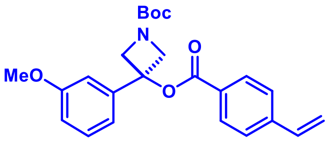

Prepared following the **General procedure 1** using 1-bromo-3-methoxybenzene (1.1 equiv., 5.5 mmol), *n*-BuLi (2.5 M in hexanes, 1.1 equiv., 5.5 mmol), tert-butyl 3-oxoazetidine-1-carboxylate (1.0 equiv., 5 mmol), 4-vinylbenzoyl chloride (1.2 equiv., 6 mmol) and purified by silica gel column chromatography to afford the desired **Compound S12** (1.42 g, 69%) as a colorless oil.
^{1}H NMR (400 MHz, CDCl$_3$) δ 8.03 (d, *J* = 8.4 Hz, 2H), 7.49 (d, *J* = 8.5 Hz, 2H), 7.29 (t, *J* = 8.0 Hz, 1H), 7.05 (ddd, *J* = 7.8, 1.8, 0.9 Hz, 1H), 6.99 (t, *J* = 2.1 Hz, 1H), 6.83 (ddd, *J* = 8.3, 2.6, 0.9 Hz, 1H), 6.76 (dd, *J* = 17.6, 10.9 Hz, 1H), 5.89 (dd, *J* = 17.6, 0.7 Hz, 1H), 5.44 – 5.38 (m, 1H), 4.46 (q, *J* = 10.0 Hz, 4H), 3.78 (s, 3H), 1.47 (s, 9H).
^{13}C NMR (101 MHz, CDCl$_3$) δ 164.86, 159.98, 156.43, 142.68, 141.87, 136.03, 130.26, 129.95, 128.79, 127.19, 126.39, 117.08, 113.10, 111.16, 80.25, 76.46, 61.54, 55.40, 28.48.
HRMS (ESI-TOF) m/z: calc'd for $C_{24}H_{27}NNaO_5$ $[M+Na]^+$: 432.1782; found 432.1786.

Compound S13

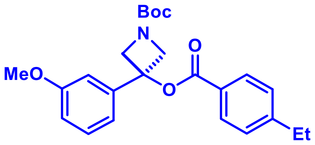

Prepared following the **General procedure 1** using 1-bromo-3-methoxybenzene (1.1 equiv., 5.5 mmol), *n*-BuLi (2.5 M in hexanes, 1.1 equiv., 5.5 mmol), tert-butyl 3-oxoazetidine-1-carboxylate (1.0 equiv., 5 mmol), 4-ethylbenzoyl chloride (1.2 equiv., 6 mmol) and purified by silica gel column chromatography to afford the desired **Compound S13** (1.39 g, 67%) as a white solid.

^{1}H NMR (400 MHz, CDCl$_3$) δ 8.02 – 7.94 (m, 2H), 7.28 (td, *J* = 7.7, 6.3, 3.8 Hz, 3H), 7.04 (dt, *J* = 7.4, 2.4 Hz, 1H), 6.99 (q, *J* = 2.4 Hz, 1H), 6.83 (dt, *J* = 8.0, 2.5 Hz, 1H), 4.55 – 4.33 (m, 4H), 3.78 (s, 3H), 2.71 (q, *J* = 7.6, 6.2 Hz, 2H), 1.46 (s, 9H), 1.32 – 1.22 (m, 3H).

^{13}C NMR (101 MHz, CDCl$_3$) δ 165.15, 159.93, 156.42, 150.66, 141.97, 130.06, 129.91, 128.20, 127.14, 117.01, 112.99, 111.09, 80.21, 76.23, 61.63, 55.37, 29.14, 28.46, 15.38.

HRMS (ESI-TOF) m/z: calc'd for $C_{24}H_{29}NNaO_5$ $[M+Na]^+$: 434.1938; found 434.1937.

Compound S14

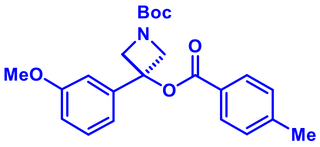

Prepared following the **General procedure 1** using 1-bromo-3-methoxybenzene (1.1 equiv., 5.5 mmol), *n*-BuLi (2.5 M in hexanes, 1.1 equiv., 5.5 mmol), tert-butyl 3-oxoazetidine-1-carboxylate (1.0 equiv., 5 mmol), 4-methylbenzoyl chloride (1.2 equiv., 6 mmol) and purified by silica gel column chromatography to afford the desired **Compound S14** (1.73 g, 86%) as a white solid.

^{1}H NMR (400 MHz, CDCl$_3$) δ 7.97 (d, *J* = 8.0 Hz, 2H), 7.32 – 7.27 (m, 3H), 7.09 – 7.04 (m, 1H), 7.00 (d, *J* = 2.3 Hz, 1H), 6.84 (dd, *J* = 8.3, 2.5 Hz, 1H), 4.47 (q, *J* = 10.0 Hz, 4H), 3.78 (s, 3H), 2.43 (s, 3H), 1.47 (s, 9H).

^{13}C NMR (101 MHz, CDCl$_3$) δ 165.13, 159.91, 156.40, 144.44, 141.95, 129.92, 129.89, 129.35, 126.93, 116.99, 113.00, 111.06, 80.19, 76.22, 61.82, 55.34, 28.44, 21.82.

HRMS (ESI-TOF) m/z: calc'd for $C_{23}H_{27}NNaO_5$ $[M+Na]^+$: 432.1782; found 432.1780.

Compound S15

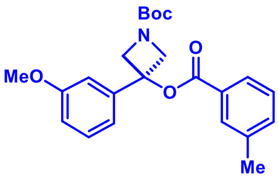

Prepared following the **General procedure 1** using 1-bromo-3-methoxybenzene (1.1 equiv., 5.5 mmol), *n*-BuLi (2.5 M in hexanes, 1.1 equiv., 5.5 mmol), tert-butyl 3-oxoazetidine-1-carboxylate (1.0 equiv., 5 mmol), 3-methylbenzoyl chloride (1.2 equiv., 6 mmol) and purified by silica gel column chromatography to afford the desired **Compound S15** (1.55 g, 78%) as a white solid.

^{1}H NMR (500 MHz, CDCl$_3$) δ 7.88 (d, *J* = 7.5 Hz, 2H), 7.41 (d, *J* = 7.6 Hz, 1H), 7.35 (t, *J* = 7.6 Hz, 1H), 7.29 (t, *J* = 8.0 Hz, 1H), 7.05 (dd, *J* = 7.8, 1.7 Hz, 1H), 7.00 (d, *J* = 2.3 Hz, 1H), 6.83 (dd,

J = 8.3, 2.6 Hz, 1H), 4.47 (q, J = 10.0 Hz, 4H), 3.78 (s, 3H), 2.41 (s, 3H), 1.47 (s, 9H).
^{13}C NMR (101 MHz, CDCl$_3$) δ 165.23, 159.92, 156.42, 141.85, 138.48, 134.40, 130.40, 129.91, 129.59, 128.55, 127.05, 117.05, 113.01, 111.15, 80.23, 76.36, 61.84, 55.36, 28.45, 21.38.
HRMS (ESI-TOF) m/z: calc'd for $C_{23}H_{27}NNaO_5$ [M+Na]$^+$: 432.1782; found 432.1779.

Compound S16

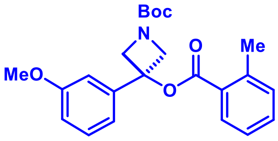

Prepared following the **General procedure 1** using 1-bromo-3-methoxybenzene (1.1 equiv., 5.5 mmol), *n*-BuLi (2.5 M in hexanes, 1.1 equiv., 5.5 mmol), tert-butyl 3-oxoazetidine-1-carboxylate (1.0 equiv., 5 mmol), 2-methylbenzoyl chloride (1.2 equiv., 6 mmol) and purified by silica gel column chromatography to afford the desired **Compound S16** (1.15 g, 58%) as a white solid.
^{1}H NMR (400 MHz, CDCl$_3$) δ 8.06 (dd, J = 7.8, 1.6 Hz, 1H), 7.45 (tt, J = 7.5, 1.5 Hz, 1H), 7.36 – 7.25 (m, 3H), 7.10 – 7.06 (m, 1H), 7.02 – 6.99 (m, 1H), 6.86 (ddd, J = 8.2, 2.6, 1.3 Hz, 1H), 4.54 – 4.40 (m, 4H), 3.80 (s, 3H), 2.58 (s, 3H), 1.48 (s, 9H).
^{13}C NMR (101 MHz, CDCl$_3$) δ 165.79, 159.97, 156.42, 141.97, 141.08, 132.75, 132.08, 130.94, 129.95, 128.73, 126.02, 117.04, 113.06, 111.08, 80.24, 76.27, 61.69, 55.39, 28.47, 22.02.
HRMS (ESI-TOF) m/z: calc'd for $C_{23}H_{27}NNaO_5$ [M+Na]$^+$: 432.1782; found 432.1781.

Compound S17

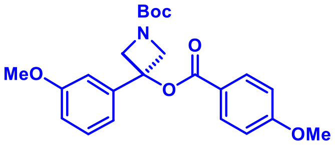

Prepared following the **General procedure 1** using 1-bromo-3-methoxybenzene (1.1 equiv., 5.5 mmol), *n*-BuLi (2.5 M in hexanes, 1.1 equiv., 5.5 mmol), tert-butyl 3-oxoazetidine-1-carboxylate (1.0 equiv., 5 mmol), 4-methoxybenzoyl chloride (1.2 equiv., 6 mmol) and purified by silica gel column chromatography to afford the desired **Compound S17** (1.59 g, 77%) as a colorless oil.
^{1}H NMR (400 MHz, CDCl$_3$) δ 8.03 (d, J = 8.8 Hz, 2H), 7.32 – 7.25 (m, 1H), 7.08 – 7.01 (m, 1H), 6.99 (d, J = 2.0 Hz, 1H), 6.96 – 6.92 (m, 2H), 6.83 (dd, J = 8.2, 2.6 Hz, 1H), 4.45 (q, J = 10.0 Hz, 4H), 3.86 (s, 3H), 3.77 (s, 3H), 1.47 (s, 9H).
^{13}C NMR (101 MHz, CDCl$_3$) δ 164.85, 163.98, 159.99, 156.46, 142.16, 132.05, 129.93, 122.13, 117.06, 113.95, 113.04, 111.13, 80.22, 76.18, 55.65, 55.41, 28.50.
HRMS (ESI-TOF) m/z: calc'd for $C_{23}H_{27}NNaO_6$ [M+Na]$^+$: 436.1731; found 436.1736.

Compound S18

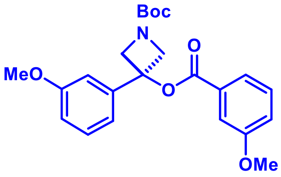

Prepared following the **General procedure 1** using 1-bromo-3-methoxybenzene (1.1 equiv., 5.5

mmol), *n*-BuLi (2.5 M in hexanes, 1.1 equiv., 5.5 mmol), tert-butyl 3-oxoazetidine-1-carboxylate (1.0 equiv., 5 mmol), 3-methoxybenzoyl chloride (1.2 equiv., 6 mmol) and purified by silica gel column chromatography to afford the desired **Compound S18** (1.50 g, 73%) as a colorless oil.

^{1}H NMR (400 MHz, CDCl$_3$) δ 7.69 (d, *J* = 7.6 Hz, 1H), 7.57 (t, *J* = 2.2 Hz, 1H), 7.38 (t, *J* = 8.0 Hz, 1H), 7.29 (t, *J* = 8.0 Hz, 1H), 7.14 (dd, *J* = 8.3, 2.7 Hz, 1H), 7.07 – 7.02 (m, 1H), 6.99 (t, *J* = 2.2 Hz, 1H), 6.84 (dd, *J* = 8.2, 2.5 Hz, 1H), 4.52 – 4.39 (m, 4H), 3.85 (s, 3H), 3.78 (s, 3H), 1.47 (s, 9H).

^{13}C NMR (101 MHz, CDCl$_3$) δ 165.00, 159.97, 159.78, 156.42, 141.77, 130.97, 129.95, 129.70, 122.28, 120.16, 117.09, 114.33, 113.09, 111.19, 80.27, 76.55, 61.98, 55.59, 55.40, 28.47.

HRMS (ESI-TOF) m/z: calc'd for $C_{23}H_{27}NNaO_6$ [M+Na]$^+$: 436.1731; found 436.1728.

Compound S19

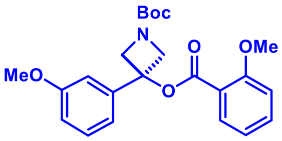

Prepared following the **General procedure 1** using 1-bromo-3-methoxybenzene (1.1 equiv., 5.5 mmol), *n*-BuLi (2.5 M in hexanes, 1.1 equiv., 5.5 mmol), tert-butyl 3-oxoazetidine-1-carboxylate (1.0 equiv., 5 mmol), 2-methoxybenzoyl chloride (1.2 equiv., 6 mmol) and purified by silica gel column chromatography to afford the desired **Compound S19** (1.44 g, 70%) as a colorless oil.

^{1}H NMR (400 MHz, CDCl$_3$) δ 7.96 – 7.84 (m, 1H), 7.56 – 7.47 (m, 1H), 7.29 (td, *J* = 8.0, 1.8 Hz, 1H), 7.09 (d, *J* = 7.9 Hz, 1H), 7.02 (ddt, *J* = 17.9, 8.5, 2.1 Hz, 3H), 6.83 (dd, *J* = 8.3, 2.4 Hz, 1H), 4.53 – 4.37 (m, 4H), 3.90 (s, 3H), 3.79 (s, 3H), 1.46 (s, 9H).

^{13}C NMR (101 MHz, CDCl$_3$) δ 164.40, 159.95, 159.90, 156.45, 142.09, 134.41, 132.14, 129.86, 120.28, 119.08, 117.17, 113.06, 112.24, 111.11, 80.13, 76.25, 61.70, 56.02, 55.36, 28.46.

HRMS (ESI-TOF) m/z: calc'd for $C_{23}H_{27}NNaO_6$ [M+Na]$^+$: 436.1731; found 436.1732.

Compound S20

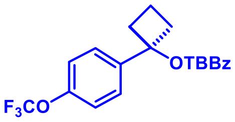

Prepared following the **General procedure 1** using 1-bromo-4-(trifluoromethoxy)benzene (1.1 equiv., 5.5 mmol), *n*-BuLi (2.5 M in hexanes, 1.1 equiv., 5.5 mmol), cyclobutanone (1.0 equiv., 5 mmol), TBBzCl (1.2 equiv., 6 mmol) and purified by silica gel column chromatography to afford the desired **Compound S20** (1.31 g, 67%) as a white solid.

^{1}H NMR (400 MHz, CDCl$_3$) δ 7.98 (d, *J* = 8.3 Hz, 2H), 7.59 (d, *J* = 8.7 Hz, 2H), 7.46 (d, *J* = 8.4 Hz, 2H), 7.23 – 7.16 (m, 2H), 2.78 (dd, *J* = 8.8, 6.8 Hz, 4H), 2.14 – 2.00 (m, 1H), 1.83 (dp, *J* = 11.3, 8.8 Hz, 1H), 1.34 (s, 9H).

^{13}C NMR (101 MHz, CDCl$_3$) δ 165.17, 156.82, 148.42 (q, *J* = 1.7 Hz), 141.64, 130.65, 129.57, 127.29, 125.48, 120.83, 120.59 (q, *J* = 257.1 Hz), 82.00, 35.20, 35.14, 31.22, 14.33.

^{19}F NMR (376 MHz, CDCl$_3$) δ -57.73.

HRMS (ESI-TOF) m/z: calc'd for $C_{22}H_{23}F_3NaO_3$ [M+Na]$^+$: 415.1492; found 415.1490.

Compound S21

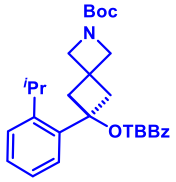

Prepared following the **General procedure 1** using 1-bromo-2-isopropylbenzene (1.1 equiv., 5.5 mmol), *n*-BuLi (2.5 M in hexanes, 1.1 equiv., 5.5 mmol), tert-butyl 6-oxo-2-azaspiro[3.3]heptane-2-carboxylate (1.0 equiv., 5 mmol), TBBzCl (1.2 equiv., 6 mmol) and purified by silica gel column chromatography to afford the desired **Compound S21** (0.98 g, 40%) as a white solid.

^{1}H NMR (400 MHz, CDCl$_3$) δ 7.88 (d, *J* = 8.2 Hz, 2H), 7.60 (d, *J* = 7.8 Hz, 1H), 7.40 (d, *J* = 8.3 Hz, 2H), 7.33 – 7.23 (m, 2H), 7.18 (td, *J* = 7.2, 6.5, 2.2 Hz, 1H), 4.03 (s, 2H), 3.80 (s, 2H), 3.35 (h, *J* = 6.8 Hz, 1H), 3.05 (t, *J* = 22.3 Hz, 4H), 1.41 (s, 9H), 1.31 (s, 9H), 1.14 (d, *J* = 6.8 Hz, 6H).

^{13}C NMR (101 MHz, CDCl$_3$) δ 165.08, 156.71, 156.22, 148.07, 137.10, 129.46, 128.70, 127.77, 127.74, 127.22, 125.41, 125.01, 80.42, 79.57, 60.66, 45.41, 35.19, 32.24, 31.22, 29.50, 28.50, 24.53.

HRMS (ESI-TOF) m/z: calc'd for $C_{31}H_{41}NNaO_4$ $[M+Na]^+$: 514.2928; found 514.2932.

Compound S22

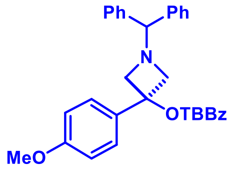

Prepared following the **General procedure 1** using 1-bromo-4-methoxybenzene (1.1 equiv., 5.5 mmol), *n*-BuLi (2.5 M in hexanes, 1.1 equiv., 5.5 mmol), 1-benzhydrylazetidin-3-one (1.0 equiv., 5 mmol), TBBzCl (1.2 equiv., 6 mmol) and purified by silica gel column chromatography to afford the desired **Compound S22** (1.80 g, 71%) as a white solid.

^{1}H NMR (400 MHz, CDCl$_3$) δ 8.00 (dd, *J* = 8.5, 1.8 Hz, 2H), 7.59 (dd, *J* = 8.8, 1.7 Hz, 2H), 7.50 – 7.42 (m, 6H), 7.29 (td, *J* = 7.5, 1.5 Hz, 4H), 7.23 – 7.16 (m, 2H), 6.93 (dd, *J* = 8.8, 1.5 Hz, 2H), 4.50 (s, 1H), 3.94 – 3.89 (m, 2H), 3.81 (s, 3H), 3.63 – 3.51 (m, 2H), 1.36 (s, 9H).

^{13}C NMR (101 MHz, CDCl$_3$) δ 165.06, 159.08, 156.99, 142.12, 133.84, 129.73, 128.62, 127.58, 127.55, 127.34, 126.61, 125.52, 113.89, 78.38, 75.97, 65.78, 55.38, 35.25, 31.24.

HRMS (ESI-TOF) m/z: calc'd for $C_{34}H_{36}NO_3$ $[M+H]^+$: 506.2690; found 506.2685.

Compound S23

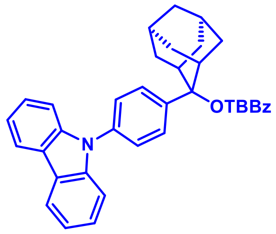

Prepared following the **General procedure 1** using 9-(4-bromophenyl)-9H-carbazole (1.1 equiv., 5.5 mmol), *n*-BuLi (2.5 M in hexanes, 1.1 equiv., 5.5 mmol), 2-adamantanone (1.0 equiv., 5 mmol), TBBzCl (1.2 equiv., 6 mmol) and purified by silica gel column chromatography to afford the desired

Compound S23 (2.41 g, 87%) as a white solid.

^{1}H NMR (400 MHz, CDCl$_3$) δ 8.18 – 8.09 (m, 2H), 8.00 (d, *J* = 8.5 Hz, 2H), 7.94 (d, *J* = 8.7 Hz, 2H), 7.56 (d, *J* = 8.4 Hz, 2H), 7.48 (dd, *J* = 8.3, 3.7 Hz, 4H), 7.43 – 7.37 (m, 2H), 7.28 (t, *J* = 7.5 Hz, 2H), 3.40 (s, 2H), 2.41 (d, *J* = 12.7 Hz, 2H), 2.02 – 1.78 (m, 10H), 1.35 (s, 9H).

^{13}C NMR (126 MHz, CDCl$_3$) δ 164.75, 156.38, 140.83, 139.54, 136.87, 129.54, 129.32, 129.03, 126.24, 125.96, 125.45, 123.48, 120.34, 120.00, 110.19, 86.34, 77.42, 37.77, 35.18, 34.65, 34.26, 33.62, 31.28, 27.53, 26.71.

HRMS (ESI-TOF) m/z: calc'd for : $C_{39}H_{40}NO_2$ [M+H]$^+$: 554.3054; found 554.3066.

Compound S24

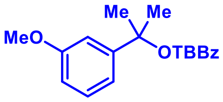

Prepared following the **General procedure 2** using 1-(3-methoxyphenyl)ethan-1-one (1.0 equiv., 5 mmol), MeMgBr (3.0 M in Et_2O, 1.1 equiv., 5.5 mmol), TBBzCl (1.2 equiv., 6 mmol) and purified by silica gel column chromatography to afford the desired **Compound S24** (0.85 g, 52%) as a pale yellow oil.

^{1}H NMR (400 MHz, CDCl$_3$) δ 8.06 (d, *J* = 8.5 Hz, 2H), 7.51 (d, *J* = 8.5 Hz, 2H), 7.31 (t, *J* = 8.0 Hz, 1H), 7.12 – 7.02 (m, 2H), 6.84 (dd, *J* = 8.3, 2.5 Hz, 1H), 3.82 (s, 3H), 1.96 (s, 6H), 1.40 (s, 9H).

^{13}C NMR (101 MHz, CDCl$_3$) δ 165.16, 159.57, 156.30, 147.83, 129.50, 129.41, 128.80, 125.31, 116.79, 111.61, 111.05, 81.78, 55.15, 35.07, 31.19, 28.86.

HRMS (ESI-TOF) m/z: calc'd for $C_{21}H_{26}NaO_3$ [M+Na]$^+$: 349.1775; found 349.1771.

Compound S25

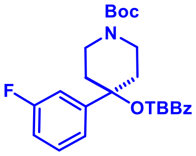

Prepared following the **General procedure 1** using 1-bromo-3-fluorobenzene (1.1 equiv., 5.5 mmol), *n*-BuLi (2.5 M in hexanes, 1.1 equiv., 5.5 mmol), tert-butyl 4-oxopiperidine-1-carboxylate (1.0 equiv., 5 mmol), TBBzCl (1.2 equiv., 6 mmol) and purified by silica gel column chromatography to afford the desired **Compound S25** (1.09 g, 48%) as a white solid.

^{1}H NMR (400 MHz, CDCl$_3$) δ 8.02 – 7.91 (m, 2H), 7.52 – 7.45 (m, 2H), 7.30 (q, *J* = 7.6 Hz, 1H), 7.18 (d, *J* = 7.8 Hz, 1H), 7.13 – 7.06 (m, 1H), 6.95 (td, *J* = 8.4, 2.5 Hz, 1H), 4.24 – 3.95 (m, 2H), 3.20 (t, *J* = 13.1 Hz, 2H), 2.64 (d, *J* = 13.6 Hz, 2H), 2.02 (td, *J* = 13.2, 5.1 Hz, 2H), 1.48 (s, 9H), 1.35 (s, 9H).

^{13}C NMR (101 MHz, CDCl$_3$) δ 164.72, 163.04 (d, *J* = 245.7 Hz), 157.09, 154.91, 146.94 (d, *J* = 6.7 Hz), 130.21 (d, *J* = 8.2 Hz), 129.58, 127.87, 125.62, 120.16 (d, *J* = 2.9 Hz), 114.55 (d, *J* = 21.0 Hz), 111.97 (d, *J* = 22.7 Hz), 80.17, 79.91, 39.81, 35.72, 35.23, 31.21, 28.54.

^{19}F NMR (376 MHz, CDCl$_3$) δ -112.38.

HRMS (ESI-TOF) m/z: calc'd for $C_{27}H_{34}FNNaO_4$ [M+Na]$^+$: 478.2365; found 478.2365.

Compound S26

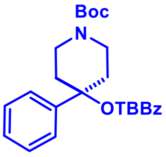

Prepared following the **General procedure 1** using bromobenzene (1.1 equiv., 5.5 mmol), *n*-BuLi (2.5 M in hexanes, 1.1 equiv., 5.5 mmol), tert-butyl 4-oxopiperidine-1-carboxylate (1.0 equiv., 5 mmol), TBBzCl (1.2 equiv., 6 mmol) and purified by silica gel column chromatography to afford the desired **Compound S26** (1.00 g, 46%) as a white solid.

^{1}H NMR (400 MHz, CDCl$_3$) δ 7.98 (d, *J* = 8.4 Hz, 2H), 7.47 (d, *J* = 8.4 Hz, 2H), 7.40 (d, *J* = 7.4 Hz, 2H), 7.33 (dd, *J* = 8.7, 6.7 Hz, 2H), 7.29 – 7.23 (m, 1H), 4.12 (m, 2H), 3.22 (t, *J* = 12.4 Hz, 2H), 2.72 – 2.60 (m, 2H), 2.15 – 2.00 (m, 2H), 1.47 (s, 9H), 1.35 (s, 9H).

^{13}C NMR (101 MHz, CDCl$_3$) δ 164.81, 156.90, 155.03, 144.13, 129.58, 128.67, 128.21, 127.66, 125.58, 124.56, 80.81, 79.87, 39.96, 35.82, 35.24, 31.25, 28.58.

HRMS (ESI-TOF) m/z: calc'd for $C_{27}H_{35}NNaO_4$ $[M+Na]^+$: 460.2459; found 460.2458.

Compound S27

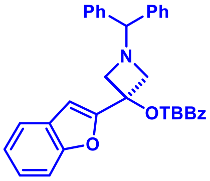

Prepared following the **General procedure 1** using benzofuran (1.1 equiv., 5.5 mmol), *n*-BuLi (2.5 M in hexanes, 1.1 equiv., 5.5 mmol), 1-benzhydrylazetidin-3-one (1.0 equiv., 5 mmol), TBBzCl (1.2 equiv., 6 mmol) and purified by silica gel column chromatography to afford the desired **Compound S27** (1.35 g, 49%) as a white solid.

^{1}H NMR (400 MHz, CDCl$_3$) δ 7.99 (d, *J* = 8.7 Hz, 2H), 7.57 (ddd, *J* = 7.4, 1.6, 0.7 Hz, 1H), 7.51 – 7.41 (m, 7H), 7.33 – 7.24 (m, 5H), 7.25 – 7.14 (m, 3H), 6.94 (d, *J* = 1.0 Hz, 1H), 4.52 (s, 1H), 4.08 (d, *J* = 9.8 Hz, 2H), 3.62 (d, *J* = 9.8 Hz, 2H), 1.34 (s, 9H).

^{13}C NMR (101 MHz, CDCl$_3$) δ 165.06, 157.23, 155.91, 154.93, 141.88, 129.86, 128.66, 128.35, 127.61, 127.43, 127.15, 125.55, 124.49, 122.98, 121.47, 111.52, 104.75, 78.16, 71.71, 63.74, 35.27, 31.24.

HRMS (ESI-TOF) m/z: calc'd for $C_{35}H_{34}NO_3$ $[M+H]^+$: 516.2534; found 516.2540.

Compound S28

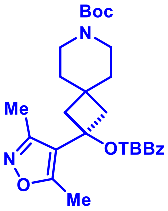

Prepared following the **General procedure 1** using 4-bromo-3,5-dimethylisoxazole (1.1 equiv., 5.5 mmol), *n*-BuLi (2.5 M in hexanes, 1.1 equiv., 5.5 mmol), tert-butyl 2-oxo-7-azaspiro[3.5]nonane-7-carboxylate (1.0 equiv., 5 mmol), TBBzCl (1.2 equiv., 6 mmol) and purified by silica gel column

chromatography to afford the desired **Compound S28** (0.99 g, 40%) as a white solid.

^{1}H NMR (400 MHz, CDCl$_3$) δ 7.89 (d, J = 8.5 Hz, 2H), 7.45 (d, J = 8.5 Hz, 2H), 3.33 (dt, J = 14.1, 5.5 Hz, 4H), 2.73 – 2.60 (m, 4H), 2.54 (s, 3H), 2.31 (s, 3H), 1.70 (t, J = 5.7 Hz, 2H), 1.50 (t, J = 5.6 Hz, 2H), 1.44 (s, 9H), 1.33 (s, 9H).

^{13}C NMR (101 MHz, CDCl$_3$) δ 167.12, 165.13, 159.21, 157.03, 155.01, 129.39, 127.83, 125.63, 116.08, 79.64, 73.76, 43.87, 37.99, 37.80, 35.25, 33.01, 31.24, 28.58, 13.17, 11.66.

HRMS (ESI-TOF) m/z: calc'd for $C_{29}H_{41}N_2O_5$ [M+H]$^+$: 497.3010; found 497.3011.

Compound S29

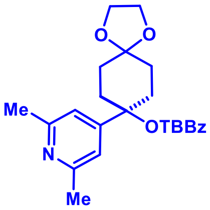

Prepared following the **General procedure 1** using 4-bromo-2,6-dimethylpyridine (1.1 equiv., 5.5 mmol), *n*-BuLi (2.5 M in hexanes, 1.1 equiv., 5.5 mmol), 1,4-dioxaspiro[4.5]decan-8-one (1.0 equiv., 5 mmol), TBBzCl (1.2 equiv., 6 mmol) and purified by silica gel column chromatography to afford the desired **Compound S29** (0.87 g, 41%) as a white solid.

^{1}H NMR (400 MHz, CDCl$_3$) δ 7.99 (d, J = 8.4 Hz, 2H), 7.47 (d, J = 8.5 Hz, 2H), 6.95 (s, 2H), 3.97 (s, 4H), 2.64 – 2.55 (m, 2H), 2.48 (s, 6H), 2.13 (td, J = 13.6, 3.6 Hz, 2H), 2.07 – 1.95 (m, 2H), 1.81 – 1.72 (m, 2H), 1.34 (s, 9H).

^{13}C NMR (101 MHz, CDCl$_3$) δ 164.81, 157.96, 157.00, 154.22, 129.61, 127.83, 125.59, 116.20, 107.80, 80.81, 64.58, 64.39, 35.20, 33.60, 31.18, 30.85, 24.63.

HRMS (ESI-TOF) m/z: calc'd for $C_{26}H_{34}NO_4$ [M+H]$^+$: 424.2483; found 424.2477.

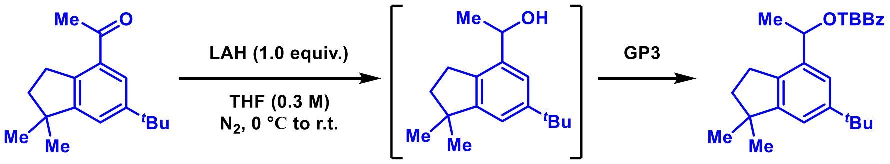

Under nitrogen atmosphere, to a solution of Celestolide (1.0 equiv., 5 mmol) in dry THF (0.3 M) was treated dropwise with a solution of LAH (1.1 equiv., 5 mmol in THF) at 0 °C. After stirring for 3 h, the reaction was quenched by the careful addition of saturated aqueous NaOH. The organic layer was separated. The aqueous layer was extracted with EtOAc (×3), and then the combined organic layer was washed with brine, dried over Na_2SO_4, filtrated, and removed under reduced pressure to afford the crude alcohol, which was used in next step without further purification.

Compound S30 was prepared following the **General procedure 3** using the crude alcohol, TEA (2.0 equiv., 10 mmol), DMAP (10 mol%, 0.5 mmol), TBBzCl (1.2 equiv., 6 mmol) and purified by silica gel column chromatography to afford the desired **Compound S30** (1.38 g, 68%, 2 steps) as a white solid.

Compound S30

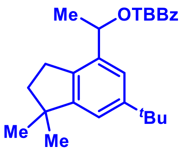

^{1}H NMR (400 MHz, CDCl$_3$) δ 8.03 (d, J = 8.4 Hz, 2H), 7.47 (d, J = 8.5 Hz, 2H), 7.37 (d, J = 1.8 Hz, 1H), 7.14 (d, J = 1.8 Hz, 1H), 6.23 (q, J = 6.6 Hz, 1H), 3.11 – 2.82 (m, 2H), 1.96 (t, J = 7.2 Hz, 2H), 1.66 (d, J = 6.5 Hz, 3H), 1.36 (d, J = 2.9 Hz, 18H), 1.28 (d, J = 2.5 Hz, 6H).
^{13}C NMR (126 MHz, CDCl$_3$) δ 166.01, 156.54, 153.02, 150.18, 137.35, 136.73, 129.62, 128.03, 125.44, 120.70, 118.56, 71.55, 43.98, 41.60, 35.20, 34.93, 31.80, 31.27, 28.89, 28.84, 28.16, 21.53.
HRMS (ESI-TOF) m/z: calc'd for $C_{28}H_{38}NaO_2$ [M+Na]$^+$: 429.2765; found 429.2764.

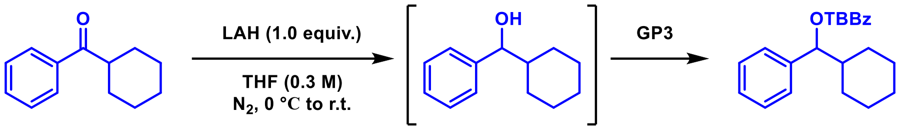

Under nitrogen atmosphere, to a solution of cyclohexyl(phenyl)methanone (1.0 equiv., 5 mmol) in dry THF (0.3 M) was treated dropwise with a solution of LAH (1.1 equiv., 5 mmol in THF) at 0 °C. After stirring for 3 h, the reaction was quenched by the careful addition of saturated aqueous NaOH. The organic layer was separated. The aqueous layer was extracted with EtOAc (×3), and then the combined organic layer was washed with brine, dried over Na_2SO_4, filtrated, and removed under reduced pressure to afford the crude cyclohexyl(phenyl)methanol, which was used in next step without further purification.
Compound S31 was prepared following the **General procedure 3** using the crude cyclohexyl(phenyl)methanol, TEA (2.0 equiv., 10 mmol), DMAP (10 mol%, 0.5 mmol), TBBzCl (1.2 equiv., 6 mmol) and purified by silica gel column chromatography to afford the desired **Compound S31**(1.08 g, 62%, 2 steps) as a white solid.

Compound S31

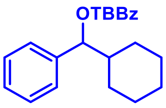

^{1}H NMR (500 MHz, CDCl$_3$) δ 8.03 (dd, J = 8.4, 1.8 Hz, 2H), 7.47 (dd, J = 8.5, 1.8 Hz, 2H), 7.38 – 7.34 (m, 2H), 7.34 – 7.31 (m, 2H), 7.28 – 7.23 (m, 1H), 5.73 (d, J = 7.3 Hz, 1H), 1.98 – 1.87 (m, 2H), 1.79 – 1.74 (m, 1H), 1.72 – 1.67 (m, 1H), 1.65 (d, J = 11.2 Hz, 1H), 1.53 – 1.46 (m, 1H), 1.34 (s, 9H), 1.27 – 1.12 (m, 4H), 1.03 (qd, J = 12.0, 3.4 Hz, 1H).
^{13}C NMR (126 MHz, CDCl$_3$) δ 165.99, 156.66, 139.99, 129.65, 128.30, 127.95, 127.76, 127.14, 125.49, 80.65, 43.46, 35.21, 31.27, 29.32, 28.96, 26.45, 26.10, 26.04.
HRMS (ESI-TOF) m/z: calc'd for $C_{24}H_{30}NaO_2$ [M+Na]$^+$: 373.2138; found 373.2132.

Compound S32

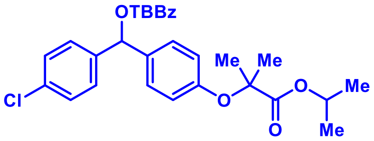

Prepared following the **General procedure 3** using isopropyl 2-(4-((4-chlorophenyl)(hydroxy)methyl)phenoxy)-2-methylpropanoa (1.0 equiv., 5.0 mmol) (*6*), TEA (2.0 equiv., 10 mmol), DMAP (10 mol%, 0.5 mmol), TBBzCl (1.2 equiv., 6 mmol) and purified by silica gel column chromatography to afford the desired **Compound S32** (1.70 g, 65%) as a pale yellow oil.

^{1}H NMR (400 MHz, CDCl$_3$) δ 8.04 (d, *J* = 8.3 Hz, 2H), 7.47 (d, *J* = 8.2 Hz, 2H), 7.36 – 7.27 (m, 4H), 7.25 (d, *J* = 8.5 Hz, 2H), 7.02 (s, 1H), 6.81 (d, *J* = 8.6 Hz, 2H), 5.06 (hept, *J* = 6.3 Hz, 1H), 1.57 (s, 6H), 1.33 (s, 9H), 1.19 (dd, *J* = 6.2, 1.4 Hz, 6H).

^{13}C NMR (101 MHz, CDCl$_3$) δ 173.68, 165.60, 157.10, 155.58, 139.22, 133.77, 133.25, 129.77, 128.78, 128.57, 128.30, 127.38, 125.59, 118.84, 79.26, 76.17, 69.10, 35.25, 31.23, 25.53, 21.66.

HRMS (ESI-TOF) m/z: calc'd for $C_{31}H_{35}ClNaO_5$ $[M+Na]^+$: 545.2066; found 545.2070.

Compound S33

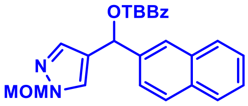

Prepared following the **General procedure 1** using 4-bromo-1-(methoxymethyl)-1H-pyrazole (1.1 equiv., 5.5 mmol), *n*-BuLi (2.5 M in hexanes, 1.1 equiv., 5.5 mmol), 2-naphthaldehyde (1.0 equiv., 5 mmol), TBBzCl (1.2 equiv., 6 mmol) and purified by silica gel column chromatography to afford the desired **Compound S33** (0.77 g, 36%) as a white solid.

^{1}H NMR (500 MHz, CDCl$_3$) δ 8.07 (d, *J* = 8.4 Hz, 2H), 7.97 (s, 1H), 7.86 (td, *J* = 7.8, 7.0, 4.8 Hz, 3H), 7.59 (dd, *J* = 8.6, 1.8 Hz, 1H), 7.56 (s, 1H), 7.52 – 7.46 (m, 5H), 7.28 (s, 1H), 5.33 (s, 2H), 3.32 (s, 3H), 1.34 (s, 9H).

^{13}C NMR (126 MHz, CDCl$_3$) δ 165.88, 157.07, 139.63, 137.20, 133.24, 133.22, 129.79, 129.40, 128.64, 128.28, 127.84, 127.48, 126.51, 126.44, 125.89, 125.58, 124.86, 123.44, 82.15, 70.55, 56.99, 35.25, 31.23.

HRMS (ESI-TOF) m/z: calc'd for $C_{27}H_{29}N_2O_3$ $[M+H]^+$: 429.2173; found 429.2176.

Compound S34

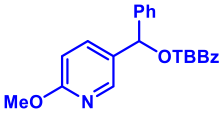

Prepared following the **General procedure 1** using 5-bromo-2-methoxypyridine (1.1 equiv., 5.5 mmol), *n*-BuLi (2.5 M in hexanes, 1.1 equiv., 5.5 mmol), benzaldehyde (1.0 equiv., 5 mmol), TBBzCl (1.2 equiv., 6 mmol) and purified by silica gel column chromatography to afford the desired **Compound S34** (0.98 g, 52%) as a white solid.

^{1}H NMR (400 MHz, CDCl$_3$) δ 8.30 (d, *J* = 2.5 Hz, 1H), 8.13 – 8.03 (m, 2H), 7.64 – 7.58 (m, 1H), 7.52 – 7.48 (m, 2H), 7.45 (d, *J* = 7.3 Hz, 2H), 7.38 (td, *J* = 7.3, 1.2 Hz, 2H), 7.34 – 7.27 (m, 1H), 7.12 (s, 1H), 6.74 (d, *J* = 8.6 Hz, 1H), 3.94 (s, 3H), 1.35 (s, 9H).

^{13}C NMR (101 MHz, CDCl$_3$) δ 165.53, 163.97, 157.06, 146.14, 139.85, 138.05, 129.75, 129.06, 128.73, 128.13, 127.29, 126.85, 125.54, 111.06, 74.96, 53.56, 35.18, 31.17.
HRMS (ESI-TOF) m/z: calc'd for $C_{24}H_{26}NO_3$ $[M+H]^+$: 376.1908; found 376.1903.

Compound S35

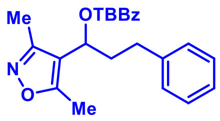

Prepared following the **General procedure 1** using 4-bromo-3,5-dimethylisoxazole (1.1 equiv., 5.5 mmol), *n*-BuLi (2.5 M in hexanes, 1.1 equiv., 5.5 mmol), 3-phenylpropanal (1.0 equiv., 5 mmol), TBBzCl (1.2 equiv., 6 mmol) and purified by silica gel column chromatography to afford the desired **Compound S35** (1.01 g, 52%) as a pale yellow oil.
^{1}H NMR (400 MHz, CDCl$_3$) δ 7.97 (d, *J* = 8.4 Hz, 2H), 7.48 (d, *J* = 8.4 Hz, 2H), 7.30 (t, *J* = 7.5 Hz, 2H), 7.24 – 7.20 (m, 1H), 7.18 (d, *J* = 8.3 Hz, 2H), 5.86 (dd, *J* = 8.0, 7.0 Hz, 1H), 2.79 – 2.64 (m, 2H), 2.58 – 2.45 (m, 1H), 2.42 (s, 3H), 2.38 (s, 3H), 2.27 – 2.12 (m, 1H), 1.35 (s, 9H).
^{13}C NMR (101 MHz, CDCl$_3$) δ 167.19, 165.72, 158.75, 157.06, 140.46, 129.50, 128.69, 128.37, 127.16, 126.38, 125.61, 113.25, 67.33, 35.57, 35.20, 32.00, 31.18, 11.52, 11.13.
HRMS (ESI-TOF) m/z: calc'd for $C_{25}H_{30}NO_3$ $[M+H]^+$: 392.2221; found 392.2219.

Compound S36

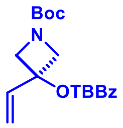

Prepared following the **General procedure 2** using tert-butyl 3-oxoazetidine-1-carboxylate (1.0 equiv., 5 mmol), vinylmagnesium bromide (1.0 M in THF, 1.1 equiv., 5.5 mmol), TBBzCl (1.2 equiv., 6 mmol) and purified by silica gel column chromatography to afford the desired **Compound S36** (1.47 g, 82%) as a white solid.
^{1}H NMR (400 MHz, CDCl$_3$) δ 7.97 (d, *J* = 8.4 Hz, 2H), 7.47 (d, *J* = 8.5 Hz, 2H), 6.24 (dd, *J* = 17.4, 10.9 Hz, 1H), 5.43 – 5.20 (m, 2H), 4.39 – 4.05 (m, 4H), 1.44 (s, 9H), 1.34 (s, 9H).
^{13}C NMR (101 MHz, CDCl$_3$) δ 165.14, 157.37, 156.44, 136.55, 129.74, 126.94, 125.61, 115.76, 80.12, 75.17, 60.51, 35.28, 31.21, 28.45.
HRMS (ESI-TOF) m/z: calc'd for $C_{21}H_{29}NNaO_4$ $[M+Na]^+$: 382.1989; found 382.1989.

Compound S37

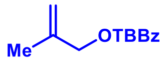

Prepared following the **General procedure 3** using 2-methylprop-2-en-1-ol (1.0 equiv., 5.0 mmol), TEA (2.0 equiv., 10 mmol), DMAP (10 mol%, 0.5 mmol), TBBzCl (1.2 equiv., 6 mmol) and purified by silica gel column chromatography to afford the desired **Compound S37** (0.84 g, 72%) as a pale yellow oil.
^{1}H NMR (400 MHz, CDCl$_3$) δ 8.02 (d, *J* = 8.5 Hz, 2H), 7.47 (d, *J* = 8.5 Hz, 2H), 5.08 (d, *J* = 1.4 Hz, 1H), 4.98 (d, *J* = 1.4 Hz, 1H), 4.75 (s, 2H), 1.84 (s, 3H), 1.34 (s, 9H).

^{13}C NMR (101 MHz, CDCl$_3$) δ 166.34, 156.72, 140.23, 129.62, 127.51, 125.46, 112.82, 67.97, 35.17, 31.21, 19.66.
HRMS (ESI-TOF) m/z: calc'd for $C_{15}H_{21}O_2$ $[M+H]^+$: 233.1537; found 233.1535.

Compound S38

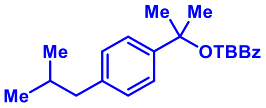

Prepared following the **General procedure 2** using 1-(4-isobutylphenyl)ethan-1-one (1.0 equiv., 5 mmol), MeMgBr (3.0 M in Et_2O, 1.1 equiv., 5.5 mmol), TBBzCl (1.2 equiv., 6 mmol) and purified by silica gel column chromatography to afford the desired **Compound S38** (1.00 g, 57%) as a pale yellow oil.
^{1}H NMR (400 MHz, CDCl$_3$) δ 8.04 (d, *J* = 8.5 Hz, 2H), 7.50 (d, *J* = 8.4 Hz, 2H), 7.39 (d, *J* = 8.0 Hz, 2H), 7.15 (d, *J* = 8.1 Hz, 2H), 2.49 (d, *J* = 7.2 Hz, 2H), 1.95 (s, 6H), 1.88 (dt, *J* = 13.5, 6.7 Hz, 1H), 1.38 (s, 9H), 0.95 (d, *J* = 6.6 Hz, 6H).
^{13}C NMR (101 MHz, CDCl$_3$) δ 165.23, 156.25, 143.33, 140.36, 129.55, 129.53, 129.09, 125.32, 124.19, 82.07, 45.14, 35.11, 31.24, 30.21, 28.90, 22.57.
HRMS (ESI-TOF) m/z: calc'd for $C_{24}H_{32}NaO_2$ $[M+Na]^+$: 375.2295; found 375.2295.

Compound S39

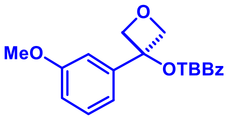

Prepared following the **General procedure 1** using 1-bromo-3-methoxybenzene (1.1 equiv., 5.5 mmol), *n*-BuLi (2.5 M in hexanes, 1.1 equiv., 5.5 mmol), oxetan-3-one (1.0 equiv., 5 mmol), TBBzCl (1.2 equiv., 6 mmol) and purified by silica gel column chromatography to afford the desired **Compound S39** (1.07 g, 63%) as a pale yellow oil.
^{1}H NMR (400 MHz, CDCl$_3$) δ 8.04 (d, *J* = 8.4 Hz, 2H), 7.50 (d, *J* = 8.5 Hz, 2H), 7.31 (t, *J* = 8.0 Hz, 1H), 7.14 (ddd, *J* = 7.8, 1.9, 1.0 Hz, 1H), 7.13 – 7.07 (m, 1H), 6.86 (ddd, *J* = 8.3, 2.6, 0.9 Hz, 1H), 5.20 (d, *J* = 8.1 Hz, 2H), 5.03 (d, *J* = 8.1 Hz, 2H), 3.80 (s, 3H), 1.36 (s, 9H).
^{13}C NMR (101 MHz, CDCl$_3$) δ 165.09, 160.04, 157.48, 141.44, 129.94, 129.87, 126.83, 125.69, 116.87, 113.14, 110.85, 82.54, 80.30, 55.40, 35.31, 31.23.
HRMS (ESI-TOF) m/z: calc'd for $C_{21}H_{25}O_4$ $[M+H]^+$: 341.1748; found 341.1741.

The Stability of Compound 4

Compound 4 can be left at room temperature under air atmosphere for prolonged periods of time (>100 days) without any change in appearance and purity.

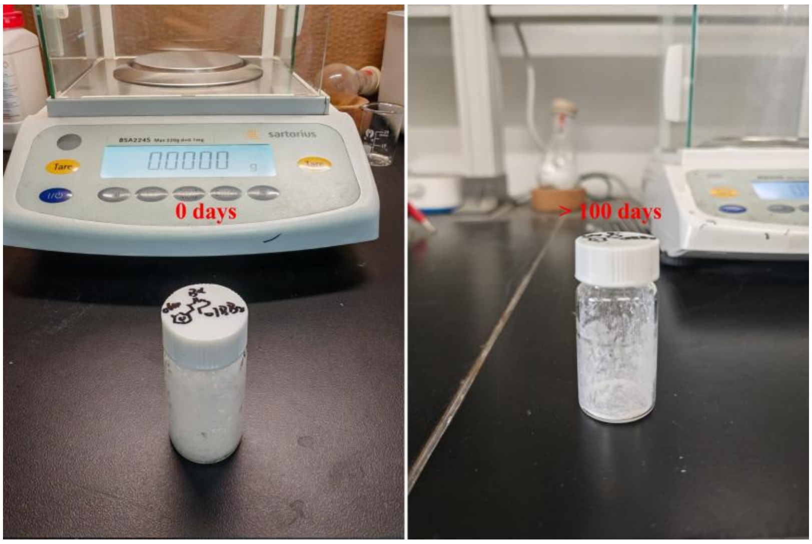

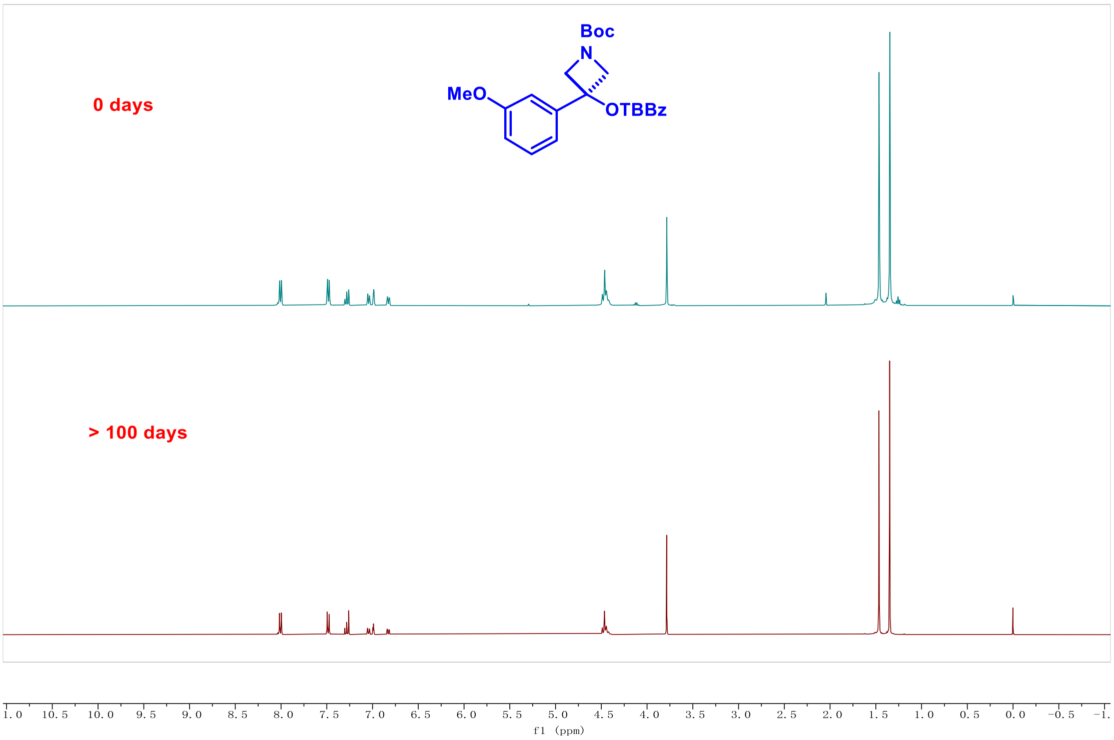

Fig. S1. No change in appearance and ^{1}H NMR spectrum of **Compound 4** after 100 days.

Reaction Condition Optimization

All optimization reactions were carried out on a 0.2 mmol scale.

Table S2. Evaluation of activating groups.

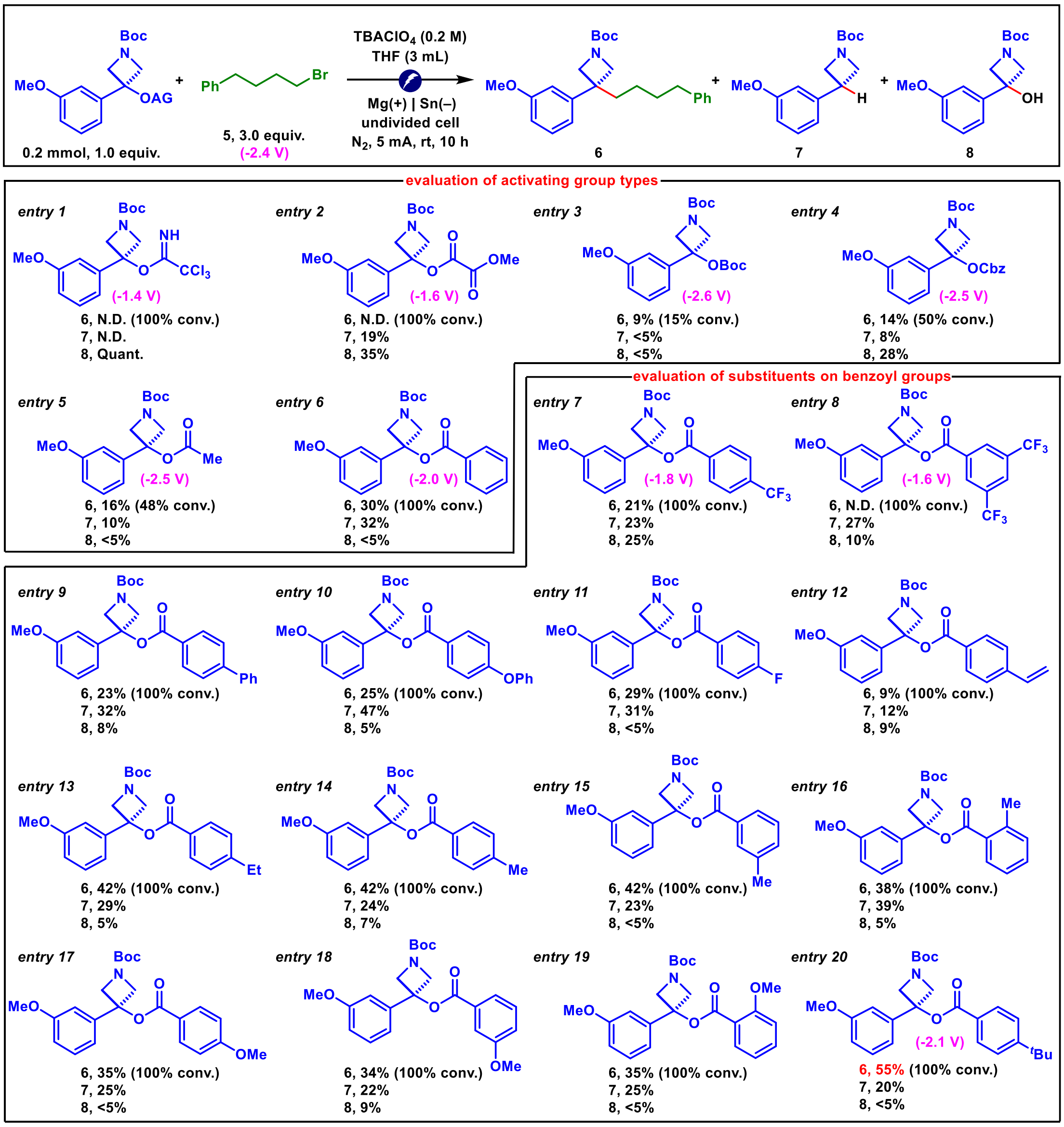

Yields of 6 and 7 were determined by ^{1}H-NMR, while the yields of 8 were determined by GC-MS, both using 1,3,5-trimethoxybenzene as an internal standard. All potentials were measured by cyclic voltammetry using a glassy carbon working electrode, a platinum wire counter electrode, and an Ag/AgCl reference electrode in THF containing 0.1 M $TBAClO_4$ at room temperature. Values correspond to the onset potentials of the reduction waves (see the Cyclic Voltammetry Studies for details). AG = activating group. N.D. = not detected. conv. = conversion. Quant. = quantitative.

Table S3. Evaluation of electrode material.

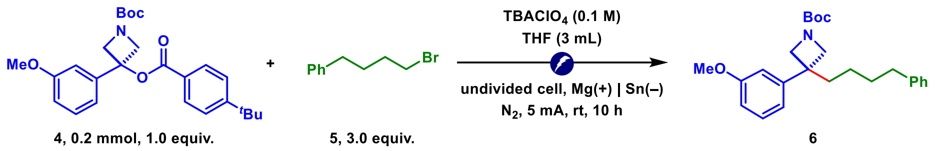

Entry	Deviation from above	Yield of 6 (%)
1	none	68
2	Zn(+) instead of Mg(+)	N.D.
3	Al(+) instead of Mg(+)	N.D.
4	C(+) instead of Mg(+)	N.D.
5	Ni(–) instead of Sn(–)	8
6	Cu(–) instead of Sn(–)	40
7	Pt(–) instead of Sn(–)	13
8	C(–) instead of Sn(–)	45
9	Stainless Steel(–) instead of Sn(–)	<5
10	Zn(–) instead of Sn(–)	<5

Yield was determined by ^{1}H-NMR using 1,3,5-trimethoxybenzene as internal standard.

Table S4. Evaluation of solvent.

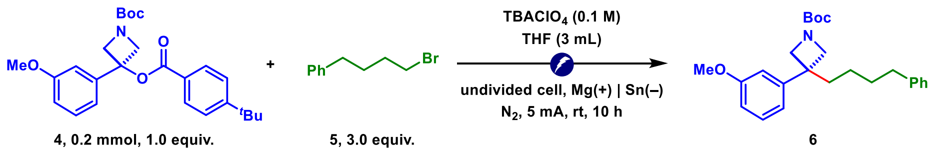

Entry	Deviation from above	Yield of 6 (%)
1	none	68
2	DCM instead of THF	N.D.
3	DCE instead of THF	N.D.
4	NMP instead of THF	22
5	DMF instead of THF	N.D.
6	MeCN instead of THF	N.D.
7	DME instead of THF	37
8	DMSO instead of THF	N.D.
9	EA instead of THF	N.D.

Yield was determined by ^{1}H-NMR using 1,3,5-trimethoxybenzene as internal standard.

Table S5. Evaluation of electrolyte and electrolyte concentration.

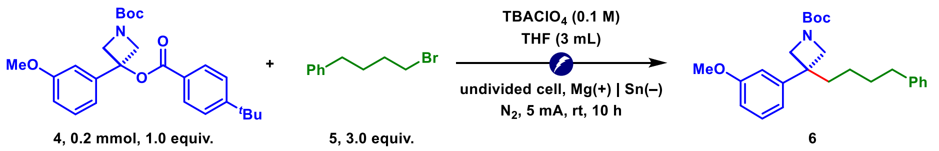

Entry	Deviation from above	Yield of 6 (%)
1	none	68
2	TBAOTf instead of $TBAClO_4$	42
3	$TBABF_4$ instead of $TBAClO_4$	37
4	$TBAPF_6$ instead of $TBAClO_4$	N.D.
5	$TBAClO_4$ (0.05 M)	64
6	$TBAClO_4$ (0.2 M)	55
7	$TBAClO_4$ (0.3 M)	54

Yield was determined by ^{1}H-NMR using 1,3,5-trimethoxybenzene as internal standard. TBACl, TBABr, TBAI and TBAOAc exhibited incompatibility with this system owing to their insufficient solubility under the experimental conditions.

Table S6. Evaluation of other parameters.

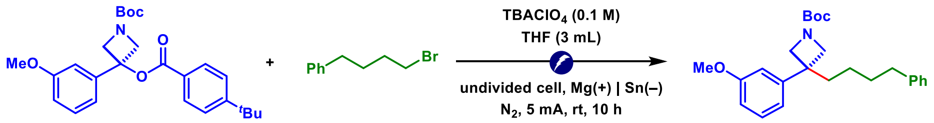

4, 0.2 mmol, 1.0 equiv. 5, 3.0 equiv. 6

Entry	Deviation from above	Yield of 6 (%)
1	none	68
2	4 (0.1 mmol)	50
3	4 (0.3 mmol)	66
4	5 (2.0 equiv.)	43
5	air instead of N_2	31
6	without electricity	N.D.
7	Mg powder/tunings instead of electricity	N.D.
8	Passing 0.2 F/mol of charge instead of 5 mA, 10 h	<5

Yield was determined by ^{1}H-NMR using 1,3,5-trimethoxybenzene as internal standard.

General Procedure for 0.2 mmol Scale Reaction

General Procedure A: An oven-dried 5 mL electrochemical cell equipped with a stir bar, a magnesium plate anode, and a tin plate cathode was charged with activated benzyl alcohol (1.0 equiv., 0.2 mmol) and $TBAClO_4$ (0.1 M). The cell was evacuated and back-filled with N_2 for three times. A solution of alkyl bromide or chlorosilane (3.0 equiv., 0.6 mmol) in dry THF (3.0 mL) was then added *via* syringe under nitrogen. After pre-stirring for 5 min, the mixture was electrolyzed at a constant current of 5 mA (current density = 1.18 mA/cm^2) for 10 h at room temperature. Upon completion, the reaction mixture was diluted with EtOAc and filtered through a short plug of silica gel. The filtrate was concentrated under reduced pressure, and the residue was purified by silica gel column chromatography or preparative thin layer chromatography (PTLC) to afford the desired product.

General Procedure B: An oven-dried 5 mL electrochemical cell equipped with a stir bar, a magnesium plate anode, and a tin plate cathode was charged with activated benzyl alcohol (1.0 equiv., 0.2 mmol) and $TBAClO_4$ (0.1 M). The cell was evacuated and back-filled with CO_2 for three times, and dry DMF (3.0 mL) was added by syringe. After pre-stirring for 5 min, the mixture was electrolyzed at a constant current of 10 mA (current density = 1.18 mA/cm^2) for 10 h at room temperature. Upon completion, the reaction mixture was acidified with 1 M HCl and extracted with EtOAc (3 ×). The combined organic layers were washed with saturated brine (3 ×), dried over Na_2SO_4, filtered, and concentrated under reduced pressure. The crude product was purified by reverse-phase column chromatography or preparative thin-layer chromatography (PTLC) to afford the desired product.

Graphical Guide

Representative photographs were taken from the reaction between **Compound S37** and **Compound 5** (Following **General Procedure A**)

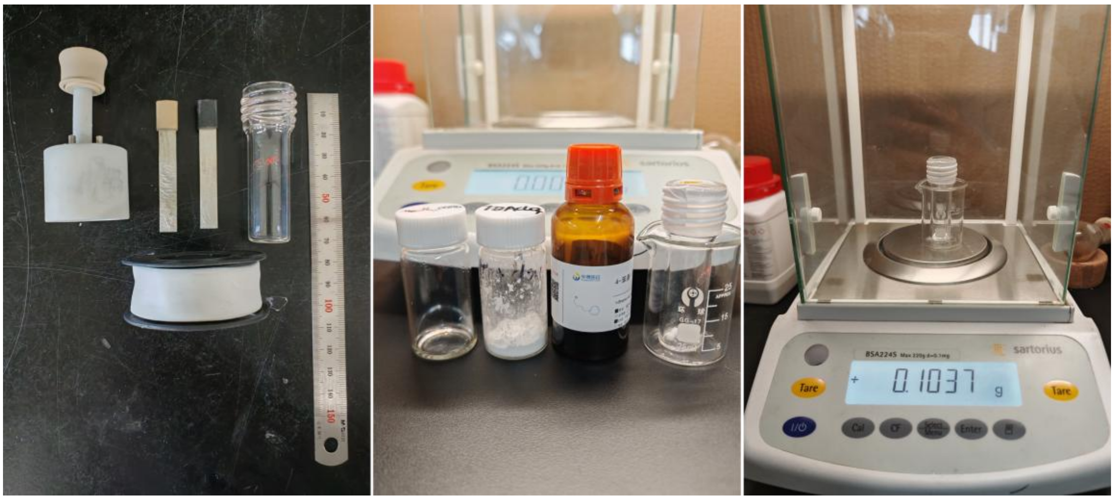

Left: Electrochemical cell (5 mL), Mg plate (left side), Sn plate (right side) and Teflon tape (keep a good sealing during the reaction). **Center:** All reagents for this reaction. **Right:** $TBAClO_4$ (0.1 M).

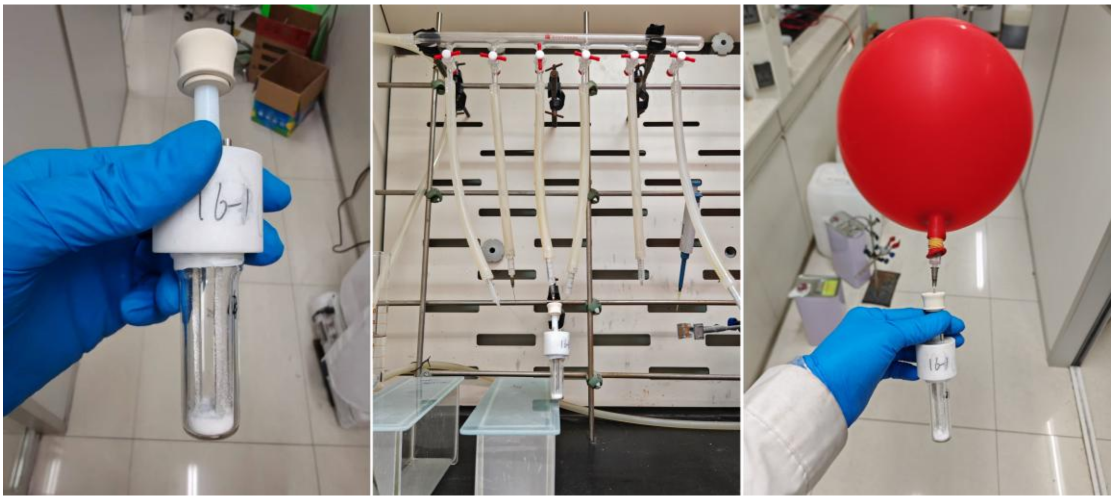

Left: The cap was tightly screwed into the vial. **Center:** The reaction vessel was connected to a vacuum line through a needle. **Right:** Backfilled with a nitrogen balloon and repeated this cycle for three times.

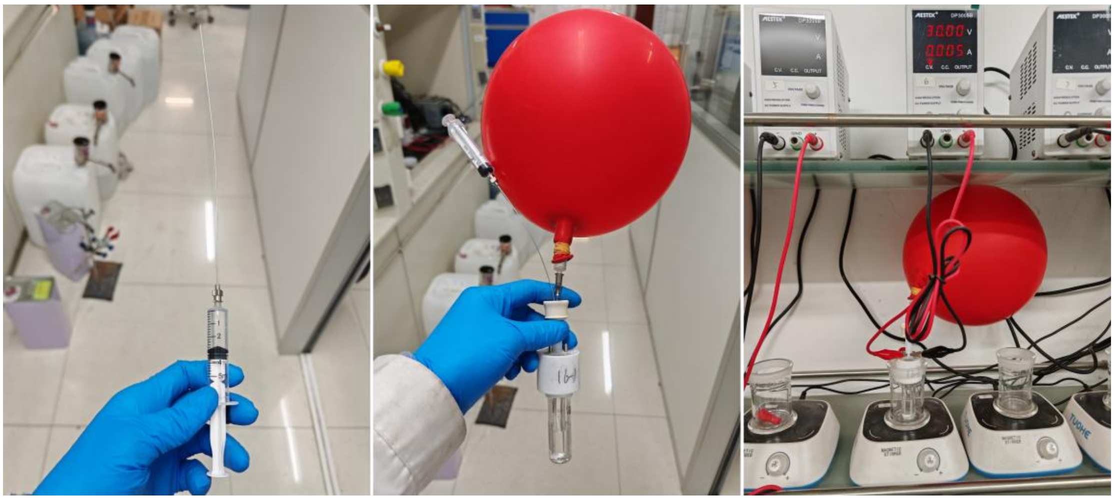

Left: Compound S37 and **Compound 5** were all dissolved in dry THF (3 mL). **Center:** Added *via* syringe under nitrogen atmosphere. **Right:** Pre-stirring the resulting mixture for 5 minutes and then start the reaction at a constant current of 5 mA.

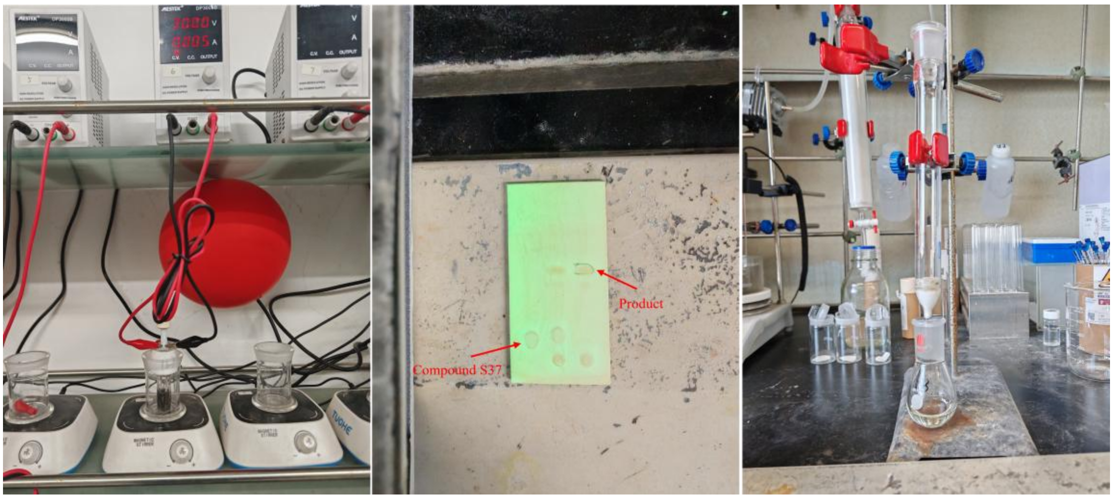

Left: Reaction completed. **Center:** The crude reaction mixture was monitored by TLC (PE), with visualization under UV light and $KMnO_4$. **Right:** The reaction mixture was filtered through a thin layer of silica gel.

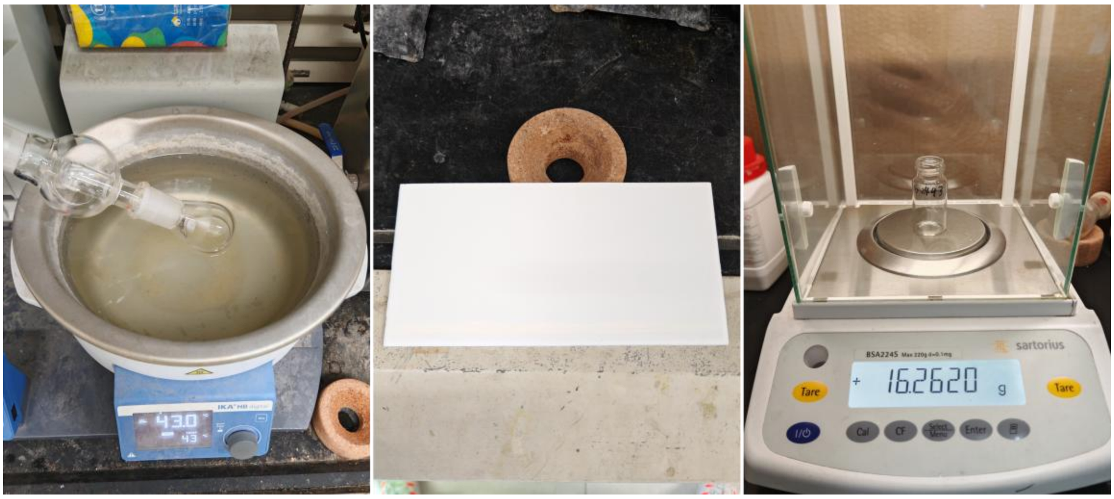

Left: Concentrating under reduced pressure. **Center:** Purified by PTLC (PE). **Right:** Isolated product as a colorless oil (17.7 mg, 47%).

Further details on the electrochemical cell setup

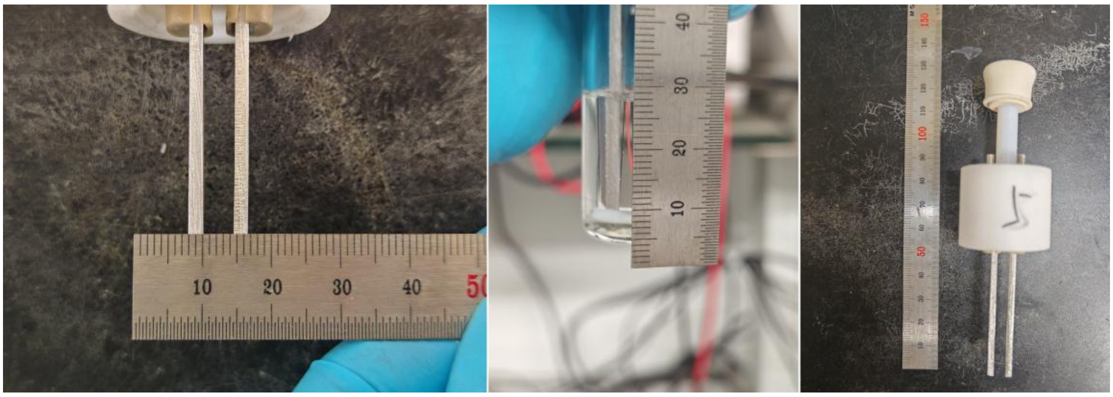

Left: Interelectrode spacing (~4.5 mm). **Center**: Immersed electrode depth (~18 mm). **Right**: Ruler used for measurement (total length 150 mm).

Experimental Procedures and Characterization Data of Products

Compound 6

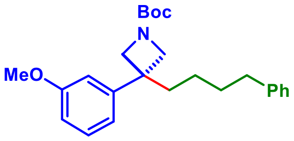

Following **General Procedure A**, electrolysis was conducted for 10 h. Purification by PTLC (silica, 10:1, PE: EA) afforded 45.1 mg (57%) of the title **Compound 6** as a colorless oil.

^{1}H NMR (500 MHz, CDCl$_3$) δ 7.25 – 7.20 (m, 3H), 7.17 – 7.12 (m, 1H), 7.10 – 7.07 (m, 2H), 6.76 (ddd, *J* = 8.2, 2.5, 0.8 Hz, 1H), 6.66 (dt, *J* = 7.7, 1.2 Hz, 1H), 6.60 (t, *J* = 2.1 Hz, 1H), 4.14 (d, *J* = 8.2 Hz, 2H), 3.91 (d, *J* = 8.1 Hz, 2H), 3.80 (s, 3H), 2.51 (dd, *J* = 9.0, 6.8 Hz, 2H), 1.95 – 1.87 (m, 2H), 1.53 (p, *J* = 7.8 Hz, 2H), 1.42 (s, 9H), 1.15 (p, *J* = 7.6 Hz, 2H).

^{13}C NMR (101 MHz, CDCl$_3$) δ 159.74, 156.68, 147.10, 142.55, 129.52, 128.43, 128.40, 125.80, 118.59, 112.46, 111.28, 79.56, 59.22, 55.38, 42.15, 42.12, 35.88, 31.79, 28.54, 24.32.

HRMS (ESI-TOF) m/z: calc'd for $C_{25}H_{33}NNaO_3$ $[M+Na]^+$: 418.2353; found 418.2352.

Compound 9

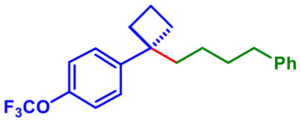

Following **General Procedure A**, electrolysis was conducted for 10 h. Purification by PTLC (silica, 50:1, PE: EA) afforded 40.4 mg (58%) of the title **Compound 9** as a colorless oil.

^{1}H NMR (500 MHz, CDCl$_3$) δ 7.36 – 7.27 (m, 2H), 7.25 – 7.08 (m, 7H), 2.55 (dd, *J* = 8.9, 6.7 Hz, 2H), 2.41 – 2.31 (m, 2H), 2.21 – 2.05 (m, 3H), 1.91 – 1.79 (m, 3H), 1.58 – 1.47 (m, 2H), 1.17 – 1.03 (m, 2H).

^{13}C NMR (126 MHz, CDCl$_3$) δ 149.38, 146.86 (q, *J* = 2.1 Hz), 142.79, 128.45, 128.35, 127.17, 125.72, 120.70 (q, *J* = 256.4 Hz), 120.51, 46.33, 42.40, 35.93, 32.98, 32.01, 24.32, 16.07.

^{19}F NMR (471 MHz, CDCl$_3$) δ -57.84.

HRMS (ESI-TOF) m/z: calc'd for $C_{21}H_{24}F_3O$ $[M+H]^+$: 349.1774; found 349.1773.

Compound 10

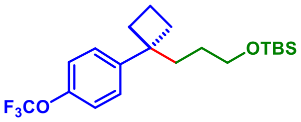

Following **General Procedure A**, electrolysis was conducted for 10 h. Purification by PTLC (silica, 50:1, PE: EA) afforded 51.3 mg (66%) of the title **Compound 10** as a colorless oil.

^{1}H NMR (500 MHz, CDCl$_3$) δ 7.14 – 7.08 (m, 4H), 3.49 (t, *J* = 6.5 Hz, 2H), 2.38 – 2.26 (m, 2H), 2.18 – 1.99 (m, 3H), 1.88 – 1.75 (m, 3H), 1.26 – 1.18 (m, 2H), 0.85 (s, 9H), -0.01 (s, 6H).

^{13}C NMR (126 MHz, CDCl$_3$) δ 149.24, 146.92 (q, *J* = 1.7 Hz), 127.18, 120.70 (q, *J* = 256.3 Hz), 120.58, 63.48, 46.06, 38.69, 32.94, 28.19, 26.07, 18.45, 16.02, -5.17.

^{19}F NMR (471 MHz, CDCl$_3$) δ -57.90.

HRMS (ESI-TOF) m/z: calc'd for $C_{20}H_{32}F_3O_2Si$ $[M+H]^+$: 389.2119; found 389.2109.

Compound 11

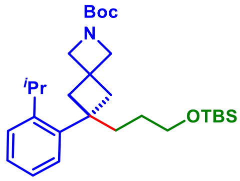

Following **General Procedure A**, electrolysis was conducted for 10 h. Purification by PTLC (silica, 10:1, PE: EA) afforded 68.3 mg (70%) of the title **Compound 11** as a colorless oil.

^{1}H NMR (400 MHz, CDCl$_3$) δ 7.23 (dd, J = 7.8, 1.5 Hz, 1H), 7.16 (td, J = 7.5, 1.4 Hz, 1H), 7.07 (td, J = 7.5, 1.5 Hz, 1H), 6.89 (dd, J = 7.7, 1.4 Hz, 1H), 4.07 (s, 2H), 3.79 (s, 2H), 3.47 (t, J = 6.2 Hz, 2H), 2.94 (p, J = 6.8 Hz, 1H), 2.64 – 2.46 (m, 4H), 1.74 (s, 2H), 1.43 (s, 9H), 1.19 (br, 8H), 0.86 (s, 9H), -0.01 (s, 6H).

^{13}C NMR (101 MHz, CDCl$_3$) δ 156.26, 146.68, 144.52, 127.46, 126.75, 126.46, 125.04, 79.38, 63.33, 47.64, 43.77, 42.36, 37.40, 33.01, 28.89, 28.54, 26.08, 24.87, 24.59, 18.44, -5.20.

HRMS (ESI-TOF) m/z: calc'd for $C_{29}H_{49}NNaO_3Si$ $[M+Na]^+$: 510.3374; found 510.3368.

Compound 12

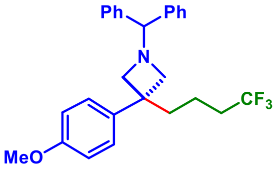

Following **General Procedure A**, electrolysis was conducted for 10 h. Purification by PTLC (silica, 20:1, PE: EA) afforded 46.6 mg (53%) of the title **Compound 12** as a colorless oil.

^{1}H NMR (400 MHz, CDCl$_3$) δ 7.41 (d, J = 7.5 Hz, 4H), 7.27 (t, J = 7.3 Hz, 4H), 7.18 (t, J = 7.3 Hz, 2H), 6.91 (d, J = 8.6 Hz, 2H), 6.83 (d, J = 8.6 Hz, 2H), 4.34 (s, 1H), 3.79 (s, 3H), 3.38 (d, J = 7.0 Hz, 2H), 3.23 (d, J = 7.0 Hz, 2H), 2.12 – 1.90 (m, 4H), 1.43 – 1.32 (m, 2H).

^{13}C NMR (101 MHz, CDCl$_3$) δ 157.85, 142.54, 139.06, 128.56, 127.61, 127.21, 126.90, 113.86, 78.12, 63.69, 55.39, 41.21, 41.04, 34.13 (q, J = 28.2 Hz), 17.68 (q, J = 2.9, 2.4 Hz).

^{19}F NMR (376 MHz, CDCl$_3$) δ -66.27.

HRMS (ESI-TOF) m/z: calc'd for $C_{27}H_{29}F_3NO$ $[M+H]^+$: 440.2196; found 440.2190.

Compound 13

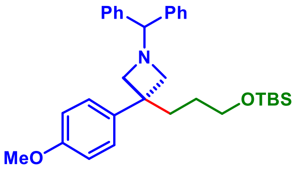

Following **General Procedure A**, electrolysis was conducted for 10 h. Purification by PTLC (silica, 20:1, PE: EA) afforded 44.1 mg (44%) of the title **Compound 13** as a colorless oil.

^{1}H NMR (500 MHz, CDCl$_3$) δ 7.42 (d, J = 7.6 Hz, 4H), 7.29 – 7.24 (m, 4H), 7.18 (d, J = 7.3 Hz, 2H), 6.94 (d, J = 8.6 Hz, 2H), 6.82 (d, J = 8.4 Hz, 2H), 4.35 (s, 1H), 3.79 (s, 3H), 3.53 (t, J = 6.5 Hz, 2H), 3.37 (d, J = 6.9 Hz, 2H), 3.23 (d, J = 6.9 Hz, 2H), 2.04 – 1.98 (m, 2H), 1.38 – 1.31 (m, 2H), 0.89 (s, 9H), 0.02 (s, 6H).

^{13}C NMR (126 MHz, CDCl$_3$) δ 157.67, 142.80, 139.89, 128.50, 127.64, 127.11, 127.02, 113.69, 78.12, 63.91, 63.49, 55.41, 41.15, 38.22, 28.49, 26.12, 18.48, -5.10.
HRMS (ESI-TOF) m/z: calc'd for $C_{32}H_{44}NO_2Si$ [M+H]$^+$: 502.3136; found 502.3140.

Compound 14

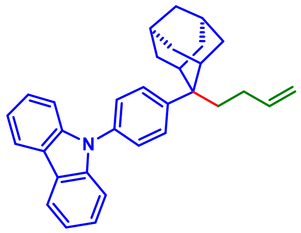

Following **General Procedure A**, electrolysis was conducted for 10 h. Purification by PTLC (silica, 50:1, PE: EA) afforded 54.4 mg (63%) of the title **Compound 14** as a white solid.
^{1}H NMR (500 MHz, CDCl$_3$) δ 8.16 (d, *J* = 7.7 Hz, 2H), 7.51 (s, 4H), 7.48 – 7.40 (m, 4H), 7.32 – 7.27 (m, 2H), 5.74 (ddt, *J* = 16.7, 10.2, 6.4 Hz, 1H), 5.00 – 4.87 (m, 2H), 2.47 (s, 2H), 2.35 – 2.24 (m, 2H), 2.02 – 1.96 (m, 3H), 1.89 – 1.73 (m, 9H), 1.65 (d, *J* = 12.7 Hz, 2H).
^{13}C NMR (126 MHz, CDCl$_3$) δ 146.77, 141.12, 139.62, 134.60, 128.39, 126.47, 125.95, 123.41, 120.38, 119.86, 114.09, 110.07, 46.28, 39.54, 39.00, 34.09, 32.94, 32.90, 28.53, 27.62, 27.40.
HRMS (ESI-TOF) m/z: calc'd for $C_{32}H_{34}N$ [M+H]$^+$: 432.2686; found 432.2683.

Compound 15

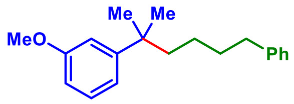

Following **General Procedure A**, electrolysis was conducted for 10 h. Purification by PTLC (silica, 100:1, PE: EA) afforded 27.1 mg (48%) of the title **Compound 15** as a colorless oil.
^{1}H NMR (400 MHz, CDCl$_3$) δ 7.33 – 7.27 (m, 3H), 7.22 – 7.19 (m, 1H), 7.16 (d, *J* = 7.7 Hz, 2H), 7.00 – 6.96 (m, 1H), 6.93 (d, *J* = 2.2 Hz, 1H), 6.77 (dd, *J* = 8.2, 2.5 Hz, 1H), 3.86 (s, 3H), 2.62 – 2.52 (m, 2H), 1.70 – 1.64 (m, 2H), 1.56 (q, *J* = 7.8 Hz, 2H), 1.32 (s, 6H), 1.23 – 1.14 (m, 2H).
^{13}C NMR (126 MHz, CDCl$_3$) δ 159.52, 151.68, 143.00, 129.02, 128.47, 128.34, 125.67, 118.59, 112.79, 109.91, 55.28, 44.50, 37.91, 35.98, 32.39, 29.08, 24.65.
HRMS (ESI-TOF) m/z: calc'd for $C_{20}H_{26}NaO$ [M+Na]$^+$: 305.1876; found 305.1882.

Compound 16

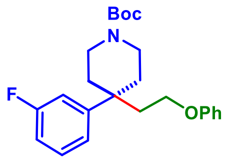

Following **General Procedure A**, electrolysis was conducted for 10 h. Purification by PTLC (silica, 10:1, PE: EA) afforded 46.3 mg (58%) of the title **Compound 16** as a colorless oil.
^{1}H NMR (500 MHz, CDCl$_3$) δ 7.32 (dt, *J* = 9.8, 7.0 Hz, 1H), 7.22 (t, *J* = 7.9 Hz, 2H), 7.10 (d, *J* = 7.9 Hz, 1H), 7.02 (dd, *J* = 11.3, 2.4 Hz, 1H), 6.92 (dt, *J* = 14.9, 7.6 Hz, 2H), 6.71 (d, *J* = 8.1 Hz, 2H), 3.72 – 3.62 (m, 4H), 3.20 (ddd, *J* = 13.3, 9.5, 3.1 Hz, 2H), 2.16 (d, *J* = 14.2 Hz, 2H), 2.09 (t, *J* = 6.8, 2H), 1.85 (t, *J* = 10.4 Hz, 2H), 1.45 (s, 9H).

^{13}C NMR (101 MHz, CDCl$_3$) δ 163.40 (d, J = 244.8 Hz), 158.62, 155.06, 147.48, 130.25 (d, J = 8.5 Hz), 129.55, 122.47 (d, J = 2.4 Hz), 120.82, 114.42, 114.00 (d, J = 21.9 Hz), 113.38 (d, J = 21.1 Hz), 79.62, 64.04, 41.33, 40.88, 39.50, 35.71, 28.59.
^{19}F NMR (376 MHz, CDCl$_3$) δ -112.41.
HRMS (ESI-TOF) m/z: calc'd for $C_{24}H_{31}FNO_3$ [M+H]$^+$: 400.2283; found 400.2287.

Compound 17

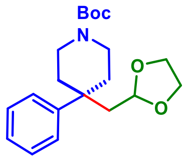

Following **General Procedure A**, electrolysis was conducted for 10 h. Purification by PTLC (silica, 5:1, PE: EA) afforded 36.1 mg (52%) of the title **Compound 17** as a white solid.
^{1}H NMR (400 MHz, CDCl$_3$) δ 7.33 (d, J = 5.8 Hz, 4H), 7.21 (tt, J = 5.7, 2.5 Hz, 1H), 4.32 (t, J = 4.8 Hz, 1H), 3.90 – 3.82 (m, 2H), 3.70 – 3.57 (m, 4H), 3.22 (ddd, J = 13.1, 9.1, 3.2 Hz, 2H), 2.20 - 2.14 (m, 2H), 1.96 (d, J = 4.8 Hz, 2H), 1.85 (ddd, J = 13.1, 9.1, 3.2 Hz, 2H), 1.43 (s, 9H).
^{13}C NMR (126 MHz, CDCl$_3$) δ 155.12, 144.49, 128.69, 126.81, 126.29, 102.66, 79.35, 64.59, 45.98, 40.66, 39.82, 38.35, 35.71, 28.58.
HRMS (ESI-TOF) m/z: calc'd for $C_{20}H_{30}NO_4$ [M+H]$^+$: 348.2170; found 348.2178.

Compound 18

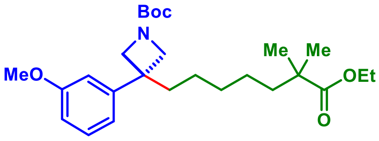

Following **General Procedure A**, electrolysis was conducted for 10 h. Purification by PTLC (silica, 5:1, PE: EA) afforded 46.7 mg (51%) of the title **Compound 18** as a colorless oil.
^{1}H NMR (400 MHz, CDCl$_3$) δ 7.24 (t, J = 6.8 Hz, 1H), 6.75 (dd, J = 8.2, 2.5 Hz, 1H), 6.65 (dd, J = 7.7, 1.6 Hz, 1H), 6.59 (d, J = 2.2 Hz, 1H), 4.14 (d, J = 8.1 Hz, 2H), 4.07 (q, J = 7.1 Hz, 2H), 3.91 (d, J = 8.0 Hz, 2H), 3.80 (s, 3H), 1.84 (dd, J = 10.4, 5.8 Hz, 2H), 1.45 – 1.39 (m, 11H), 1.21 (t, J = 7.1 Hz, 3H), 1.17 – 1.02 (m, 12H).
^{13}C NMR (126 MHz, CDCl$_3$) δ 178.13, 159.70, 156.66, 147.14, 129.47, 118.56, 112.44, 111.20, 79.52, 60.27, 59.09, 55.34, 42.29, 42.20, 42.13, 40.76, 30.41, 28.52, 25.23, 24.93, 24.49, 14.36.
HRMS (ESI-TOF) m/z: calc'd for $C_{26}H_{41}NNaO_5$ [M+Na]$^+$: 470.2877; found 470.2871.

Compound 19

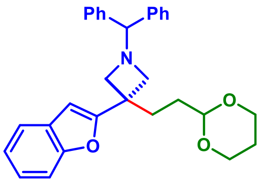

Following **General Procedure A**, electrolysis was conducted for 10 h. Purification by PTLC (silica, 5:1, PE: EA) afforded 41.7 mg (46%) of the title **Compound 19** as a colorless oil.

^{1}H NMR (500 MHz, CDCl$_3$) δ 7.49 (dd, *J* = 7.3, 1.7 Hz, 1H), 7.43 – 7.35 (m, 5H), 7.24 (q, *J* = 7.7 Hz, 4H), 7.18 (dtd, *J* = 12.1, 7.5, 3.3 Hz, 4H), 6.47 (s, 1H), 4.49 (t, *J* = 5.1 Hz, 1H), 4.44 (s, 1H), 4.12 – 4.05 (m, 2H), 3.72 (td, *J* = 12.3, 2.5 Hz, 2H), 3.42 (d, *J* = 7.4 Hz, 2H), 3.28 (d, *J* = 7.4 Hz, 2H), 2.22 – 2.14 (m, 2H), 2.05 (qt, *J* = 12.7, 5.0 Hz, 1H), 1.57 (dt, *J* = 13.1, 4.9 Hz, 2H), 1.35 – 1.23 (m, 1H).
^{13}C NMR (126 MHz, CDCl$_3$) δ 162.00, 154.85, 142.42, 128.85, 128.55, 127.63, 127.20, 123.52, 122.63, 120.63, 111.10, 102.32, 102.13, 77.86, 77.41, 67.04, 62.27, 37.79, 32.04, 30.91, 25.92.
HRMS (ESI-TOF) m/z: calc'd for $C_{30}H_{32}NO_3$ $[M+H]^+$: 454.2377; found 454.2378.

Compound 20

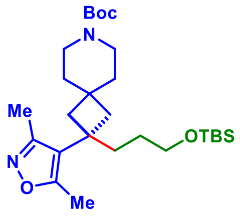

Following **General Procedure A**, electrolysis was conducted for 10 h. Purification by PTLC (silica, 5:1, PE: EA) afforded 29.6 mg (30%) of the title **Compound 20** as a colorless oil.
^{1}H NMR (500 MHz, CDCl$_3$) δ 3.52 (t, *J* = 6.1 Hz, 2H), 3.33 (t, *J* = 5.3 Hz, 2H), 3.27 (t, *J* = 5.7 Hz, 2H), 2.30 (s, 3H), 2.17 (d, *J* = 7.6 Hz, 5H), 2.07 (d, *J* = 12.0 Hz, 2H), 1.78 – 1.72 (m, 2H), 1.63 (d, *J* = 6.5 Hz, 2H), 1.60 (s, 2H), 1.44 (s, 9H), 1.32 – 1.20 (m, 2H), 0.86 (s, 9H), 0.01 (s, 6H).
^{13}C NMR (126 MHz, CDCl$_3$) δ 163.88, 159.08, 155.11, 79.53, 62.95, 42.81, 40.49, 39.77, 38.49, 32.44, 32.15, 28.59, 27.90, 26.02, 18.37, 12.83, 11.90, -5.16.
HRMS (ESI-TOF) m/z: calc'd for $C_{27}H_{49}N_2O_4Si$ $[M+H]^+$: 493.3457; found 493.3457.

Compound 21

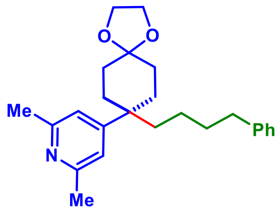

Following **General Procedure A**, electrolysis was conducted for 10 h. Purification by PTLC (silica, 1:1, PE: EA) afforded 35.7 mg (47%) of the title **Compound 21** as a colorless oil.
^{1}H NMR (400 MHz, CDCl$_3$) δ 7.23 (t, *J* = 7.6 Hz, 2H), 7.14 (t, *J* = 7.2 Hz, 1H), 7.08 – 7.04 (m, 2H), 6.88 (s, 2H), 3.97 – 3.85 (m, 4H), 2.54 – 2.43 (m, 8H), 2.14 (d, *J* = 13.4 Hz, 2H), 1.84 – 1.56 (m, 6H), 1.56 – 1.36 (m, 4H), 0.99 (tq, *J* = 12.1, 7.6, 5.9 Hz, 2H).
^{13}C NMR (101 MHz, CDCl$_3$) δ 157.56, 142.64, 128.40, 128.35, 125.74, 118.94, 108.99, 91.28, 64.38, 64.33, 40.73, 35.70, 32.74, 32.03, 31.49, 24.78, 23.43.
HRMS (ESI-TOF) m/z: calc'd for $C_{25}H_{34}NO_2$ $[M+Na]^+$: 380.2585; found 380.2582.

Compound 22

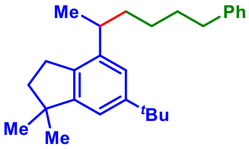

Following **General Procedure A**, electrolysis was conducted for 10 h. Purification by PTLC (silica, PE) afforded 42.1 mg (58%) of the title **Compound 22** as a colorless oil.

^{1}H NMR (500 MHz, CDCl$_3$) δ 7.29 – 7.24 (m, 2H), 7.20 – 7.12 (m, 3H), 7.06 (d, *J* = 1.8 Hz, 1H), 7.02 (d, *J* = 1.8 Hz, 1H), 2.83 – 2.77 (m, 3H), 2.59 (td, *J* = 7.3, 1.7 Hz, 2H), 1.91 (t, *J* = 7.2 Hz, 2H), 1.73 – 1.58 (m, 4H), 1.46 – 1.36 (m, 2H), 1.33 (s, 9H), 1.27 (d, *J* = 3.7 Hz, 6H), 1.23 (d, *J* = 7.0 Hz, 3H).

^{13}C NMR (126 MHz, CDCl$_3$) δ 152.14, 149.83, 143.00, 142.94, 137.85, 128.52, 128.36, 125.69, 120.43, 116.29, 44.19, 41.48, 37.57, 36.62, 36.01, 34.93, 31.86, 31.78, 28.99, 28.92, 28.12, 27.62, 21.09.

HRMS (ESI-TOF) m/z: calc'd for $C_{27}H_{38}Na$ [M+Na]$^+$: 385.2866; found 385.2867.

Compound 23

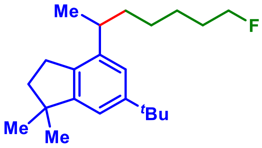

Following **General Procedure A**, electrolysis was conducted for 10 h. Purification by PTLC (silica, PE) afforded 39.5 mg (62%) of the title **Compound 23** as a colorless oil.

^{1}H NMR (500 MHz, CDCl$_3$) δ 7.06 (d, *J* = 1.9 Hz, 1H), 7.02 (d, *J* = 1.8 Hz, 1H), 4.48 (t, *J* = 6.2 Hz, 1H), 4.38 (t, *J* = 6.2 Hz, 1H), 2.89 – 2.74 (m, 3H), 1.93 (t, *J* = 7.2 Hz, 2H), 1.75 – 1.62 (m, 3H), 1.57 – 1.51 (m, 1H), 1.40 (qd, *J* = 6.4, 5.5, 3.3 Hz, 4H), 1.34 (s, 9H), 1.27 (d, *J* = 3.7 Hz, 6H), 1.25 (d, *J* = 6.9 Hz, 3H).

^{13}C NMR (126 MHz, CDCl$_3$) δ 152.17, 149.85, 142.81, 137.84, 120.37, 116.32, 84.34 (d, *J* = 163.8 Hz), 44.20, 41.47, 37.55, 36.61, 34.93, 31.85, 30.46 (d, *J* = 19.3 Hz), 28.97, 28.92, 28.10, 27.55, 25.39 (d, *J* = 5.5 Hz), 21.15.

^{19}F NMR (471 MHz, CDCl$_3$) δ -217.99.

HRMS (ESI-TOF) m/z: calc'd for $C_{22}H_{35}FNa$ [M+Na]$^+$: 341.2615; found 341.2618.

Compound 24

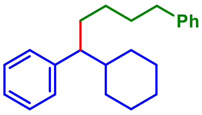

Following **General Procedure A**, electrolysis was conducted for 10 h. Purification by PTLC (silica, PE) afforded 34.1 mg (56%) of the title **Compound 24** as a colorless oil.

^{1}H NMR (400 MHz, CDCl$_3$) δ 7.29 – 7.22 (m, 4H), 7.20 – 7.02 (m, 6H), 2.50 (qdd, *J* = 13.8, 9.3, 6.4 Hz, 2H), 2.27 (ddd, *J* = 11.3, 7.5, 4.1 Hz, 1H), 1.89 (dt, *J* = 12.8, 3.1 Hz, 1H), 1.84 – 1.67 (m, 2H), 1.64 – 1.51 (m, 5H), 1.41 (tt, *J* = 10.7, 3.3 Hz, 2H), 1.29 – 1.19 (m, 1H), 1.17 – 1.03 (m, 4H), 0.97 – 0.85 (m, 1H), 0.76 (qd, *J* = 12.4, 3.2 Hz, 1H).

^{13}C NMR (101 MHz, CDCl$_3$) δ 144.91, 143.03, 128.69, 128.46, 128.32, 128.02, 125.77, 125.64,

52.24, 43.38, 35.96, 32.48, 31.81, 31.57, 31.22, 27.68, 26.76, 26.75, 26.70.

HRMS (ESI-TOF) m/z: calc'd for $C_{23}H_{30}Na$ $[M+Na]^+$: 329.2240; found 329.2249.

Compound 25

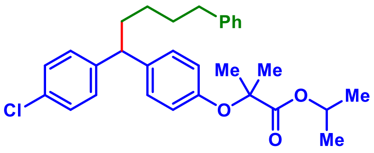

Following **General Procedure A**, electrolysis was conducted for 10 h. Purification by PTLC (silica, 20:1, PE: EA) afforded 73.8 mg (77%) of the title **Compound 25** as a colorless oil.

^{1}H NMR (500 MHz, $CDCl_3$) δ 7.26 – 7.07 (m, 9H), 7.07 – 6.96 (m, 2H), 6.80 – 6.73 (m, 2H), 5.07 (hept, *J* = 6.2 Hz, 1H), 3.80 (dt, *J* = 15.7, 7.8 Hz, 1H), 2.55 (t, *J* = 7.8 Hz, 2H), 2.05 – 1.91 (m, 2H), 1.69 – 1.59 (m, 2H), 1.56 (s, 6H), 1.35 – 1.23 (m, 2H), 1.20 (d, *J* = 6.3 Hz, 6H).

^{13}C NMR (126 MHz, $CDCl_3$) δ 173.90, 154.02, 144.09, 142.68, 138.20, 131.75, 129.30, 128.57, 128.47, 128.39, 127.96, 125.78, 119.10, 79.18, 68.96, 49.87, 35.87, 35.75, 31.50, 27.67, 25.55, 21.68.

HRMS (ESI-TOF) m/z: calc'd for $C_{30}H_{35}ClNaO_3$ $[M+Na]^+$: 501.2167; found 501.2170.

Compound 26

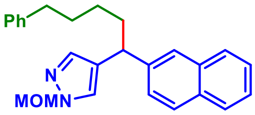

Following **General Procedure A**, electrolysis was conducted for 10 h. Purification by PTLC (silica, 5:1, PE: EA) afforded 29.2 mg (38%) of the title **Compound 26** as a colorless oil.

^{1}H NMR (400 MHz, $CDCl_3$) δ 7.85 – 7.76 (m, 3H), 7.67 – 7.61 (m, 1H), 7.52 – 7.39 (m, 3H), 7.33 (dd, *J* = 8.5, 1.8 Hz, 1H), 7.30 (s, 1H), 7.23 (dd, *J* = 7.3, 1.0 Hz, 2H), 7.19 – 7.10 (m, 3H), 5.31 (s, 2H), 3.98 (dd, *J* = 8.6, 6.8 Hz, 1H), 3.30 (s, 3H), 2.60 – 2.49 (m, 2H), 2.13 – 2.04 (m, 2H), 1.73 – 1.63 (m, 2H), 1.43 – 1.29 (m, 2H).

^{13}C NMR (101 MHz, $CDCl_3$) δ 142.69, 142.54, 139.47, 133.64, 132.40, 128.47, 128.38, 128.34, 127.78, 127.75, 126.35, 126.22, 126.15, 125.77, 125.57, 82.05, 56.77, 42.05, 36.51, 35.88, 31.49, 27.57.

HRMS (ESI-TOF) m/z: calc'd for $C_{26}H_{29}N_2O$ $[M+H]^+$: 385.2275; found 385.2275.

Compound 27

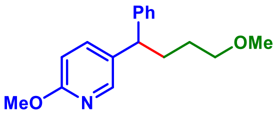

Following **General Procedure A**, electrolysis was conducted for 10 h. Purification by PTLC (silica, 5:1, PE: EA) afforded 10.9 mg (20%) of the title **Compound 27** as a colorless oil.

^{1}H NMR (400 MHz, $CDCl_3$) δ 8.06 (d, *J* = 2.6 Hz, 1H), 7.41 (dd, *J* = 8.7, 2.5 Hz, 1H), 7.30 – 7.26 (m, 2H), 7.23 – 7.15 (m, 3H), 6.66 (d, *J* = 8.6 Hz, 1H), 3.90 (s, 3H), 3.84 (d, *J* = 7.7 Hz, 1H), 3.37 (t, *J* = 6.4 Hz, 2H), 3.30 (s, 3H), 2.08 (dq, *J* = 12.9, 7.9 Hz, 2H), 1.52 (q, *J* = 7.6 Hz, 2H).

^{13}C NMR (126 MHz, $CDCl_3$) δ 162.92, 145.84, 144.50, 138.32, 133.19, 128.71, 127.83, 126.49, 110.87, 72.63, 58.71, 53.47, 48.00, 32.18, 28.22.

HRMS (ESI-TOF) m/z: calc'd for $C_{17}H_{22}NO_2$ $[M+H]^+$: 272.1646; found 272.1648.

Compound 28

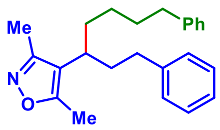

Following **General Procedure A**, electrolysis was conducted for 10 h. Purification by PTLC (silica, 5:1, PE: EA) afforded 29.9 mg (43%) of the title **Compound 28** as a colorless oil.

^{1}H NMR (500 MHz, $CDCl_3$) δ 7.30 – 7.23 (m, 4H), 7.20 – 7.15 (m, 2H), 7.12 (dd, *J* = 8.0, 1.4 Hz, 2H), 7.09 – 7.06 (m, 2H), 2.61 – 2.49 (m, 3H), 2.41 (ddt, *J* = 18.2, 9.9, 6.0 Hz, 2H), 2.22 (s, 3H), 2.19 (s, 3H), 2.00 – 1.80 (m, 2H), 1.60 – 1.49 (m, 4H), 1.20 (p, *J* = 7.8 Hz, 2H).

^{13}C NMR (126 MHz, $CDCl_3$) δ 165.16, 159.50, 142.57, 141.79, 128.54, 128.45, 128.43, 128.41, 126.05, 125.85, 115.48, 36.07, 35.95, 34.80, 34.07, 33.95, 31.57, 27.46, 11.55, 11.44.

HRMS (ESI-TOF) m/z: calc'd for $C_{24}H_{29}NNaO$ $[M+Na]^+$: 370.2142; found 370.2137.

Compound 29

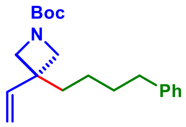

Following **General Procedure A**, electrolysis was conducted for 10 h. Purification by PTLC (silica, 20:1, PE: EA) afforded 32.8 mg (52%) of the title **Compound 29** as a colorless oil.

^{1}H NMR (400 MHz, $CDCl_3$) δ 7.31 – 7.26 (m, 2H), 7.17 (ddd, *J* = 8.7, 7.2, 1.9 Hz, 3H), 5.86 (dd, *J* = 17.4, 10.7 Hz, 1H), 5.16 (d, *J* = 10.7 Hz, 1H), 5.07 (d, *J* = 17.4 Hz, 1H), 3.81 (d, *J* = 8.2 Hz, 2H), 3.64 (d, *J* = 8.2 Hz, 2H), 2.72 – 2.47 (m, 2H), 1.73 – 1.67 (m, 2H), 1.60 (dd, *J* = 8.7, 6.8 Hz, 2H), 1.44 (s, 9H), 1.32 – 1.22 (m, 2H).

^{13}C NMR (101 MHz, $CDCl_3$) δ 156.76, 142.55, 141.59, 128.47, 128.44, 125.85, 114.00, 79.48, 58.00, 39.83, 38.77, 35.98, 31.85, 28.54, 24.29.

HRMS (ESI-TOF) m/z: calc'd for $C_{20}H_{29}NNaO_2$ $[M+Na]^+$: 338.2091; found 338.2095.

Compound 30

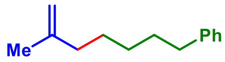

Following **General Procedure A**, electrolysis was conducted for 10 h. Purification by PTLC (silica, PE) afforded 17.7 mg (47%) of the title **Compound 30** as a colorless oil.

^{1}H NMR (500 MHz, $CDCl_3$) δ 7.28 (dd, *J* = 8.2, 7.0 Hz, 2H), 7.21 – 7.14 (m, 3H), 4.83 – 4.54 (m, 2H), 2.66 – 2.51 (m, 2H), 2.05 – 1.95 (m, 2H), 1.71 (s, 3H), 1.67 – 1.60 (m, 2H), 1.50 – 1.43 (m, 2H), 1.40 – 1.30 (m, 2H).

^{13}C NMR (126 MHz, $CDCl_3$) δ 146.34, 142.98, 128.53, 128.37, 125.72, 109.76, 37.88, 36.08, 31.55, 29.14, 27.64, 22.54.

HRMS (ESI-TOF) m/z: calc'd for $C_{14}H_{20}Na$ $[M+Na]^+$: 211.1458; found 211.1453.

Compound 31

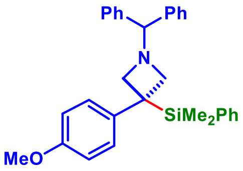

Following **General Procedure A**, electrolysis was conducted for 10 h. Purification by PTLC (silica, 20:1, PE: EA) afforded 62.1 mg (67%) of the title **Compound 31** as a colorless oil.

^{1}H NMR (400 MHz, CDCl$_3$) δ 7.42 – 7.38 (m, 3H), 7.36 – 7.31 (m, 2H), 7.23 (q, *J* = 3.5 Hz, 8H), 7.15 (tq, *J* = 5.1, 2.8 Hz, 2H), 6.70 (d, *J* = 8.6 Hz, 2H), 6.49 (d, *J* = 8.6 Hz, 2H), 4.21 (s, 1H), 3.76 (s, 3H), 3.70 (d, *J* = 6.5 Hz, 2H), 3.19 (d, *J* = 6.5 Hz, 2H), 0.38 (s, 6H).

^{13}C NMR (126 MHz, CDCl$_3$) δ 156.75, 142.80, 139.58, 136.91, 134.70, 129.30, 128.48, 127.46, 127.43, 127.08, 127.01, 113.30, 77.81, 62.44, 55.37, 31.72, -5.73.

HRMS (ESI-TOF) m/z: calc'd for $C_{31}H_{34}NOSi$ [M+H]$^+$: 464.2405; found 464.2400.

Compound 32

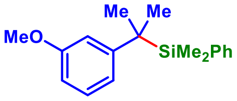

Following **General Procedure A**, electrolysis was conducted for 10 h. Purification by PTLC (silica, 50:1, PE: EA) afforded 33.6 mg (59%) of the title **Compound 32** as a colorless oil.

^{1}H NMR (500 MHz, CDCl$_3$) δ 7.37 – 7.33 (m, 1H), 7.29 (d, *J* = 4.1 Hz, 4H), 7.13 (t, *J* = 8.0 Hz, 1H), 6.65 (dt, *J* = 8.0, 2.1 Hz, 2H), 6.54 (t, *J* = 2.2 Hz, 1H), 3.67 (s, 3H), 1.34 (s, 6H), 0.22 (s, 6H).

^{13}C NMR (126 MHz, CDCl$_3$) δ 159.15, 150.21, 137.05, 134.89, 129.10, 128.49, 127.41, 119.16, 112.74, 109.72, 55.10, 27.81, 24.40, -5.60.

HRMS (ESI-TOF) m/z: calc'd for $C_{18}H_{25}OSi$ [M+H]$^+$: 285.1670; found 285.1677.

Compound 33

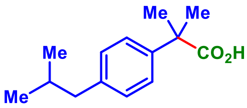

Following **General Procedure B**, electrolysis was conducted for 10 h. Purification by reverse phase column chromatography (C18, 1:1, MeCN: H_2O) afforded 25.1 mg (57%) of the title **Compound 33** as a colorless oil.

^{1}H NMR (500 MHz, CDCl$_3$) δ 7.31 (d, *J* = 8.3 Hz, 2H), 7.11 (d, *J* = 8.2 Hz, 2H), 2.45 (d, *J* = 7.2 Hz, 2H), 1.85 (dp, *J* = 13.5, 6.7 Hz, 1H), 1.59 (s, 6H), 0.91 (d, *J* = 6.6 Hz, 6H).

^{13}C NMR (101 MHz, CDCl$_3$) δ 183.28, 141.18, 140.49, 129.27, 125.67, 46.04, 45.07, 30.28, 26.37, 22.56.

HRMS (ESI-TOF) m/z: calc'd for $C_{14}H_{20}NaO_2$ [M+Na]$^+$: 243.1356; found 243.1352.

Compound 34

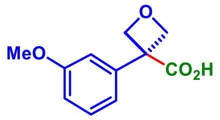

Following **General Procedure B**, electrolysis was conducted for 10 h. Purification by reverse phase column chromatography (C18, 3:2, MeCN: H_2O) afforded 31.2 mg (75%) of the title **Compound 34** as a white solid.

^{1}H NMR (500 MHz, $CDCl_3$) δ 9.62 (s, 1H), 7.30 (t, J = 7.9 Hz, 1H), 6.85 (ddd, J = 14.4, 8.1, 2.2 Hz, 2H), 6.79 (t, J = 2.2 Hz, 1H), 5.25 (d, J = 6.2 Hz, 2H), 5.04 (d, J = 6.2 Hz, 2H), 3.81 (s, 3H).

^{13}C NMR (101 MHz, $CDCl_3$) δ 178.58, 159.97, 140.39, 130.01, 118.55, 113.16, 112.41, 79.55, 55.42, 53.17.

HRMS (ESI-TOF) m/z: calc'd for $C_{11}H_{12}NaO_4$ $[M+Na]^+$: 231.0628; found 231.0630.

Compound 35

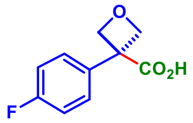

Following **General Procedure B**, electrolysis was conducted for 10 h. Purification by reverse phase column chromatography (C18, 2:3, MeCN: H_2O) afforded 20.4 mg (52%) of the title **Compound 35** as a white solid.

^{1}H NMR (500 MHz, $CDCl_3$) δ 7.26 – 7.19 (m, 2H), 7.11 – 7.04 (m, 2H), 5.26 (d, J = 6.3 Hz, 2H), 5.01 (d, J = 6.3 Hz, 2H).

^{13}C NMR (126 MHz, $CDCl_3$) δ 178.57, 162.43 (d, J = 247.5 Hz), 134.68 (d, J = 3.2 Hz), 128.14 (d, J = 8.2 Hz), 116.02 (d, J = 21.7 Hz), 79.49, 52.64.

^{19}F NMR (471 MHz, $CDCl_3$) δ -113.76.

HRMS (ESI-TOF) m/z: calc'd for $C_{10}H_9FNaO_3$ $[M+Na]^+$: 219.0428; found 219.0431.

Benzoate Group Enables Multistep Transformations

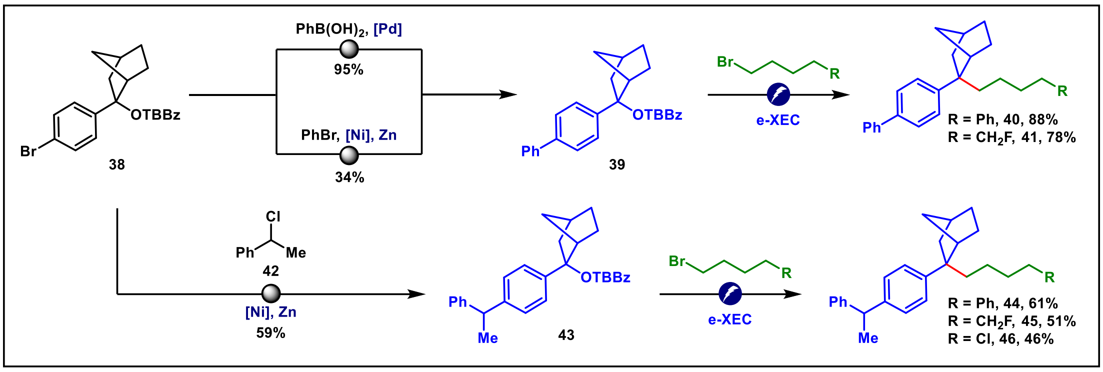

Compound 38

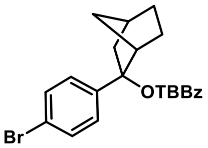

Prepared following the **General procedure 1** using 1,4-dibromobenzene (1.1 equiv., 5.5 mmol), *n*-BuLi (2.5 M in hexanes, 1.1 equiv., 5.5 mmol), bicyclo[2.2.1]heptan-2-one (1.0 equiv., 5 mmol), TBBzCl (1.2 equiv., 6 mmol) and purified by silica gel column chromatography to afford the desired **Compound 38** (1.45 g, 68%) as a white solid.

^{1}H NMR (500 MHz, CDCl$_3$) δ 7.95 (d, *J* = 8.5 Hz, 2H), 7.46 (d, *J* = 8.5 Hz, 2H), 7.42 (d, *J* = 8.7 Hz, 2H), 7.37 (d, *J* = 8.7 Hz, 2H), 3.25 – 3.09 (m, 1H), 2.47 – 2.33 (m, 2H), 2.03 – 1.87 (m, 2H), 1.66 (ddt, *J* = 11.6, 7.3, 3.6 Hz, 1H), 1.61 – 1.52 (m, 2H), 1.48 – 1.38 (m, 2H), 1.34 (s, 9H).

^{13}C NMR (126 MHz, CDCl$_3$) δ 165.64, 156.69, 145.02, 131.41, 129.51, 128.18, 127.64, 125.49, 120.85, 88.37, 46.45, 45.55, 37.71, 36.79, 35.20, 31.24, 28.96, 23.18.

HRMS (ESI-TOF) m/z: calc'd for $C_{24}H_{28}BrO_2$ [M+Na]$^+$: 427.1268; found 427.1267.

Benzoate **38** underwent both a redox-neutral palladium-catalyzed Suzuki coupling (*62*) and a nickel-catalyzed reductive C(sp^2)–C(sp^2) coupling (*63*) to furnish product **39**, with the benzoate moiety remaining intact. Likewise, benzyl benzoate **38** participated in a nickel-catalyzed reductive C(sp^2)–C(sp^3) coupling with benzyl chloride **42** (*64*), again leaving the benzoate group unaltered to afford compound **43.**

Palladium-catalyzed Suzuki coupling

In a 10 mL Schlenk flask equipped with a magnetic stir bar, **Compound 38** (1.0 equiv., 1.0 mmol), K_2CO_3 (3.0 equiv., 3.0 mmol), $PhB(OH)_2$ (2.0 equiv., 2.0 mmol) and $PdCl_2$dppf (3 mol%, 0.03 mmol) were sequentially added. The flask was evacuated and purged with nitrogen gas three times, followed by the addition of dioxane (2 mL) and H_2O (0.3 mL). The reaction mixture was stirred at 80 °C overnight. After the reaction, saturated NH_4Cl(aq.) was added, and the mixture was extracted with EtOAc. The combined organic phase was dried over anhydrous sodium sulfate, filtered, and concentrated in vacuo. The crude product was purified by silica gel column chromatography to afford the desired **Compound 39** (0.43 g, 95%) as a white solid.

Nickel-catalyzed reductive C(sp²)–C(sp²) coupling

In a 10 mL Schlenk flask equipped with a magnetic stir bar, **Compound 38** (1.0 equiv., 1.0 mmol), Zn (2.0 equiv., 2.0 mmol), TBAI (1.0 equiv., 1.0 mmol), $MgCl_2$ (1.0 equiv., 1 mmol), NiI_2 (10 mol%, 0.1 mmol) and dtbbpy (10 mol%, 0.1 mmol) were sequentially added. The flask was evacuated and purged with nitrogen gas three times, followed by the addition of DMAc (6 mL), PhBr (2.0 equiv. 2.0 mmol) and pyridine (1.0 equiv. 1.0 mmol). The reaction mixture was stirred at room tempeture overnight. After the reaction, saturated NH_4Cl(aq) was added, the mixture was extracted with EtOAc and washed by saturated NaCl(aq). The combined organic phase was dried over anhydrous sodium sulfate, filtered, and concentrated in vacuo. The crude product was purified by silica gel column chromatography to afford the desired **Compound 39** (0.14 g, 34%) as a white solid.
(Note: The low yield of the nickel-catalyzed reductive C(sp²)–C(sp²) coupling was not due to the cleavage of the OTBBz group. ***Compound 38*** *was recovered in 21% yield, and its dimer was also detected in 14% yield. Literature reports have also emphasized that a substantial difference in the electronic properties of the coupling partners is crucial for achieving high coupling efficiency (63).)*

Compound 39

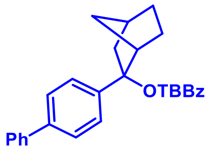

^{1}H NMR (400 MHz, $CDCl_3$) δ 7.98 (d, J = 8.5 Hz, 2H), 7.59 – 7.48 (m, 6H), 7.46 (d, J = 8.5 Hz, 2H), 7.41 (t, J = 7.6 Hz, 2H), 7.36 – 7.27 (m, 1H), 3.27 (d, J = 3.8 Hz, 1H), 2.61 – 2.49 (m, 1H), 2.41 (d, J = 4.6 Hz, 1H), 2.03 – 1.91 (m, 2H), 1.66 (dd, J = 16.4, 7.0 Hz, 3H), 1.51 – 1.38 (m, 2H), 1.34 (s, 9H).
^{13}C NMR (101 MHz, $CDCl_3$) δ 165.74, 156.54, 144.94, 140.98, 139.65, 129.56, 128.79, 128.50, 127.24, 127.21, 127.06, 126.25, 125.47, 88.80, 46.65, 45.56, 37.78, 36.83, 35.21, 31.27, 29.12, 23.28.
HRMS (ESI-TOF) m/z: calc'd for $C_{30}H_{32}NaO_2$ $[M+Na]^+$: 447.2295; found 447.2301.

Compound 40

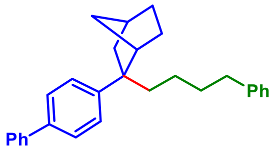

Following **General Procedure A**, electrolysis was conducted for 10 h. Purification by PTLC (silica, 100:1, PE: EA) afforded 67.0 mg (88%) of the title **Compound 40** as a colorless oil.
^{1}H NMR (400 MHz, $CDCl_3$) δ 7.67 – 7.60 (m, 2H), 7.57 – 7.50 (m, 2H), 7.48 – 7.41 (m, 2H), 7.37 – 7.30 (m, 1H), 7.29 – 7.27 (m, 2H), 7.24 – 7.17 (m, 2H), 7.16 – 7.10 (m, 1H), 7.08 – 7.03 (m, 2H), 2.52 – 2.38 (m, 3H), 2.31 (d, J = 4.5 Hz, 1H), 1.84 (ddd, J = 12.2, 6.7, 2.1 Hz, 2H), 1.70 – 1.54 (m, 3H), 1.42 (ddt, J = 13.4, 11.7, 5.8 Hz, 3H), 1.35 – 1.21 (m, 3H), 1.17 – 1.02 (m, 1H), 1.01 – 0.83 (m, 2H).
^{13}C NMR (126 MHz, $CDCl_3$) δ 146.73, 142.99, 141.19, 137.75, 128.83, 128.44, 128.29, 127.04, 126.31, 125.60, 51.26, 46.03, 44.86, 41.12, 37.37, 37.17, 35.92, 32.25, 29.10, 25.64, 23.94.

HRMS (ESI-TOF) m/z: calc'd for $C_{29}H_{33}$ $[M+H]^+$: 381.2577; found 381.2571.

Compound 41

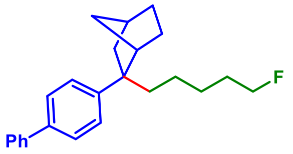

Following **General Procedure A**, electrolysis was conducted for 10 h. Purification by PTLC (silica, 100:1, PE: EA) afforded 52.5 mg (78%) of the title **Compound 41** as a colorless oil.
^{1}H NMR (400 MHz, $CDCl_3$) δ 7.63 (d, *J* = 7.9 Hz, 2H), 7.53 (d, *J* = 8.0 Hz, 2H), 7.44 (t, *J* = 7.6 Hz, 2H), 7.36 – 7.30 (m, 1H), 7.28 (d, *J* = 8.3 Hz, 2H), 4.39 (t, *J* = 6.2 Hz, 1H), 4.27 (t, *J* = 6.2 Hz, 1H), 2.48 (d, *J* = 4.0 Hz, 1H), 2.37 – 2.25 (m, 1H), 1.88 – 1.78 (m, 2H), 1.72 – 1.48 (m, 5H), 1.46 – 1.29 (m, 3H), 1.22 (dq, *J* = 15.1, 7.4, 6.7 Hz, 3H), 1.12 – 0.93 (m, 2H), 0.92 - 0.8 (m, 1H).
^{13}C NMR (126 MHz, $CDCl_3$) δ 146.61, 141.14, 137.78, 128.83, 128.76, 127.05, 127.02, 126.33, 84.27 (d, *J* = 164.0 Hz), 51.20, 46.11, 44.96, 41.05, 37.38, 37.17, 30.43 (d, *J* = 19.5 Hz), 29.05, 25.80, 25.77 (d, *J* = 5.5 Hz), 23.87.
^{19}F NMR (376 MHz, $CDCl_3$) δ -218.01.
HRMS (ESI-TOF) m/z: calc'd for $C_{24}H_{30}F$ $[M+H]^+$: 337.2327; found 337.2324.

Nickel-catalyzed reductive C(sp²)–C(sp³) coupling

In a 10 mL Schlenk flask equipped with a magnetic stir bar, **Compound 38** (1.0 equiv., 1.0 mmol), Zn (2.0 equiv., 2.0 mmol), KF (1.0 equiv., 1.0 mmol), NiI_2 (10 mol%, 0.1 mmol) and dMebpy (10 mol%, 0.1 mmol) were sequentially added. The flask was evacuated and purged with nitrogen gas three times, followed by the addition of DMAc (4 mL) and **Compound 42** (2.0 equiv. 2.0 mmol). The reaction mixture was stirred at room tempeture overnight. After the reaction, saturated NH_4Cl(aq) was added, the mixture was extracted with EtOAc and washed by saturated NaCl(aq). The combined organic phase was dried over anhydrous sodium sulfate, filtered, and concentrated in vacuo. The crude product was purified by silica gel column chromatography to afford the desired **Compound 43** (0.27 g, 59%, dr = 1:1) as a white solid.

Compound 43

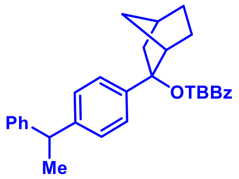

^{1}H NMR (500 MHz, $CDCl_3$) δ 7.96 (d, *J* = 8.4 Hz, 2H), 7.45 (d, *J* = 8.5 Hz, 2H), 7.39 (d, *J* = 8.4 Hz, 2H), 7.30 – 7.24 (m, 2H), 7.23 – 7.19 (m, 2H), 7.19 – 7.15 (m, 1H), 7.16 – 7.09 (m, 2H), 4.09 (q, *J* = 7.2 Hz, 1H), 3.22 (d, *J* = 4.0 Hz, 1H), 2.45 (ddd, *J* = 13.8, 4.8, 2.6 Hz, 1H), 2.35 (t, *J* = 4.6 Hz, 1H), 1.97 – 1.87 (m, 2H), 1.72 – 1.53 (m, 6H), 1.43 – 1.36 (m, 2H), 1.34 (s, 9H).
^{13}C NMR (126 MHz, $CDCl_3$) δ 165.60, 156.39, 146.47, 146.44, 144.60, 144.57, 143.40, 143.39, 129.50, 128.60, 128.43, 127.84, 127.43, 126.10, 125.81, 125.40, 88.85, 46.47, 46.45, 45.51, 44.50, 37.69, 36.76, 35.17, 31.27, 29.13, 23.25, 21.99, 21.97.

HRMS (ESI-TOF) m/z: calc'd for $C_{32}H_{36}NaO_2$ $[M+Na]^+$: 475.2608; found 475.2607.

Compound 44

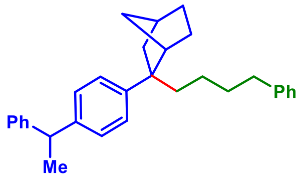

Following **General Procedure A**, electrolysis was conducted for 10 h. Purification by PTLC (silica, 100:1, PE: EA) afforded 49.9 mg (61%, dr = 1:1) of the title **Compound 44** as a colorless oil.

^{1}H NMR (400 MHz, CDCl$_3$) δ 7.32 – 7.27 (m, 2H), 7.27 – 7.15 (m, 5H), 7.16 – 7.01 (m, 7H), 4.13 (q, J = 7.2 Hz, 1H), 2.52 – 2.38 (m, 3H), 2.25 (t, J = 4.4 Hz, 1H), 1.82 – 1.70 (m, 2H), 1.64 (d, J = 7.2 Hz, 3H), 1.55 – 1.33 (m, 5H), 1.31 – 1.22 (m, 3H), 1.16 (td, J = 10.1, 9.4, 2.2 Hz, 1H), 1.03 (tdd, J = 11.9, 7.2, 4.3 Hz, 1H), 0.96 – 0.76 (m, 2H).

^{13}C NMR (101 MHz, CDCl$_3$) δ 147.03, 144.96, 143.05, 142.69, 142.67, 128.44, 128.28, 128.25, 127.85, 126.84, 126.04, 125.59, 51.03, 46.07, 44.78, 44.47, 40.97, 37.35, 37.15, 35.94, 32.23, 29.08, 25.56, 23.96, 22.21, 22.20.

HRMS (ESI-TOF) m/z: calc'd for $C_{31}H_{36}Na$ $[M+Na]^+$: 431.2710; found 431.2705.

Compound 45

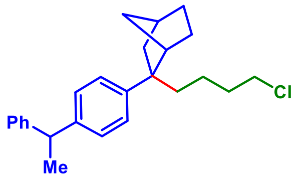

Following **General Procedure A**, electrolysis was conducted for 10 h. Purification by PTLC (silica, 100:1, PE: EA) afforded 33.8 mg (46%, dr = 1:1) of the title **Compound 45** as a colorless oil.

^{1}H NMR (400 MHz, CDCl$_3$) δ 7.32 – 7.27 (m, 2H), 7.24 – 7.16 (m, 3H), 7.15 – 7.07 (m, 4H), 4.12 (q, J = 7.3 Hz, 1H), 3.37 (td, J = 6.9, 2.4 Hz, 2H), 2.40 (d, J = 4.1 Hz, 1H), 2.29 – 2.22 (m, 1H), 1.82 – 1.73 (m, 2H), 1.63 (d, J = 7.3 Hz, 3H), 1.60 – 1.23 (m, 8H), 1.20 – 1.02 (m, 2H), 0.97 – 0.79 (m, 2H).

^{13}C NMR (101 MHz, CDCl$_3$) δ 146.98, 146.97, 144.60, 142.89, 142.86, 128.43, 128.19, 127.82, 126.96, 126.04, 50.97, 46.07, 45.11, 44.44, 44.20, 40.88, 40.87, 37.35, 37.15, 33.28, 29.01, 25.54, 22.16, 22.14, 21.70.

HRMS (ESI-TOF) m/z: calc'd for $C_{25}H_{31}ClNa$ $[M+Na]^+$: 389.2007; found 389.2009.

Applications

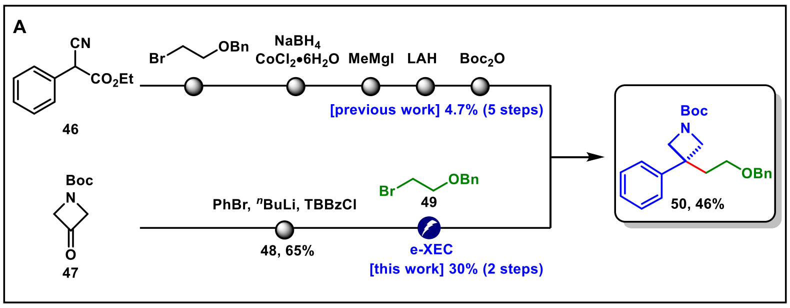

Compound 48 was prepared following the **General procedure 1** using bromobenzene (1.1 equiv., 5.5 mmol), *n*-BuLi (2.5 M in hexanes, 1.1 equiv., 5.5 mmol), **Compound 47** (1.0 equiv., 5 mmol), TBBzCl (1.2 equiv., 6 mmol). The crude product was purified by silica gel column chromatography to afford the desired **Compound 48** (1.33 g, 65%) as a white solid.

Compound 50 was prepared following **General Procedure A**, electrolysis was conducted for 10 h. The crude product was purified by PTLC (silica, 10:1, PE: EA) to afford the desired **Compound 50** **(**33.8 mg, 46%**)** as a colorless oil.

Compound 48

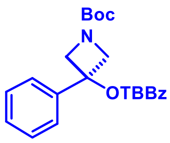

^{1}H NMR (400 MHz, CDCl$_3$) δ 8.02 (dd, J = 8.5, 1.6 Hz, 2H), 7.48 (ddd, J = 8.7, 7.7, 1.5 Hz, 4H), 7.37 (td, J = 7.5, 1.6 Hz, 2H), 7.33 – 7.27 (m, 1H), 4.55 – 4.43 (m, 4H), 1.47 (s, 9H), 1.35 (s, 9H).
^{13}C NMR (101 MHz, CDCl$_3$) δ 165.10, 157.44, 156.43, 140.26, 129.81, 128.76, 128.14, 126.93, 125.65, 124.84, 80.21, 76.39, 61.92, 35.29, 31.21, 28.47.
HRMS (ESI-TOF) m/z: calc'd for $C_{25}H_{31}NNaO_4$ [M+Na]$^+$: 432.2146; found 432.2142.

Compound 50

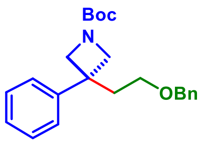

^{1}H NMR (500 MHz, CDCl$_3$) δ 7.35 – 7.30 (m, 4H), 7.29 – 7.26 (m, 3H), 7.25 – 7.20 (m, 1H), 7.12 – 7.06 (m, 2H), 4.37 (s, 2H), 4.22 (d, J = 8.4 Hz, 2H), 4.12 (d, J = 8.3 Hz, 2H), 3.27 (t, J = 6.4 Hz, 2H), 2.24 (t, J = 6.4 Hz, 2H), 1.43 (s, 9H).
^{13}C NMR (126 MHz, CDCl$_3$) δ 156.59, 144.77, 138.40, 128.54, 128.49, 127.67, 127.63, 126.52, 126.20, 79.50, 73.22, 67.29, 41.53, 40.79, 28.53.
HRMS (ESI-TOF) m/z: calc'd for $C_{23}H_{29}NNaO_3$ [M+Na]$^+$: 390.2040; found 390.2041.

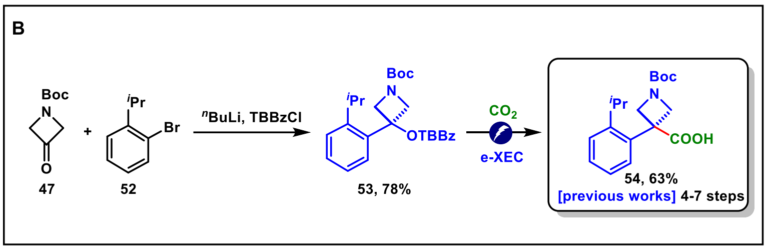

Compound 53 was prepared following the **General procedure 1** using **Compound 52** (1.1 equiv., 5.5 mmol), *n*-BuLi (2.5 M in hexanes, 1.1 equiv., 5.5 mmol), **Compound 47** (1.0 equiv., 5 mmol), TBBzCl (1.2 equiv., 6 mmol). The crude product was purified by silica gel column chromatography to afford the desired **Compound 53** (1.76 g, 78%) as a white solid.
Compound 54 was prepared following **General Procedure B**, electrolysis was conducted for 10 h. The crude product was purified by reverse phase column chromatography (C18, 7:3, MeCN: H_2O) to afford the desired **Compound 54** (40.2 mg, 63%) as a white solid.

Compound 53

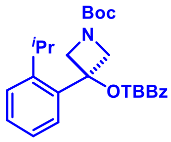

^{1}H NMR (400 MHz, $CDCl_3$) δ 7.92 (d, *J* = 8.5 Hz, 2H), 7.68 (d, *J* = 7.8 Hz, 1H), 7.43 (d, *J* = 8.5 Hz, 2H), 7.35 – 7.29 (m, 2H), 7.23 (td, *J* = 5.6, 3.1 Hz, 1H), 4.81 – 4.46 (m, 4H), 3.06 (p, *J* = 6.8 Hz, 1H), 1.45 (s, 9H), 1.32 (s, 9H), 1.19 (d, *J* = 6.8 Hz, 6H).
^{13}C NMR (101 MHz, $CDCl_3$) δ 164.96, 157.13, 156.14, 148.00, 134.99, 130.63, 129.60, 129.24, 128.68, 127.18, 125.48, 125.16, 80.09, 77.70, 60.55, 35.21, 31.18, 29.91, 28.46, 24.61.
HRMS (ESI-TOF) m/z: calc'd for $C_{28}H_{37}NNaO_4$ $[M+Na]^+$: 474.2615; found 474.2606.

Compound 54

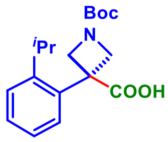

^{1}H NMR (500 MHz, $CDCl_3$) δ 7.31 (d, *J* = 4.0 Hz, 2H), 7.22 – 7.18 (m, 1H), 7.15 (d, *J* = 7.8 Hz, 1H), 4.62 (d, *J* = 8.5 Hz, 2H), 4.35 (d, *J* = 8.4 Hz, 2H), 2.60 (p, *J* = 6.7 Hz, 1H), 1.43 (s, 9H), 1.17 (d, *J* = 6.7 Hz, 6H).
^{13}C NMR (101 MHz, $CDCl_3$) δ 156.22, 147.66, 135.93, 128.58, 127.44, 127.16, 126.14, 80.30, 58.29, 31.22, 29.77, 28.48, 24.37.
HRMS (ESI-TOF) m/z: calc'd for $C_{18}H_{25}NNaO_4$ $[M+Na]^+$: 342.1676; found 342.1673.

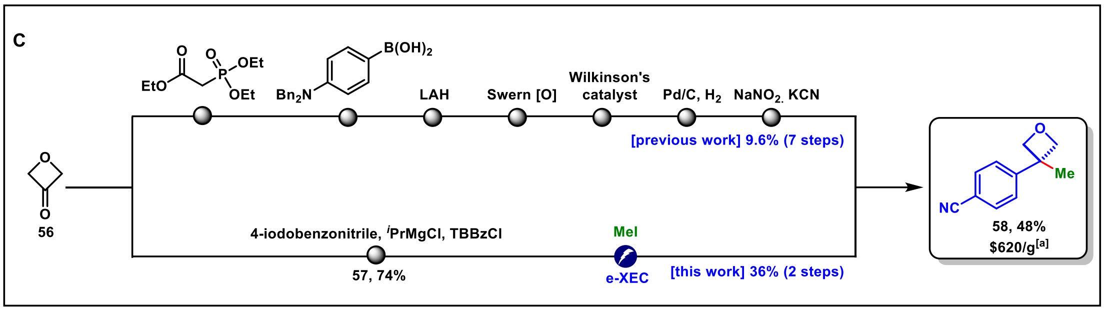

Under nitrogen atmosphere, to a solution of 4-bromobenzonitrile (1.1 equiv., 5.5 mmol) in dry THF (0.3 M) was added dropwise a solution of ^{i}PrMgCl • LiCl (1.3 M in THF, 1.1 equiv.) at –20 °C. After stirring for 1 h, **Compound 56** (1.0 equiv., 5 mmol) in dry THF was added dropwise to the reaction mixture. The reaction mixture was stirred at –20 °C for 5 minutes and allowed to warm to room temperature overnight. Then TBBzCl (1.2 equiv., 6 mmol) was added dropwise at 0 °C. The mixture was allowed to warm to room temperature for another 3 h. After the reaction, Saturated aqueous NH_4Cl solution was added to quench the reaction and the organic layer was separated. The aqueous layer was extracted with EtOAc (×3), and then the combined organic layer was washed with brine, dried over Na_2SO_4, filtrated, and removed under reduced pressure. The crude product was purified by silica gel column chromatography to afford the desired **Compound 57** (1.24 g, 74%) as a white solid.

Compound 58 was prepared following **General Procedure A** with modification: MeI (5.0 equiv.) was employed instead, electrolysis was conducted for 10 h. The crude product was purified by PTLC (silica, 10:1, PE: EA) to afford the desired **Compound 58** (16.6 mg, 48%) as a colorless oil.

Compound 57

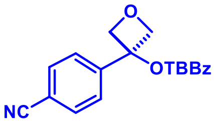

^{1}H NMR (400 MHz, $CDCl_3$) δ 8.04 (dd, *J* = 8.4, 1.5 Hz, 2H), 7.74 – 7.62 (m, 4H), 7.52 (dd, *J* = 8.5, 1.5 Hz, 2H), 5.35 – 5.13 (m, 2H), 5.00 – 4.90 (m, 2H), 1.37 (s, 9H).

^{13}C NMR (101 MHz, $CDCl_3$) δ 164.96, 158.02, 145.02, 132.73, 129.92, 126.06, 125.83, 125.25, 118.51, 112.13, 82.18, 79.54, 35.34, 31.16.

HRMS (ESI-TOF) m/z: calc'd for $C_{21}H_{21}NNaO_3$ $[M+Na]^+$: 358.1414; found 358.1419.

Compound 58

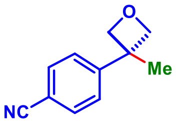

^{1}H NMR (400 MHz, $CDCl_3$) δ 7.70 – 7.59 (m, 2H), 7.36 – 7.28 (m, 2H), 4.93 (d, *J* = 5.7 Hz, 2H), 4.67 (dd, *J* = 5.9, 0.8 Hz, 2H), 1.74 (s, 3H).

^{13}C NMR (101 MHz, $CDCl_3$) δ 151.92, 132.67, 126.12, 118.87, 110.57, 83.26, 43.87, 27.50.

HRMS (ESI-TOF) m/z: calc'd for $C_{11}H_{12}NO$ $[M+H]^+$: 174.0914; found 174.0915.

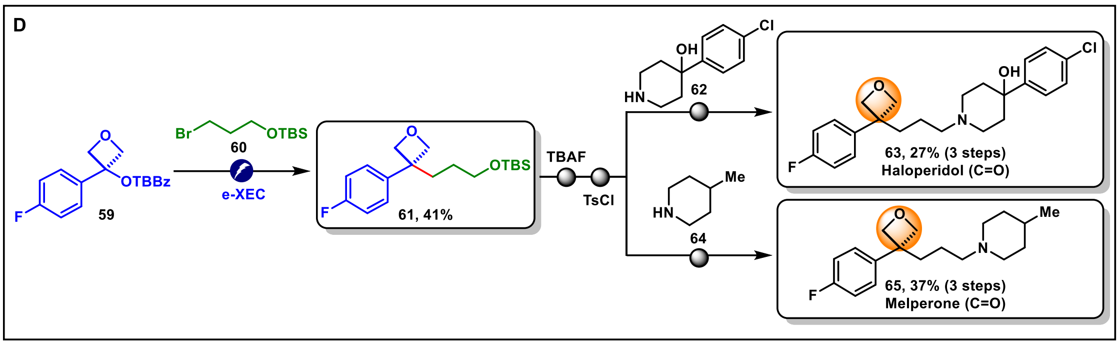

Compound 59 was prepared following the **General procedure 1** using 1-bromo-4-fluorobenzene (1.1 equiv., 5.5 mmol), *n*-BuLi (2.5 M in hexanes, 1.1 equiv., 5.5 mmol), **Compound 56** (1.0 equiv., 5 mmol), TBBzCl (1.2 equiv., 6 mmol). The crude material was purified by silica gel column chromatography to afford the desired **Compound 59** (1.12 g, 71%) as a white solid.

Compound 61 was prepared following **General Procedure A** with modification: **Compound 59** (1.0 equiv., 0.5 mmol) and $TBAClO_4$ (0.2 M) were employed instead, electrolysis was conducted for 10 h. The crude product was purified by silica gel column chromatography (silica, 10:1, PE: EA) afforded 66.5 mg (41%) of **Compound 61** as a colorless oil.

In a 10 mL Schlenk flask equipped with a magnetic stir bar. The flask was evacuated and purged with nitrogen gas three times, followed by the addition of THF (2 mL) and **Compound 61**, then TBAF (1 M in THF, 5 mmol, 5.0 equiv.) was added dropwise at 0 °C. The reaction mixture was removed from ice bath and stirred at room temperature overnight. After the reaction, saturated NH_4Cl(aq) was added, and the mixture was extracted with EtOAc. The combined organic phase was dried over anhydrous sodium sulfate, filtered, and concentrated in vacuo. The crude product was used for the next step without further purification.

In a 10 mL Schlenk flask equipped with a magnetic stir bar, DMAP (15 mol%, 0.03 mmol), and TsCl (1.1 equiv., 0.22 mmol) were added. The flask was evacuated and purged with nitrogen gas three times, followed by the addition of DCM (2 mL). The crude alcohol and TEA (2.0 equiv., 0.4 mmol) were then added. The reaction mixture was stirred at room temperature overnight. After the reaction, deionized water was added, and the mixture was extracted with DCM. The combined organic phase was dried over anhydrous sodium sulfate, filtered, and concentrated in vacuo. The crude product was used for the next step without further purification.

In a 10 mL Schlenk flask equipped with a magnetic stir bar, K_2CO_3 (1.3 equiv., 0.26 mmol) was added. The flask was evacuated and purged with nitrogen gas three times. The crude tosylate was dissolved in acetonitrile, followed by the addition of **Compound 62** (1.0 equiv., 0.2 mmol). The reaction mixture was stirred at 80 °C overnight. After the reaction, the mixture was cooled to room temperature, saturated NaCl(aq) was added, and the mixture was extracted with EtOAc. The combined organic phase was dried over anhydrous sodium sulfate, filtered, and concentrated in vacuo. The crude product was purified by PTLC (silica, 20:1, DCM: MeOH) to afford the desired **Compound 63** (22.4 mg, 27%, 3 steps) as a pale yellow oil.

Compound 59

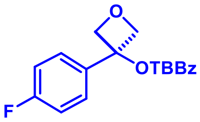

^{1}H NMR (400 MHz, CDCl$_3$) δ 8.03 (d, *J* = 8.0 Hz, 2H), 7.52 (ddt, *J* = 10.9, 8.6, 4.0 Hz, 4H), 7.13 – 7.00 (m, 2H), 5.26 – 5.18 (m, 2H), 5.01 (d, *J* = 7.5 Hz, 2H), 1.36 (s, 9H).
^{13}C NMR (101 MHz, CDCl$_3$) δ 165.05, 162.48 (d, *J* = 247.1 Hz), 157.61, 135.67 (d, *J* = 3.3 Hz), 129.84, 126.70 (d, *J* = 8.0 Hz), 125.71, 115.71 (d, *J* = 21.6 Hz), 82.46, 79.94, 35.30, 31.19.
^{19}F NMR (376 MHz, CDCl$_3$) δ -114.06.
HRMS (ESI-TOF) m/z: calc'd for $C_{20}H_{21}FNaO_3$ $[M+Na]^+$: 351.1367; found 351.1363.

Compound 61

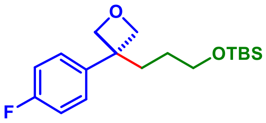

^{1}H NMR (400 MHz, CDCl$_3$) δ 7.05 – 6.97 (m, 4H), 4.92 (d, *J* = 5.6 Hz, 2H), 4.65 (d, *J* = 5.6 Hz, 2H), 3.55 (t, *J* = 6.3 Hz, 2H), 2.14 – 2.06 (m, 2H), 1.37 – 1.28 (m, 2H), 0.87 (s, 9H), 0.00 (s, 6H).
^{13}C NMR (101 MHz, CDCl$_3$) δ 161.42 (d, *J* = 244.9 Hz), 140.86 (d, *J* = 3.2 Hz), 127.45 (d, *J* = 8.0 Hz), 115.38 (d, *J* = 21.3 Hz), 82.05, 63.07, 46.85, 37.52, 27.97, 26.07, 18.45, -5.19.
^{19}F NMR (376 MHz, CDCl$_3$) δ -116.71.
HRMS (ESI-TOF) m/z: calc'd for $C_{18}H_{29}FNaO_2Si$ $[M+Na]^+$: 347.1814; found 347.1818.

Compound 63

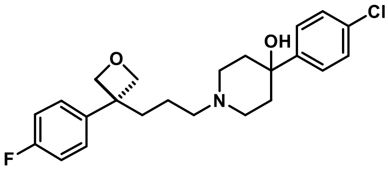

^{1}H NMR (400 MHz, CDCl$_3$) δ 7.42 (d, *J* = 8.2 Hz, 2H), 7.30 (d, *J* = 8.3 Hz, 2H), 7.10 – 6.93 (m, 4H), 4.93 (d, *J* = 5.6 Hz, 2H), 4.65 (d, *J* = 5.6 Hz, 2H), 2.71 (d, *J* = 11.2 Hz, 2H), 2.36 (t, *J* = 7.8 Hz, 4H), 2.08 (dd, *J* = 10.2, 6.7 Hz, 4H), 1.69 (d, *J* = 13.5 Hz, 2H), 1.62 (s, 1H) 1.34 (p, *J* = 8.2, 7.8 Hz, 2H).
^{13}C NMR (101 MHz, CDCl$_3$) δ 161.46 (d, *J* = 245.2 Hz), 146.96, 140.74 (d, *J* = 3.2 Hz), 132.97, 128.58, 127.42 (d, *J* = 8.0 Hz), 126.22, 115.47 (d, *J* = 21.4 Hz), 81.92, 71.19, 58.77, 49.59, 47.05, 39.17, 38.56, 22.28.
^{19}F NMR (376 MHz, CDCl$_3$) δ -116.49.
HRMS (ESI-TOF) m/z: calc'd for $C_{23}H_{28}ClFNO_2$ $[M+H]^+$: 404.1788; found 404.1786.

Compound 65

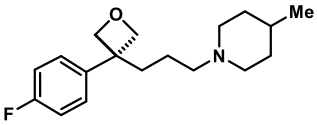

Compound 65 (21.6 mg, 37%, pale yellow oil) was prepared following the aforementioned synthetic method with **Compound 64** being used in place of **Compound 62**.

^{1}H NMR (500 MHz, $CDCl_3$) δ 7.03 - 6.96 (m, 4H), 4.92 (d, *J* = 5.6 Hz, 2H), 4.64 (d, *J* = 5.7 Hz, 2H), 2.81 – 2.70 (m, 2H), 2.29 – 2.21 (m, 2H), 2.07 – 1.95 (m, 2H), 1.82 (td, *J* = 11.6, 2.5 Hz, 2H), 1.61 – 1.52 (m, 2H), 1.35 – 1.13 (m, 5H), 0.89 (d, *J* = 6.4 Hz, 3H).

^{13}C NMR (126 MHz, $CDCl_3$) δ 161.39 (d, *J* = 244.9 Hz), 140.74 (d, *J* = 3.3 Hz), 127.40 (d, *J* = 7.9 Hz), 115.40 (d, *J* = 21.3 Hz), 81.95, 59.07, 54.17, 47.01, 39.21, 34.27, 30.87, 22.25, 21.97.

^{19}F NMR (471 MHz, $CDCl_3$) δ -116.62.

HRMS (ESI-TOF) m/z: calc'd for $C_{18}H_{27}FNO$ $[M+H]^+$: 292.2072; found 292.2074.

Scale-up

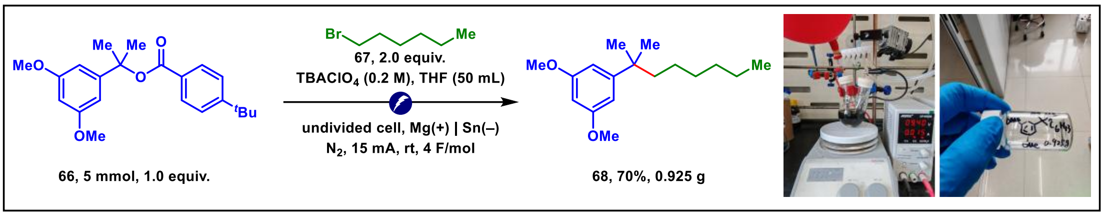

A two-necked round-bottom flask with a stir bar was charged with $TBAClO_4$ (0.2 M). The flask was evacuated and purged with nitrogen gas three times, followed by the addition of THF (50 mL), **Compound 66** (1.0 equiv., 5 mmol) and **Compound 67** (2.0 equiv., 10 mmol). The flask was equipped with anode (Mg plate) and cathode (Sn plate). Pre-stirring the resulting mixture for 5 minutes, and then the reaction mixture was electrolyzed at a constant current of 15 mA (current density: ~ 1.2 mA/cm^2) until passing 4 F/mol of charge at room temperature. After electrolysis, the crude mixture was further diluted with Et_2O and then filtered through a plug of silica gel. The solvent was removed under reduced pressure using a rotatory evaporator. The crude material was purified by silica gel column chromatography (silica, 50:1, PE: EA) to afford the desired **Compound 68** (0.925 g, 70%) as a colorless oil.

Compound 66

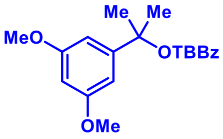

Prepared following the **General procedure 2** using 1-(3,5-dimethoxyphenyl)ethan-1-one (1.0 equiv., 5 mmol), MeMgBr (3.0 M in Et_2O, 1.1 equiv., 5.5 mmol), TBBzCl (1.2 equiv., 6 mmol) and purified by silica gel column chromatography to afford the desired **Compound 66** (0.82 g, 46%) as a pale yellow oil.

^{1}H NMR (500 MHz, $CDCl_3$) δ 7.98 (d, J = 8.5 Hz, 2H), 7.46 (d, J = 8.5 Hz, 2H), 6.58 (d, J = 2.2 Hz, 2H), 6.36 (t, J = 2.2 Hz, 1H), 3.77 (s, 6H), 1.88 (s, 6H), 1.35 (s, 9H).

^{13}C NMR (126 MHz, $CDCl_3$) δ 165.28, 160.82, 156.42, 148.84, 129.56, 128.86, 125.40, 103.23, 98.35, 81.89, 55.38, 35.18, 31.28, 28.90.

HRMS (ESI-TOF) m/z: calc'd for $C_{22}H_{29}O_4$ $[M+H]^+$: 357.2061; found 357.2061.

Compound 68

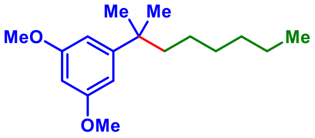

^{1}H NMR (400 MHz, $CDCl_3$) δ 6.49 (d, J = 2.3 Hz, 2H), 6.30 (t, J = 2.3 Hz, 1H), 3.80 (s, 6H), 1.61 – 1.50 (m, 2H), 1.26 (s, 6H), 1.19 (dt, J = 7.8, 3.9 Hz, 6H), 1.10 – 1.02 (m, 2H), 0.85 (t, J = 6.9 Hz, 3H).

^{13}C NMR (101 MHz, $CDCl_3$) δ 160.55, 152.73, 104.78, 96.67, 55.33, 44.64, 38.12, 31.91, 30.16,

29.10, 24.77, 22.81, 14.22.

HRMS (ESI-TOF) m/z: calc'd for $C_{17}H_{29}O_2$ $[M+H]^+$: 265.2163; found 265.2167.

Unsuccessful or Low Yielding Substrates

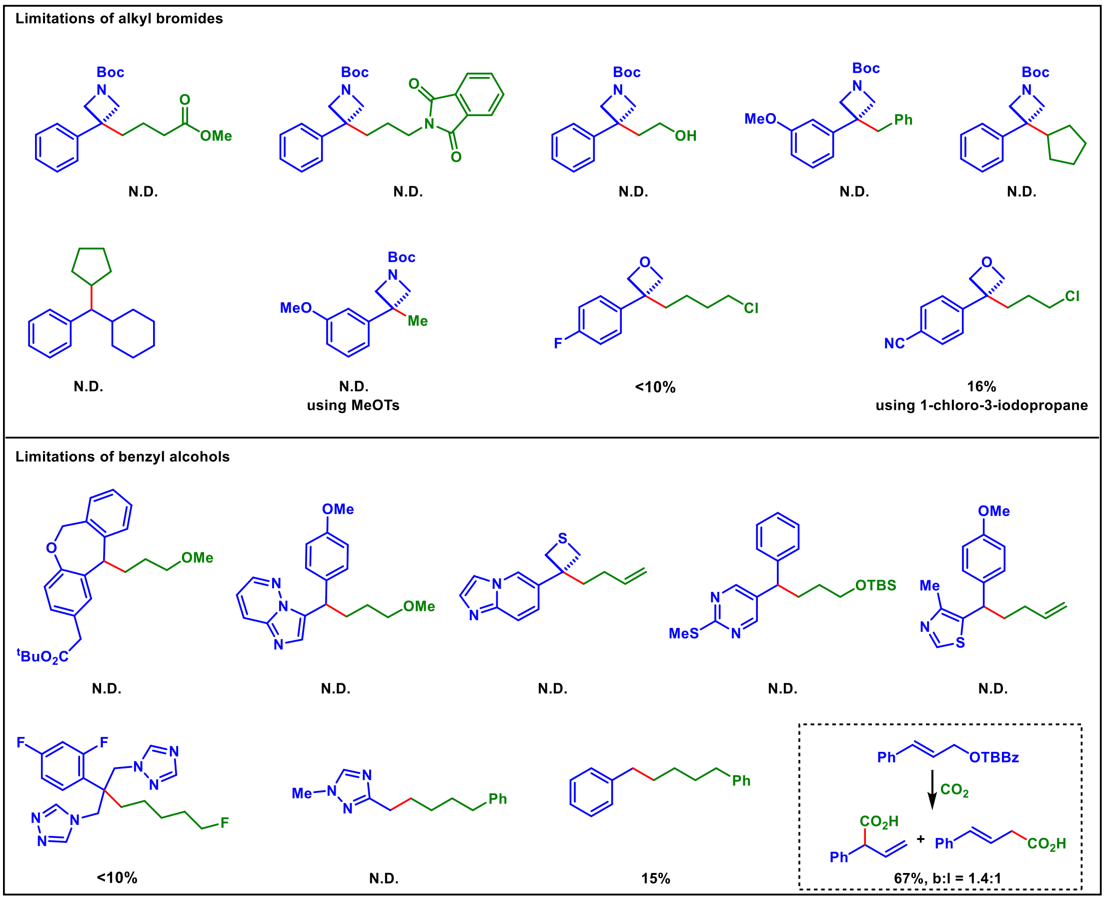

Cyclic Voltammetry Studies

All Cyclic Voltammetry studies were conducted under a nitrogen atmosphere. Measurements were performed in 0.1 M TBAClO$_4$ in THF (3 mL) using an undivided three-compartment cell. The cyclic voltammograms were recorded on a Signal 1000E instrument using a glassy carbon disk working electrode (diameter, 3 mm), a Pt wire auxiliary electrode, a commercial aqueous Ag/AgCl reference electrode (3.5 M KCl), and a scan rate of 100 mV/s.

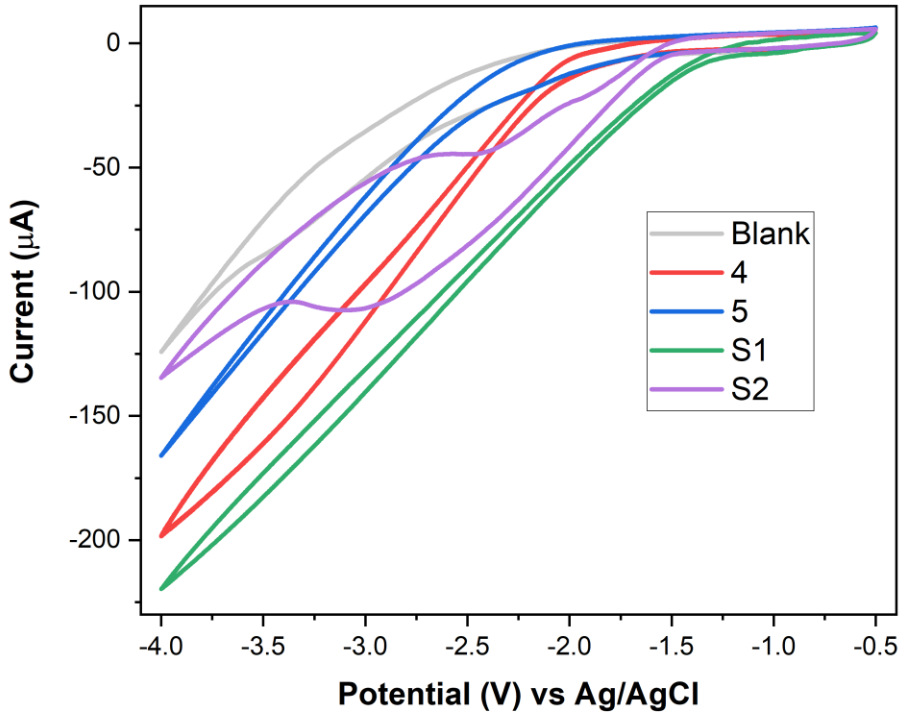

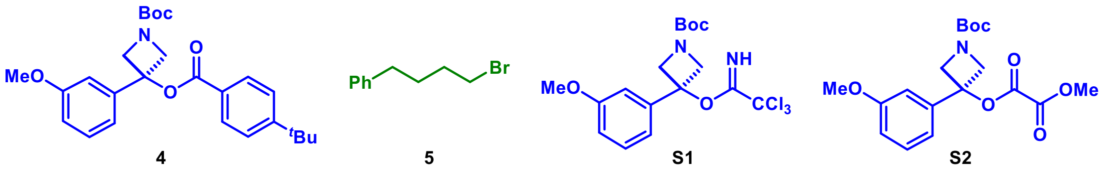

4 5 S1 S2

Fig. S2. Cyclic voltammograms of **5** (primary alkyl bromide, 20 mM) and activated benzyl alcohols (20 mM). The on-set potential for the reduction of **4** is around –2.1 V. The on-set potential for the reduction of **5** is around –2.4 V. The on-set potential for the reduction of **S1** is around –1.4 V. The on-set potential for the reduction of **S2** is around –1.6 V and the $E_{p/2}$ is approximately -2.2 V.

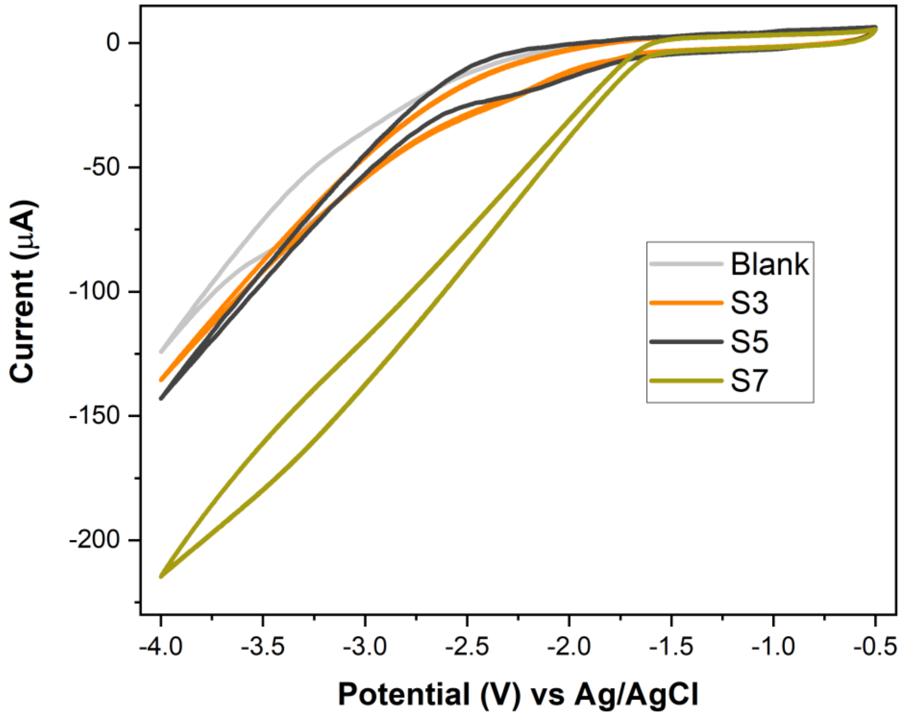

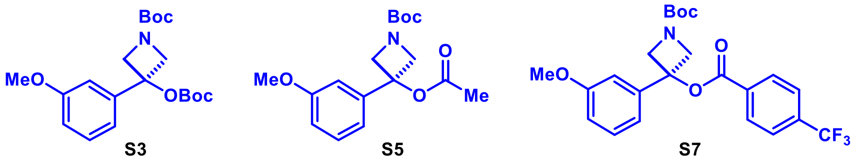

Fig. S3. Cyclic voltammograms of activated benzyl alcohols (20 mM). The on-set potential for the reduction of **S3** is around –2.6 V. The on-set potential for the reduction of **S5** is around –2.5 V. The on-set potential for the reduction of **S7** is around –1.8 V.

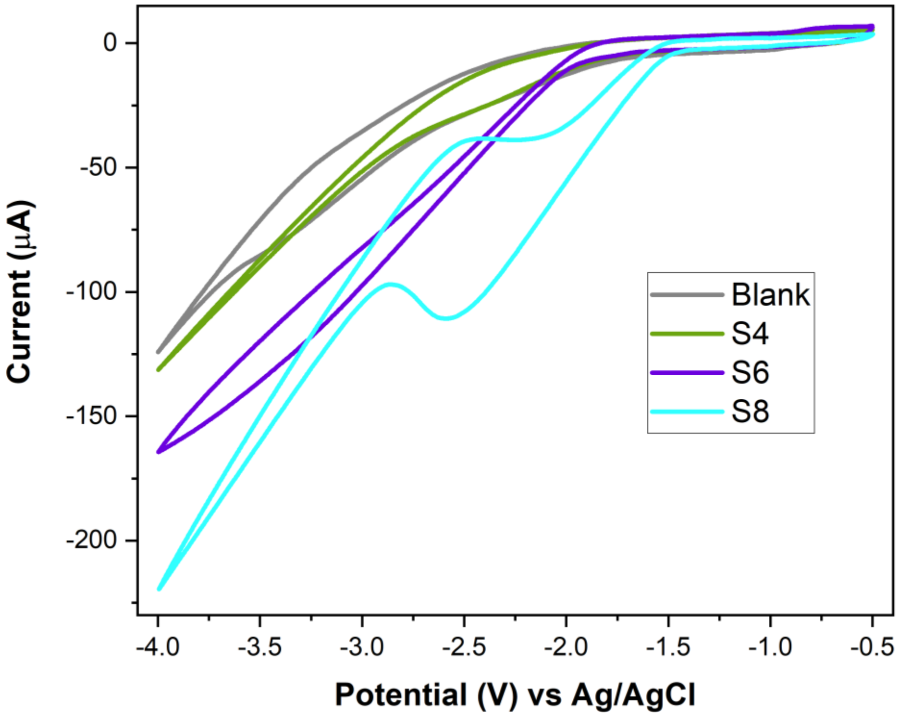

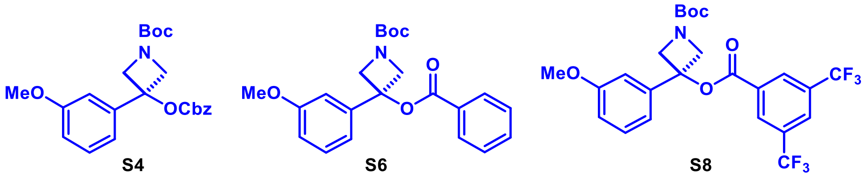

S4 S6 S8

Fig. S4. Cyclic voltammograms of activated benzyl alcohols (20 mM). The on-set potential for the reduction of **S4** is around –2.5 V. The on-set potential for the reduction of **S6** is around –2.0 V. The on-set potential for the reduction of **S8** is around –1.6 V and the $E_{p/2}$ is approximately -2.0 V.

Mechanistic Studies

Radical clock experiments

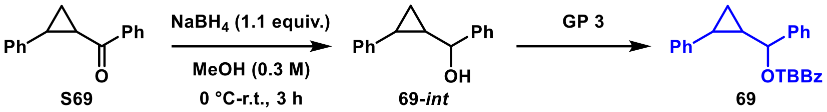

Compound S69 was prepared following the reported literature procedures (*87*).

In a 25 mL Schlenk flask equipped with a magnetic stir bar, **Compound S69** (1.0 equiv., 5 mmol) and MeOH (0.3 M) were added, then $NaBH_4$ (1.1 equiv., 5.5 mmol) was added portionwise at 0 °C. The reaction mixture was removed from ice bath and stirred at room temperature 3 h. After the reaction, saturated NH_4Cl(aq.) was added, and the mixture was extracted with EtOAc. The combined organic phase was dried over anhydrous sodium sulfate, filtered, and concentrated in vacuo. The crude product was used for the next step without further purification.

Compound 69 was prepared following the **General procedure 3** using the crude alcohol, TEA (2.0 equiv., 10 mmol), DMAP (10 mol%, 0.5 mmol), TBBzCl (1.2 equiv., 6 mmol) and purified by silica gel column chromatography to afford the desired **Compound 69** (1.33 g, 69%, 2 steps, dr = 2.4:1) as a colorless oil.

Compound 69

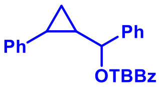

^{1}H NMR (500 MHz, $CDCl_3$) δ 8.11 – 7.93 (m, 2H), 7.55 – 7.43 (m, 4H), 7.39 – 7.27 (m, 3H), 7.27 – 7.20 (m, 2H), 7.18 – 7.13 (m, 1H), 7.11 – 6.96 (m, 2H), 5.75 (d, *J* = 8.1 Hz, 1H), 2.17 (dt, *J* = 9.7, 5.1 Hz, 1H), 1.81 – 1.66 (m, 1H), 1.33 (s, 9H), 1.20 – 1.01 (m, 2H).

^{13}C NMR (126 MHz, $CDCl_3$) δ 166.05, 156.87, 156.81, 142.08, 141.81, 140.27, 140.24, 129.77, 129.75, 128.66, 128.63, 128.46, 128.14, 128.07, 127.71, 127.69, 126.70, 126.65, 126.28, 125.95, 125.90, 125.54, 125.50, 79.09, 78.30, 35.23, 35.21, 31.25, 31.24, 28.22, 27.83, 22.49, 21.49, 14.19, 13.33.

HRMS (ESI-TOF) m/z: calc'd for $C_{27}H_{28}NaO_2$ $[M+Na]^+$: 407.1982; found 407.1981.

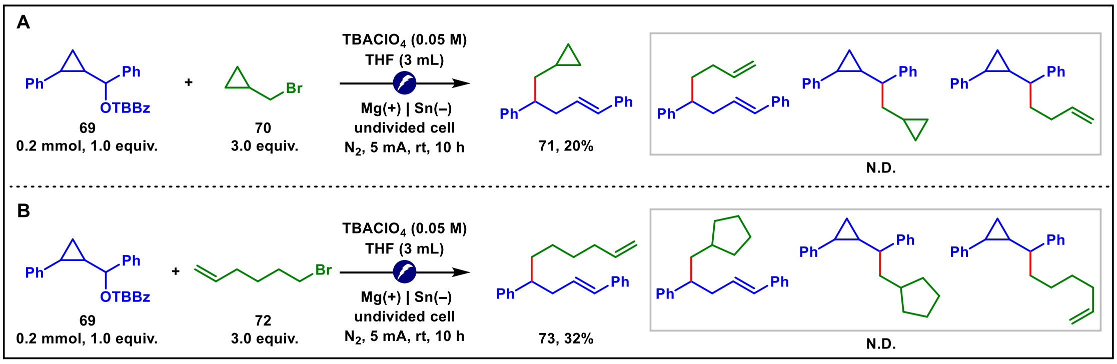

To probe for radical intermediates, **Compound 69** was reacted separately with **Compound 70** and **Compound 72** under our e-XEC conditions. In both cases, only the deoxygenative ring-opening

S$_N$2 products **71** and **73** were obtained, indicating that while the activated benzyl alcohol derivatives likely proceed through radical pathways, the primary alkyl halides do not participate in radical processes.

Compound 71

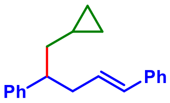

^{1}H NMR (500 MHz, CDCl$_3$) δ 7.29 (t, *J* = 7.6 Hz, 3H), 7.27 – 7.13 (m, 7H), 6.34 (dt, *J* = 15.8, 1.4 Hz, 1H), 6.07 (ddd, *J* = 15.8, 7.8, 6.6 Hz, 1H), 2.82 (tt, *J* = 8.5, 6.1 Hz, 1H), 2.63 – 2.45 (m, 2H), 1.70 (ddd, *J* = 14.4, 8.9, 5.9 Hz, 1H), 1.41 (ddd, *J* = 13.7, 7.9, 5.6 Hz, 1H), 0.54 (qq, *J* = 8.0, 5.1 Hz, 1H), 0.41 – 0.19 (m, 2H), 0.04 – 0.14 (m, 2H).
^{13}C NMR (126 MHz, CDCl$_3$) δ 145.65, 137.89, 131.21, 129.28, 128.56, 128.37, 127.89, 126.98, 126.13, 126.10, 46.82, 41.43, 40.12, 9.42, 4.82, 4.72.
HRMS (ESI-TOF) m/z: calc'd for $C_{20}H_{22}Na$ [M+Na]$^+$: 285.1614; found 285.1617.

Compound 73

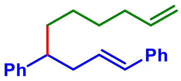

^{1}H NMR (500 MHz, CDCl$_3$) δ 7.32 – 7.27 (m, 3H), 7.26 – 7.14 (m, 7H), 6.33 (dt, *J* = 15.8, 1.4 Hz, 1H), 6.07 (dt, *J* = 15.7, 7.2 Hz, 1H), 5.75 (ddt, *J* = 16.9, 10.2, 6.6 Hz, 1H), 5.00 – 4.83 (m, 2H), 2.72 – 2.60 (m, 1H), 2.52 – 2.44 (m, 2H), 2.04 – 1.92 (m, 2H), 1.78 – 1.67 (m, 1H), 1.66 – 1.55 (m, 1H), 1.40 – 1.28 (m, 2H), 1.25 – 1.11 (m, 2H).
^{13}C NMR (126 MHz, CDCl$_3$) δ 145.48, 139.17, 137.89, 131.22, 129.23, 128.56, 128.43, 127.80, 126.99, 126.15, 126.10, 114.32, 46.38, 40.79, 35.81, 33.79, 29.11, 27.14.
HRMS (ESI-TOF) m/z: calc'd for $C_{22}H_{26}Na$ [M+Na]$^+$: 313.1927; found 313.1930.

Deuterium labeling experiments

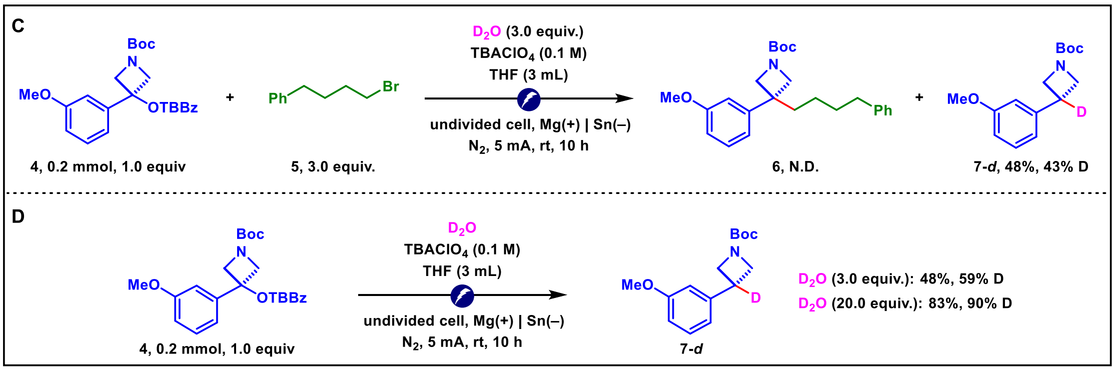

Deuterium-labeling experiments were carried out by introducing three equivalents of D_2O into the model reaction to probe the involvement of potential carbanion intermediates. This intervention led to two notable observations: (1) complete suppression of the S$_N$2 pathway, and (2) isolation of the deoxygenative deuteration product (48% yield, 43% D incorporation). Notably, in the absence of

alkyl bromide **5**, the yield of the deoxygenative deuteration product remained unchanged, while deuterium incorporation increased to 59%. Further increasing the amount of D_2O to 20 equivalents resulted in excellent product yield and high deuterium incorporation (83% yield, 90% D incorporation). These results suggested the involvement of a carbanion intermediate.

Compound 7-*d*

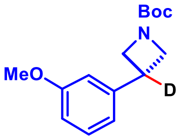

^{1}H NMR (500 MHz, $CDCl_3$) δ 7.29 – 7.24 (m, 1H), 6.90 (dt, *J* = 7.6, 1.3 Hz, 1H), 6.85 (dd, *J* = 2.7, 1.6 Hz, 1H), 6.80 (ddd, *J* = 8.2, 2.5, 1.1 Hz, 1H), 4.30 (d, *J* = 8.6 Hz, 2H), 3.97 (d, *J* = 8.6 Hz, 2H), 3.82 (s, 3H), 3.73 – 3.68 (m, 0.1H), 1.47 (s, 9H).
^{13}C NMR (101 MHz, $CDCl_3$) δ 160.03, 156.53, 143.94, 129.86, 119.19, 112.70, 112.29, 79.63, 56.46, 55.36, 33.28, 28.53.
HRMS (ESI-TOF) m/z: calc'd for $C_{15}H_{21}DNO_3$ $[M+H]^+$: 265.1657; found 265.2169.

NMR Spectra

^{1}H NMR of Compound 4 (400 MHz, $CDCl_3$):

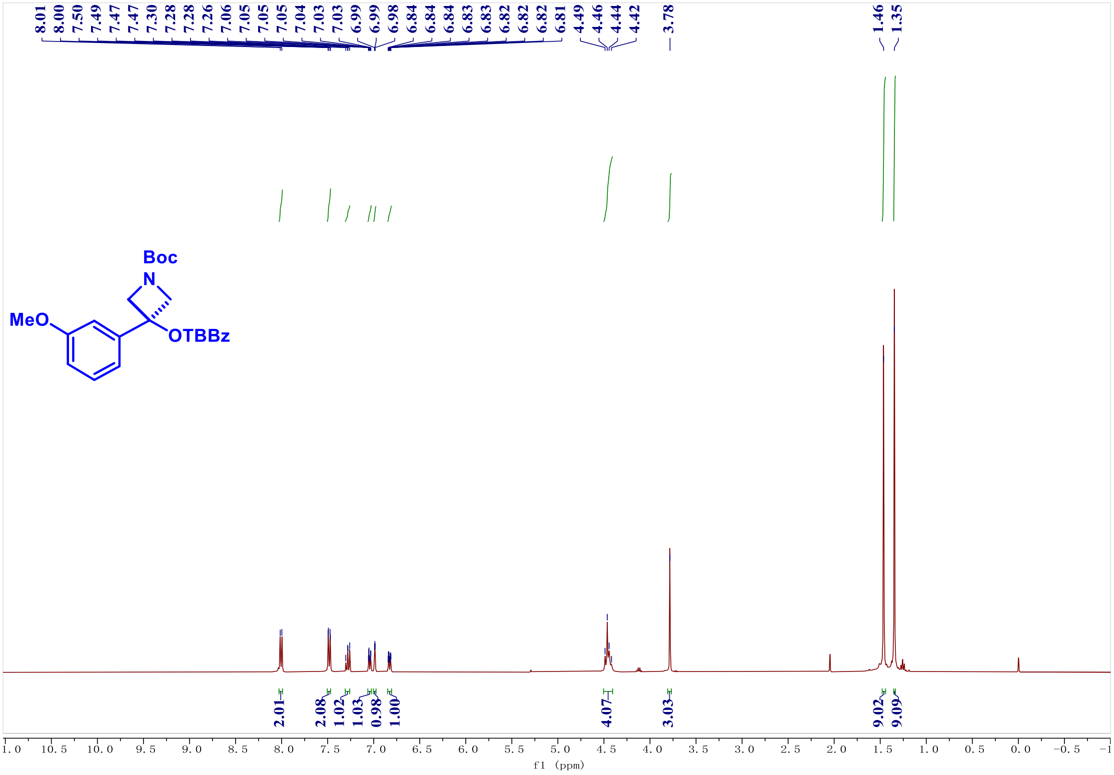

^{13}C NMR of Compound 4 (101 MHz, $CDCl_3$):

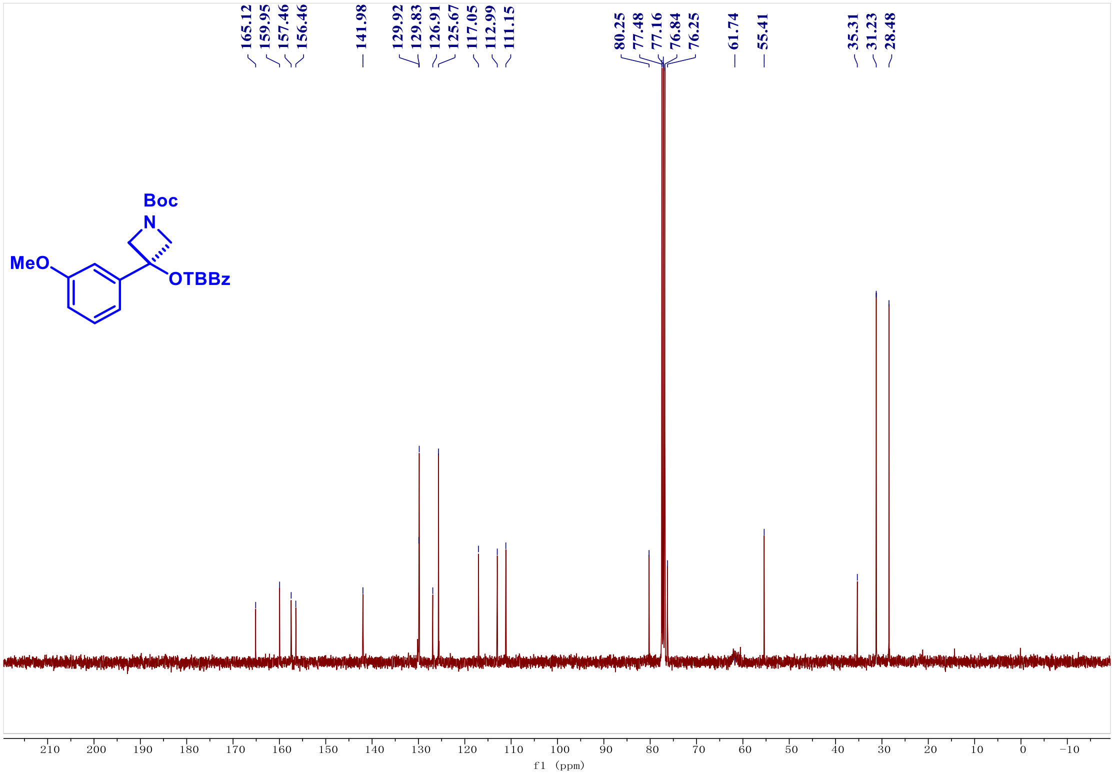

^{1}H NMR of Compound S1 (400 MHz, $CDCl_3$):

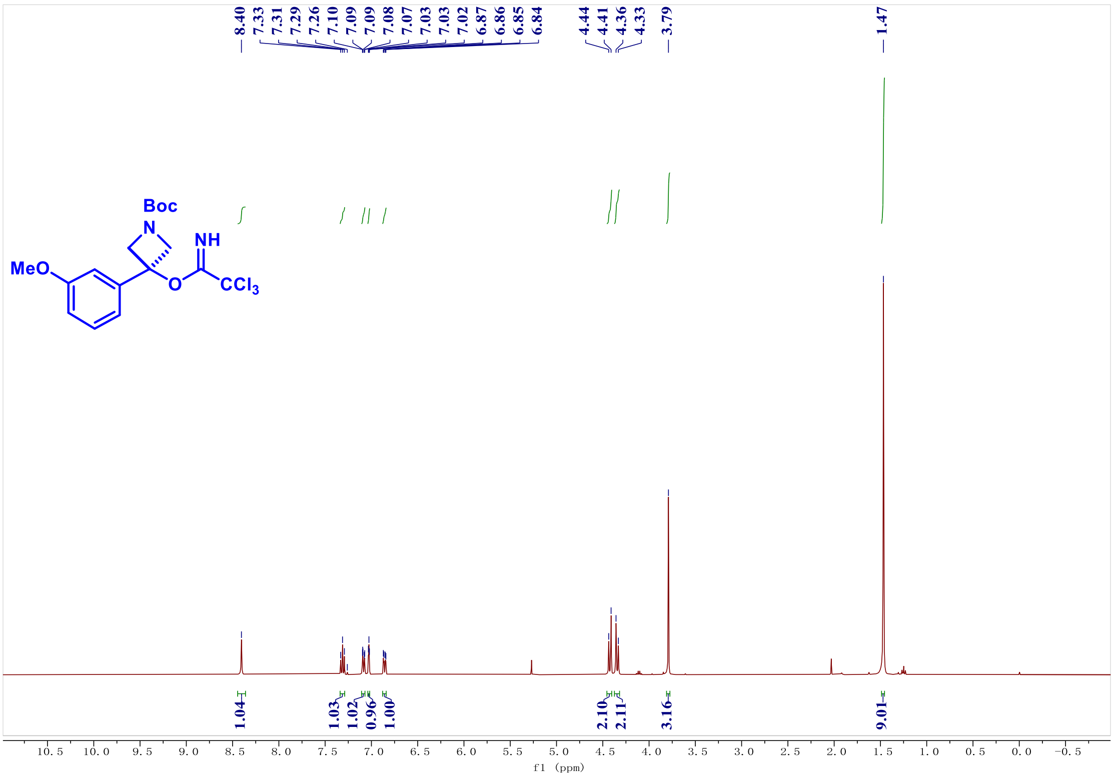

^{13}C NMR of Compound S1 (101 MHz, $CDCl_3$):

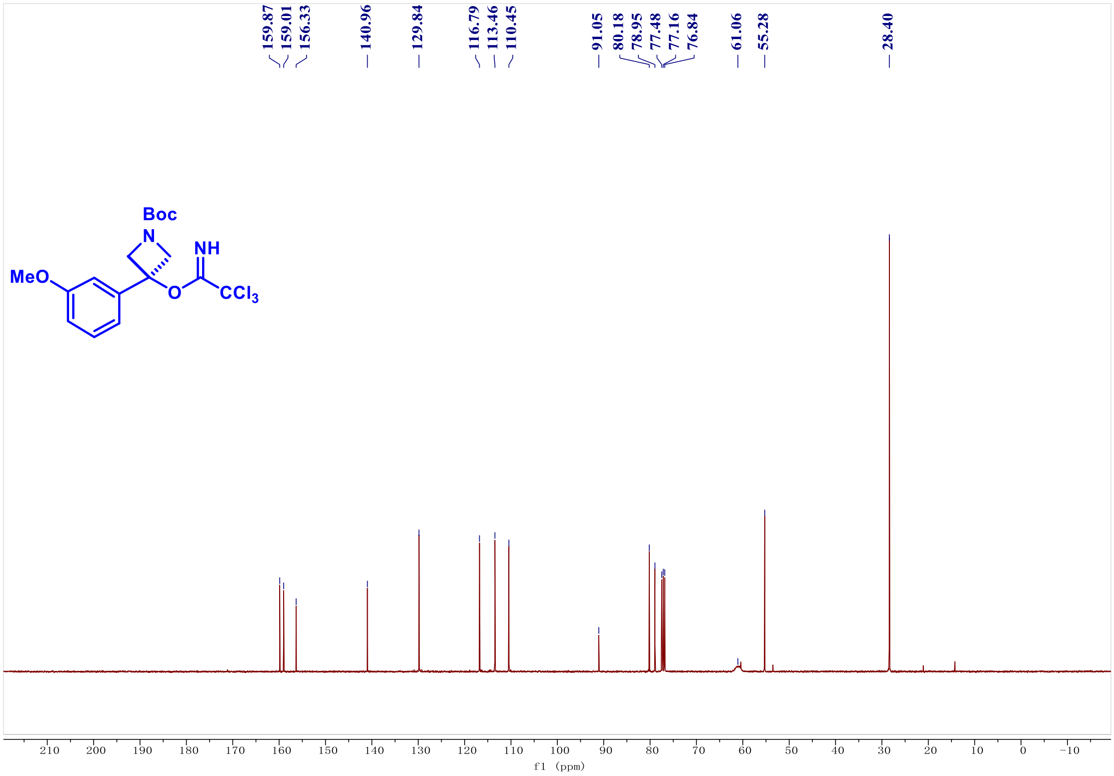

^{1}H NMR of Compound S2 (400 MHz, $CDCl_3$):

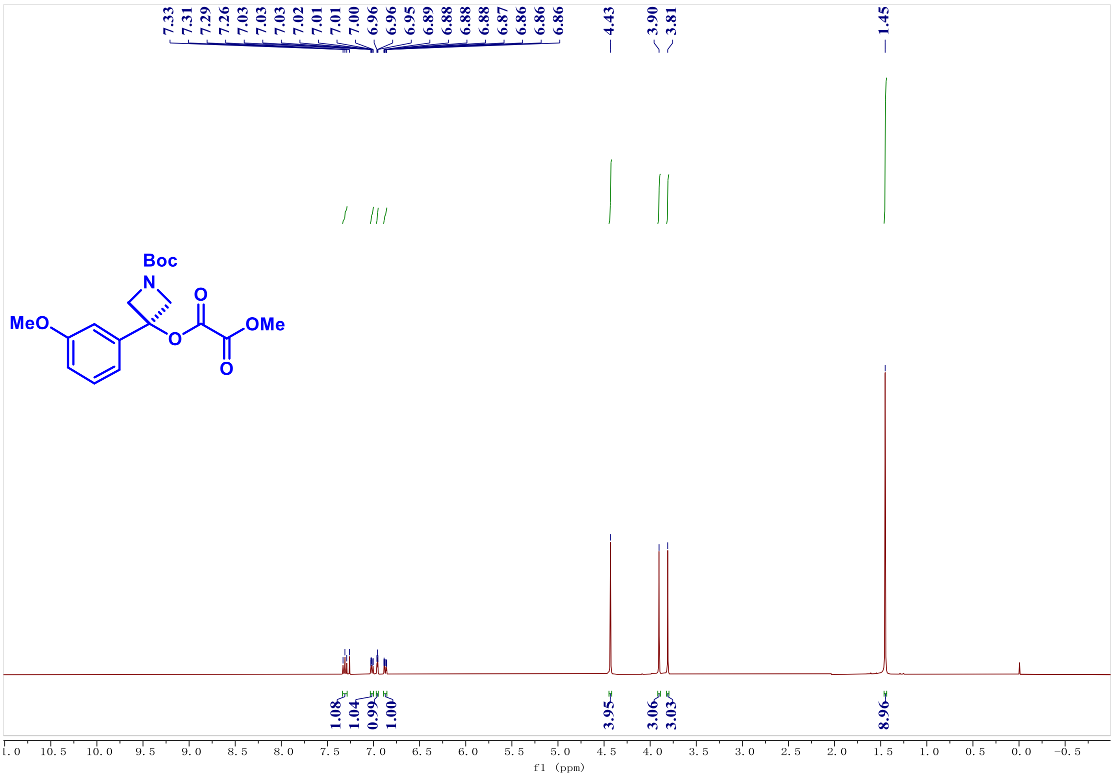

^{13}C NMR of Compound S2 (101 MHz, $CDCl_3$):

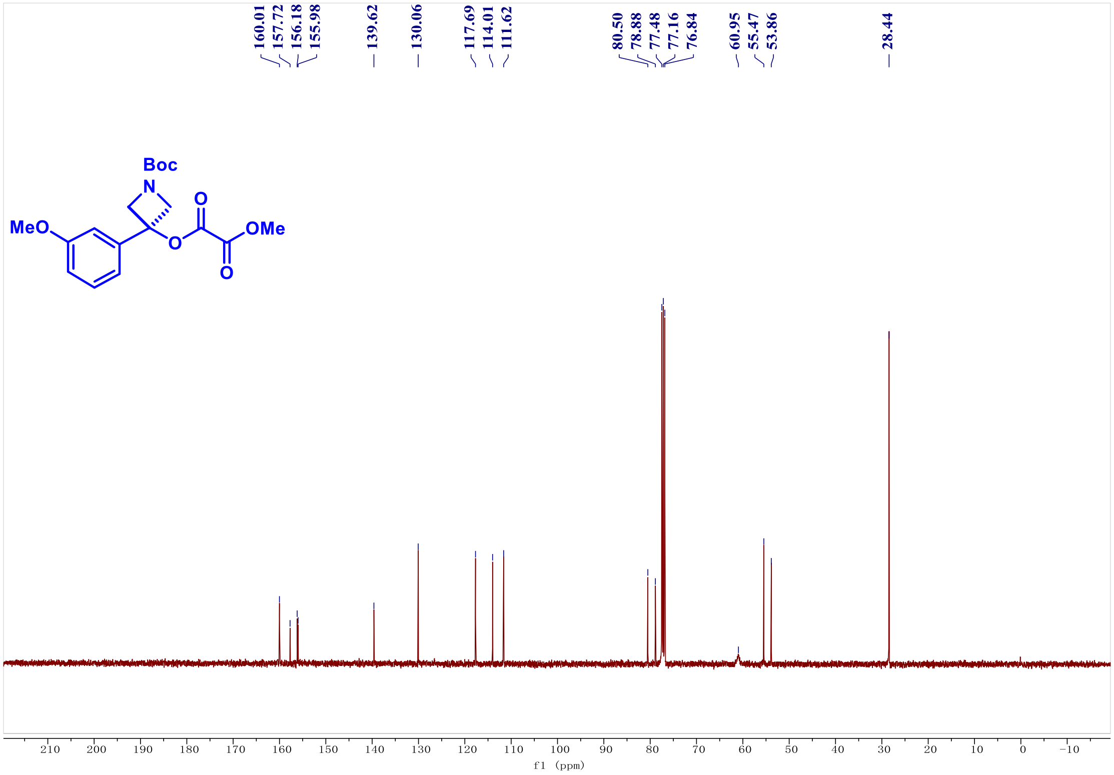

^{1}H NMR of Compound S3 (400 MHz, $CDCl_3$):

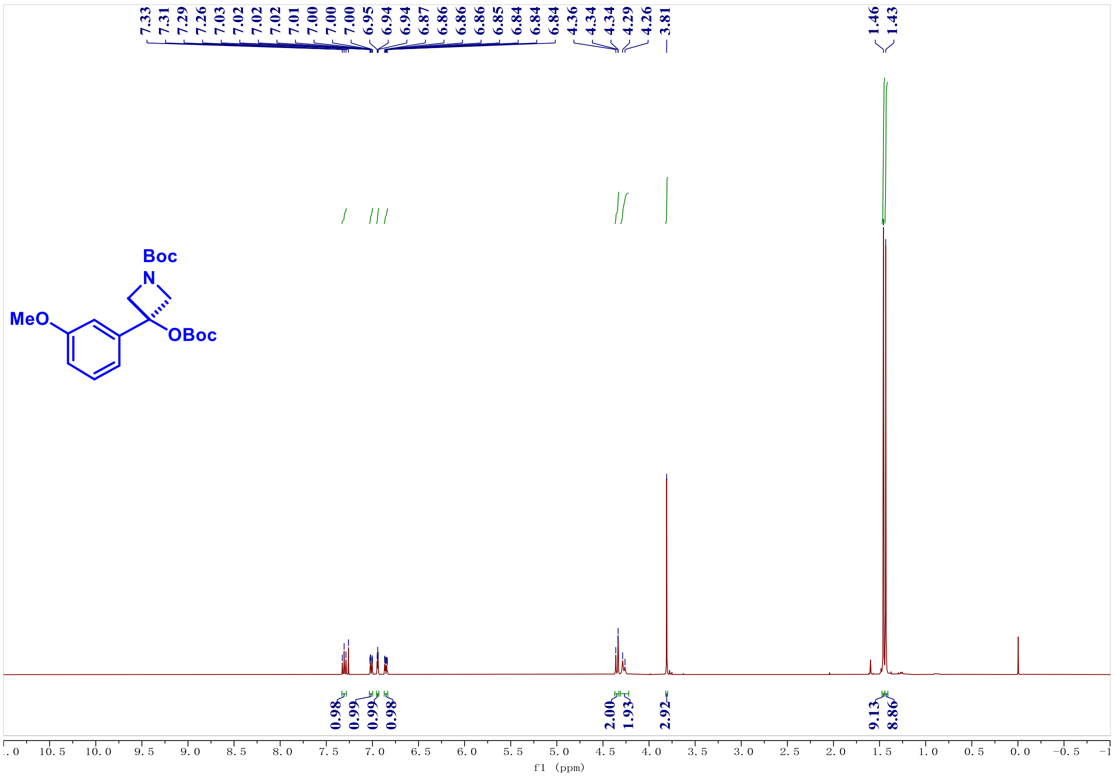

^{13}C NMR of Compound S3 (101 MHz, $CDCl_3$):

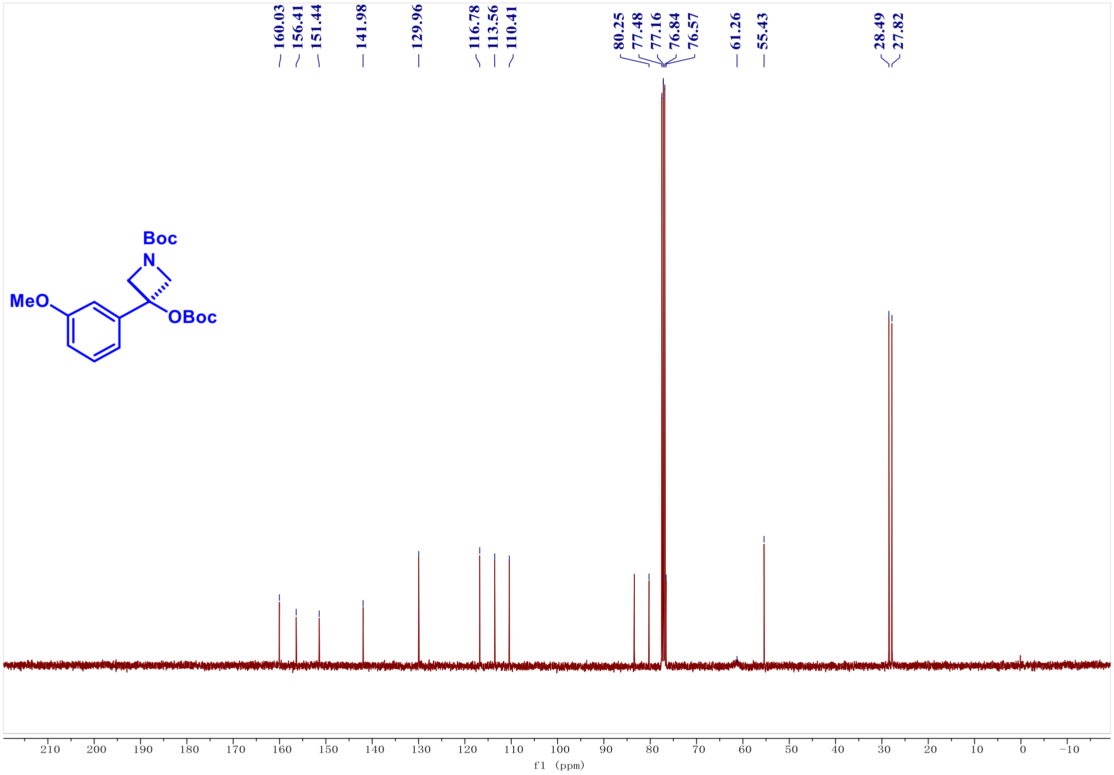

^{1}H NMR of Compound S4 (400 MHz, $CDCl_3$):

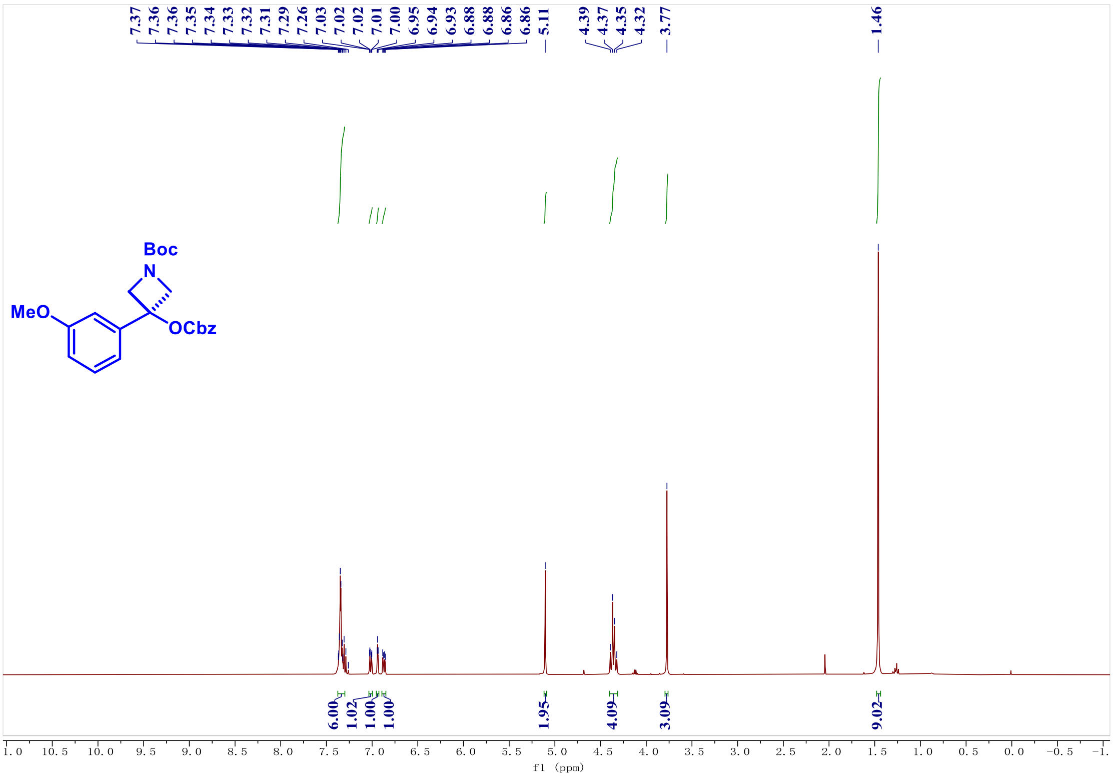

^{13}C NMR of Compound S4 (101 MHz, $CDCl_3$):

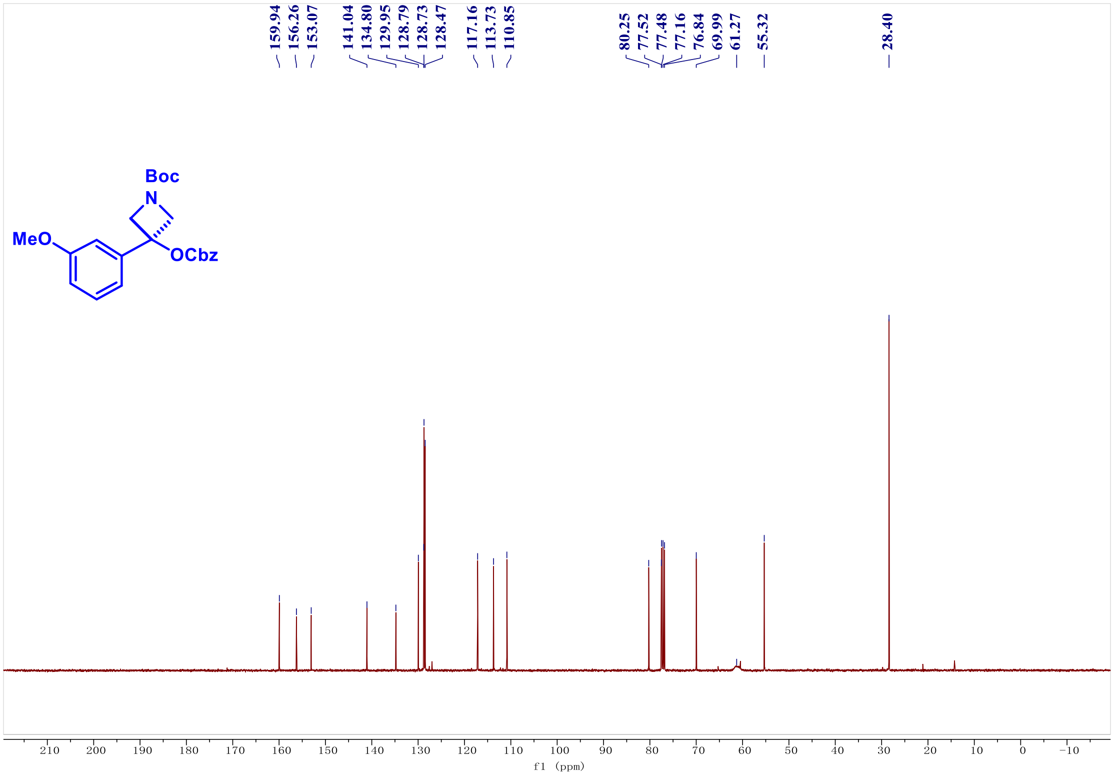

^{1}H NMR of Compound S5 (400 MHz, $CDCl_3$):

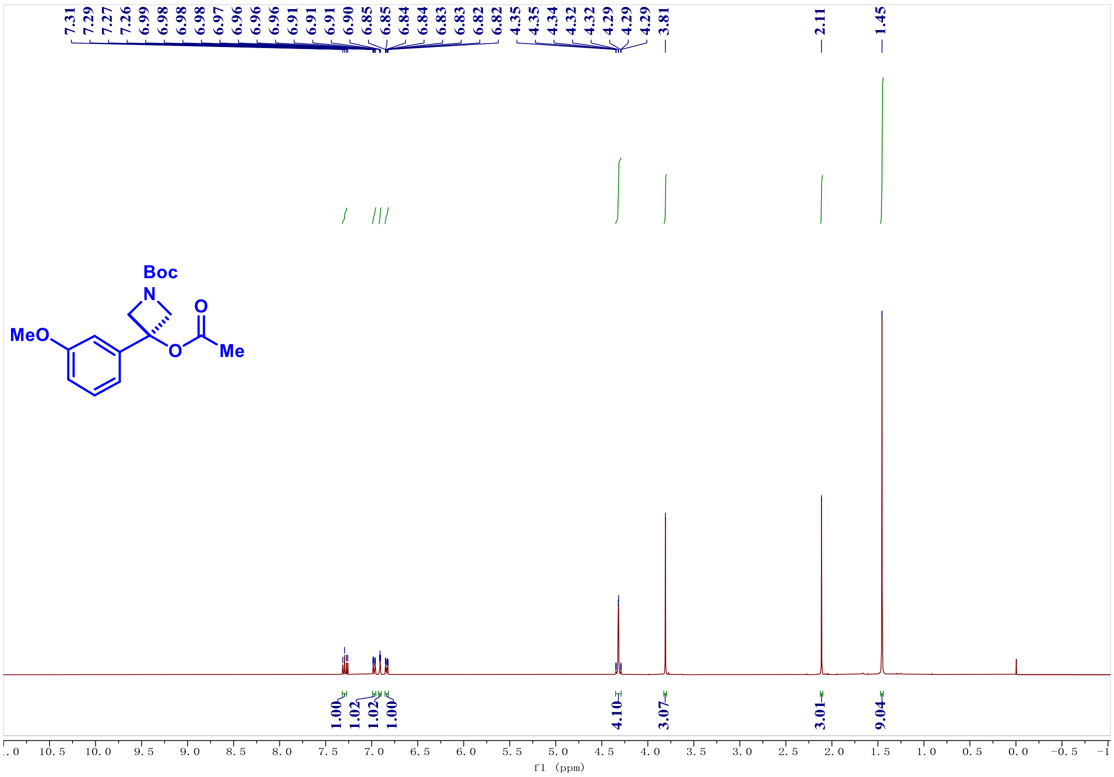

^{13}C NMR of Compound S5 (101 MHz, $CDCl_3$):

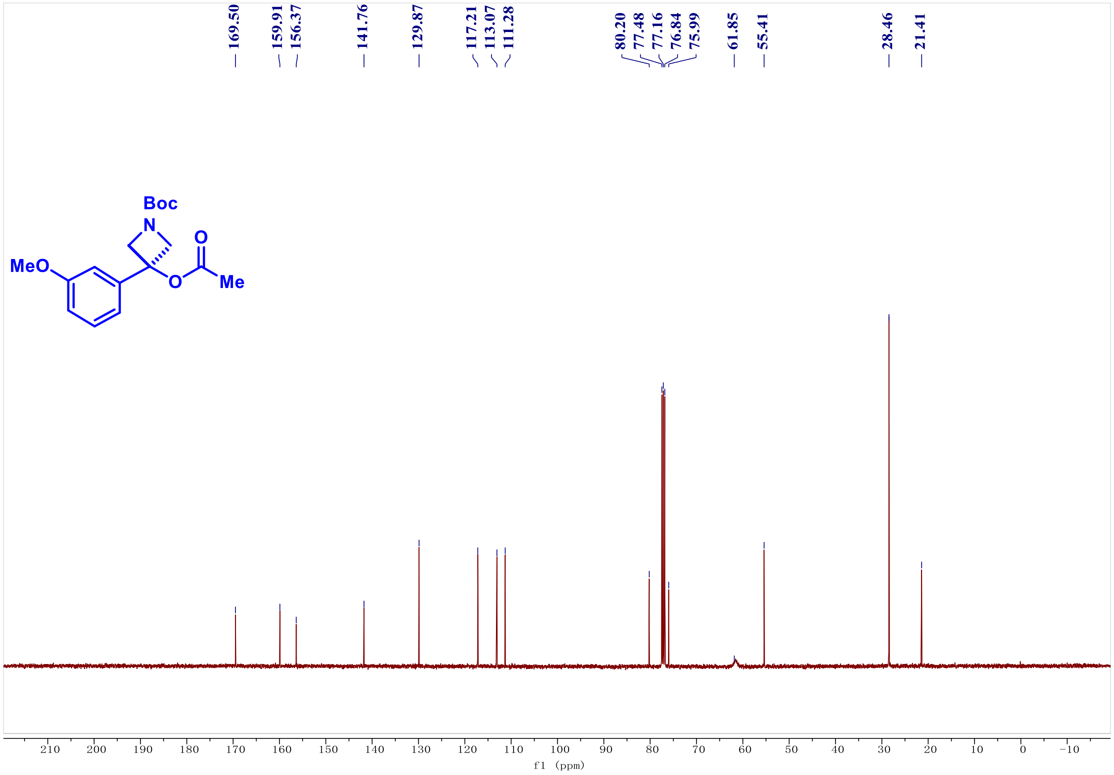

^{1}H NMR of Compound S6 (400 MHz, $CDCl_3$):

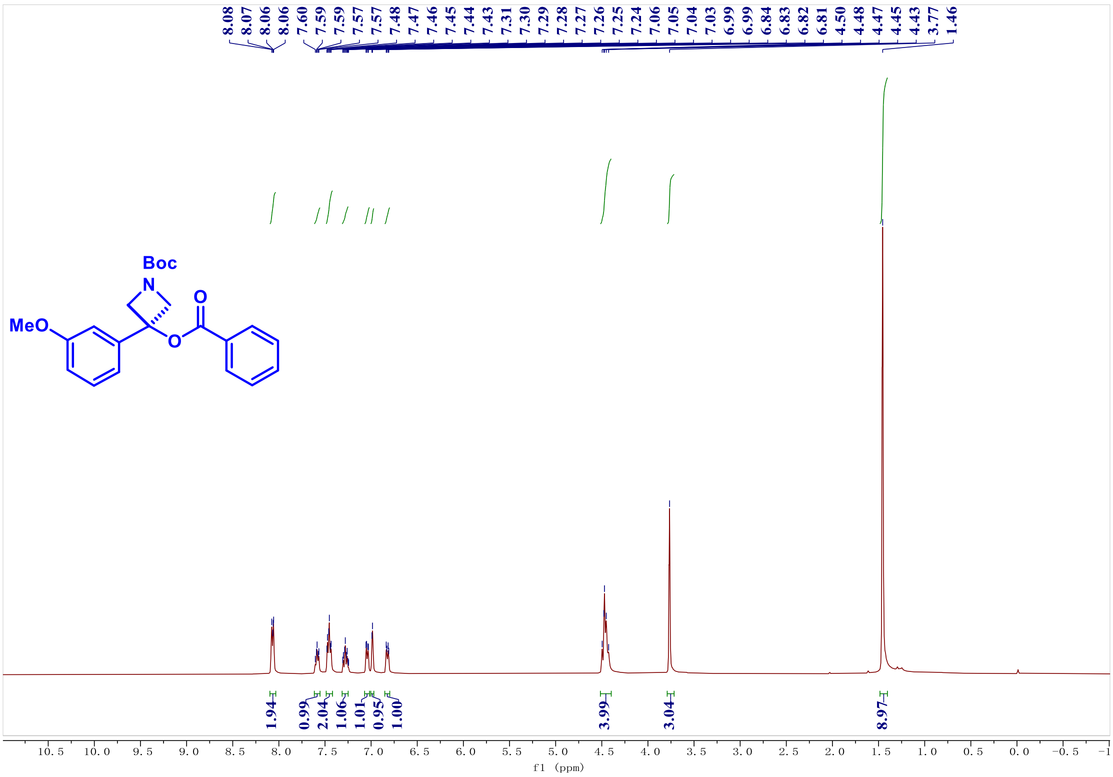

^{13}C NMR of Compound S6 (101 MHz, $CDCl_3$):

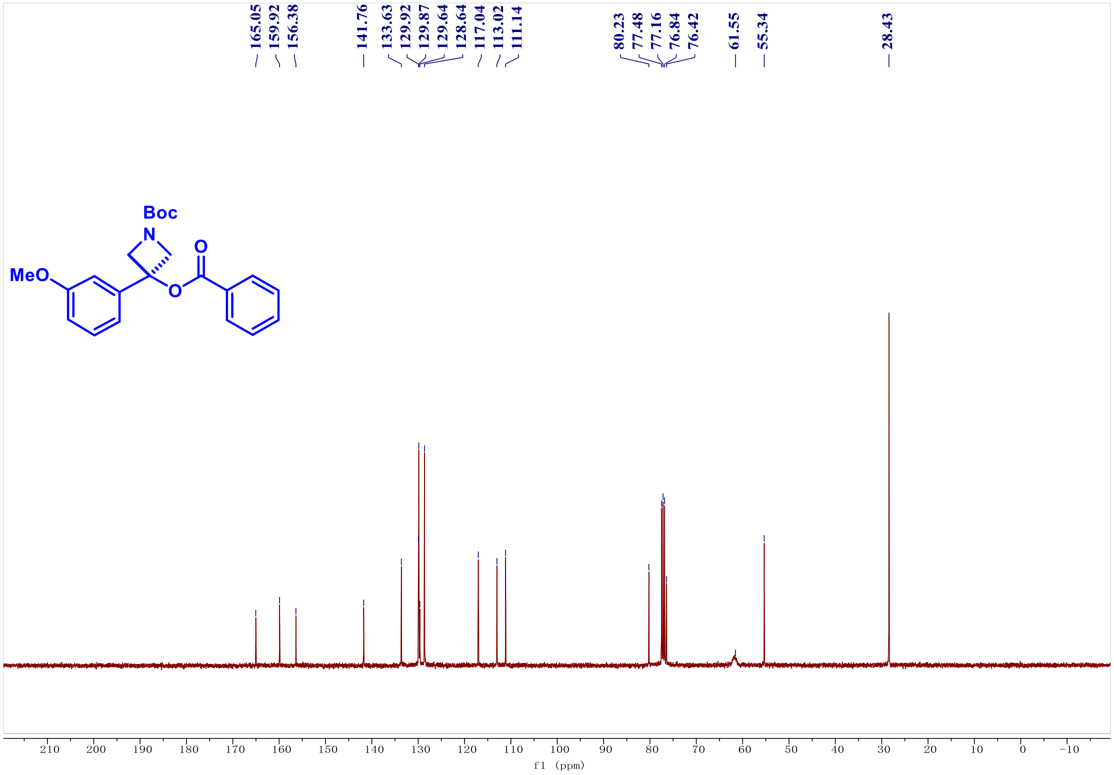

^{1}H NMR of Compound S7 (400 MHz, $CDCl_3$):

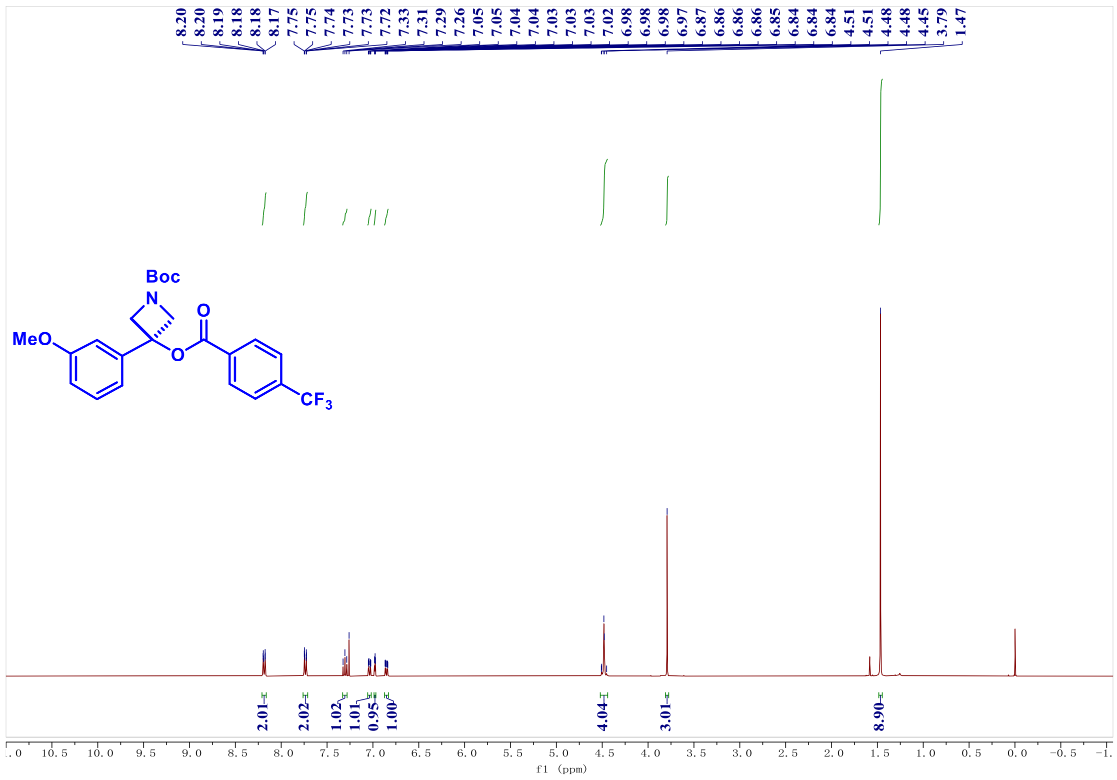

^{13}C NMR of Compound S7 (101 MHz, $CDCl_3$):

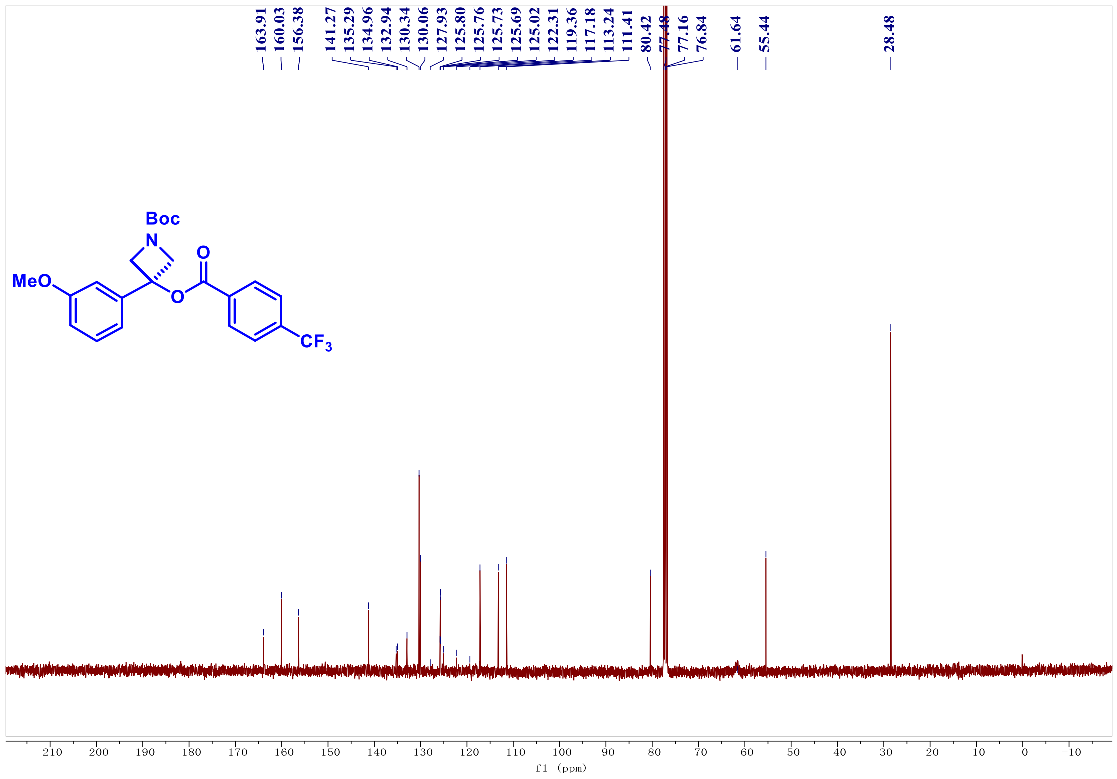

^{19}F NMR of Compound S7 (376 MHz, $CDCl_3$):

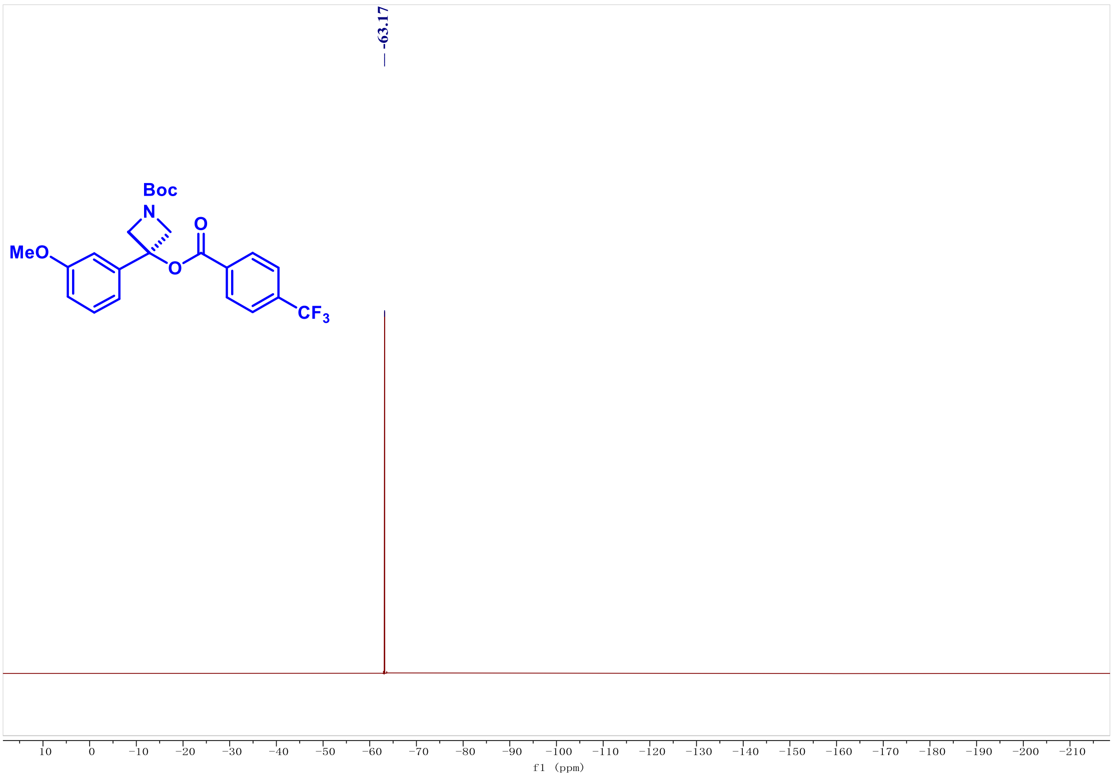

^{1}H NMR of Compound S8 (400 MHz, $CDCl_3$):

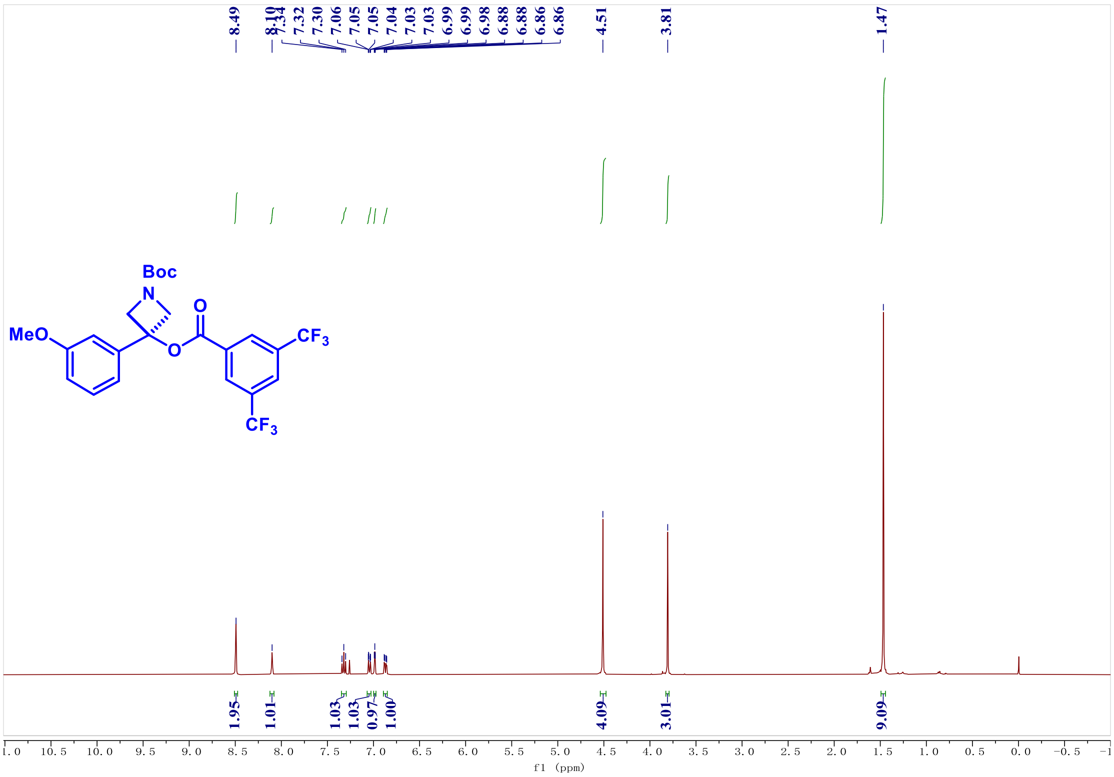

^{13}C NMR of Compound S8 (101 MHz, $CDCl_3$):

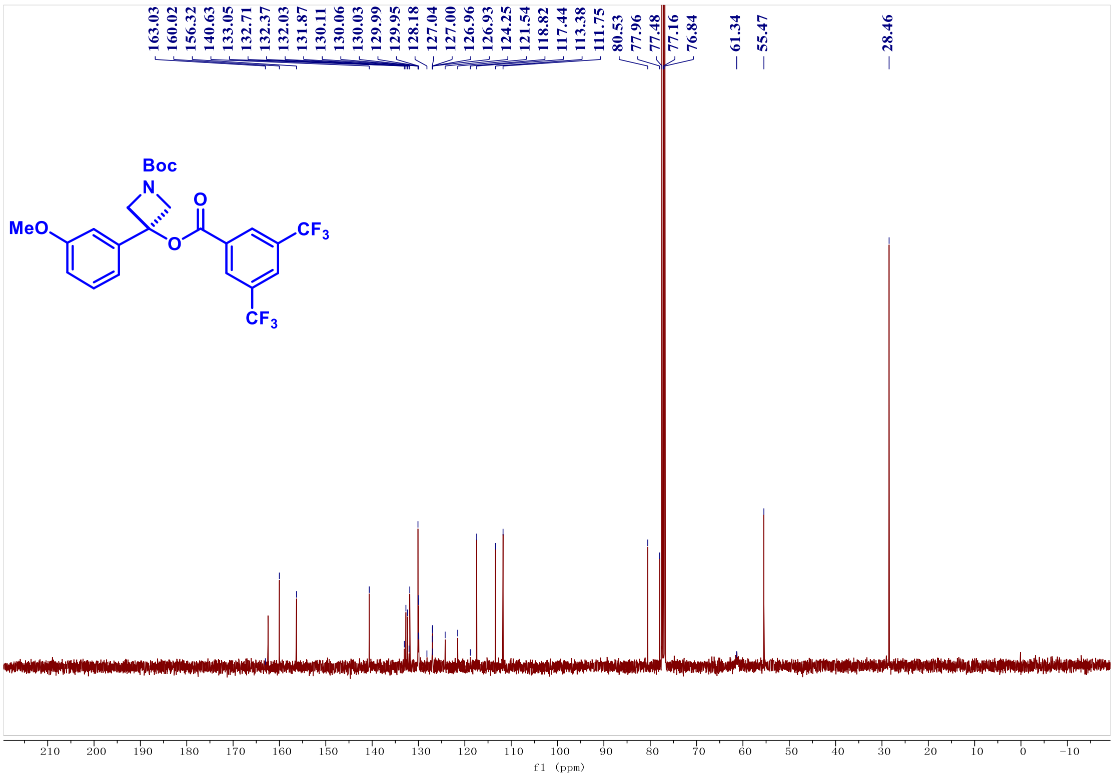

^{19}F NMR of Compound S8 (376 MHz, $CDCl_3$):

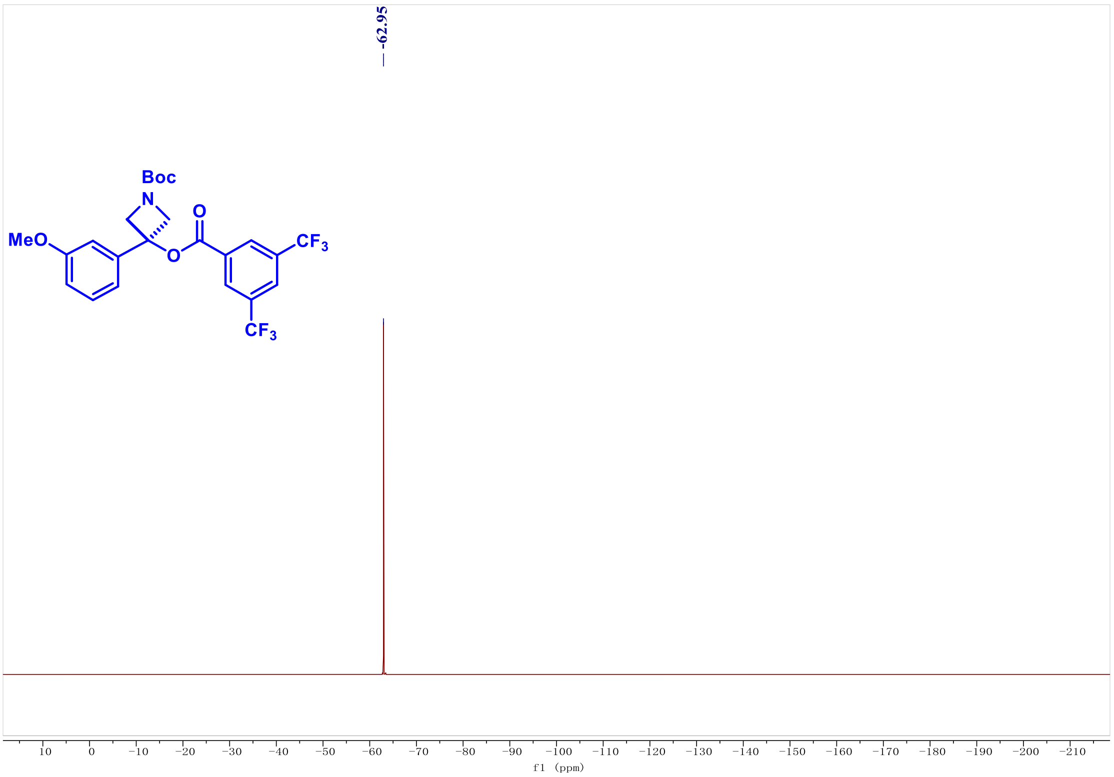

^{1}H NMR of Compound S9 (500 MHz, CDCl$_3$):

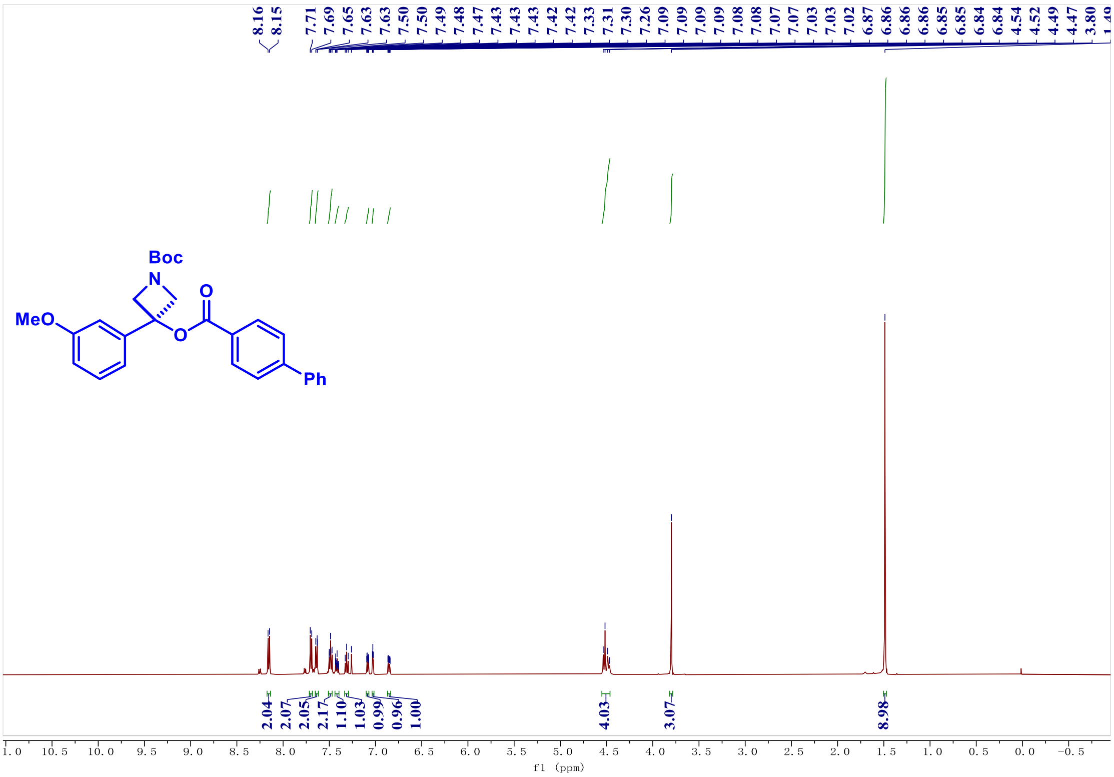

^{13}C NMR of Compound S9 (126 MHz, CDCl$_3$):

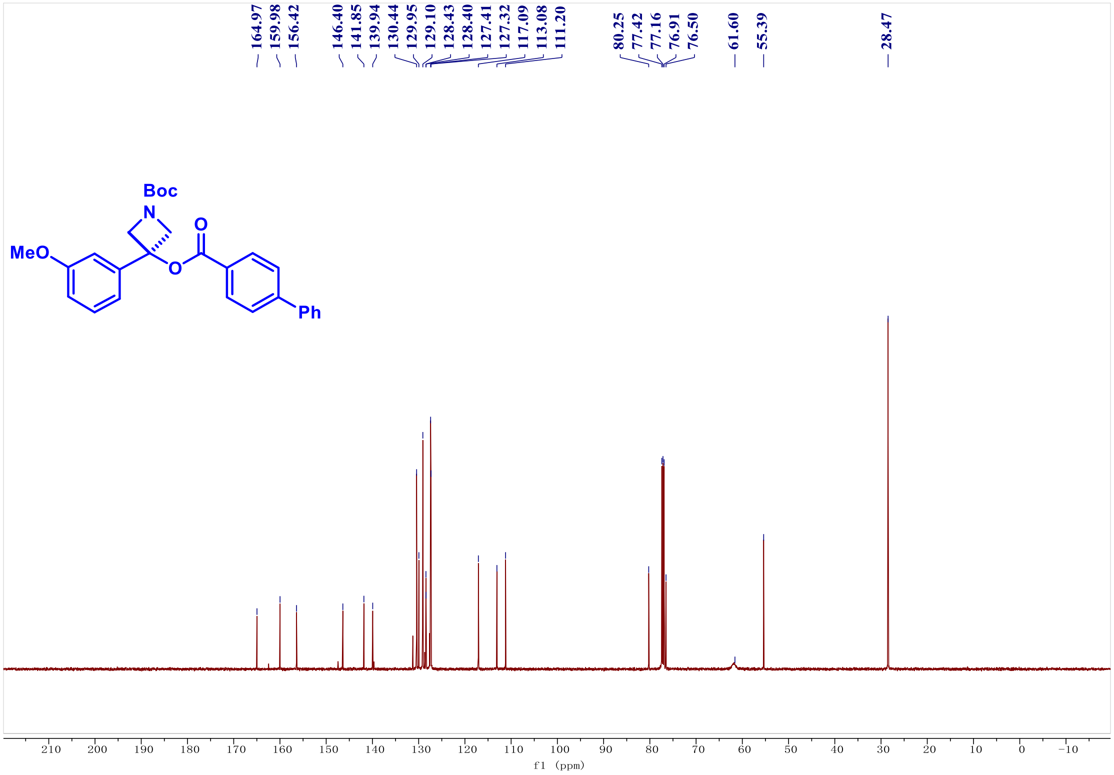

^{1}H NMR of Compound S10 (400 MHz, $CDCl_3$):

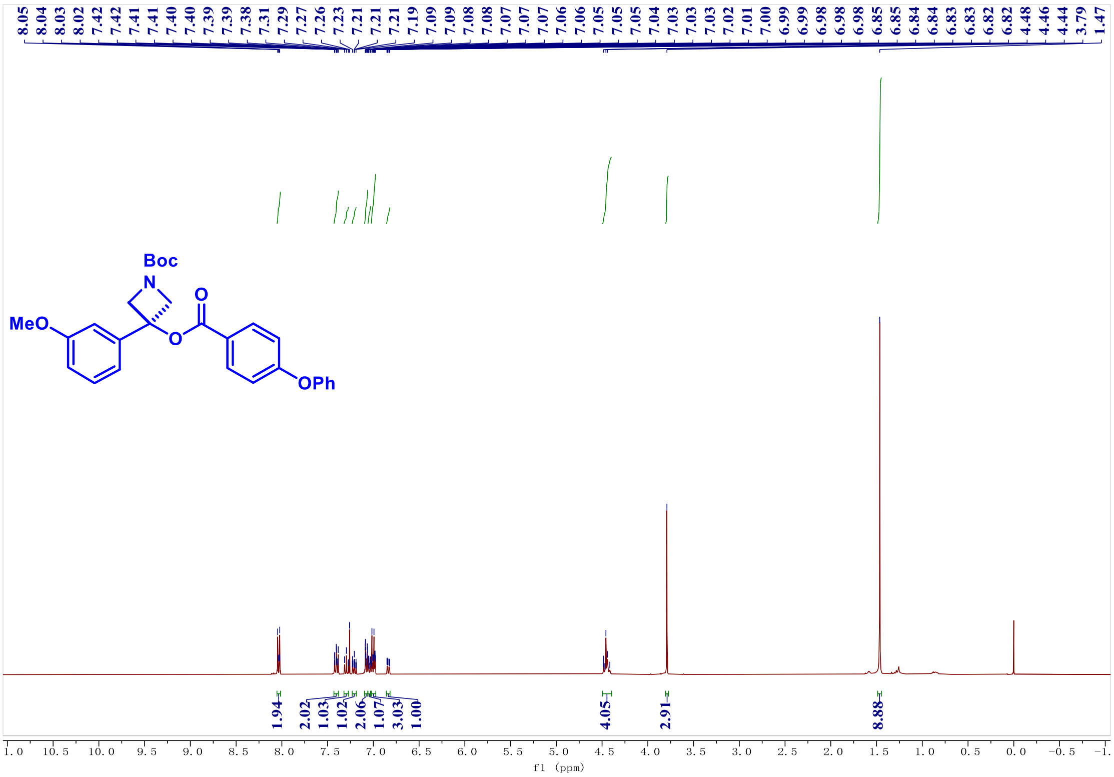

^{13}C NMR of Compound S10 (101 MHz, $CDCl_3$):

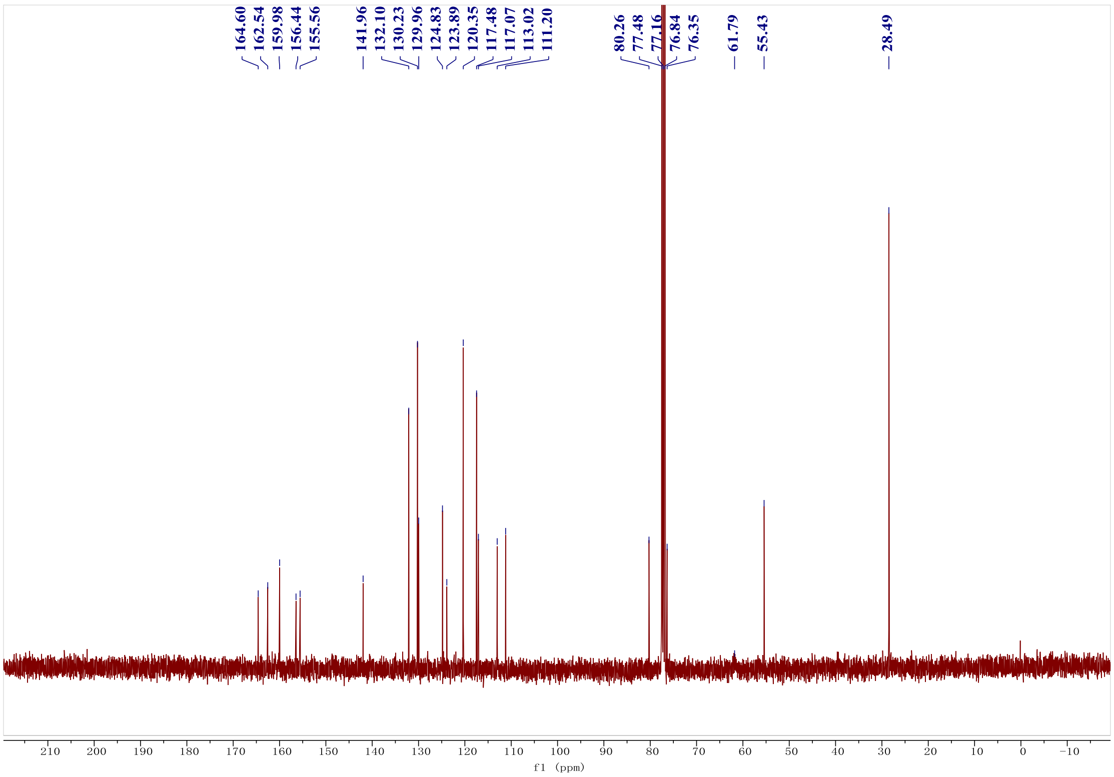

^{1}H NMR of Compound S11 (400 MHz, $CDCl_3$):

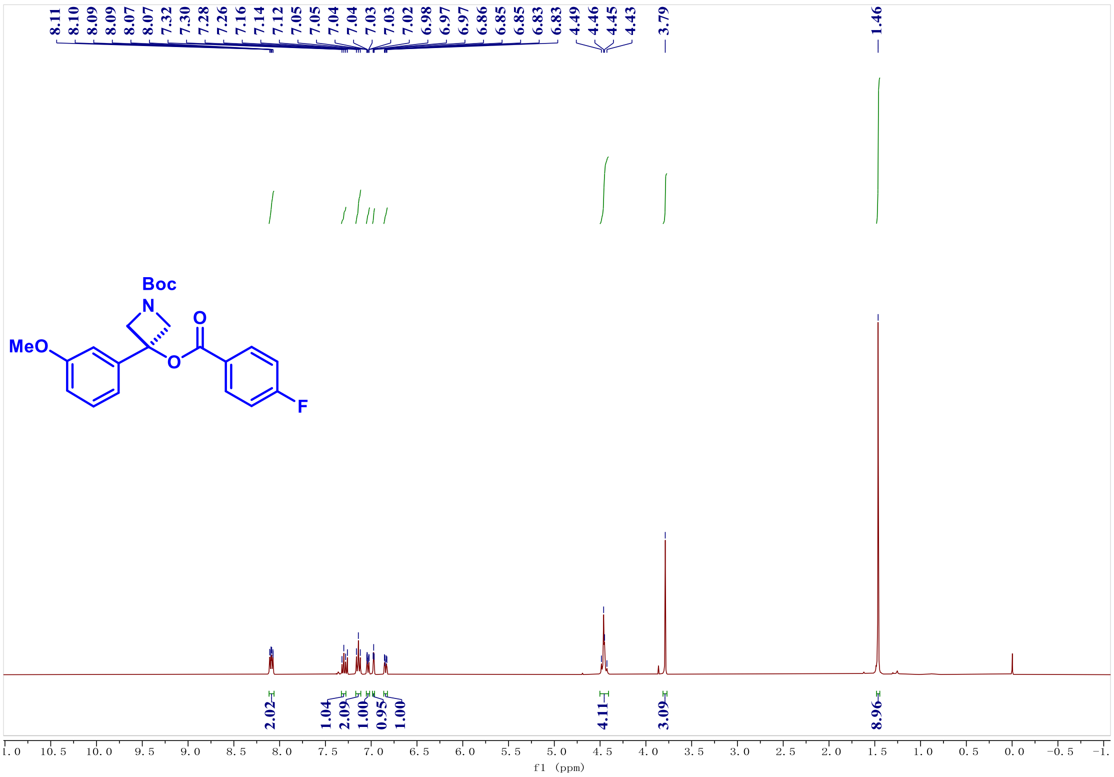

^{13}C NMR of Compound S11 (101 MHz, $CDCl_3$):

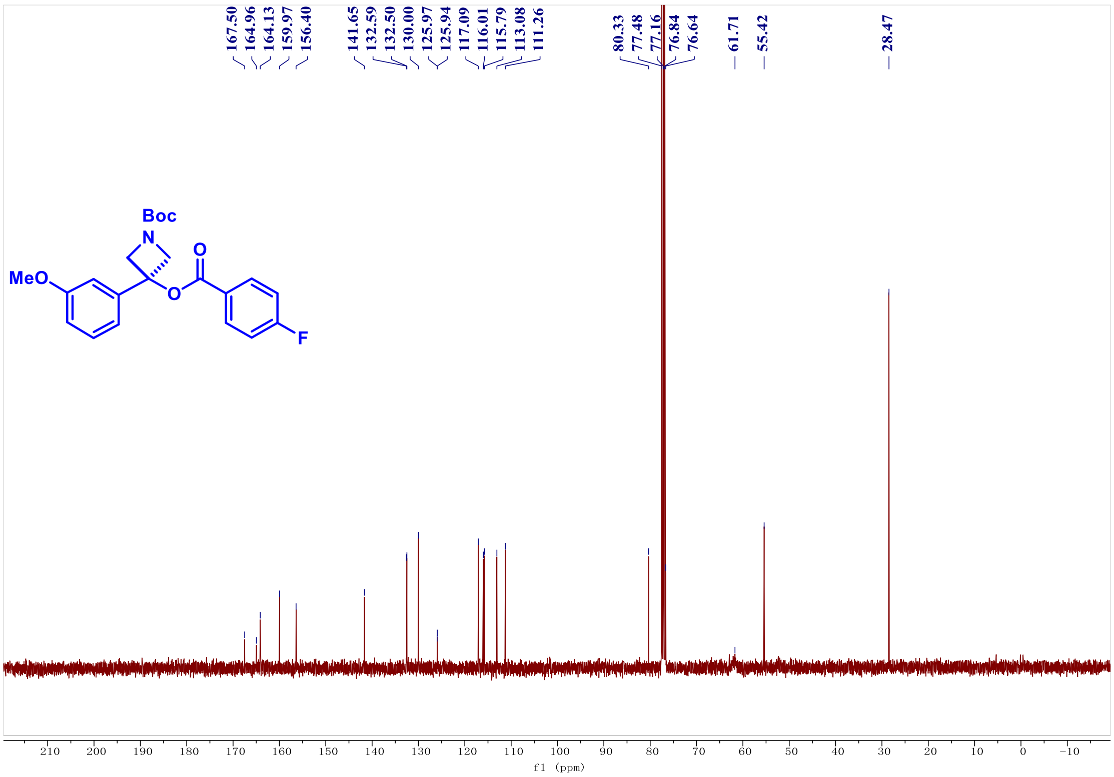

^{19}F NMR of Compound S11 (376 MHz, $CDCl_3$):

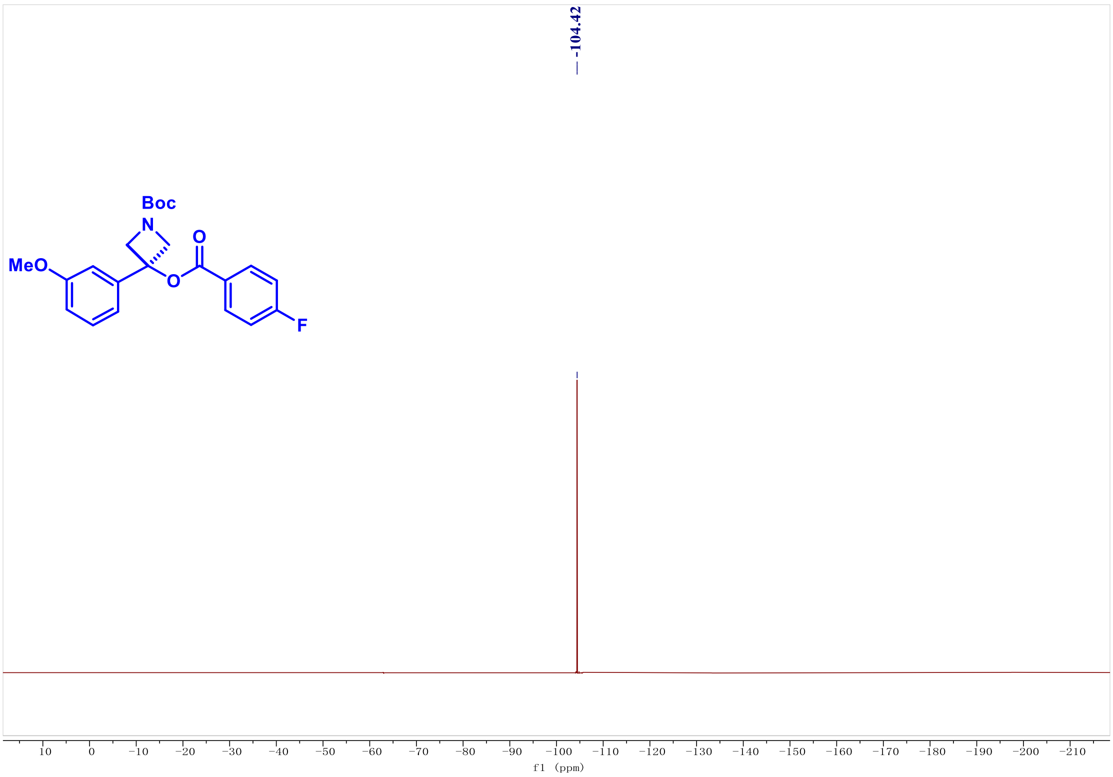

^{1}H NMR of Compound S12 (400 MHz, $CDCl_3$):

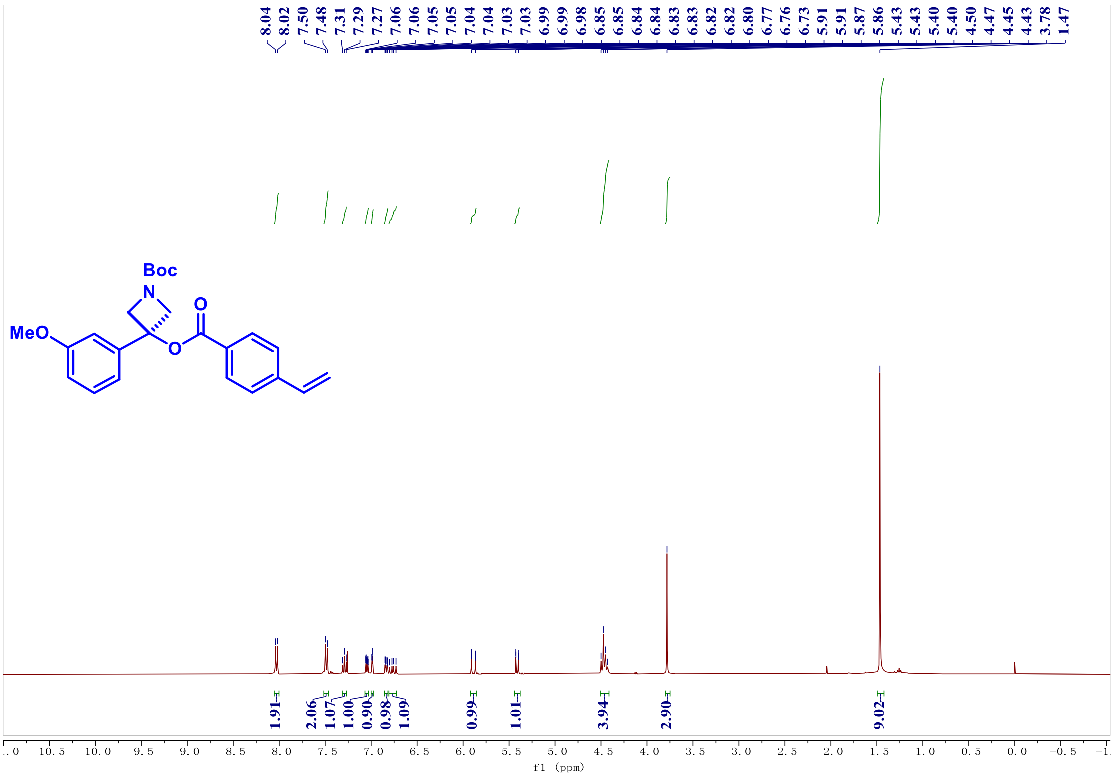

^{13}C NMR of Compound S12 (101 MHz, $CDCl_3$):

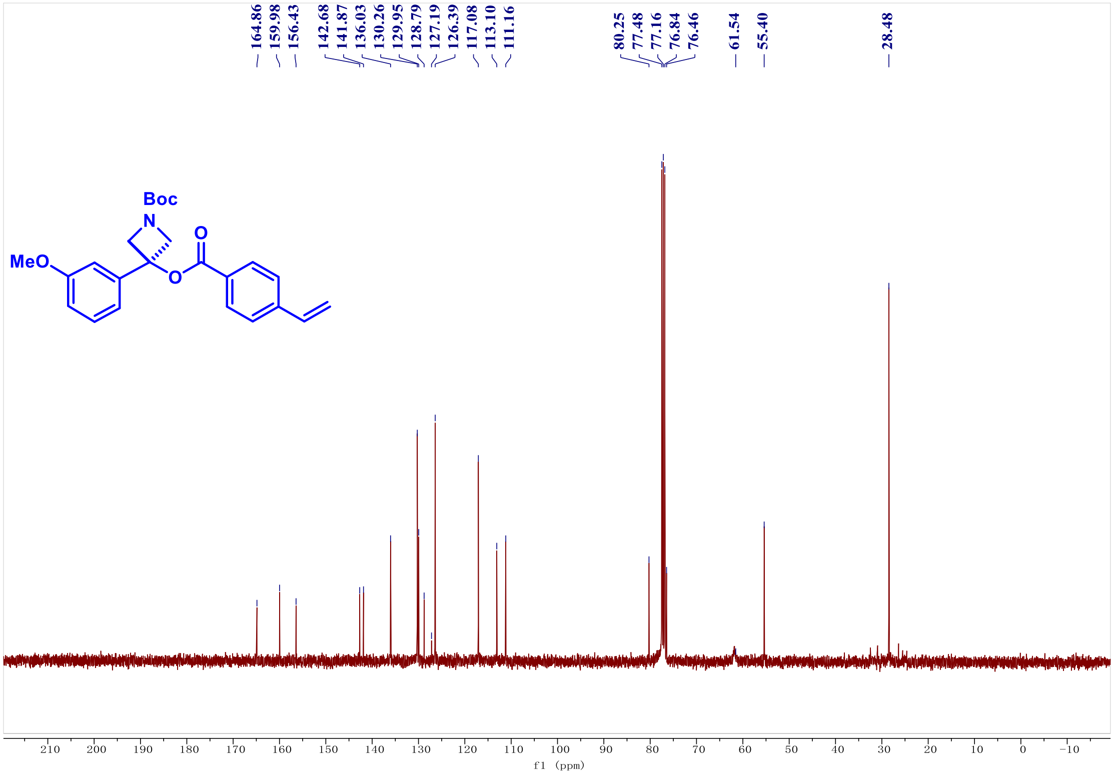

^{1}H NMR of Compound S13 (400 MHz, $CDCl_3$):

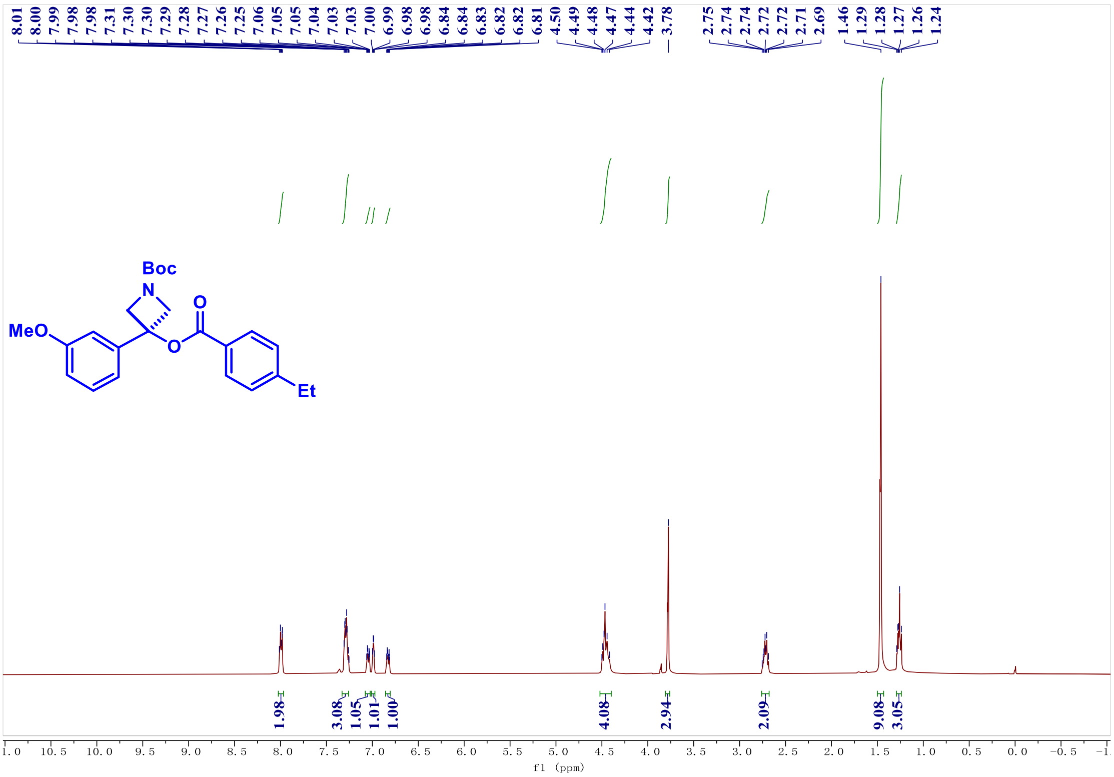

^{13}C NMR of Compound S13 (101 MHz, $CDCl_3$):

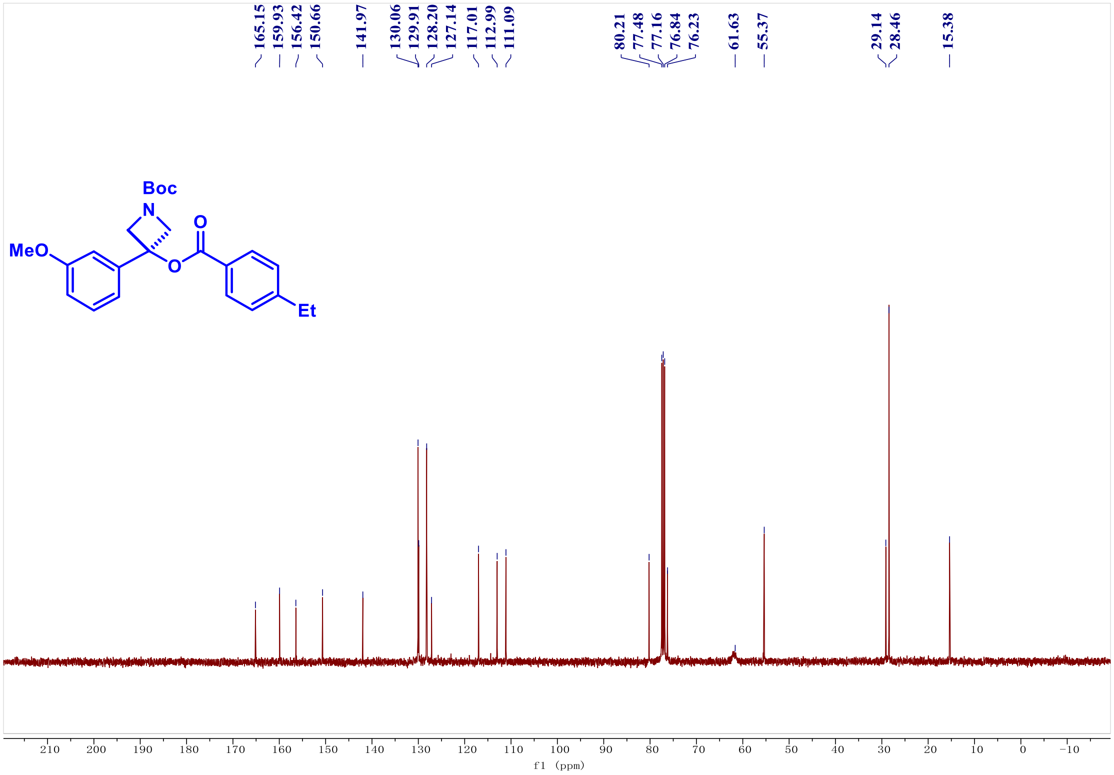

^{1}H NMR of Compound S14 (400 MHz, $CDCl_3$):

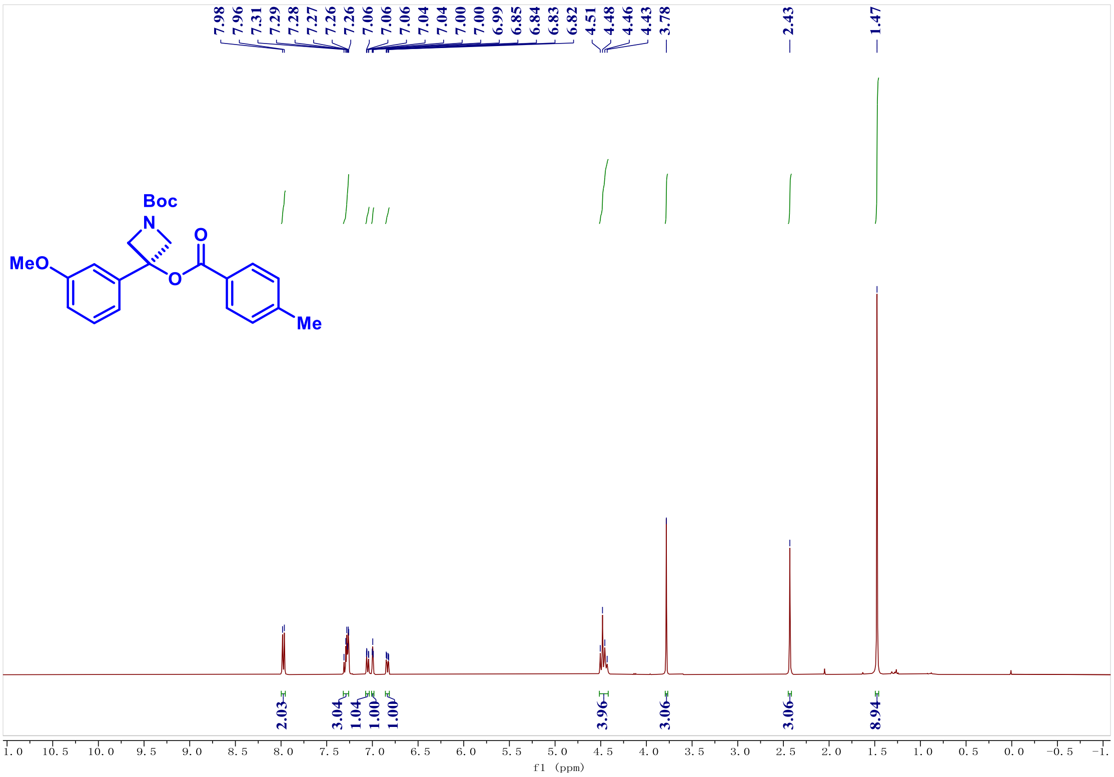

^{13}C NMR of Compound S14 (101 MHz, $CDCl_3$):

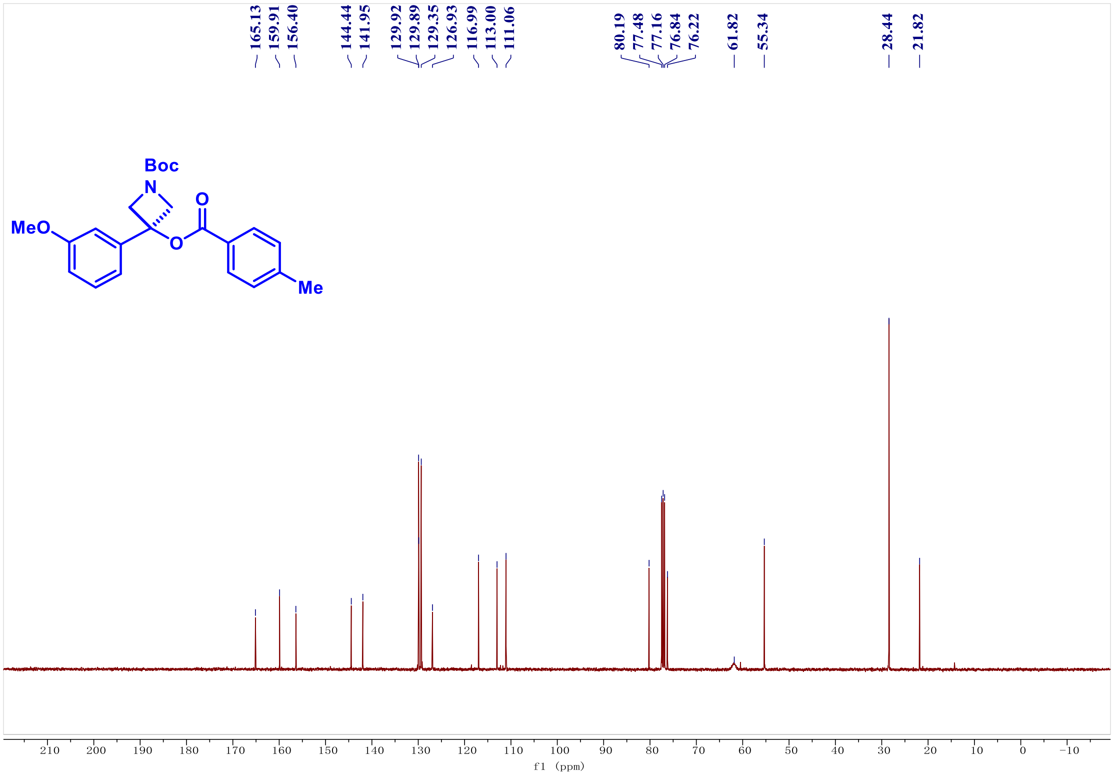

^{1}H NMR of Compound S15 (500 MHz, $CDCl_3$):

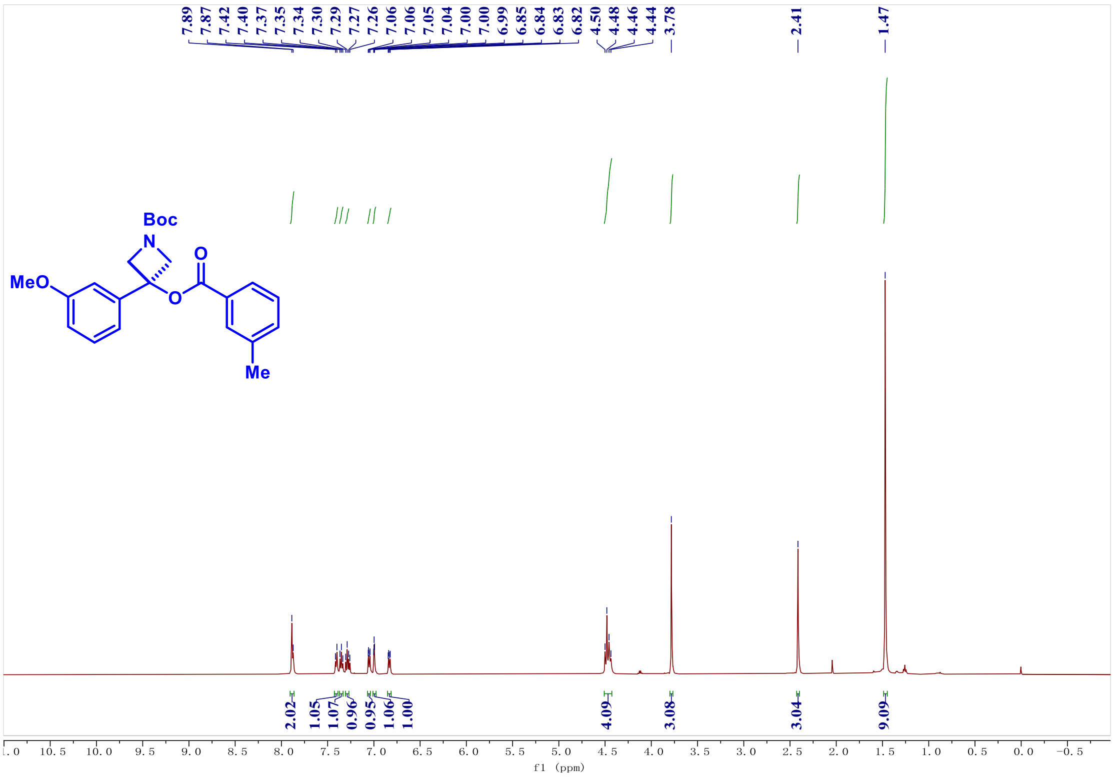

^{13}C NMR of Compound S15 (101 MHz, $CDCl_3$):

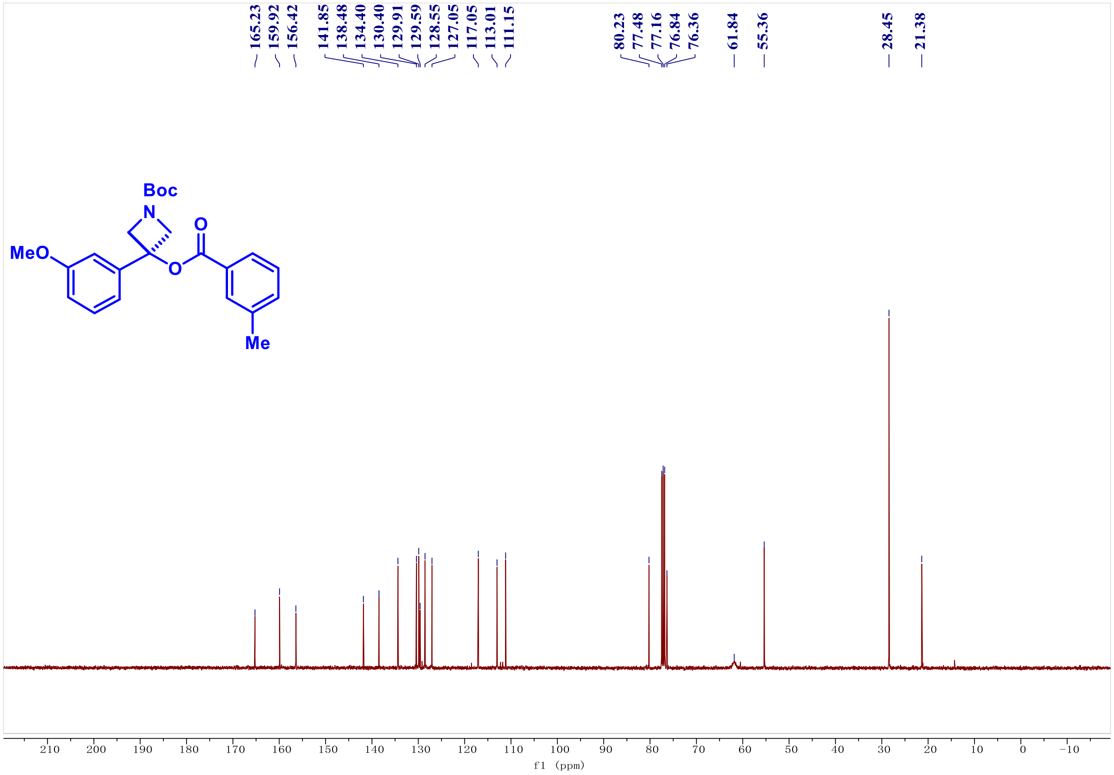

^{1}H NMR of Compound S16 (400 MHz, $CDCl_3$):

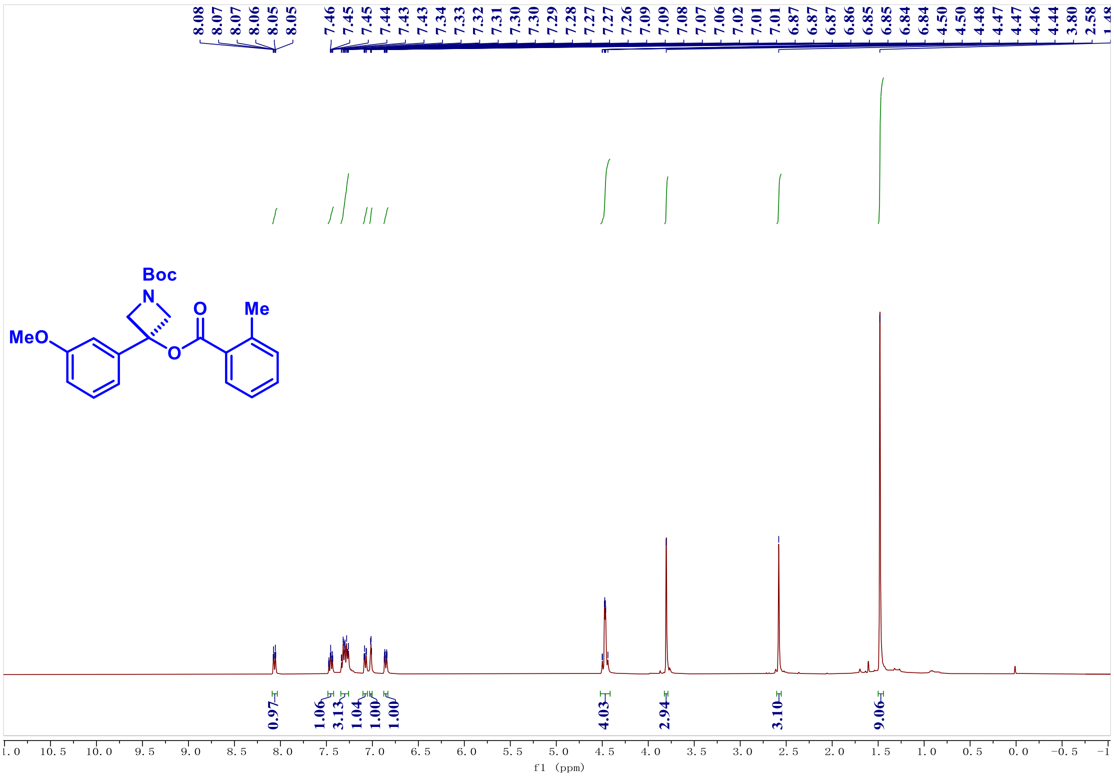

^{13}C NMR of Compound S16 (101 MHz, $CDCl_3$):

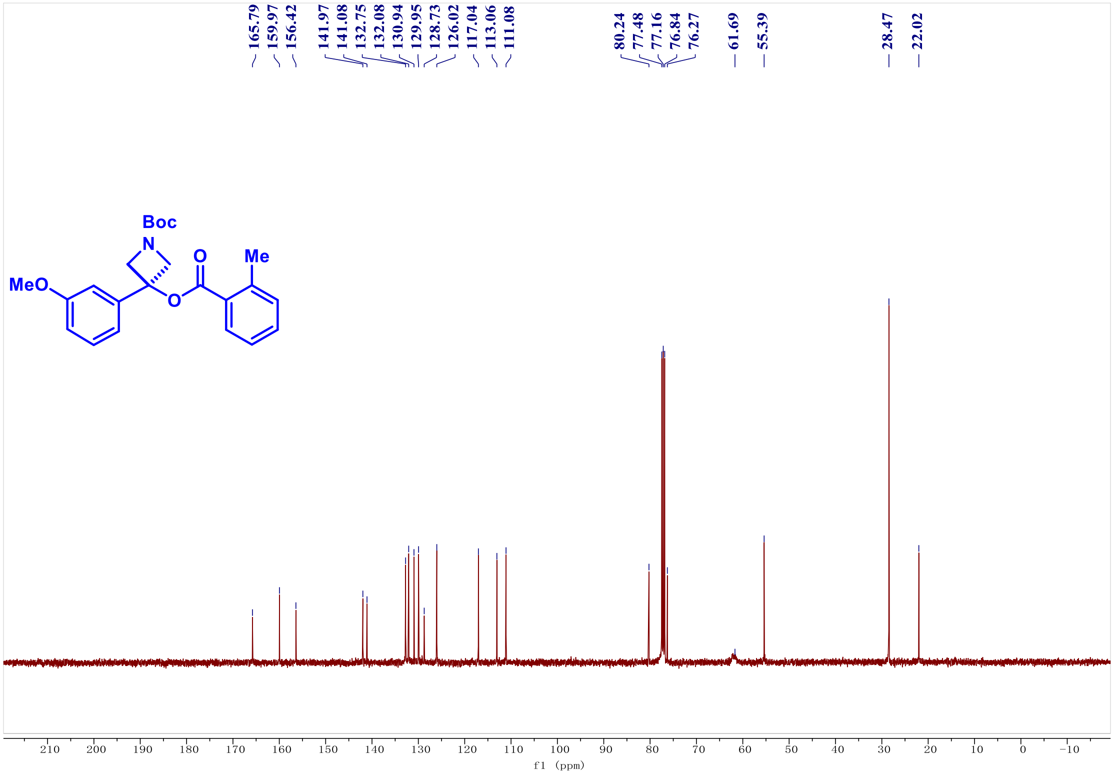

^{1}H NMR of Compound S17 (400 MHz, $CDCl_3$):

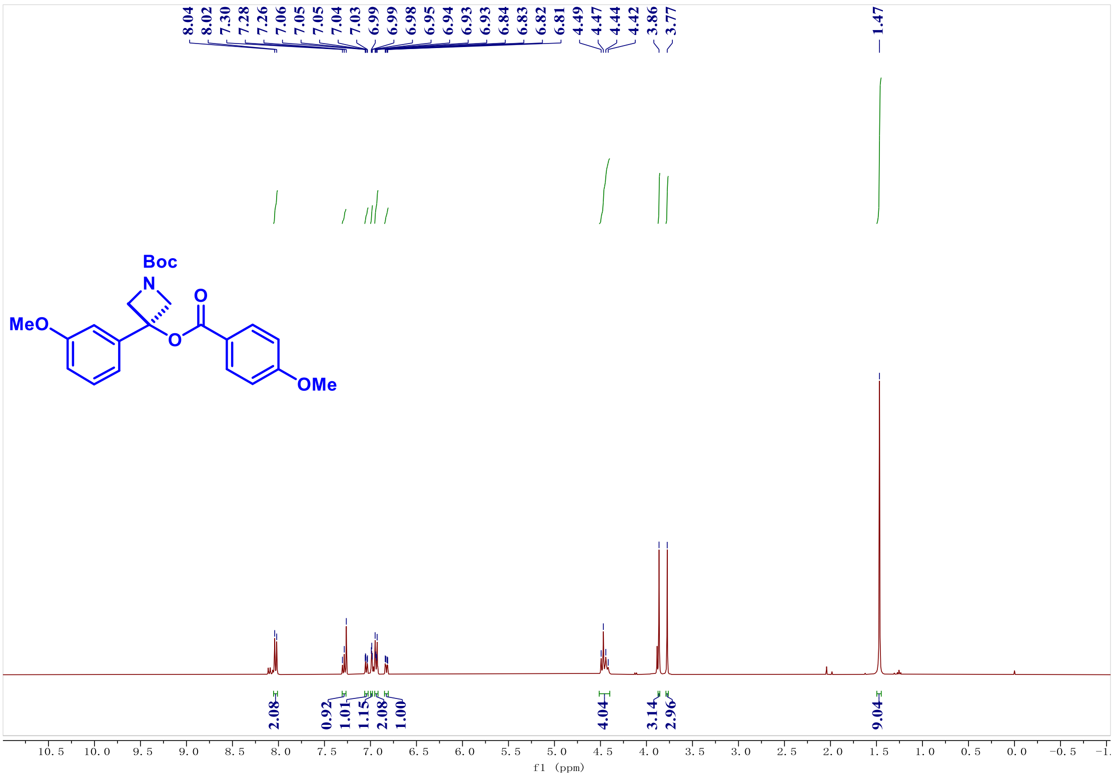

^{13}C NMR of Compound S17 (101 MHz, $CDCl_3$):

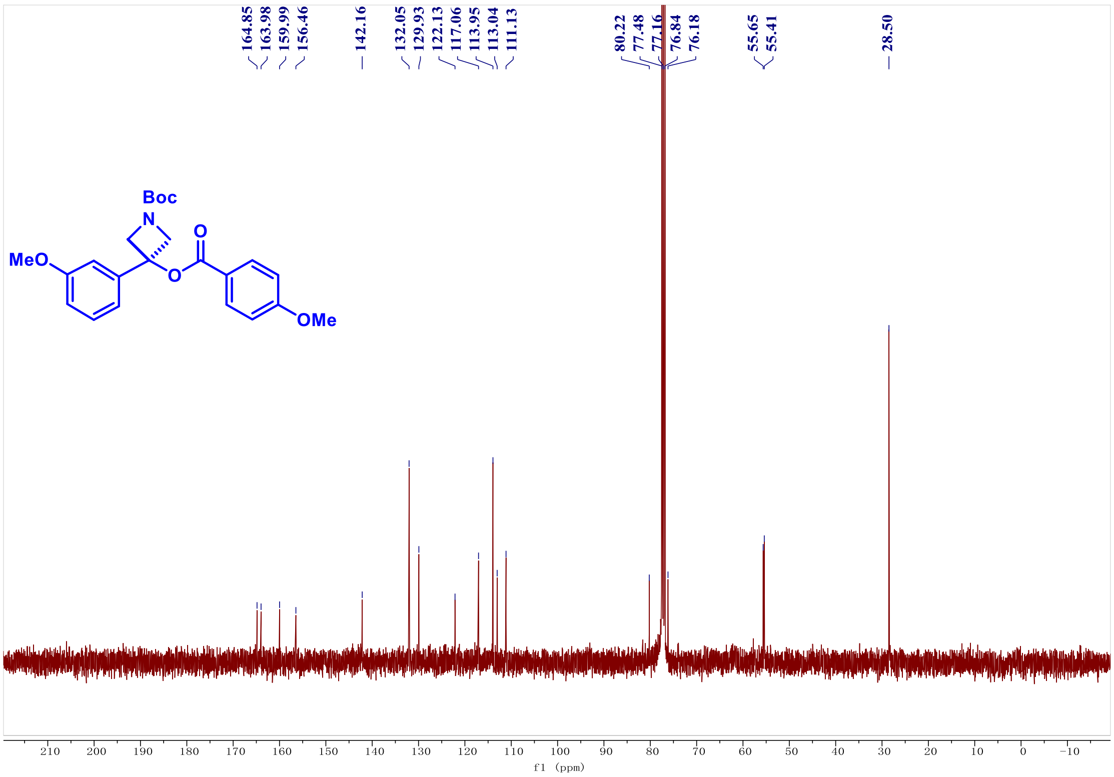

^{1}H NMR of Compound S18 (400 MHz, $CDCl_3$):

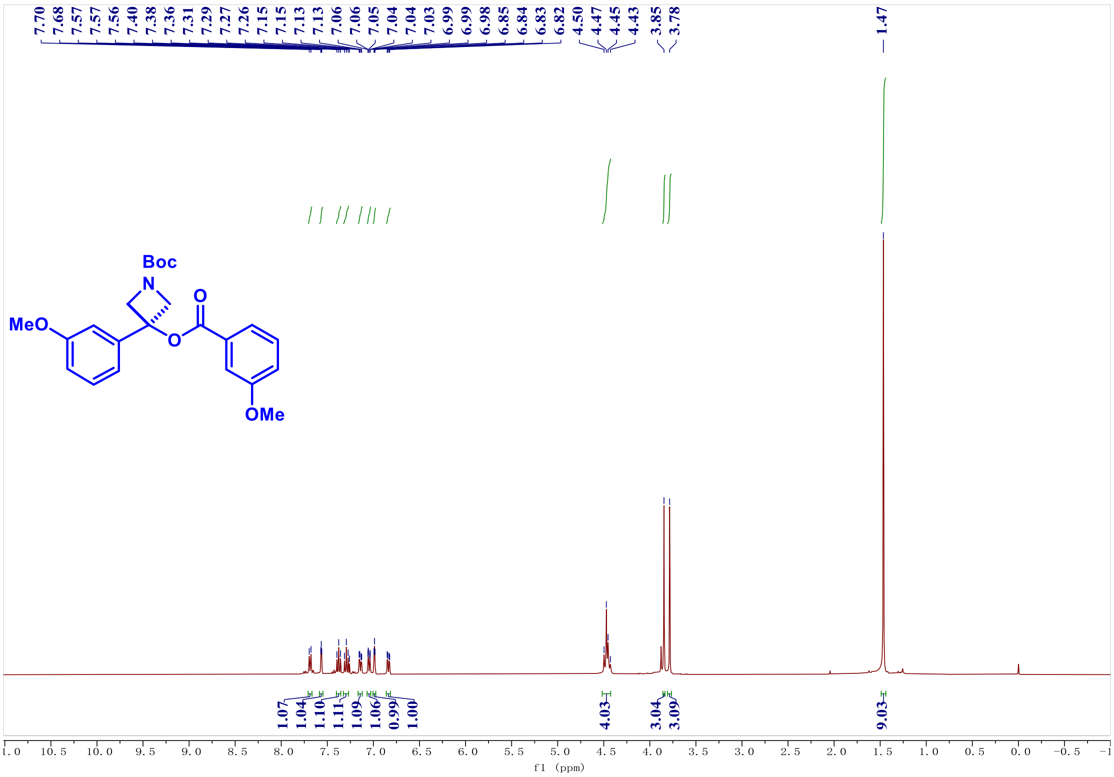

^{13}C NMR of Compound S18 (101 MHz, $CDCl_3$):

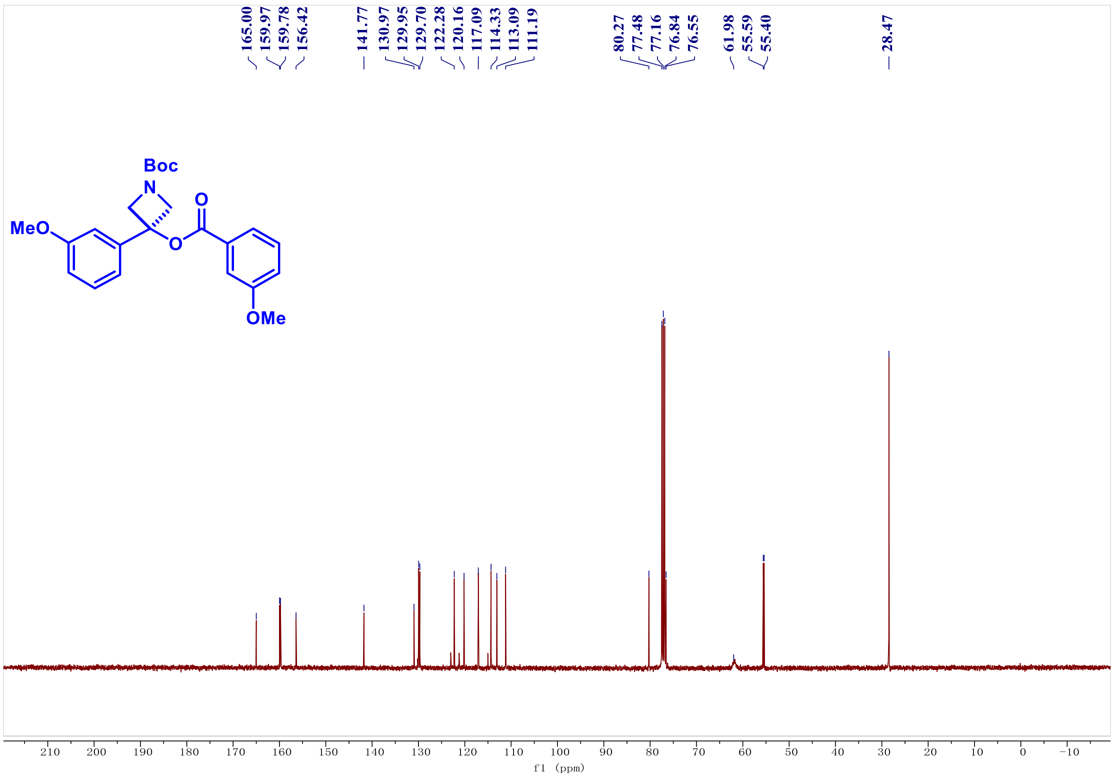

^{1}H NMR of Compound S19 (400 MHz, $CDCl_3$):

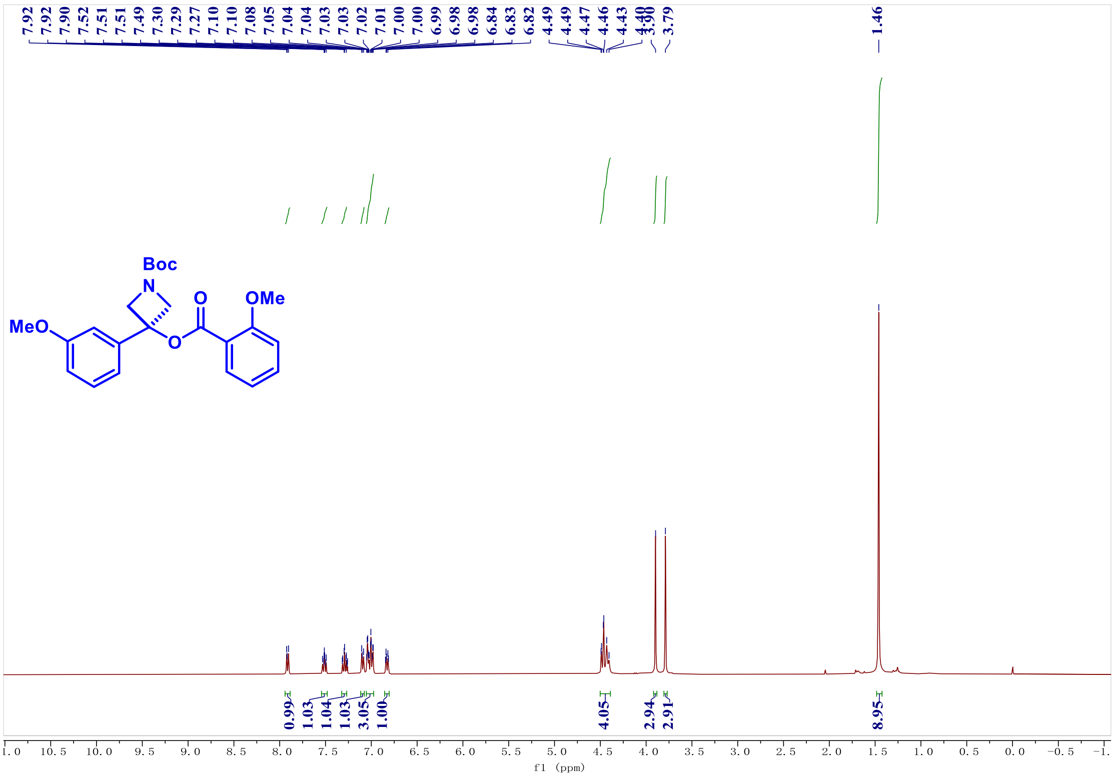

^{13}C NMR of Compound S19 (101 MHz, $CDCl_3$):

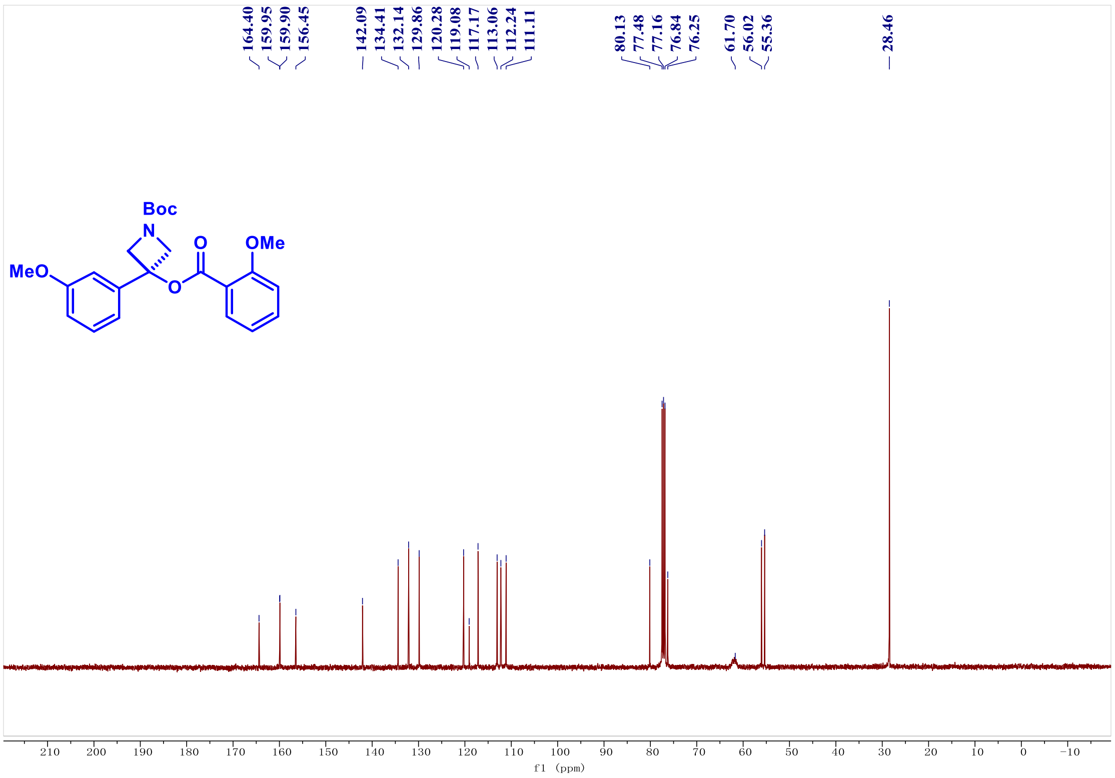

^{1}H NMR of Compound S20 (400 MHz, $CDCl_3$):

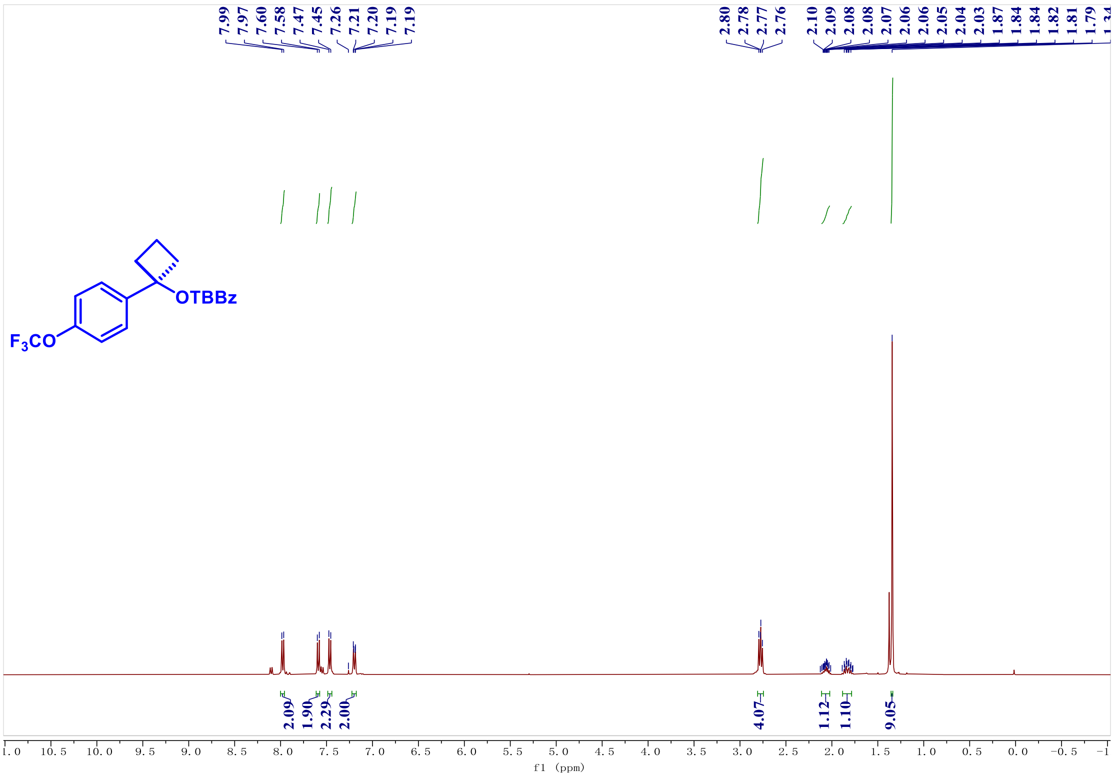

^{13}C NMR of Compound S20 (101 MHz, $CDCl_3$):

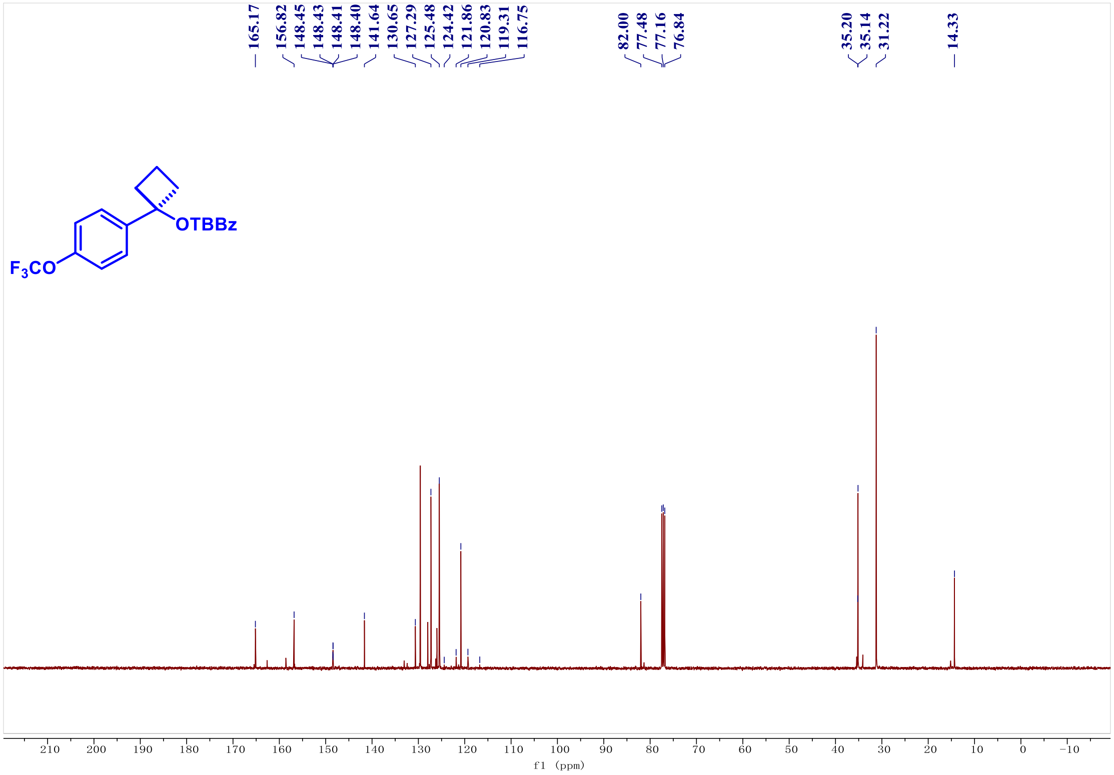

^{19}F NMR of Compound S20 (376 MHz, $CDCl_3$):

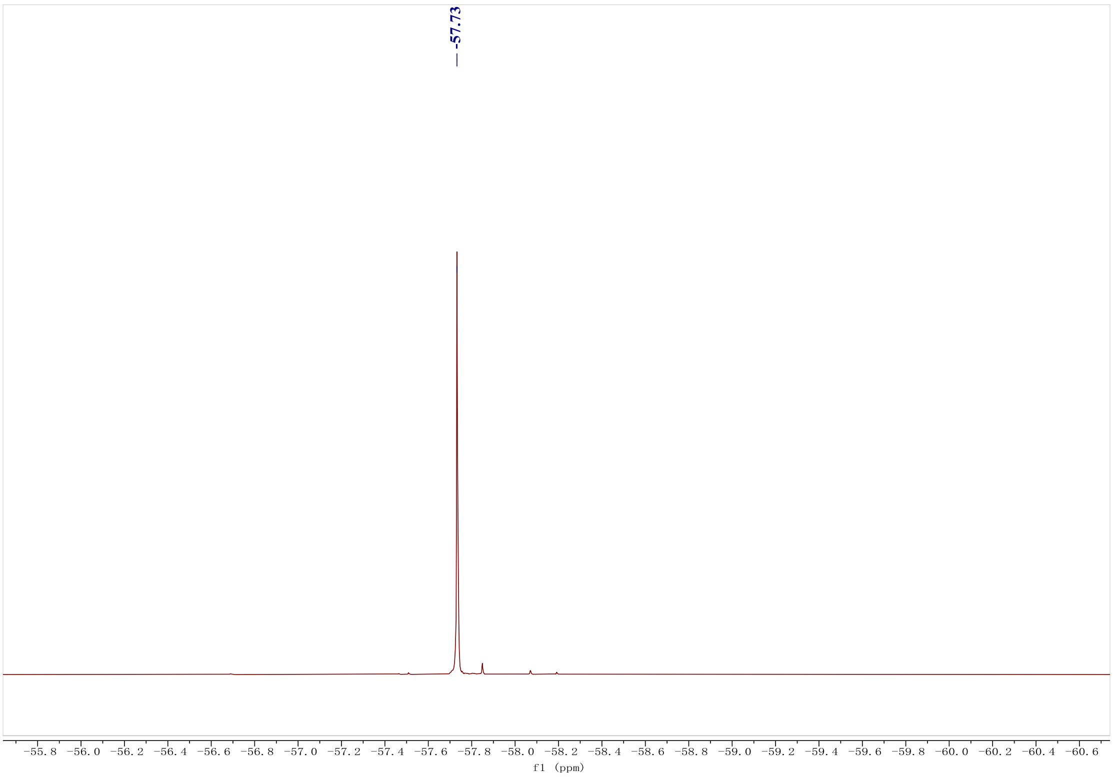

^{1}H NMR of Compound S21 (400 MHz, $CDCl_3$):

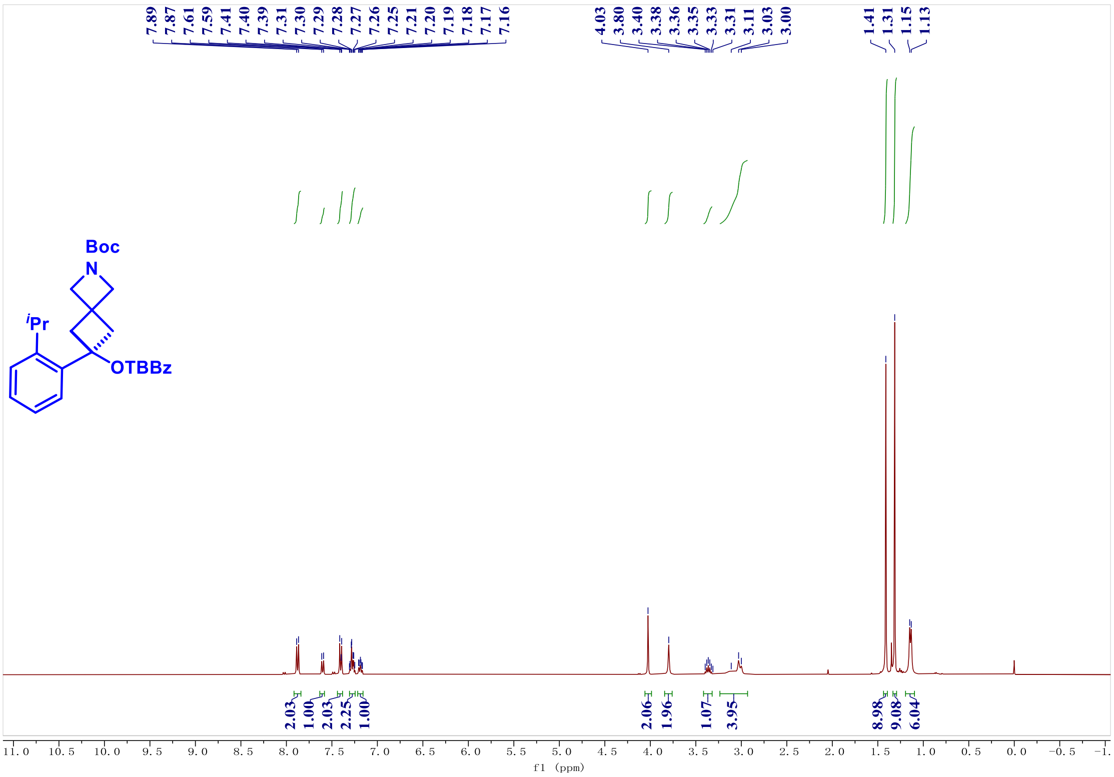

^{13}C NMR of Compound S21 (101 MHz, $CDCl_3$):

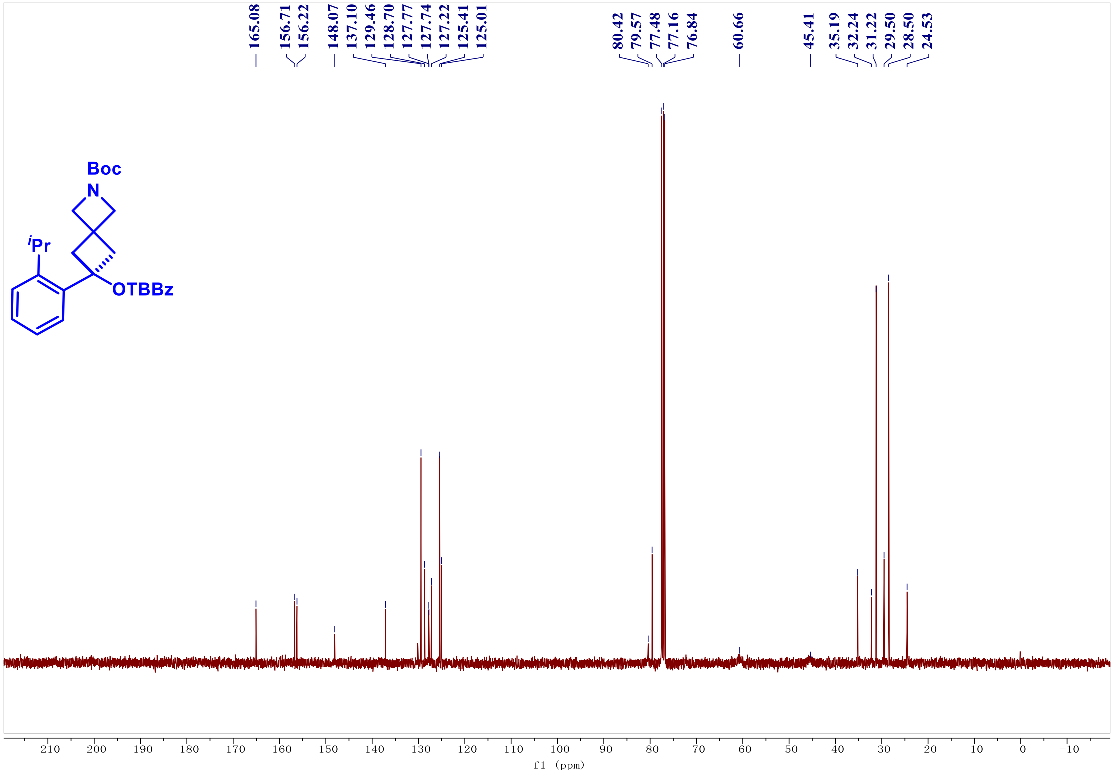

^{1}H NMR of Compound S22 (400 MHz, $CDCl_3$):

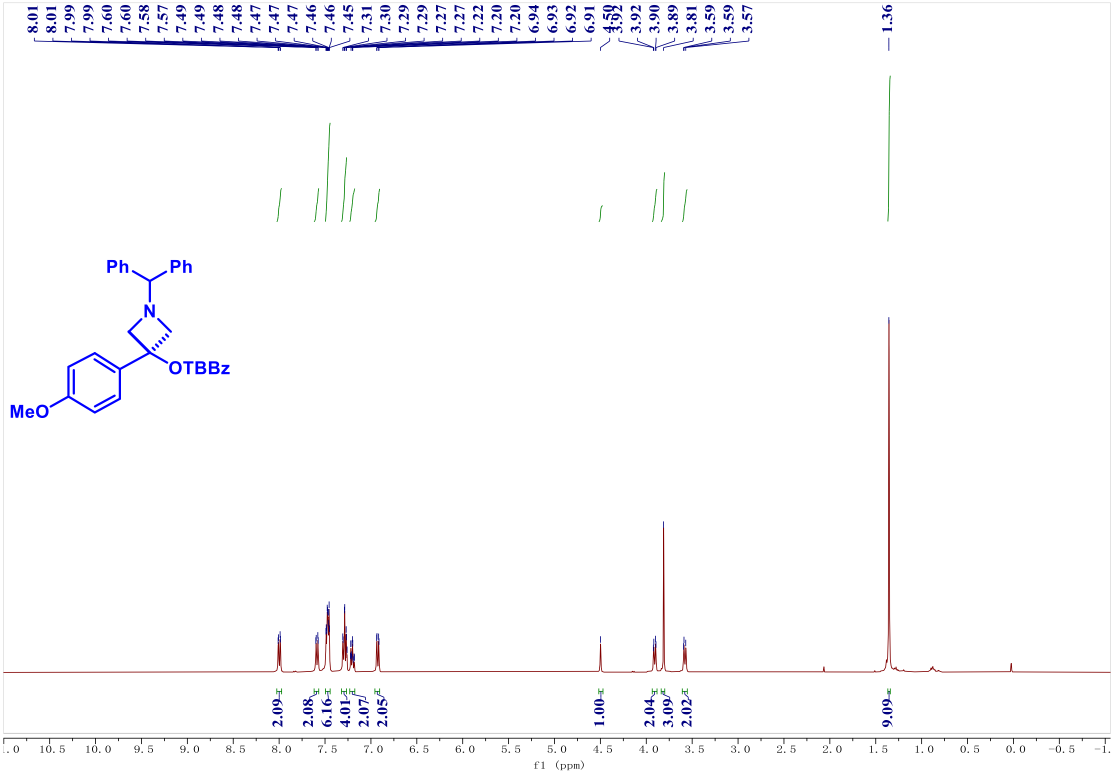

^{13}C NMR of Compound S22 (101 MHz, $CDCl_3$):

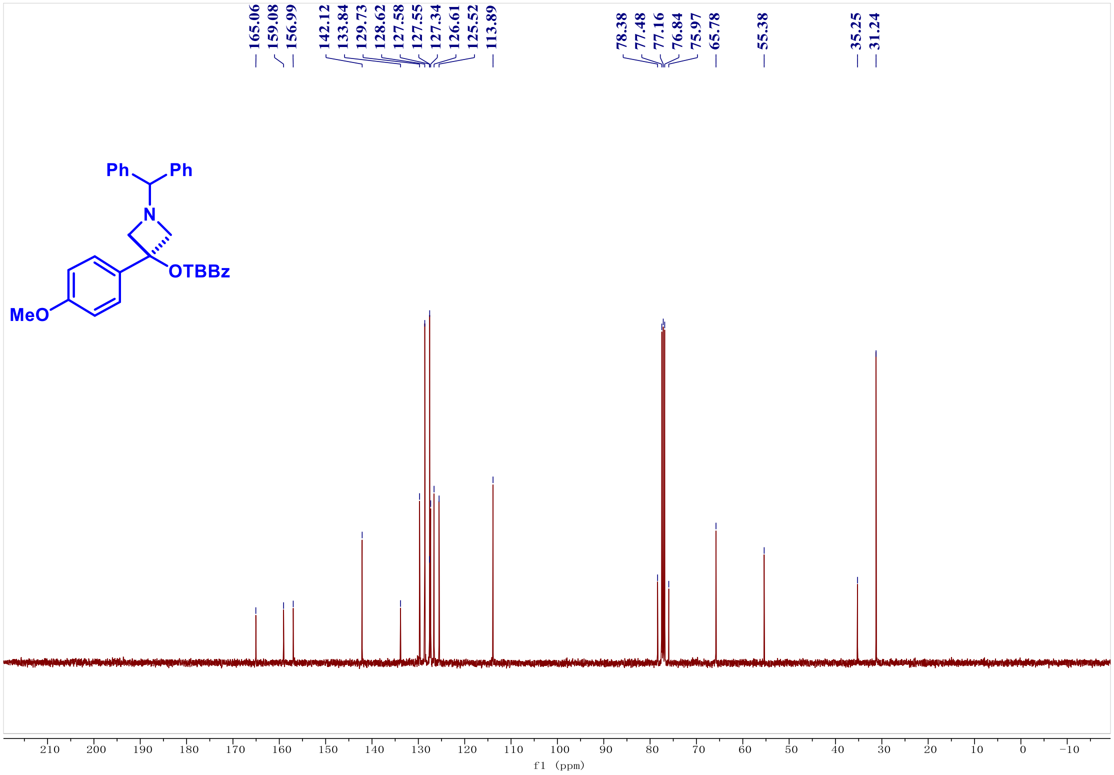

^{1}H NMR of Compound S23 (400 MHz, $CDCl_3$):

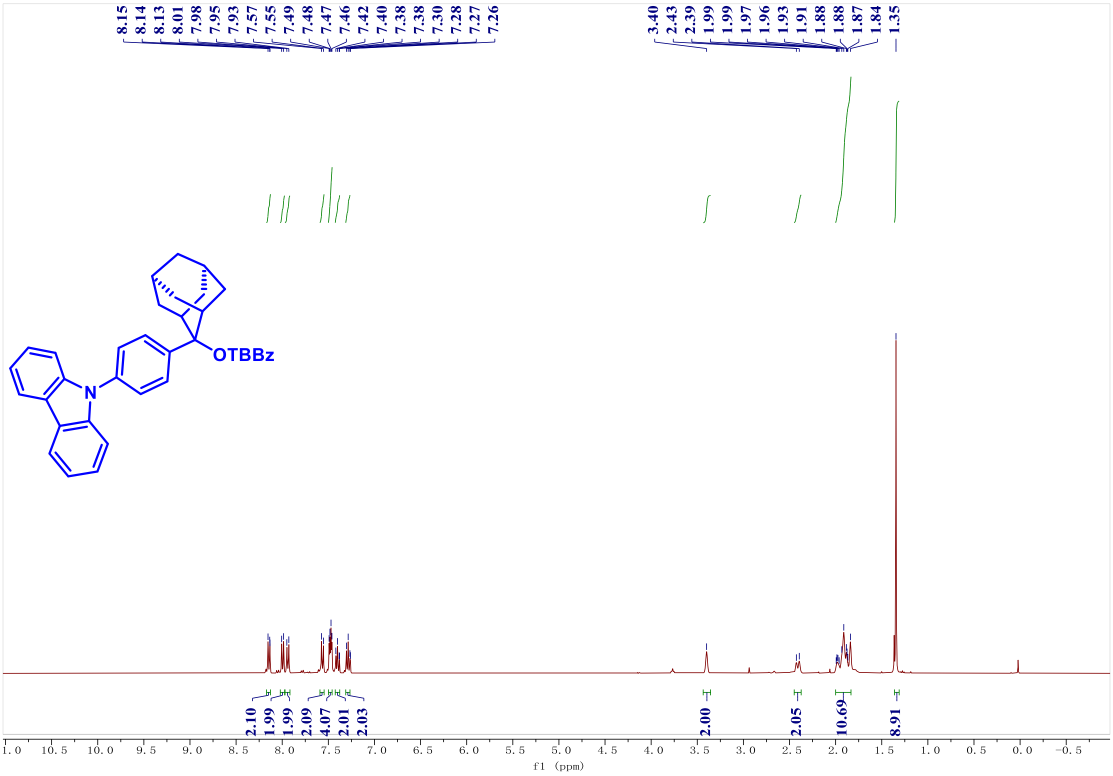

^{13}C NMR of Compound S23 (101 MHz, $CDCl_3$):

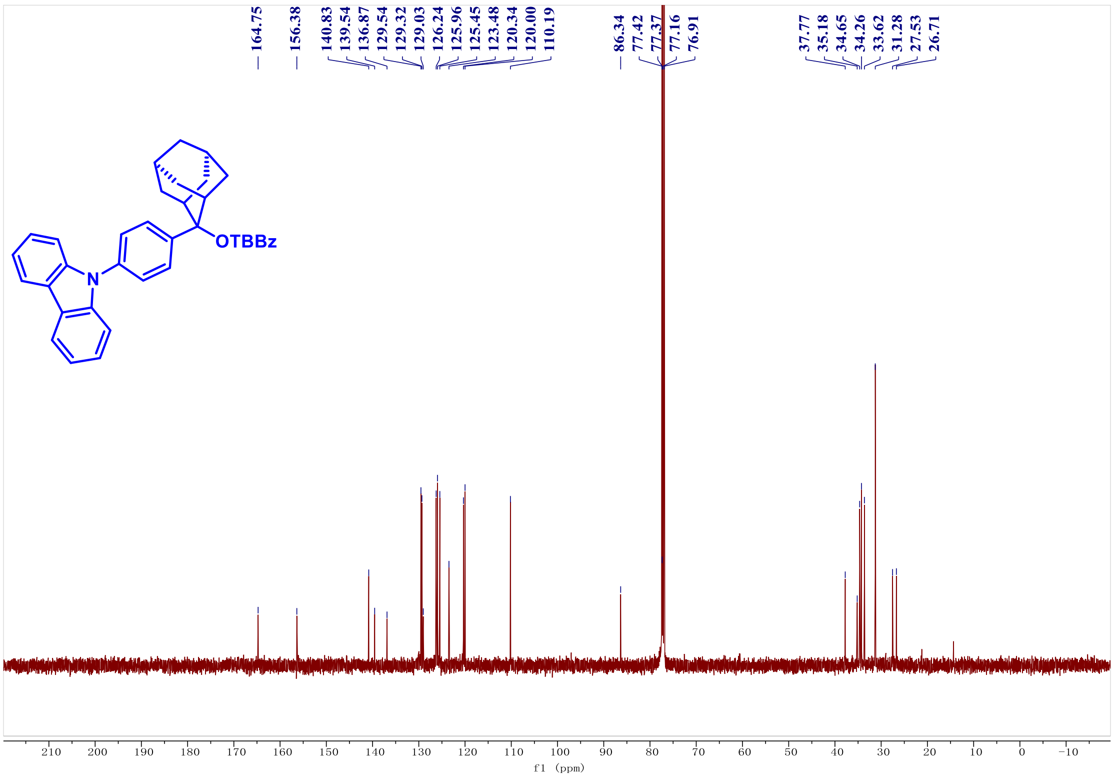

^{1}H NMR of Compound S24 (400 MHz, $CDCl_3$):

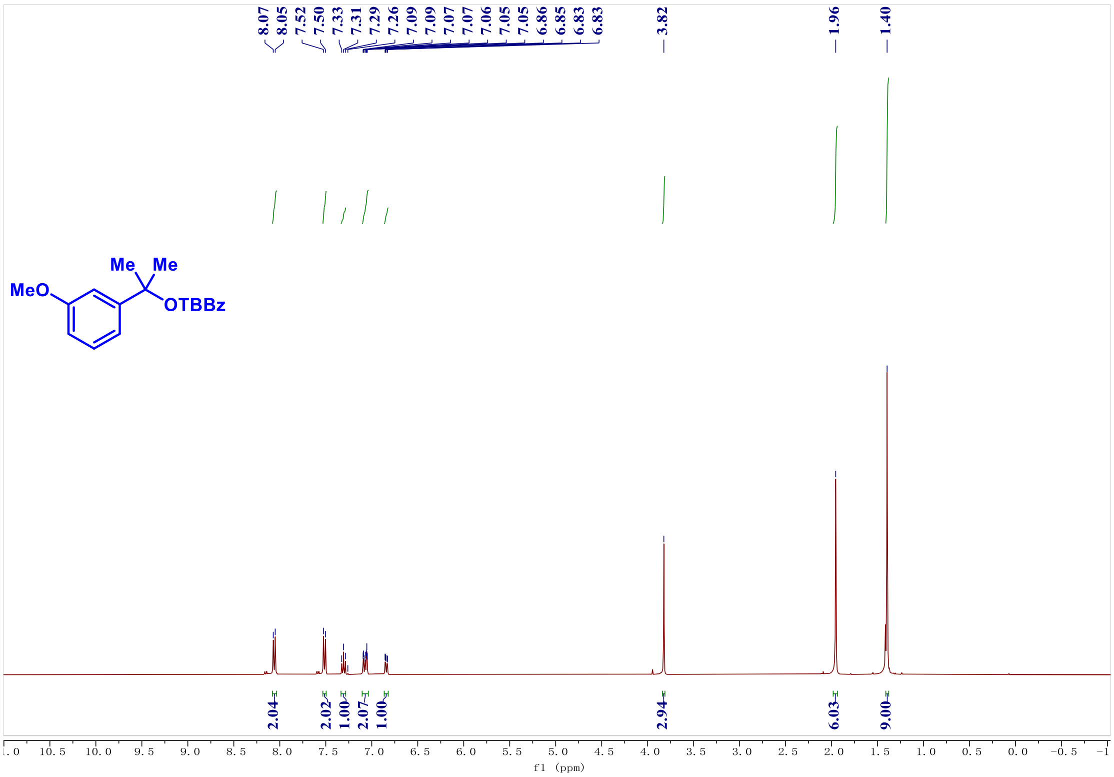

^{13}C NMR of Compound S24 (101 MHz, $CDCl_3$):

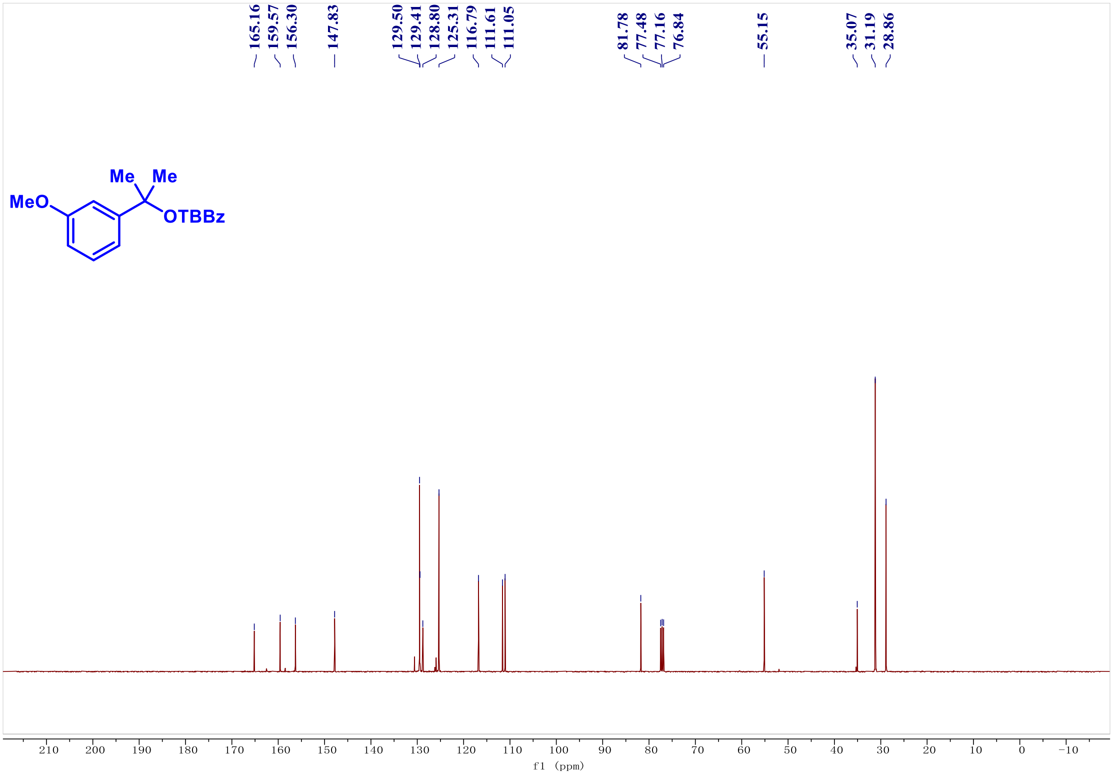

1H NMR of Compound S25 (400 MHz, $CDCl_3$):

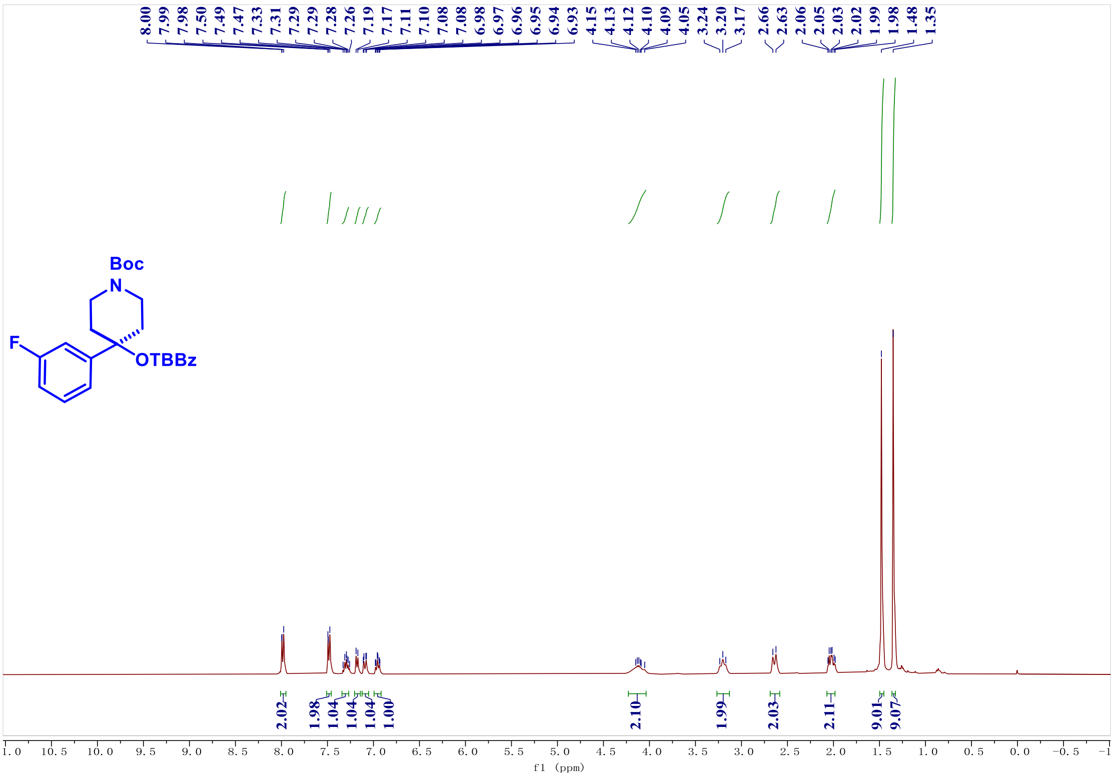

13C NMR of Compound S25 (101 MHz, $CDCl_3$):

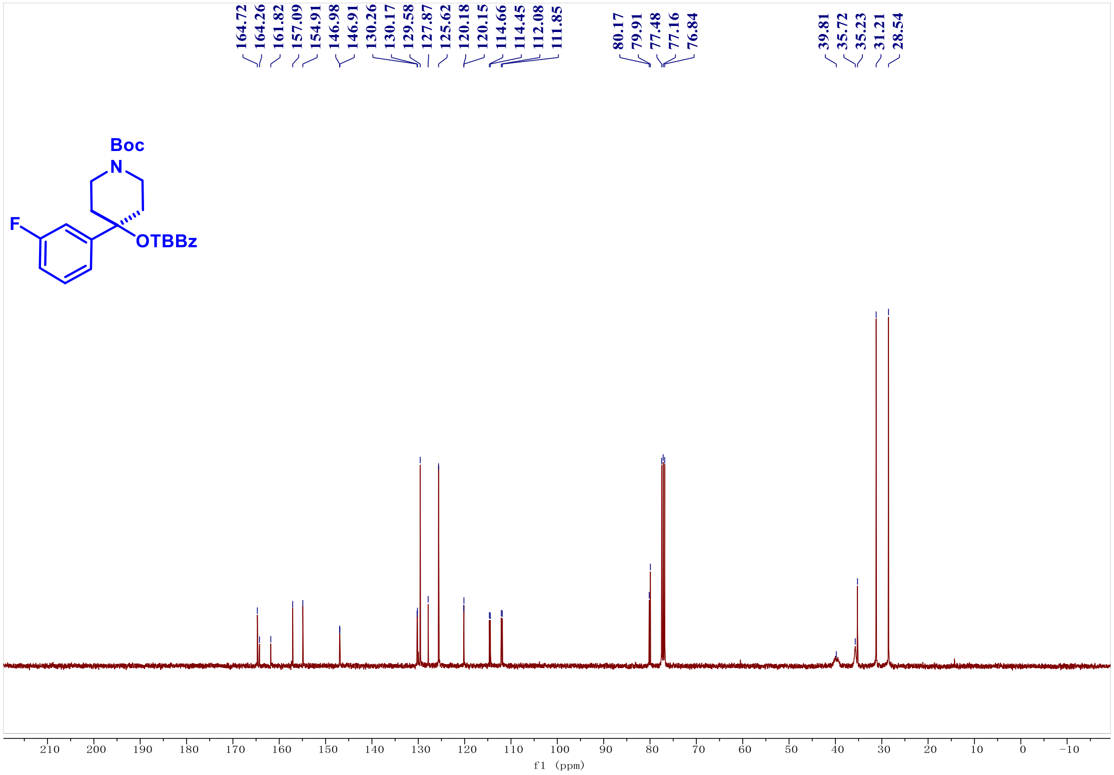

^{19}F NMR of Compound S25 (376 MHz, $CDCl_3$):

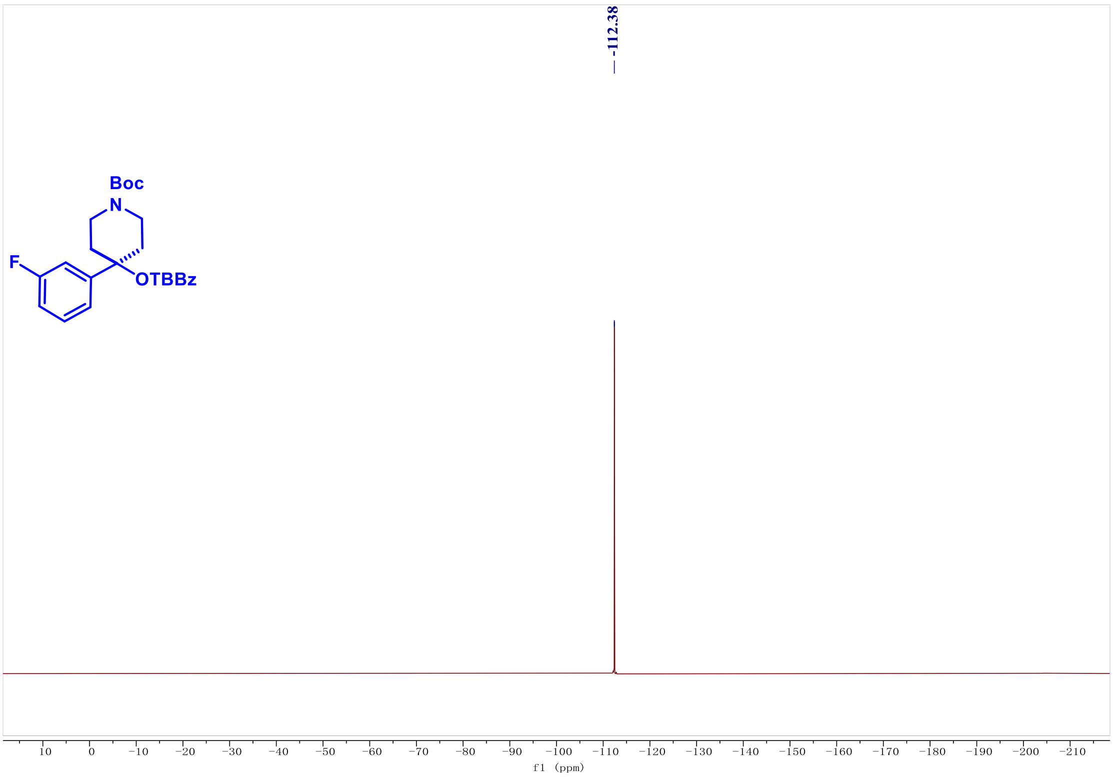

^{1}H NMR of Compound S26 (400 MHz, $CDCl_3$):

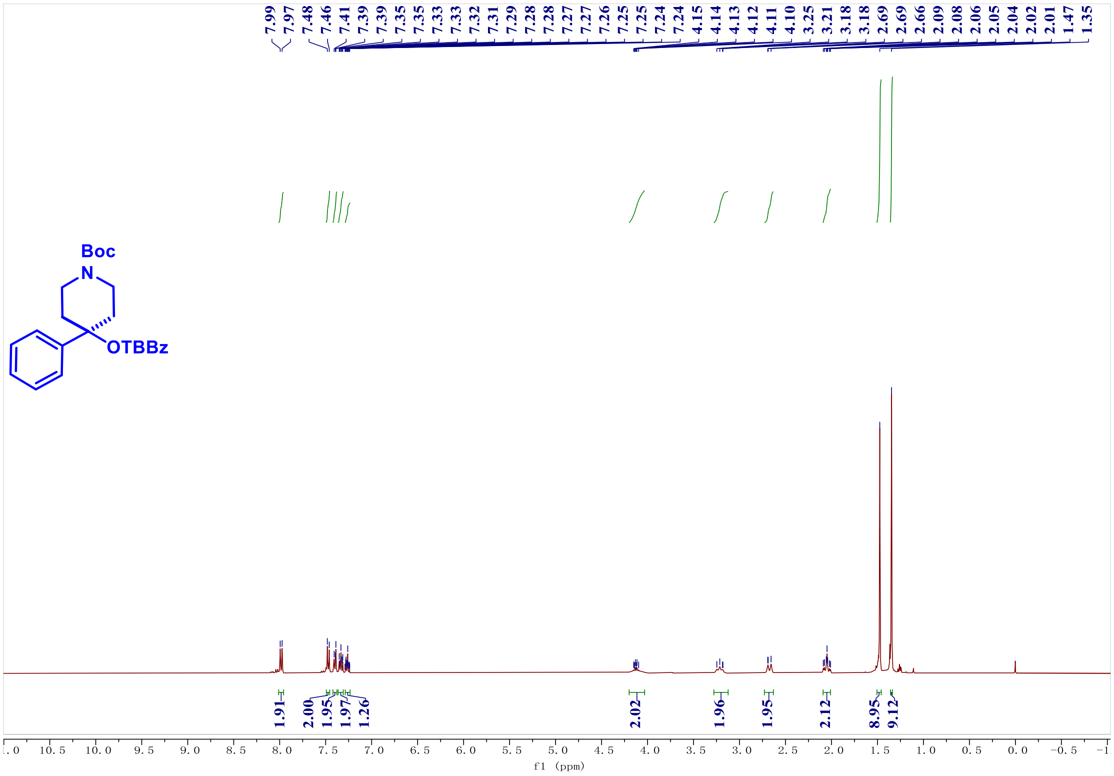

^{13}C NMR of Compound S26 (101 MHz, $CDCl_3$):

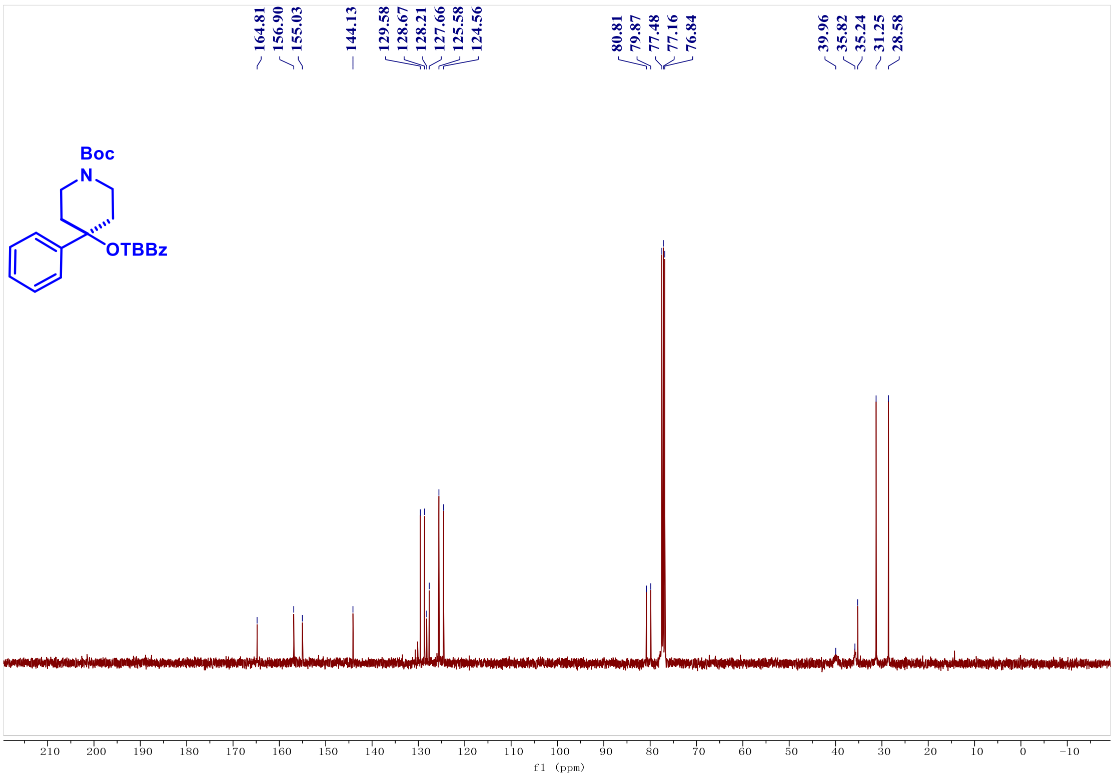

^{1}H NMR of Compound S27 (400 MHz, $CDCl_3$):

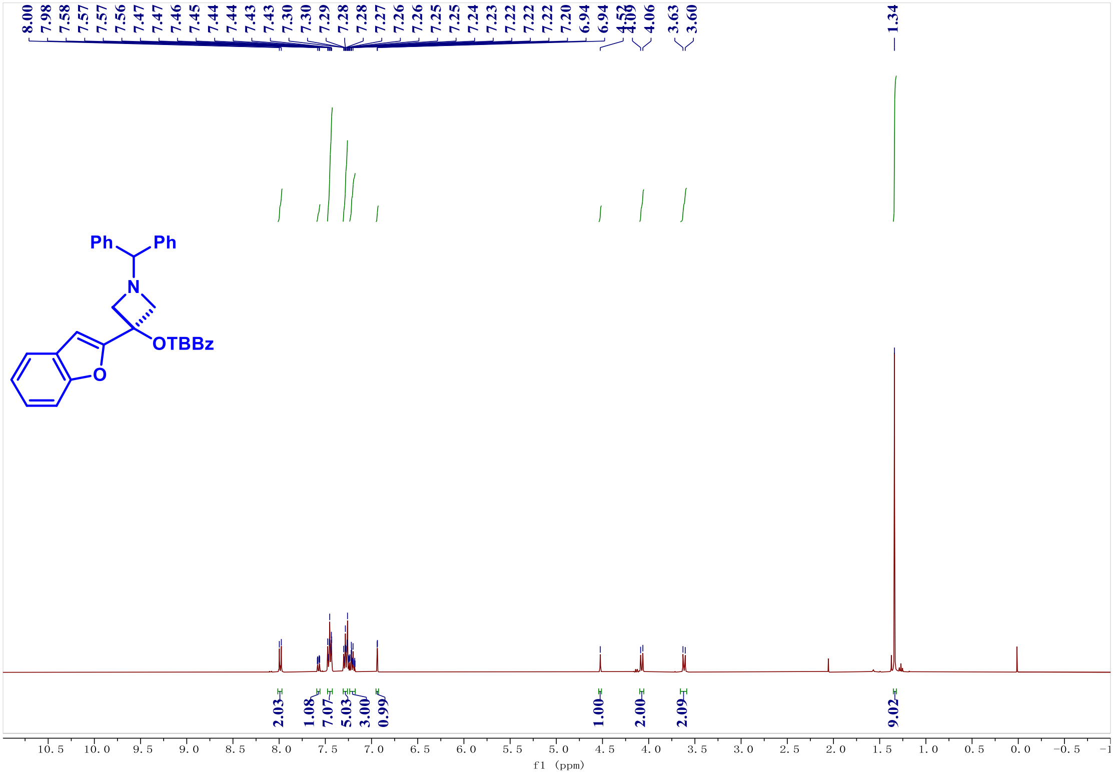

^{13}C NMR of Compound S27 (101 MHz, $CDCl_3$):

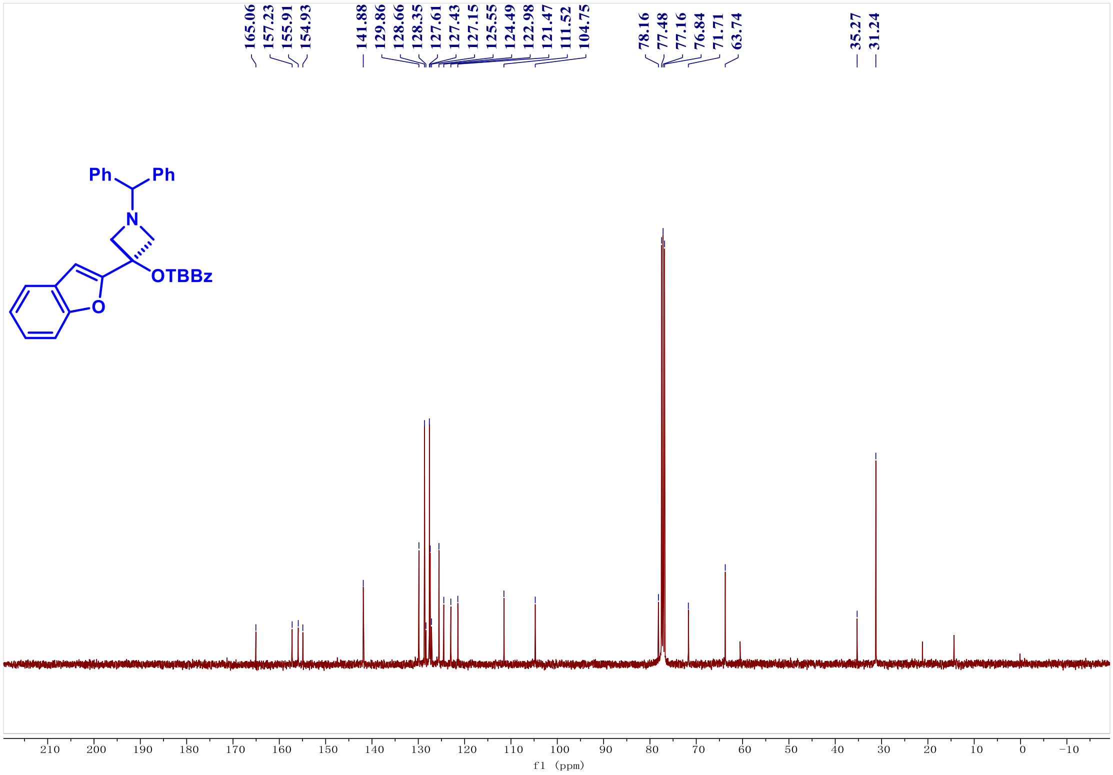

^{1}H NMR of Compound S28 (400 MHz, $CDCl_3$):

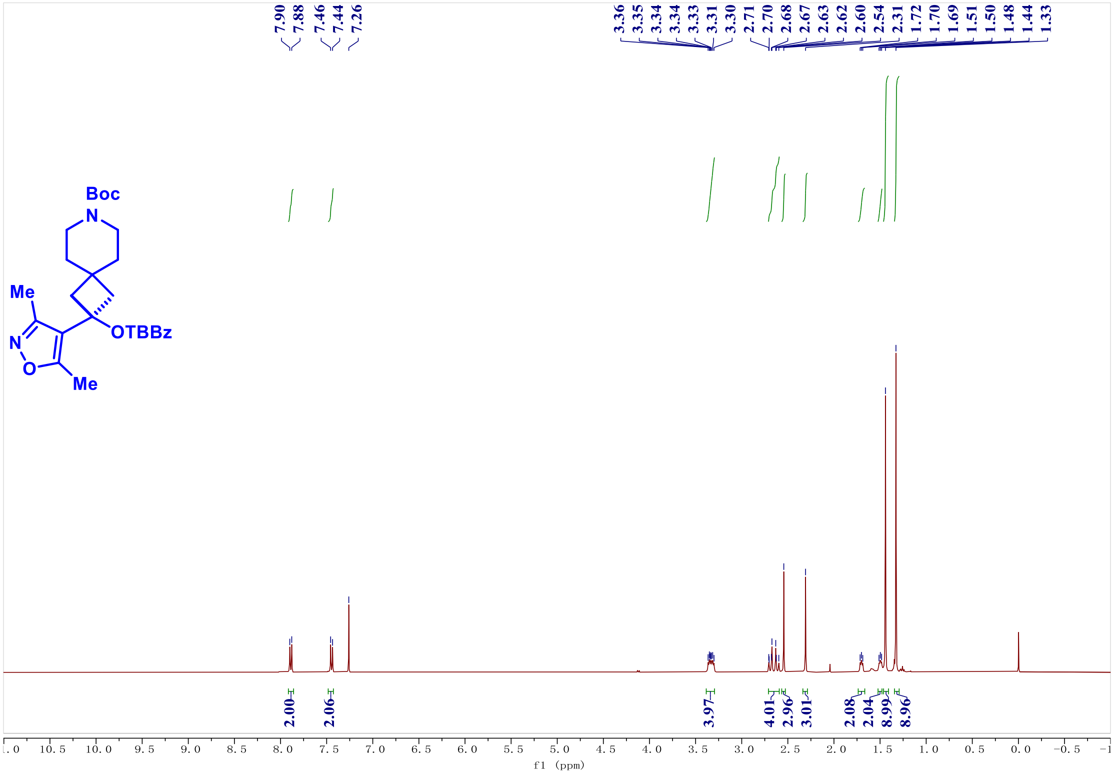

^{13}C NMR of Compound S28 (101 MHz, $CDCl_3$):

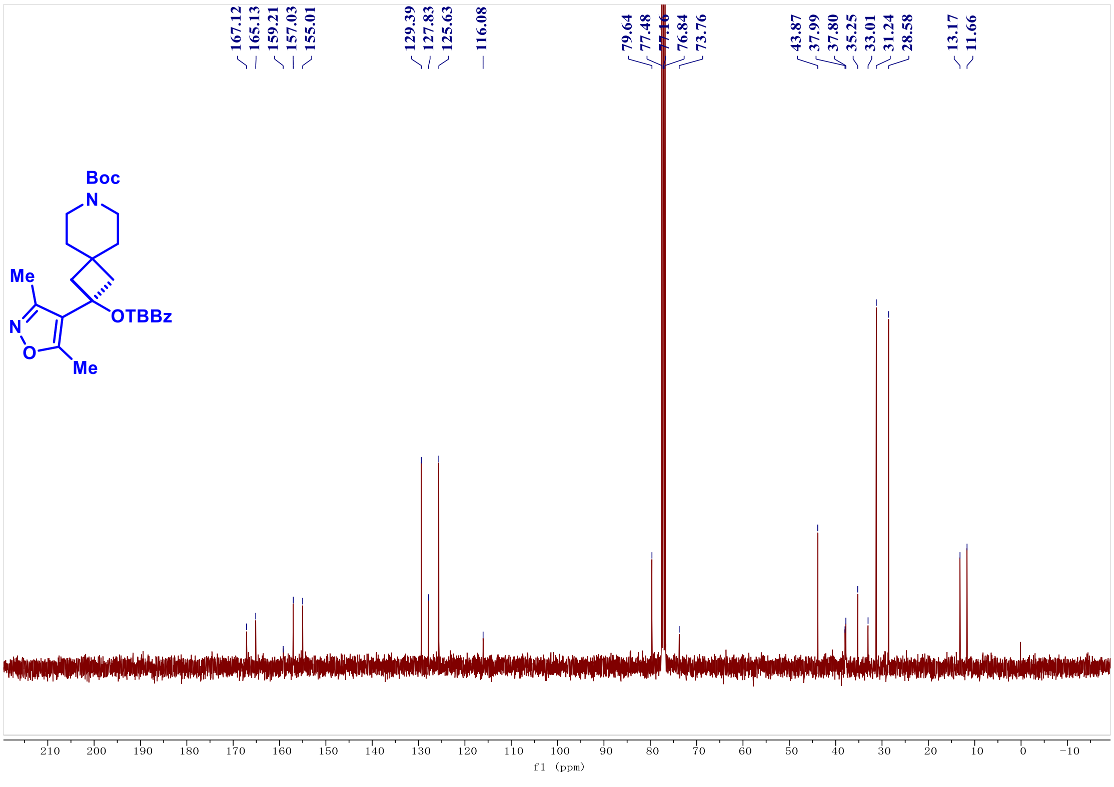

^{1}H NMR of Compound S29 (400 MHz, $CDCl_3$):

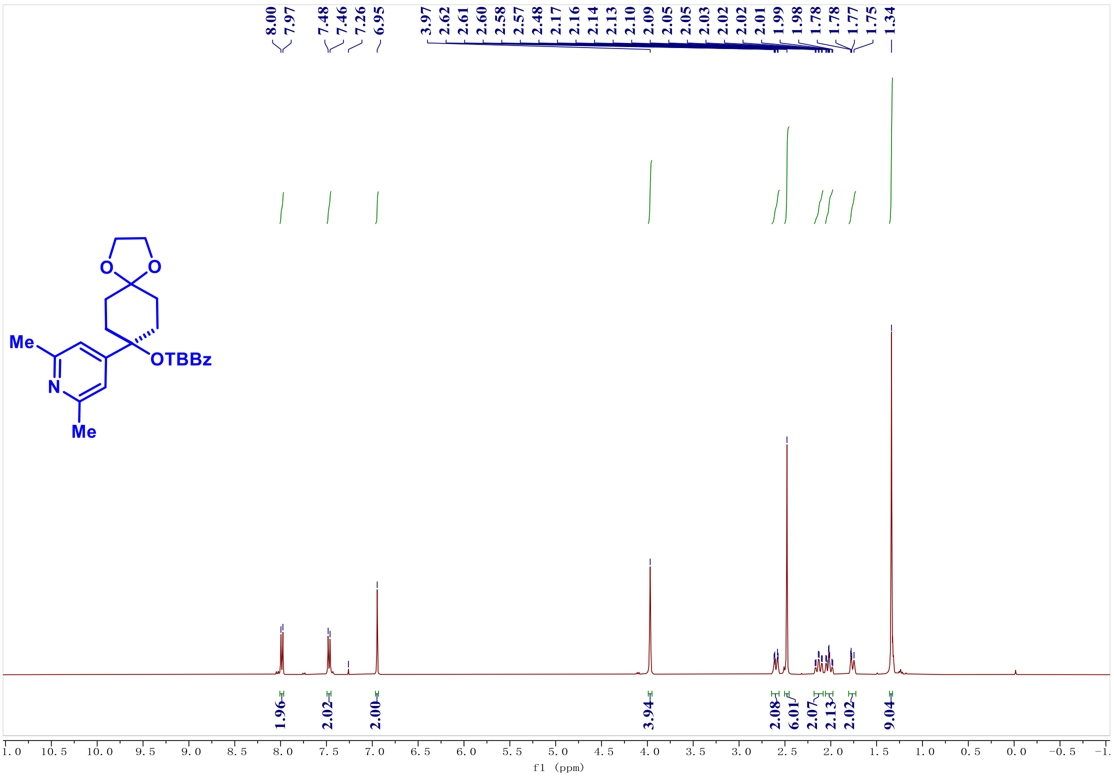

^{13}C NMR of Compound S29 (101 MHz, $CDCl_3$):

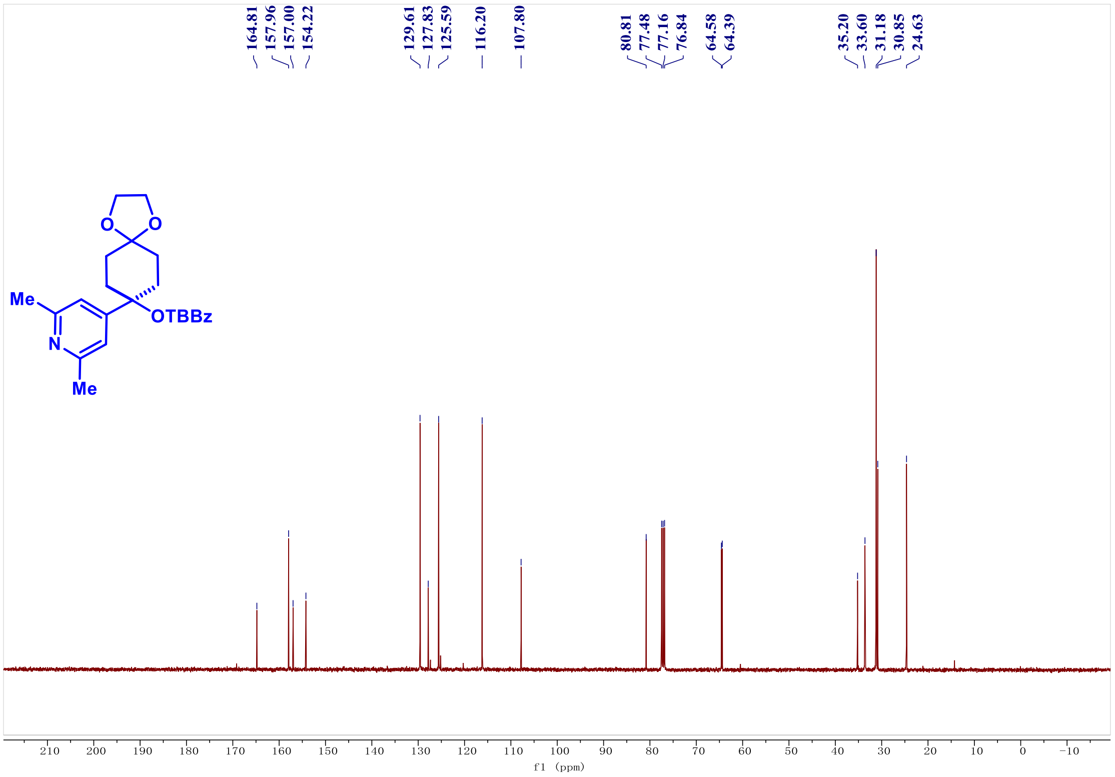

^{1}H NMR of Compound S30 (400 MHz, $CDCl_3$):

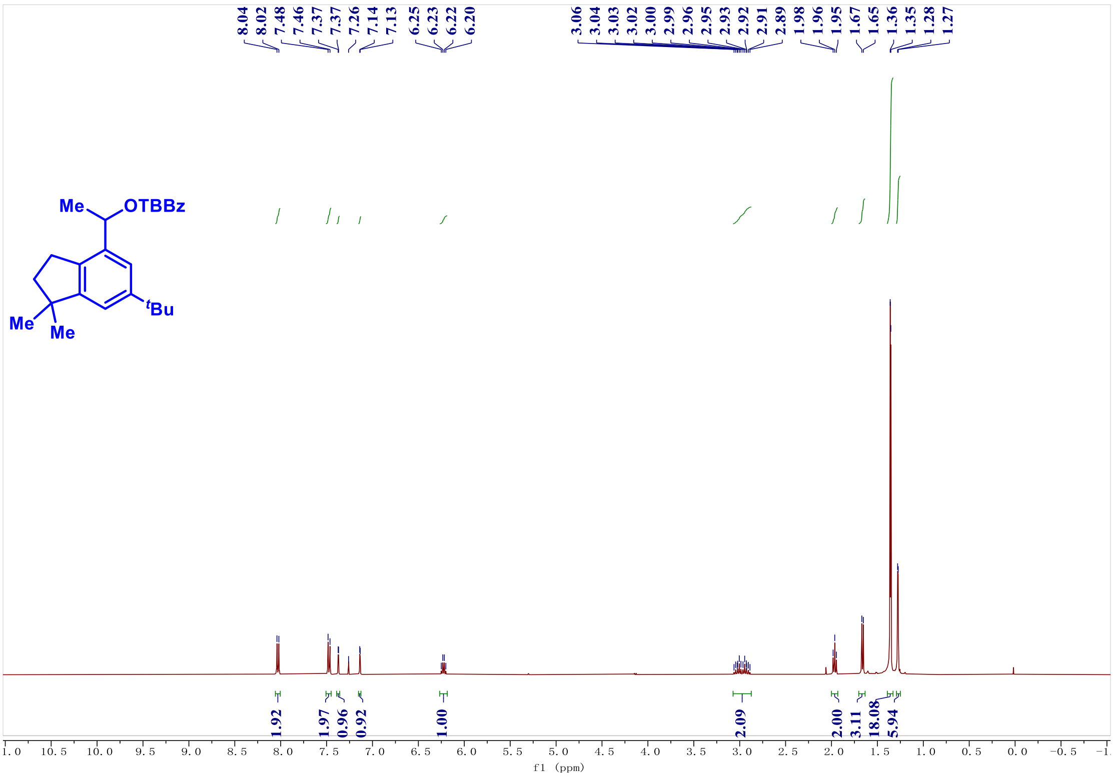

^{13}C NMR of Compound S30 (126 MHz, $CDCl_3$):

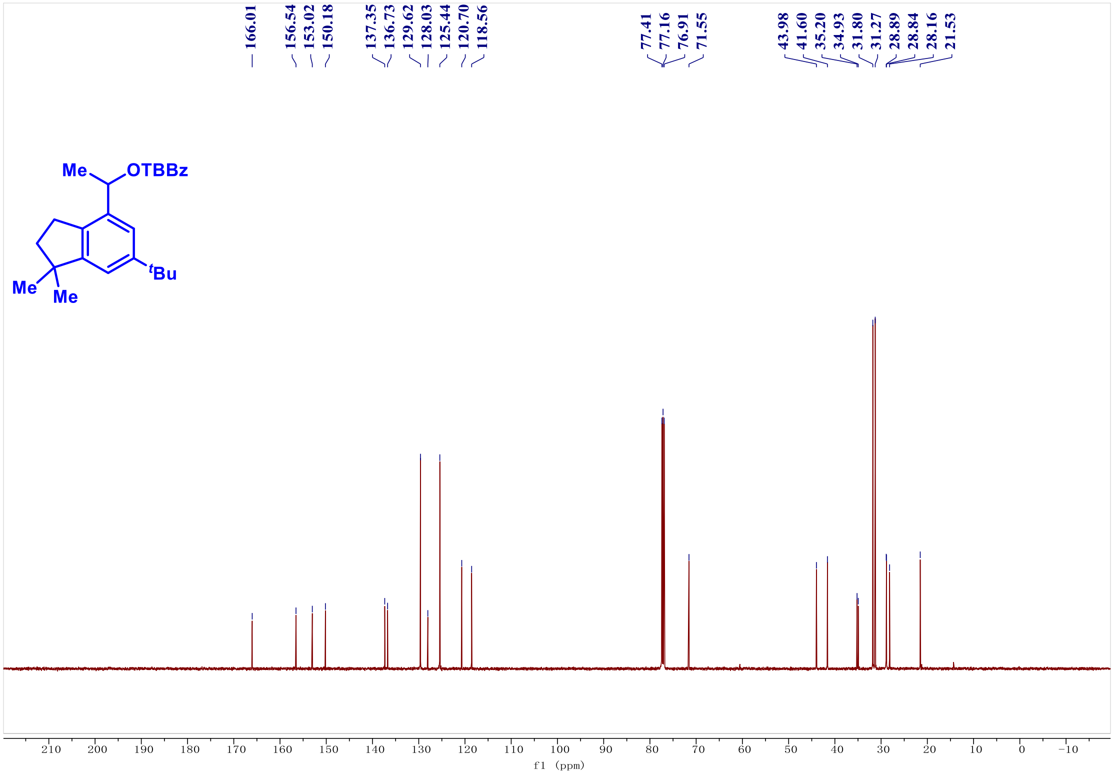

^{1}H NMR of Compound S31 (500 MHz, $CDCl_3$):

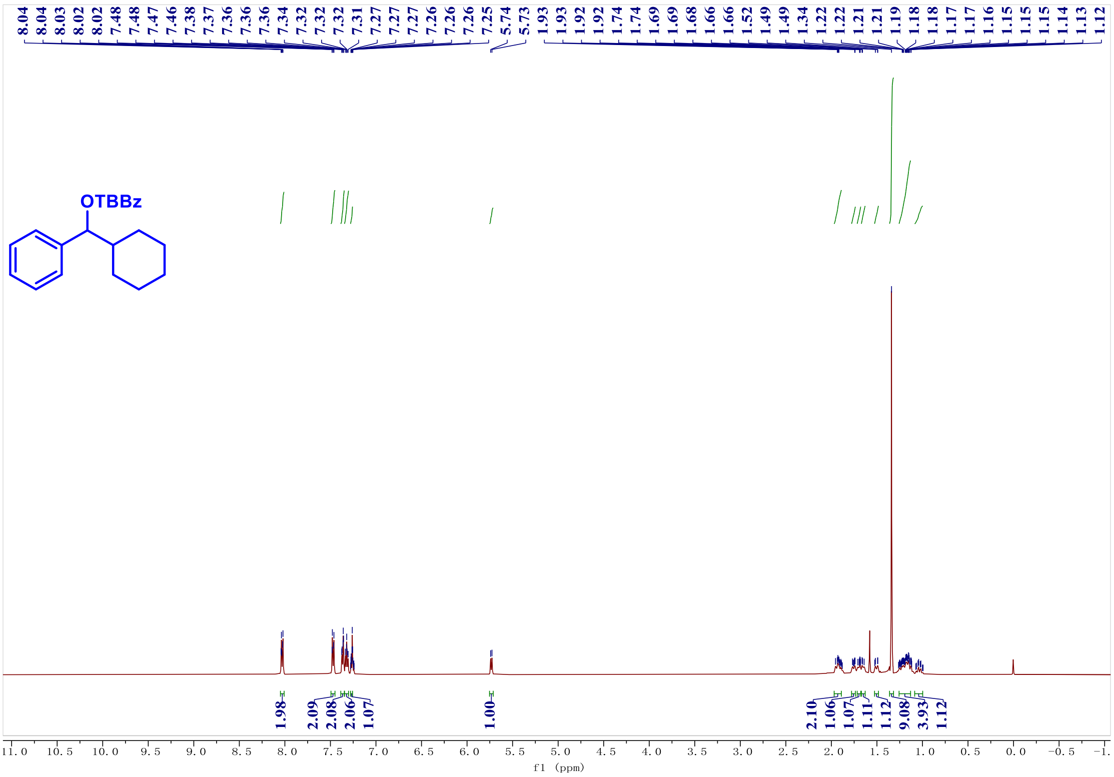

^{13}C NMR of Compound S31 (126 MHz, $CDCl_3$):

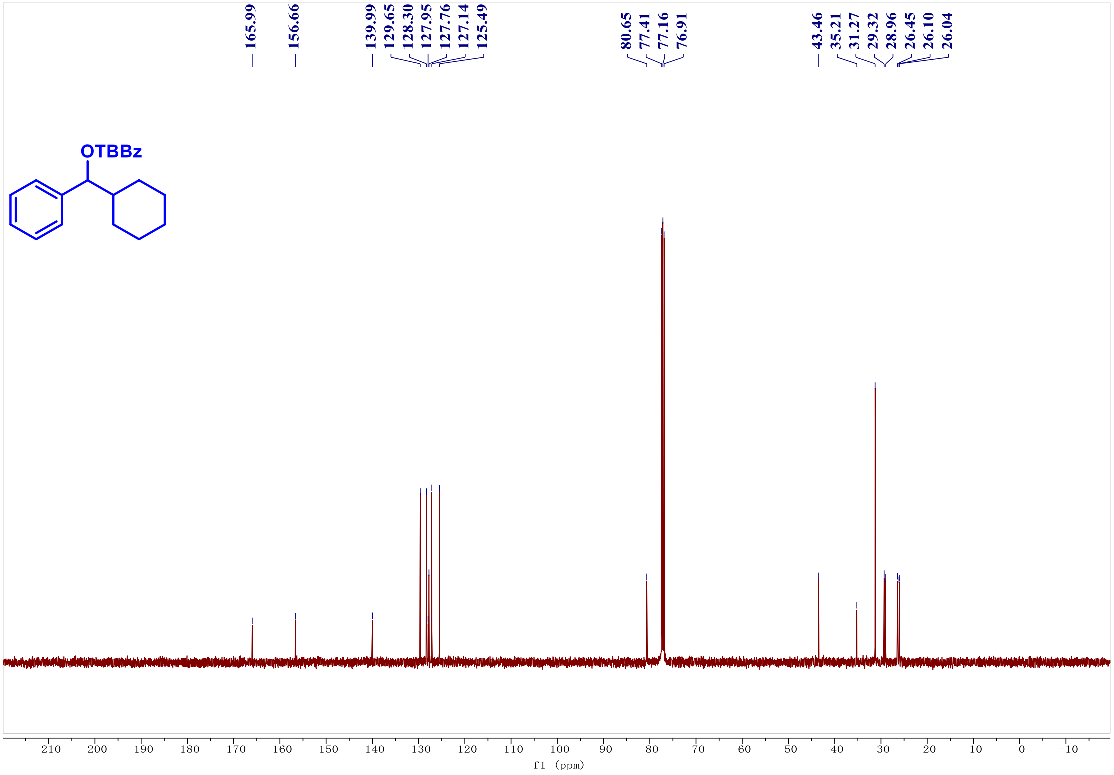

^{1}H NMR of Compound S32 (400 MHz, $CDCl_3$):

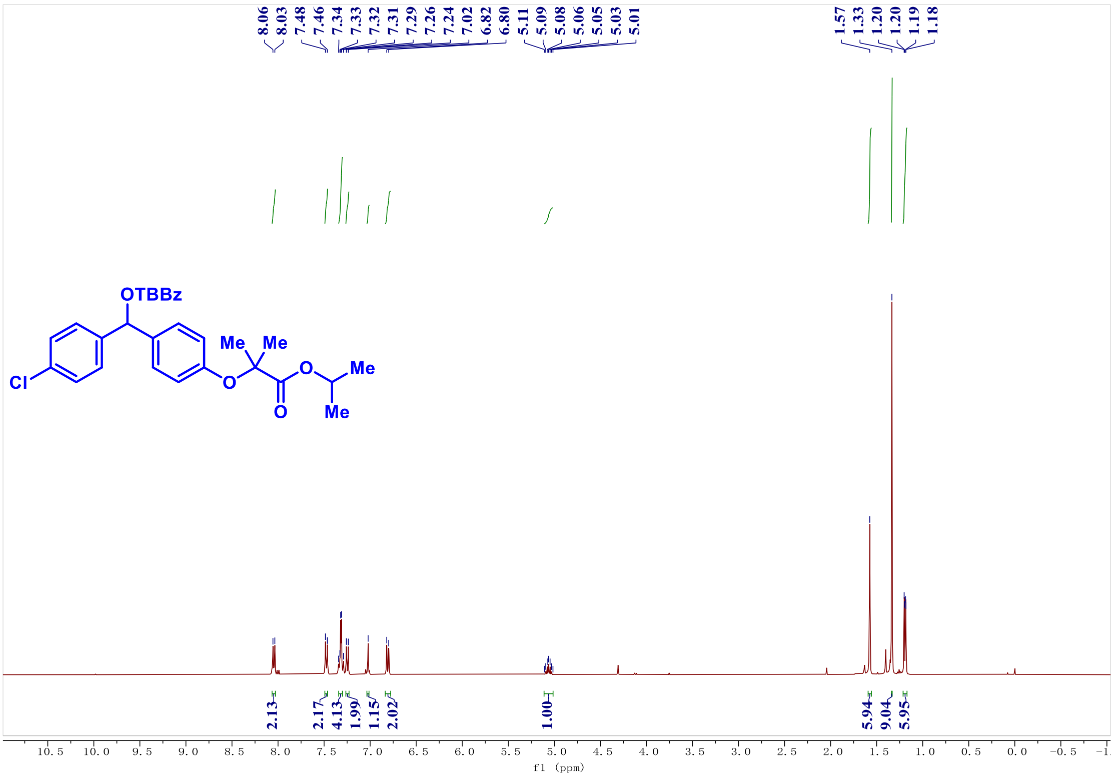

^{13}C NMR of Compound S32 (101 MHz, $CDCl_3$):

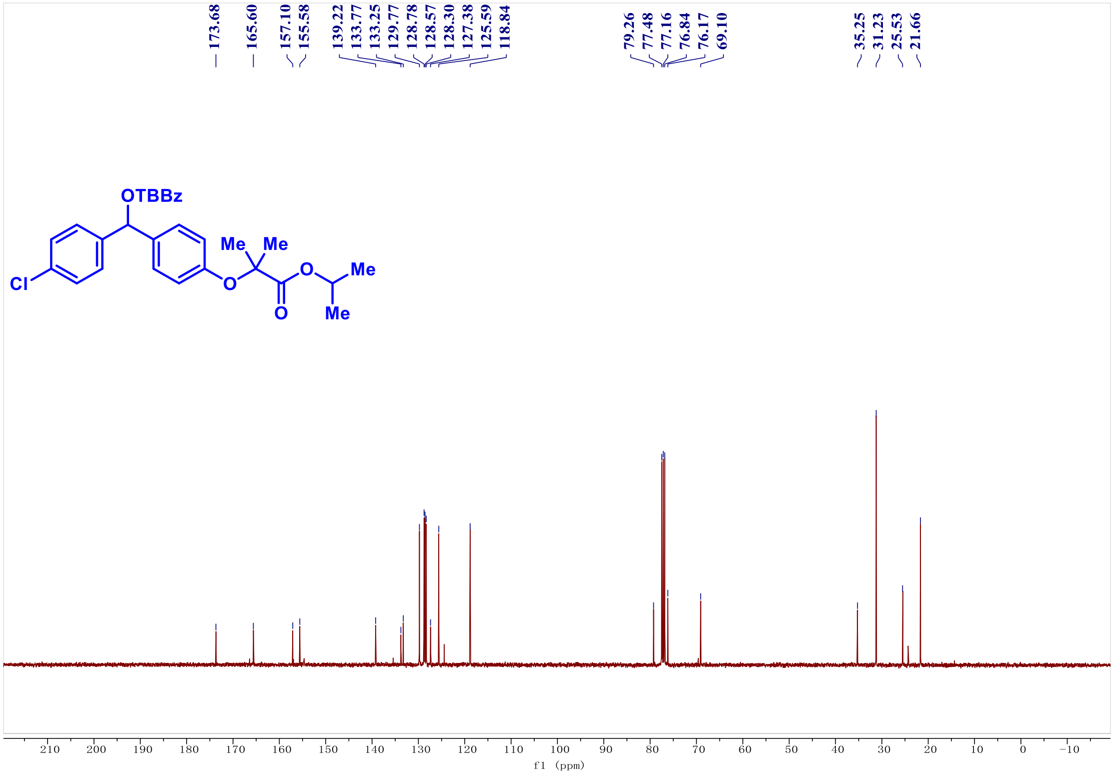

^{1}H NMR of Compound S33(500 MHz, $CDCl_3$):

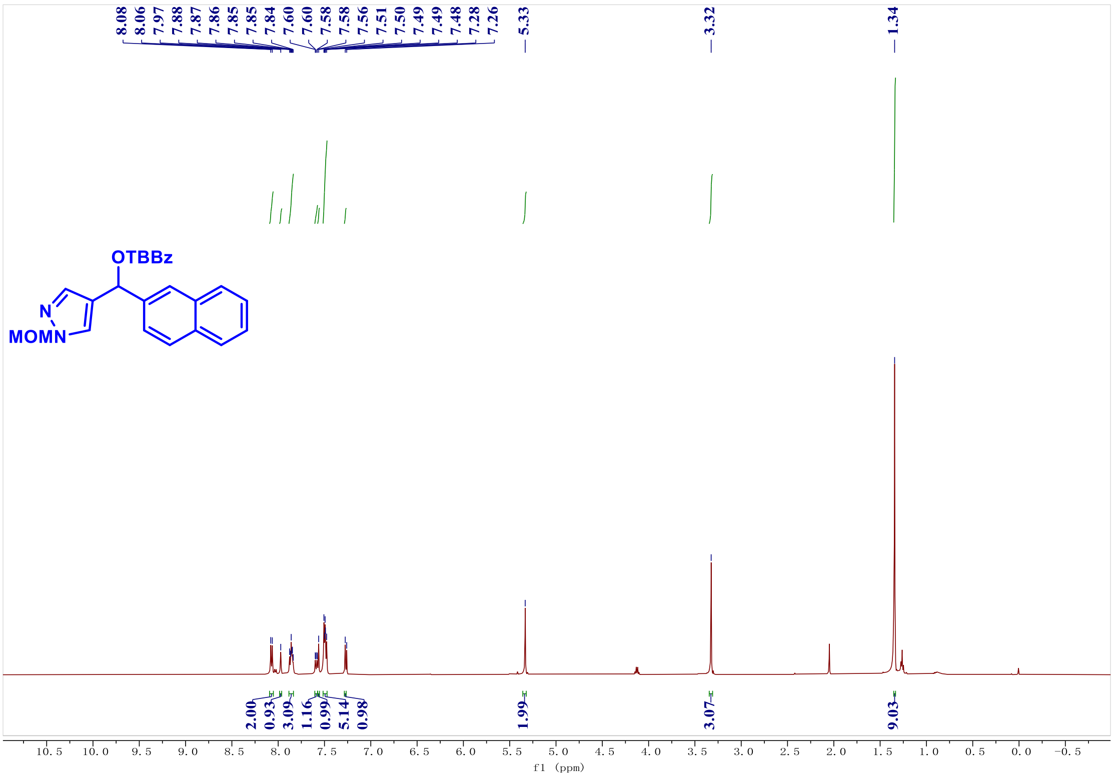

^{13}C NMR of Compound S33 (126 MHz, $CDCl_3$):

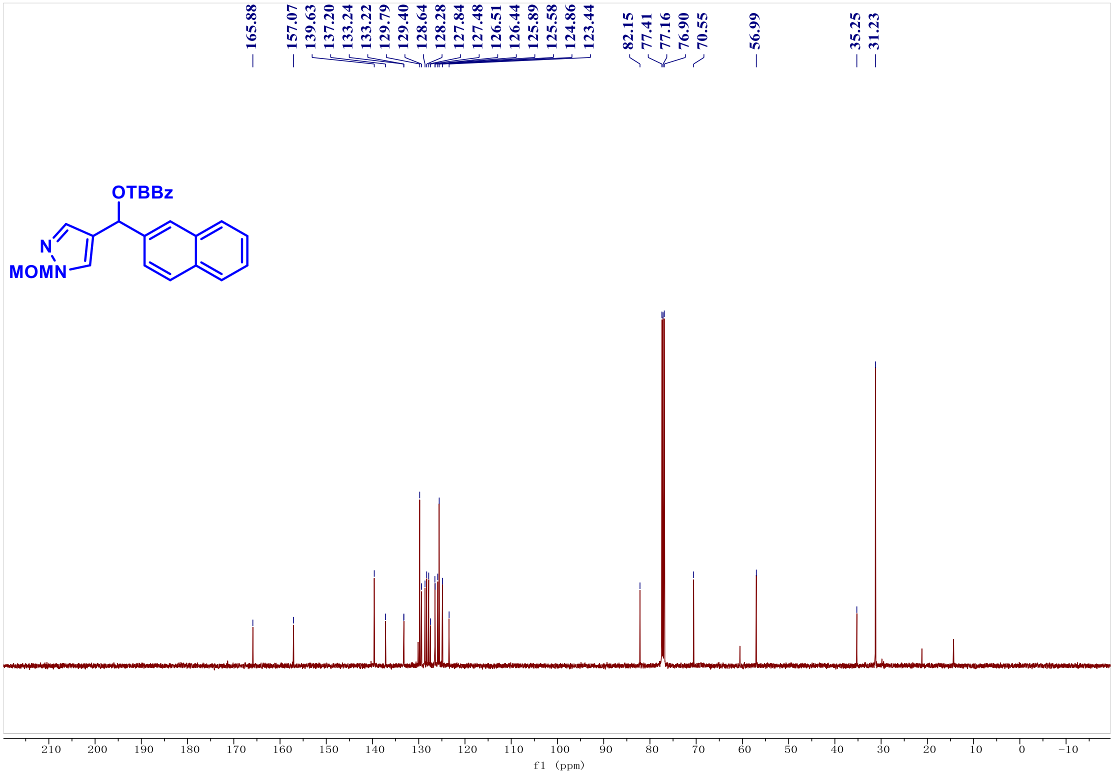

^{1}H NMR of Compound S34 (400 MHz, $CDCl_3$):

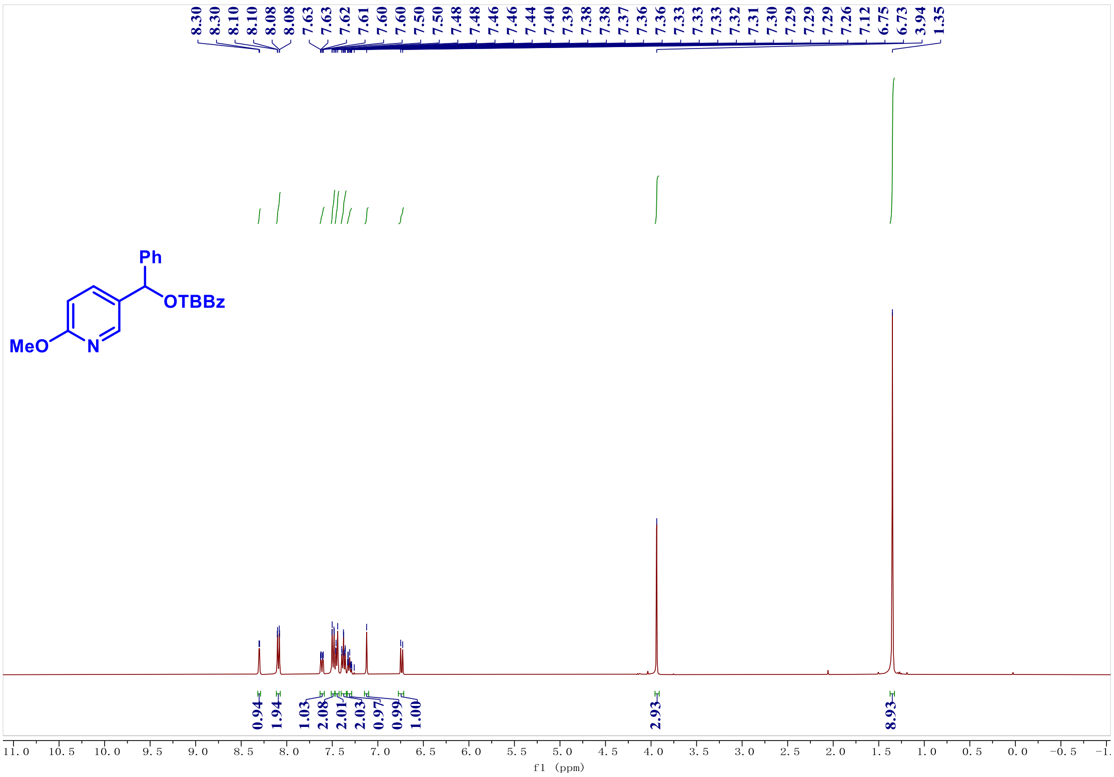

^{13}C NMR of Compound S34 (101 MHz, $CDCl_3$):

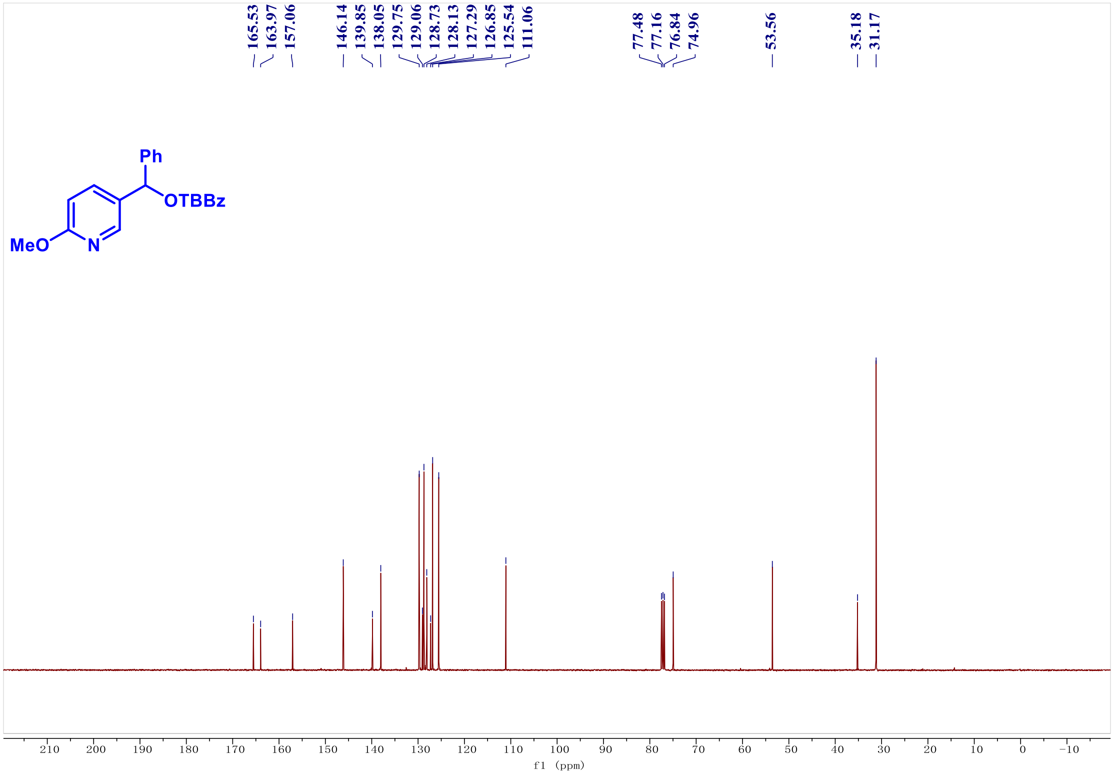

^{1}H NMR of Compound S35 (400 MHz, $CDCl_3$):

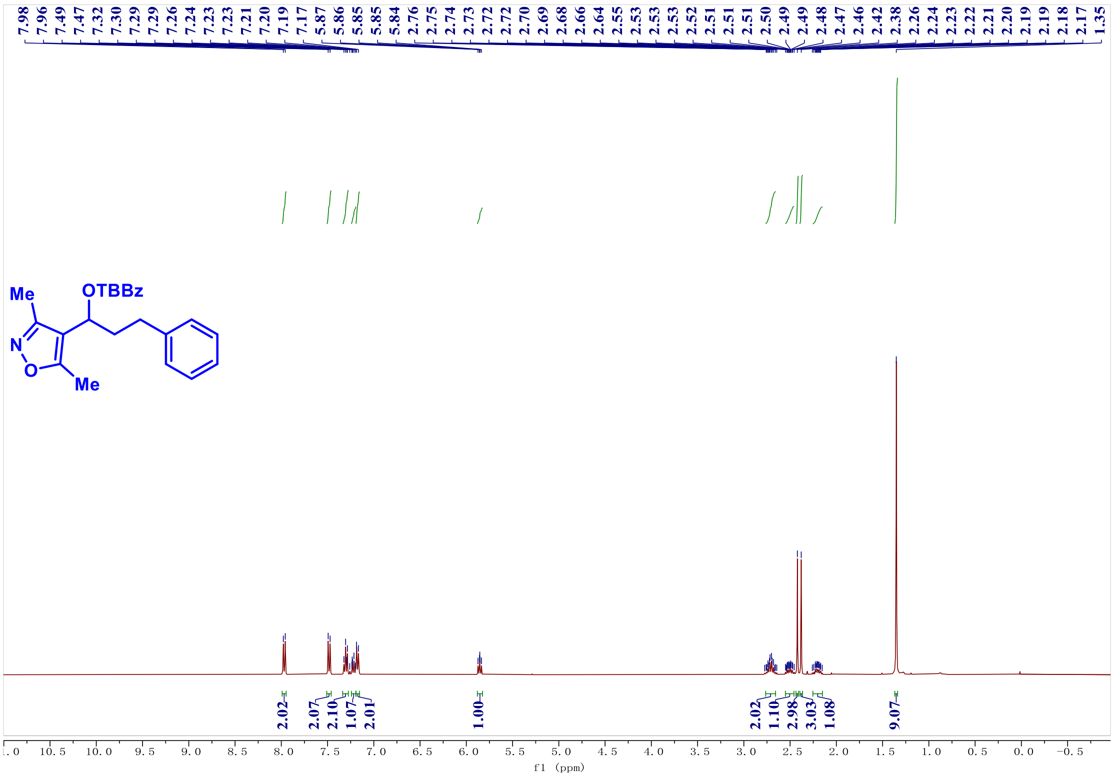

^{13}C NMR of Compound S35 (101 MHz, $CDCl_3$):

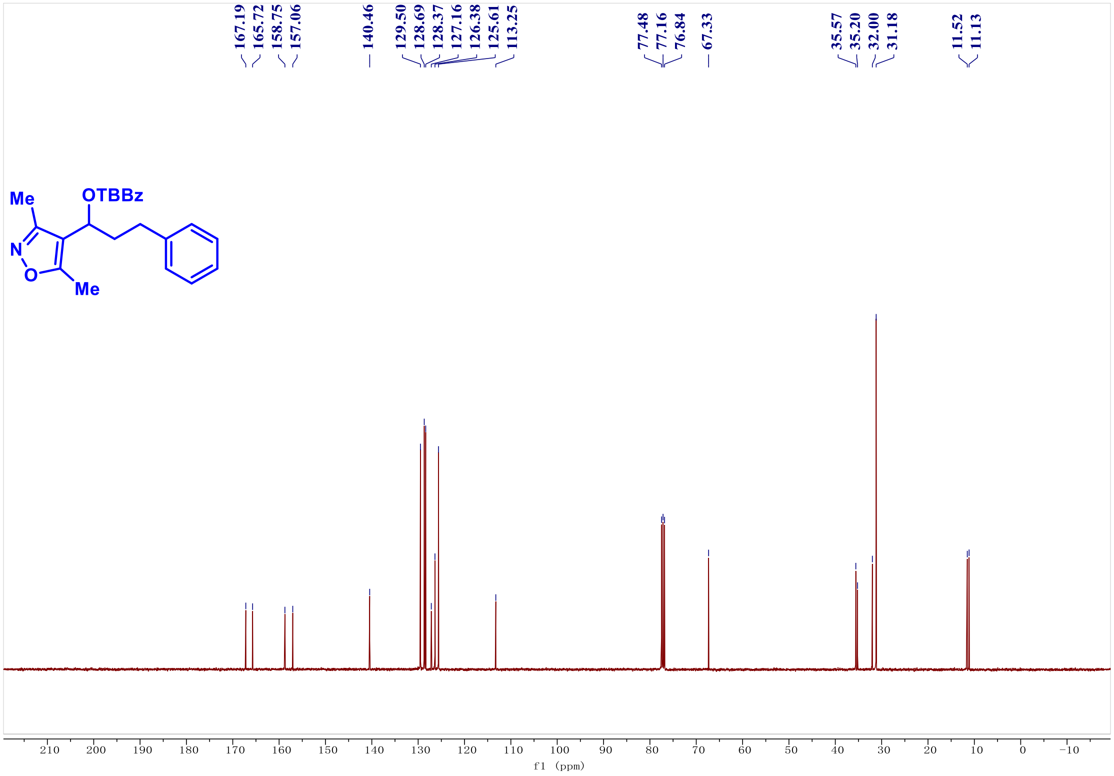

^{1}H NMR of Compound S36 (400 MHz, $CDCl_3$):

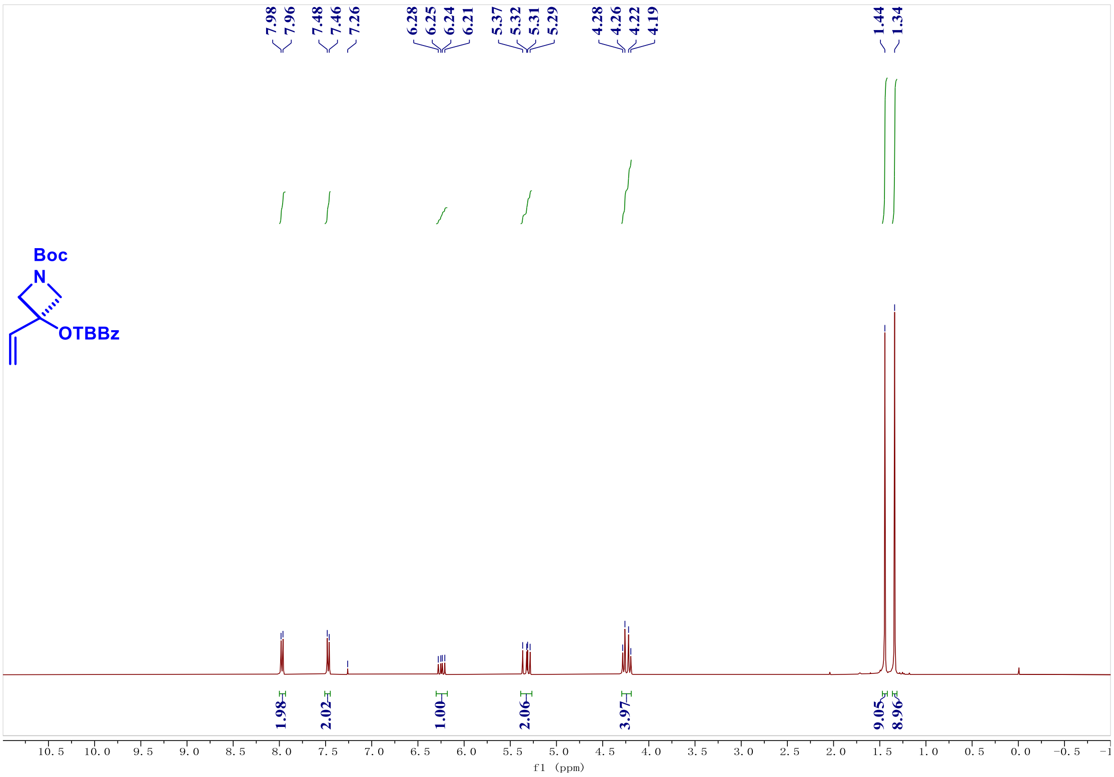

^{13}C NMR of Compound S36 (101 MHz, $CDCl_3$):

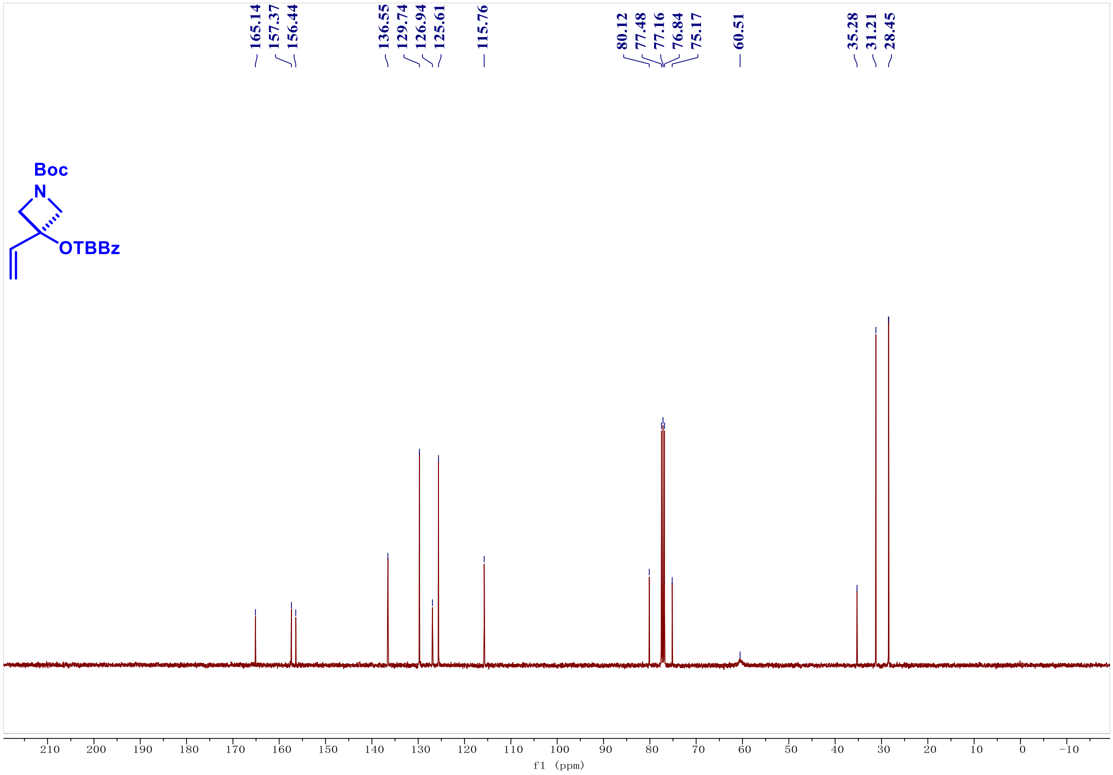

^{1}H NMR of Compound S37 (400 MHz, $CDCl_3$):

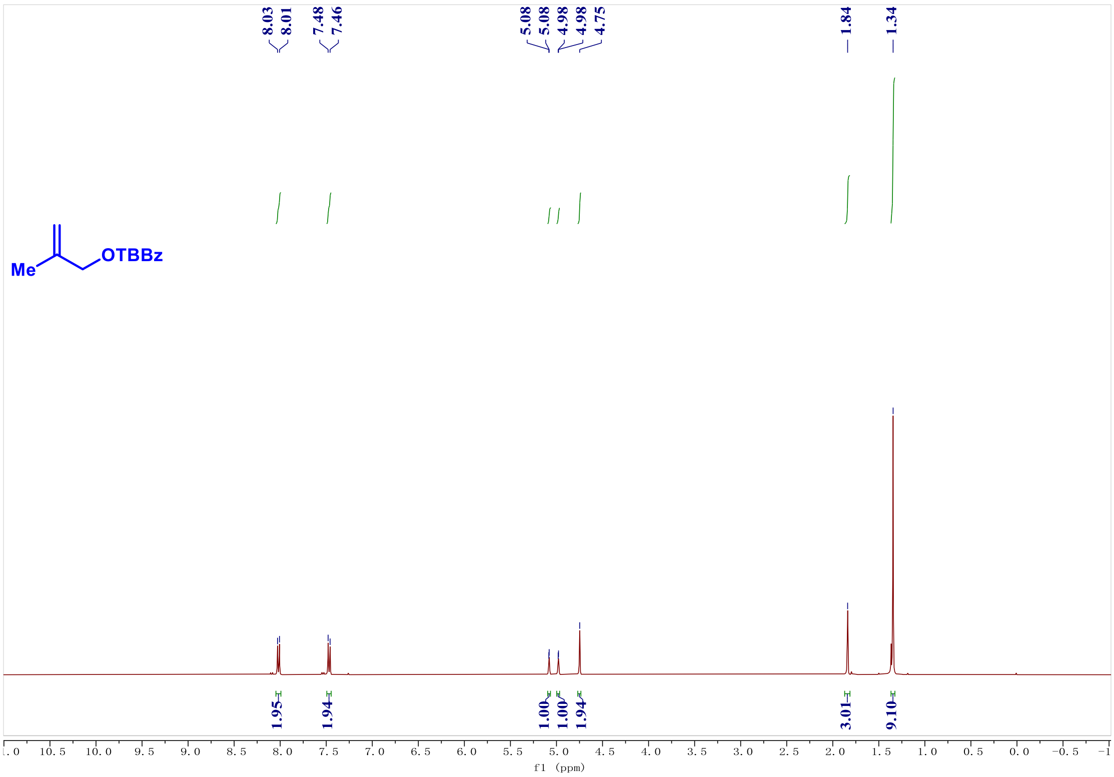

^{13}C NMR of Compound S37 (101 MHz, $CDCl_3$):

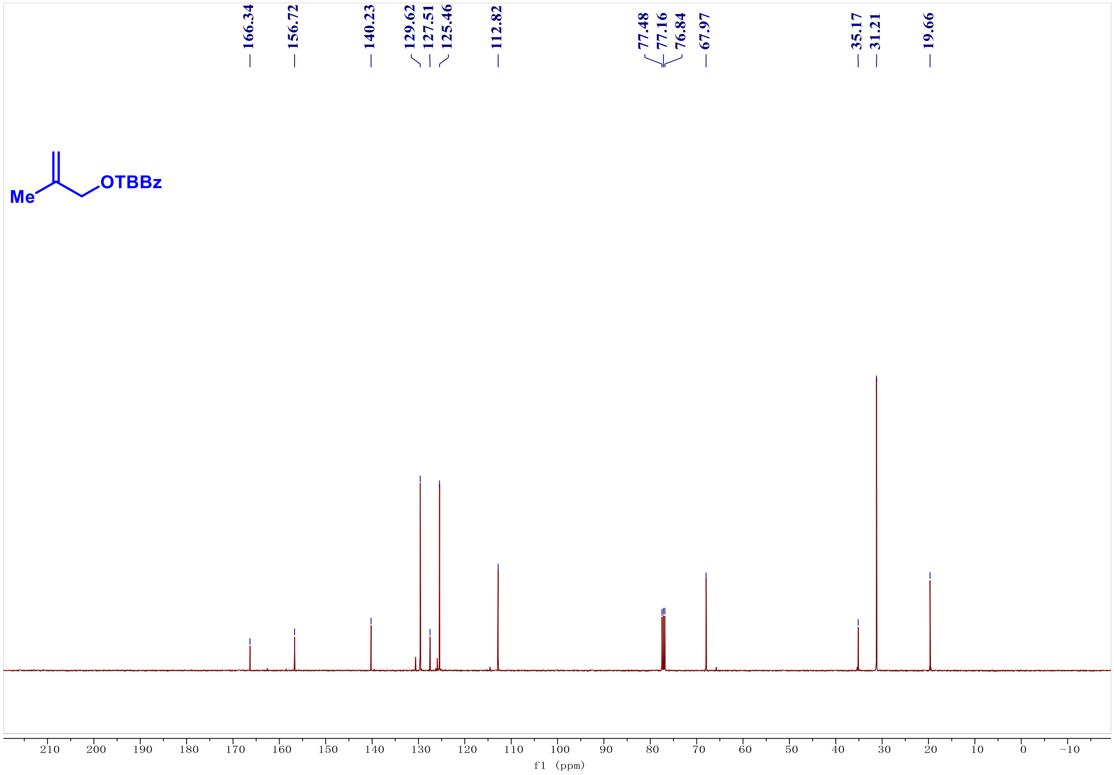

^{1}H NMR of Compound S38 (400 MHz, $CDCl_3$):

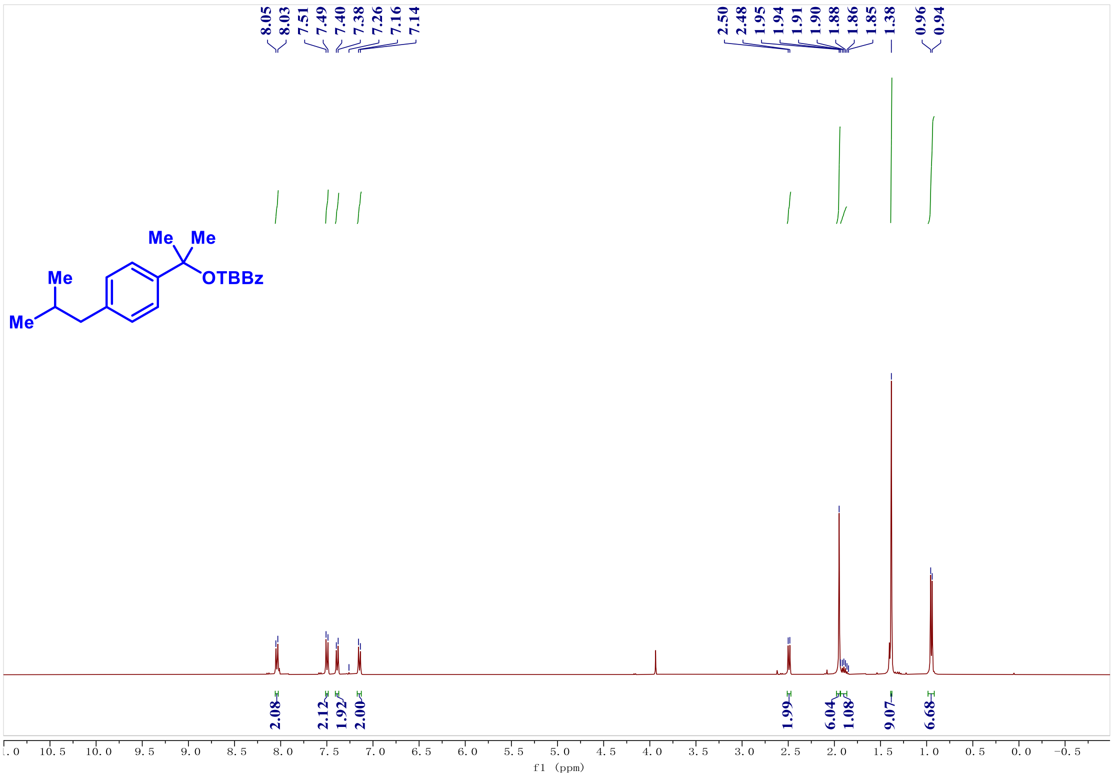

^{13}C NMR of Compound S38 (101 MHz, $CDCl_3$):

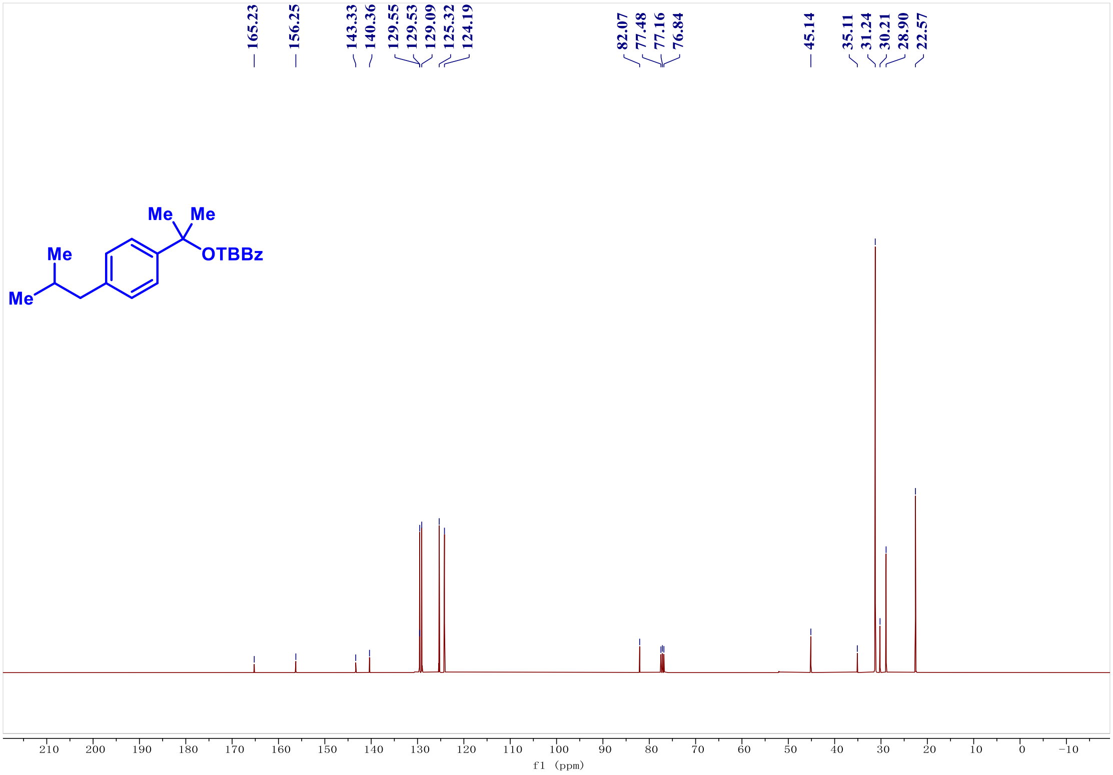

^{1}H NMR of Compound S39 (400 MHz, CDCl$_3$):

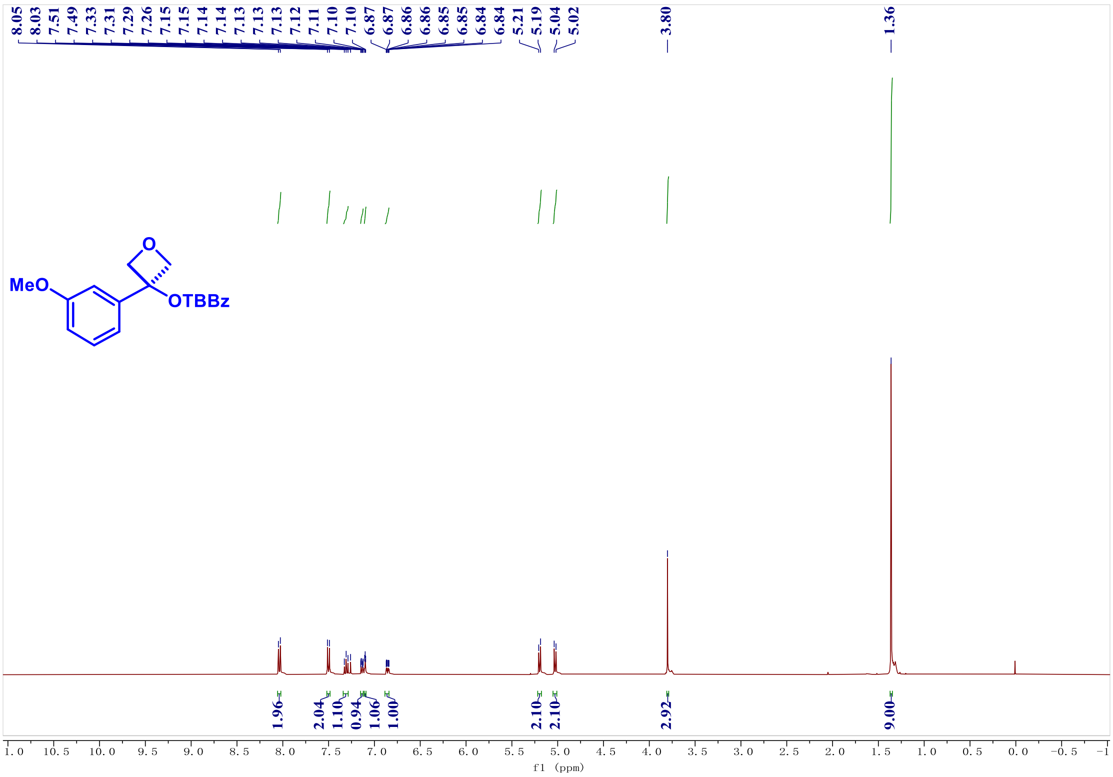

^{13}C NMR of Compound S39 (101 MHz, CDCl$_3$):

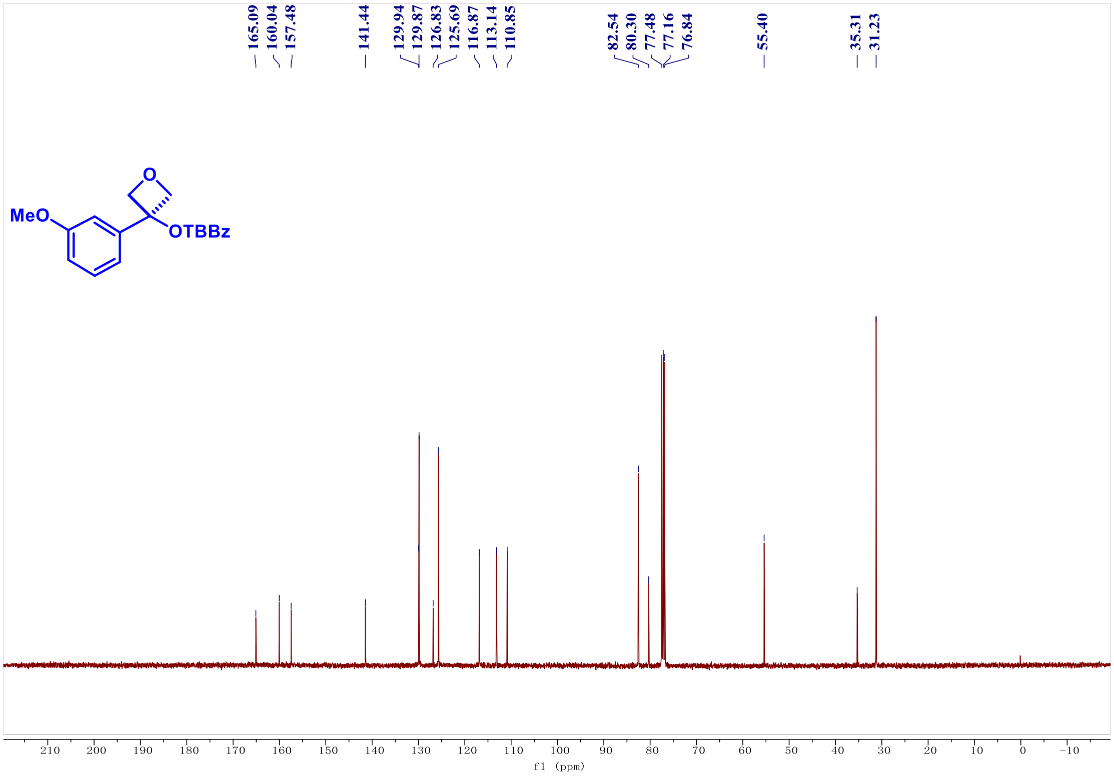

^{1}H NMR of Compound 6 (500 MHz, $CDCl_3$):

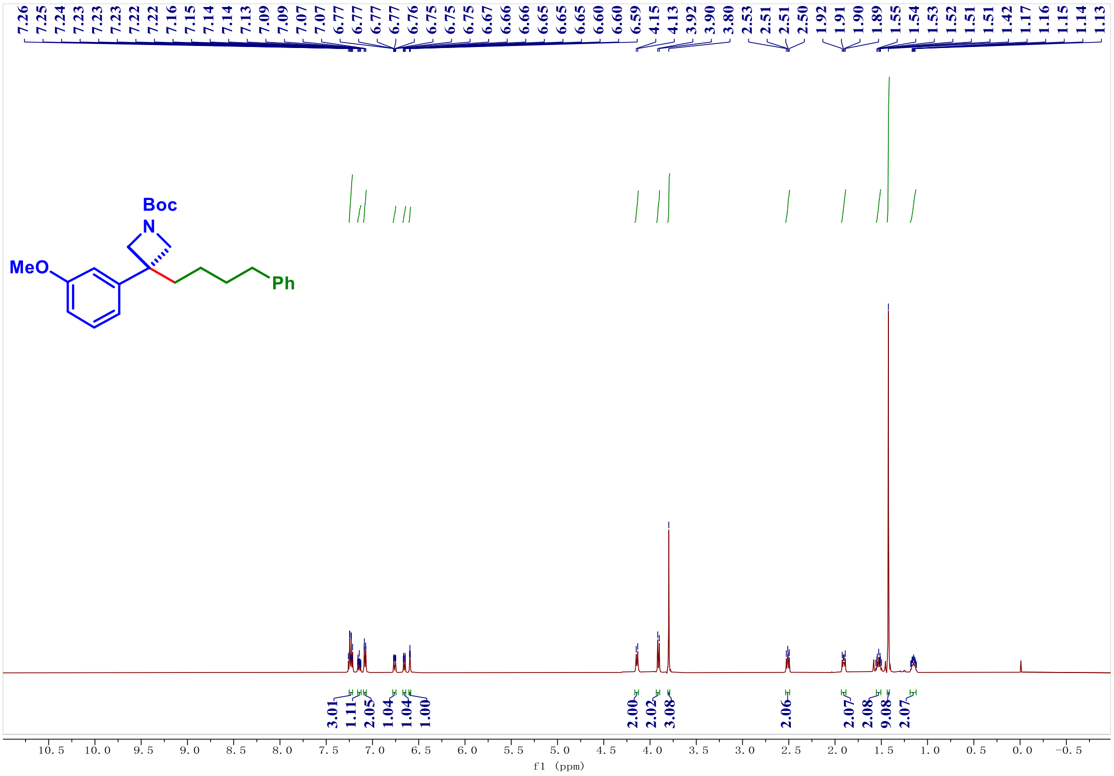

^{13}C NMR of Compound 6 (101 MHz, $CDCl_3$):

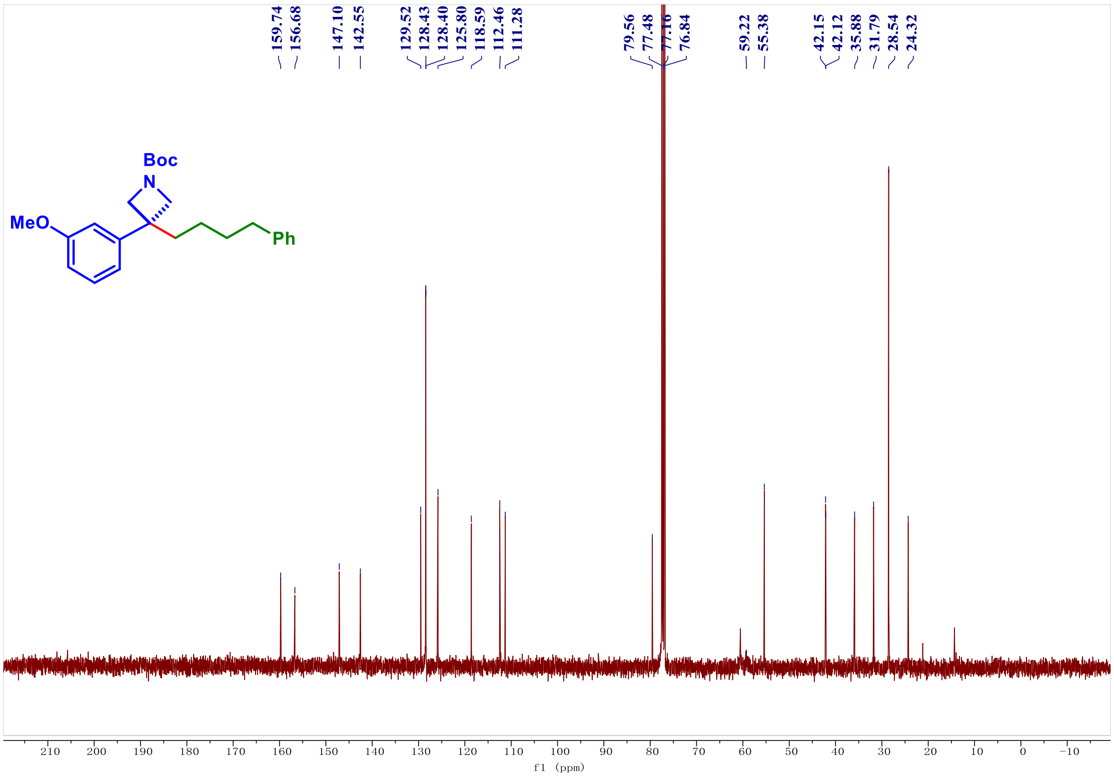

^{1}H NMR of Compound 9 (500 MHz, $CDCl_3$):

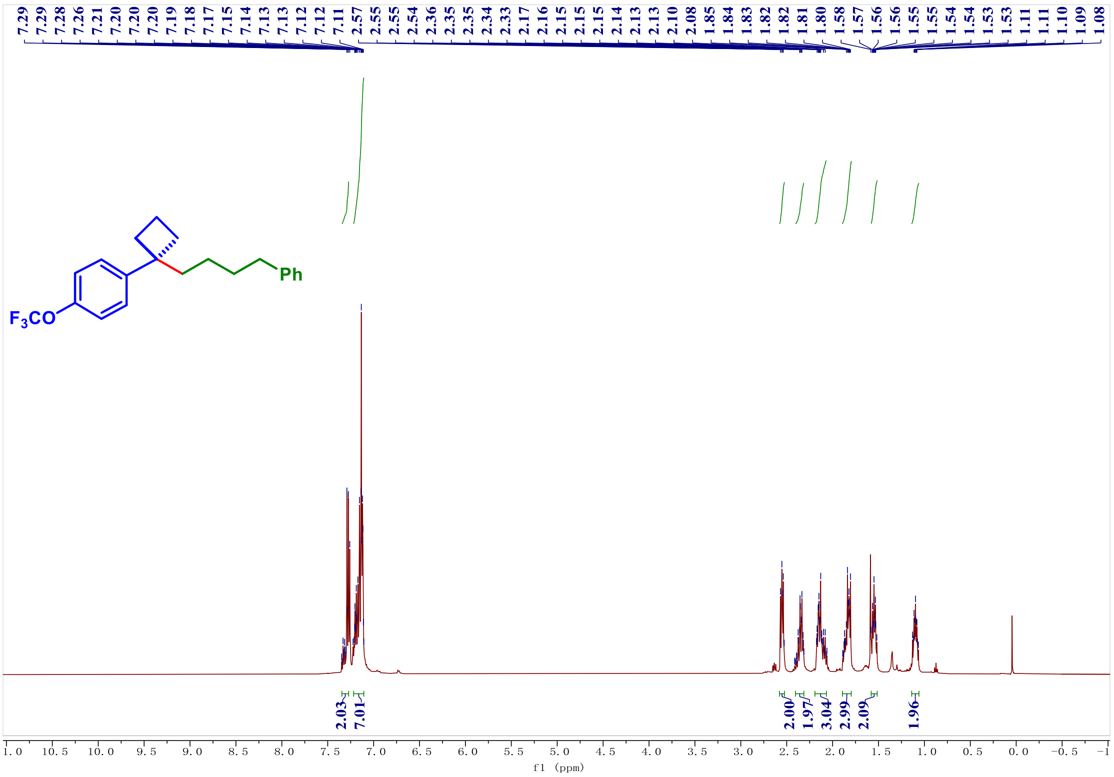

^{13}C NMR of Compound 9 (126 MHz, $CDCl_3$):

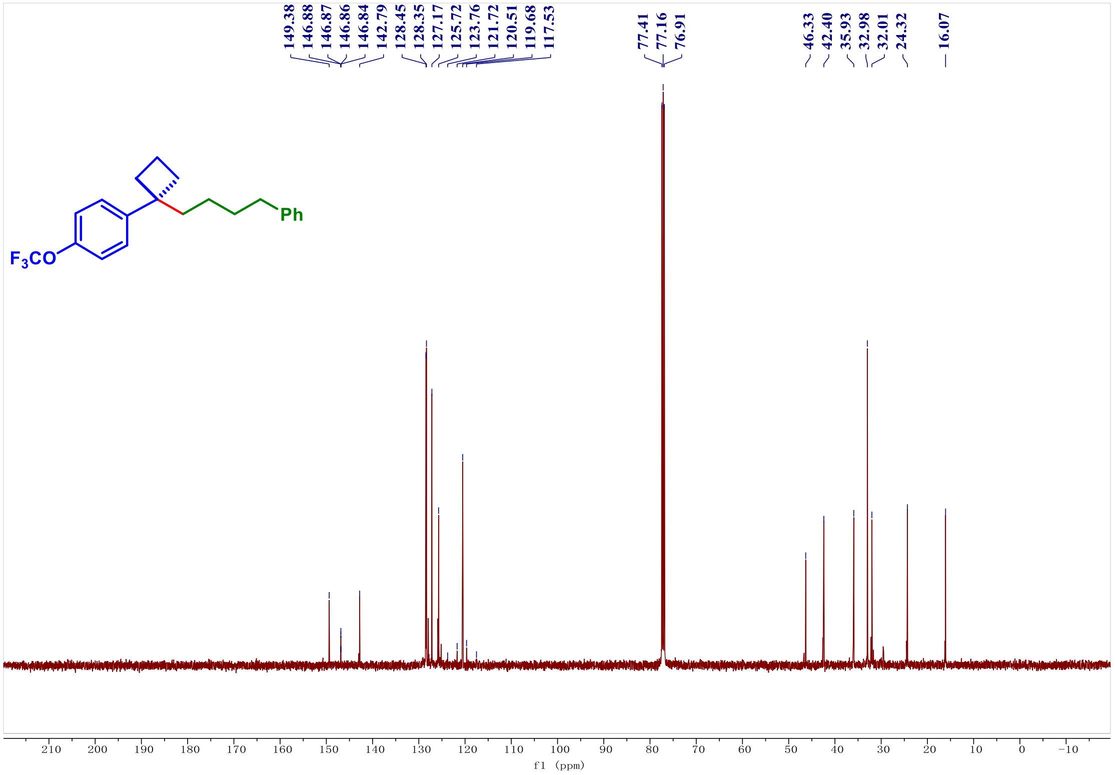

^{19}F NMR of Compound 9 (471 MHz, $CDCl_3$):

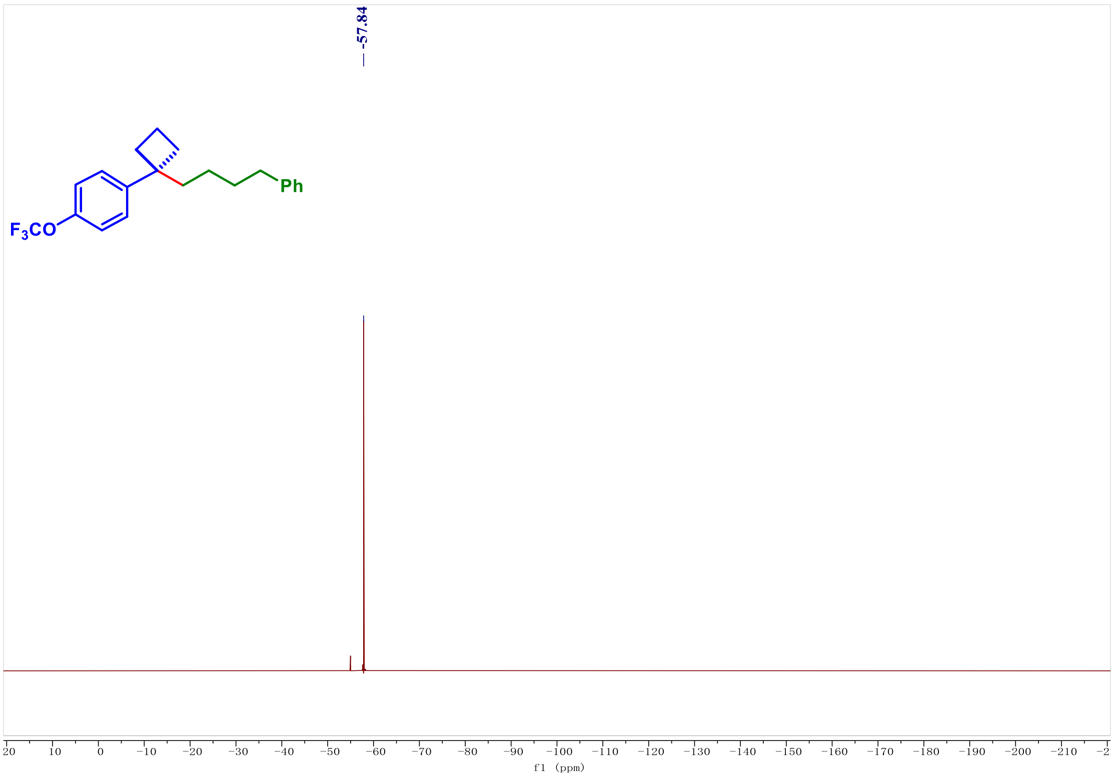

^{1}H NMR of Compound 10 (500 MHz, $CDCl_3$):

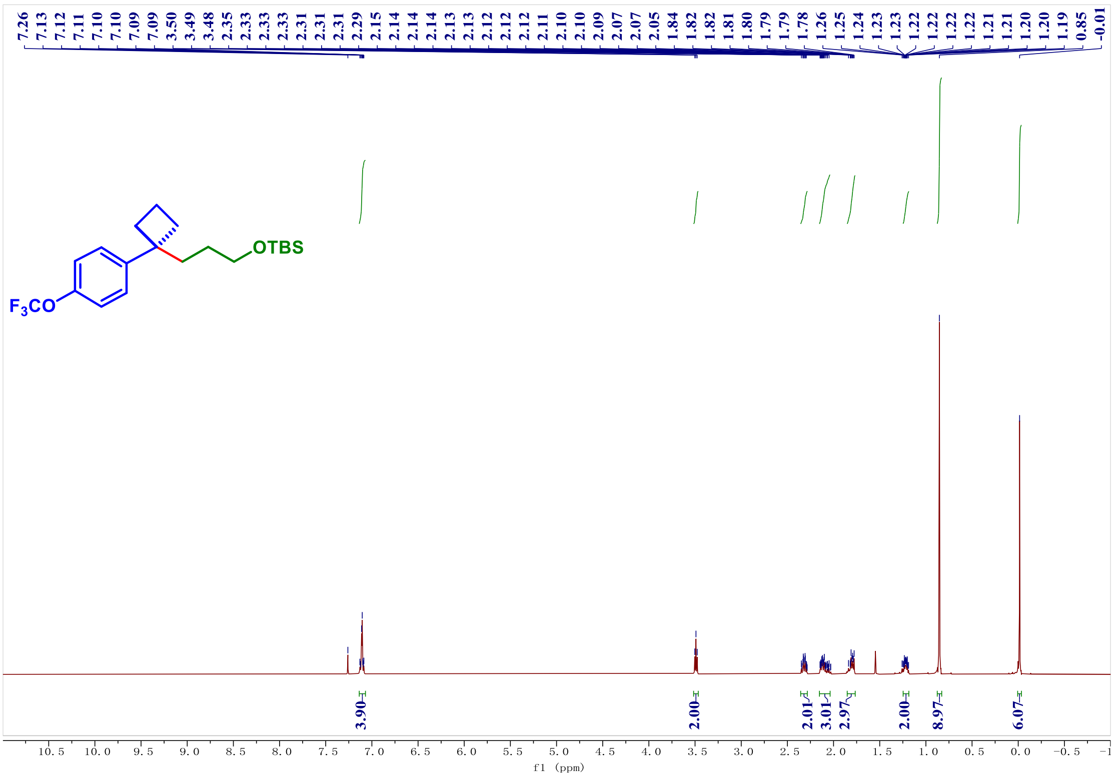

^{13}C NMR of Compound 10 (126 MHz, $CDCl_3$):

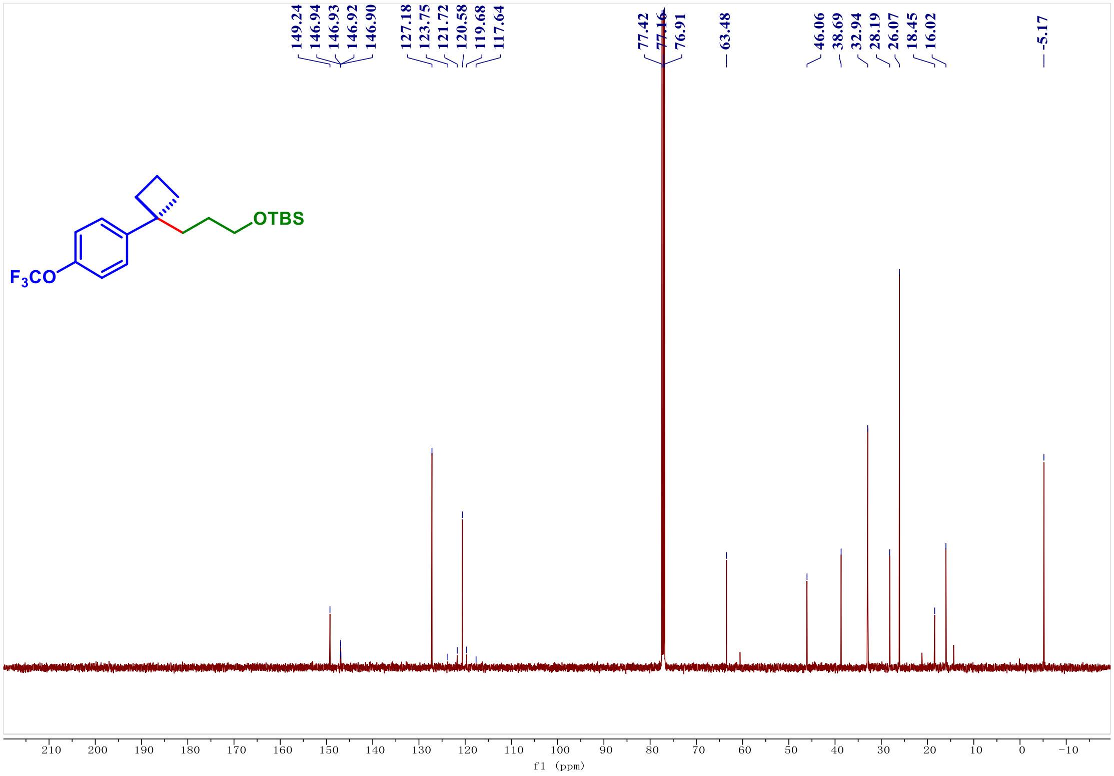

^{19}F NMR of Compound 10 (471 MHz, $CDCl_3$):

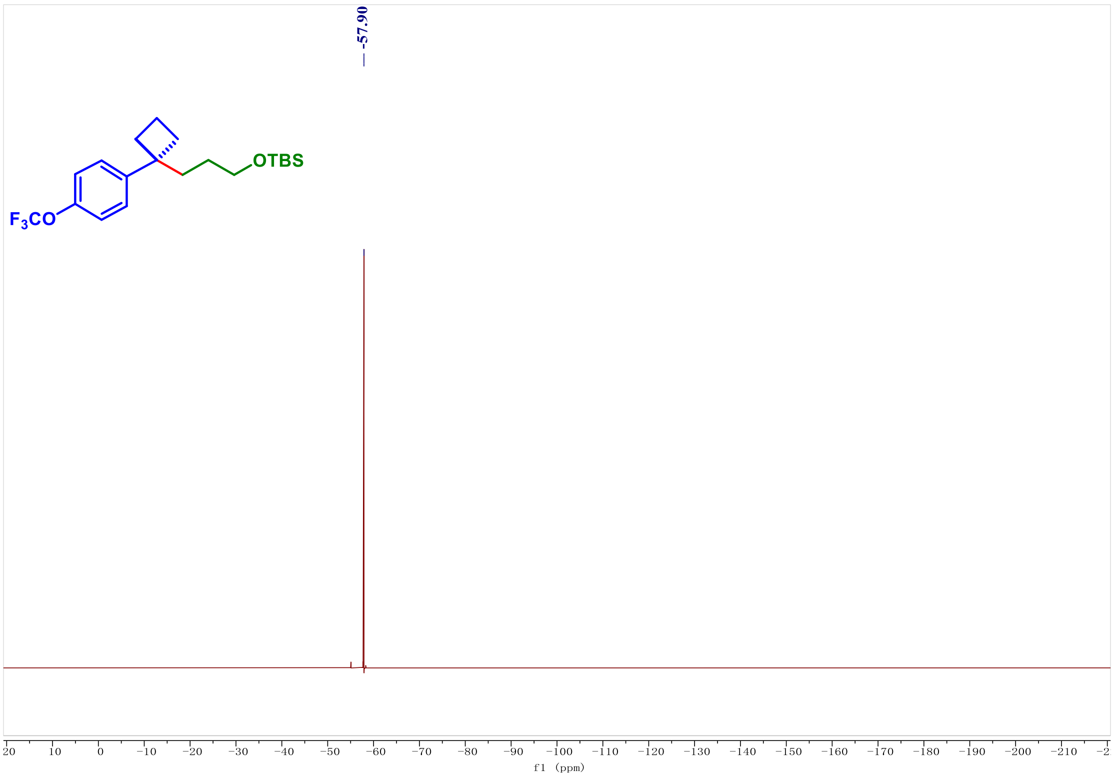

^{1}H NMR of Compound 11 (400 MHz, $CDCl_3$):

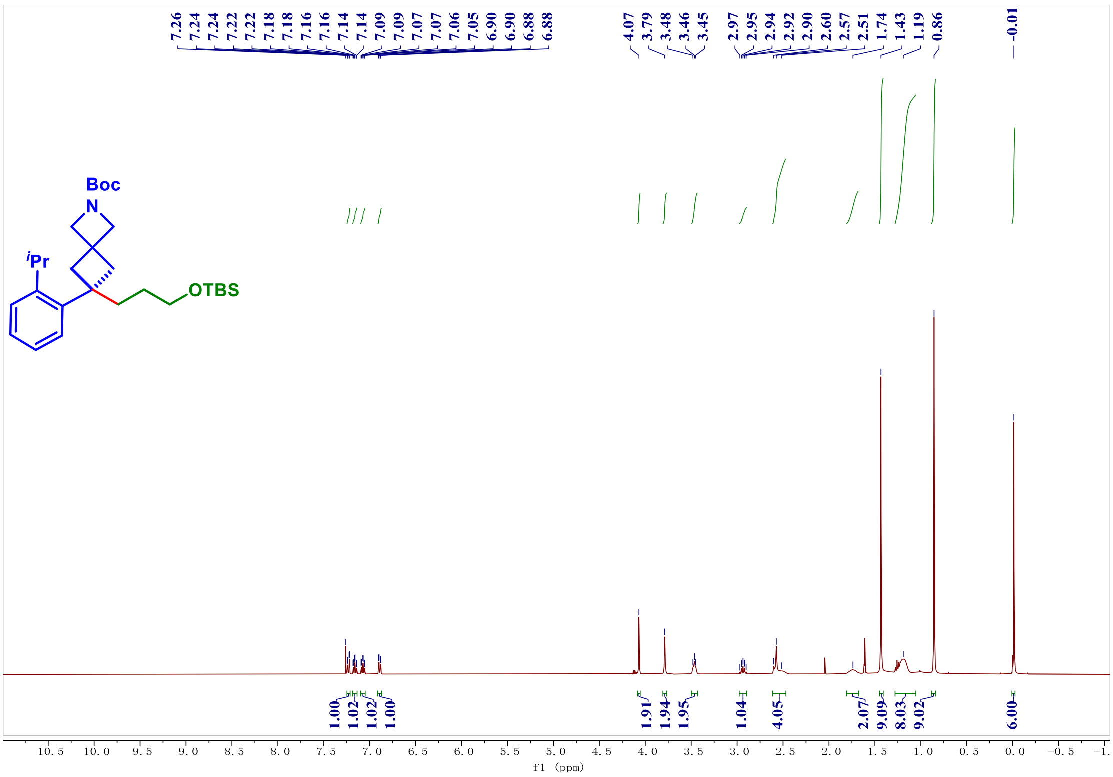

^{13}C NMR of Compound 11 (101 MHz, $CDCl_3$):

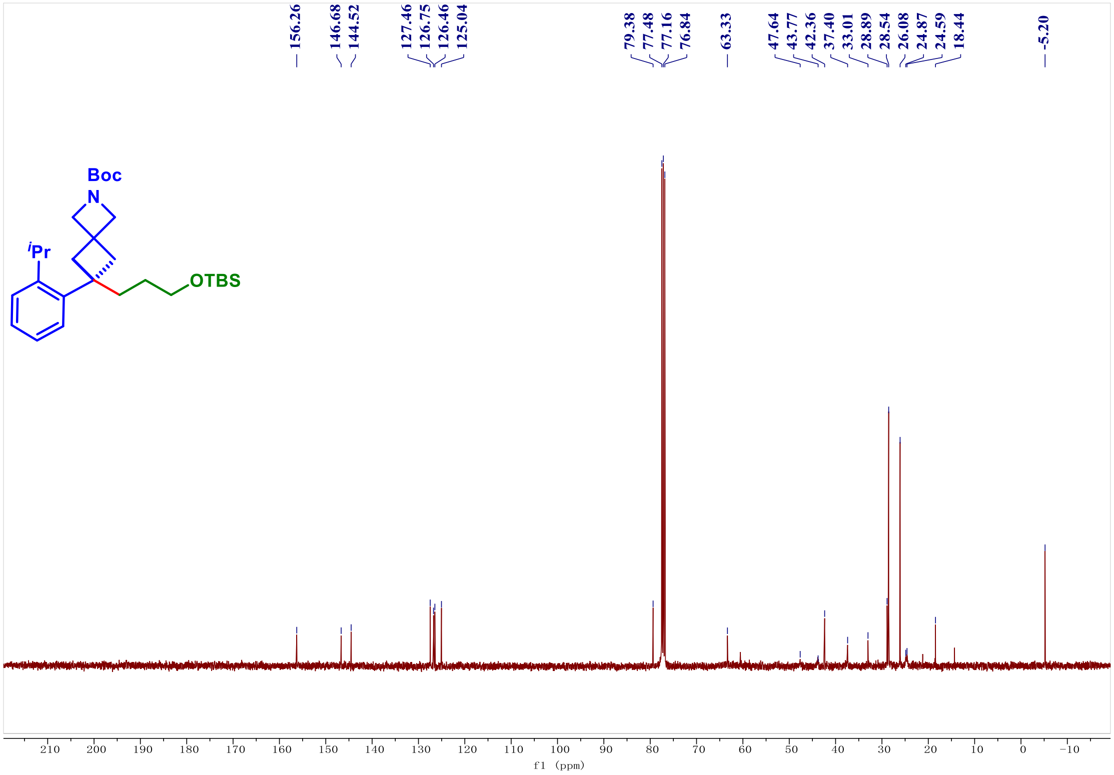

^{1}H NMR of Compound 12 (400 MHz, $CDCl_3$):

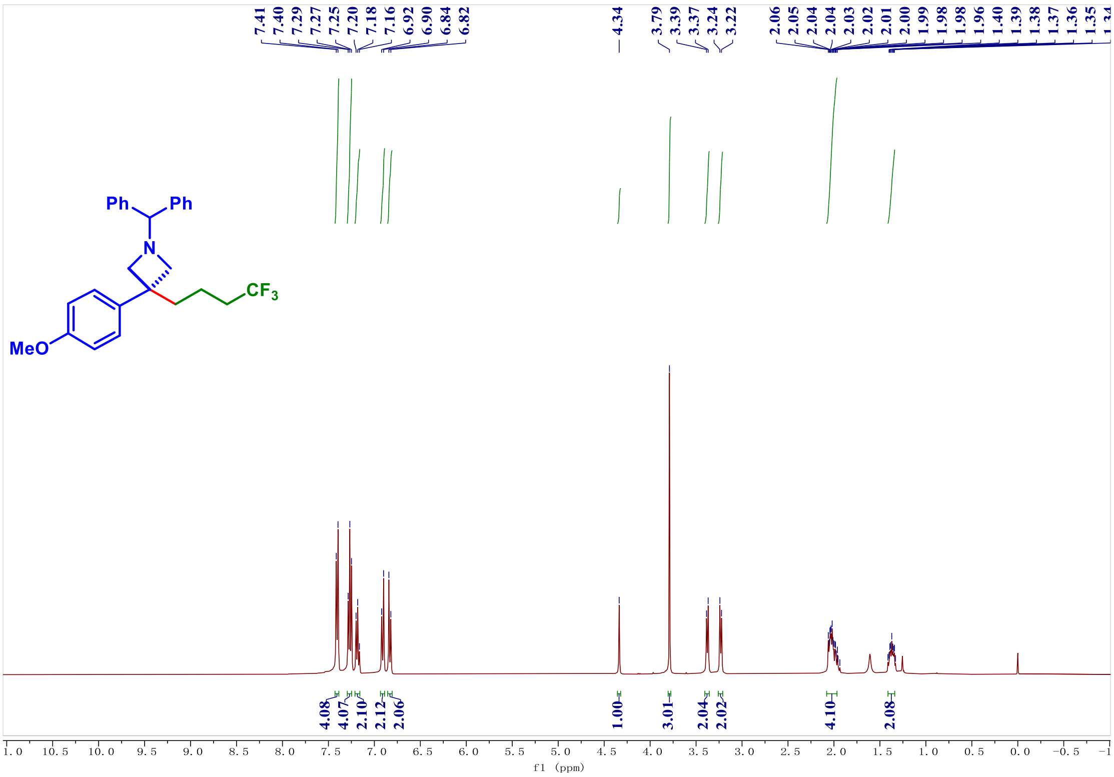

^{13}C NMR of Compound 12 (101 MHz, $CDCl_3$):

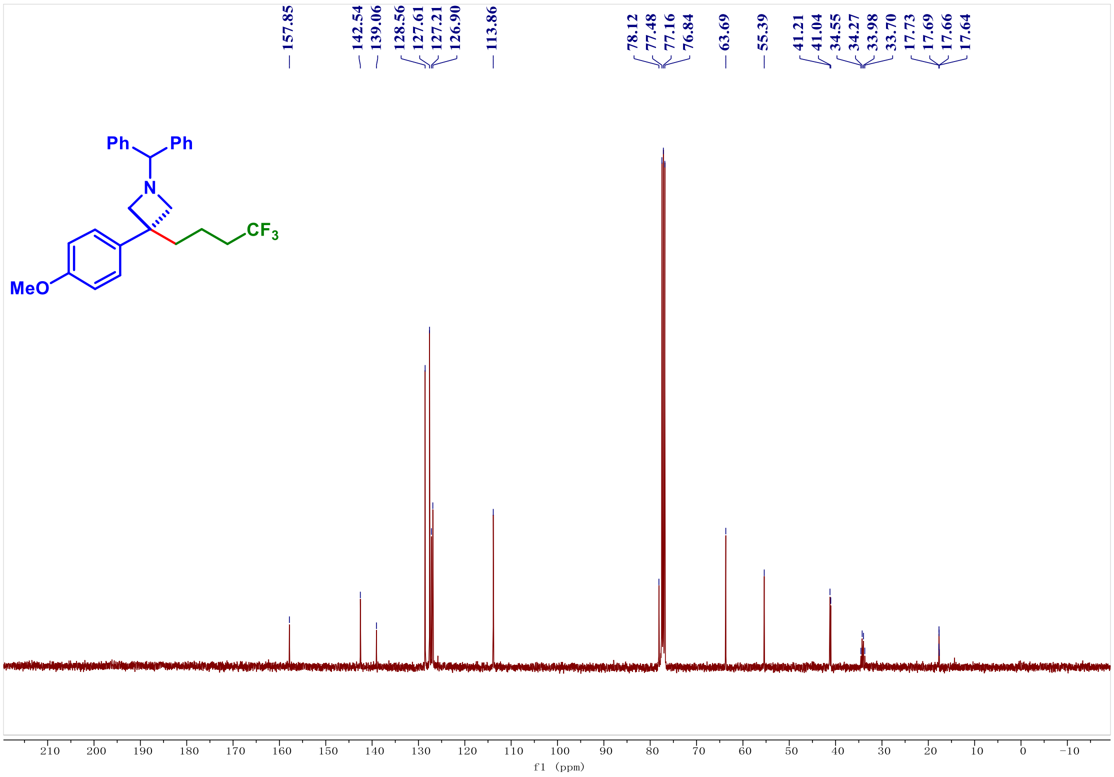

^{19}F NMR of Compound 12 (376 MHz, $CDCl_3$):

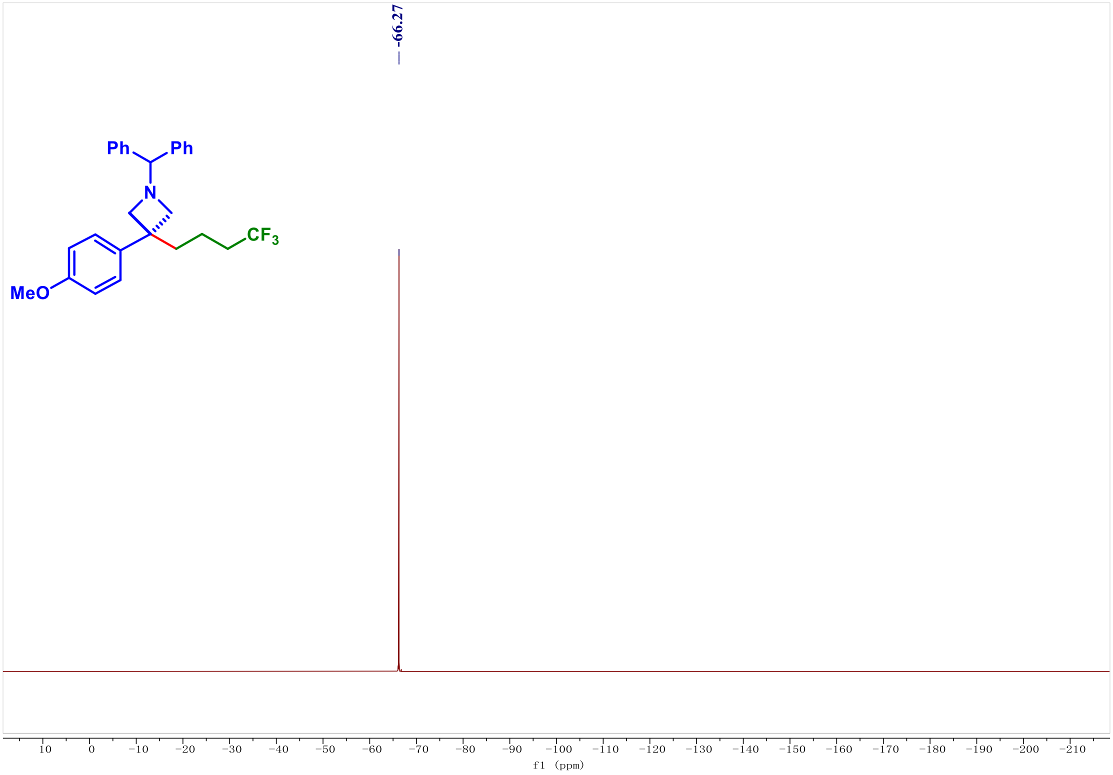

^{1}H NMR of Compound 13 (400 MHz, $CDCl_3$):

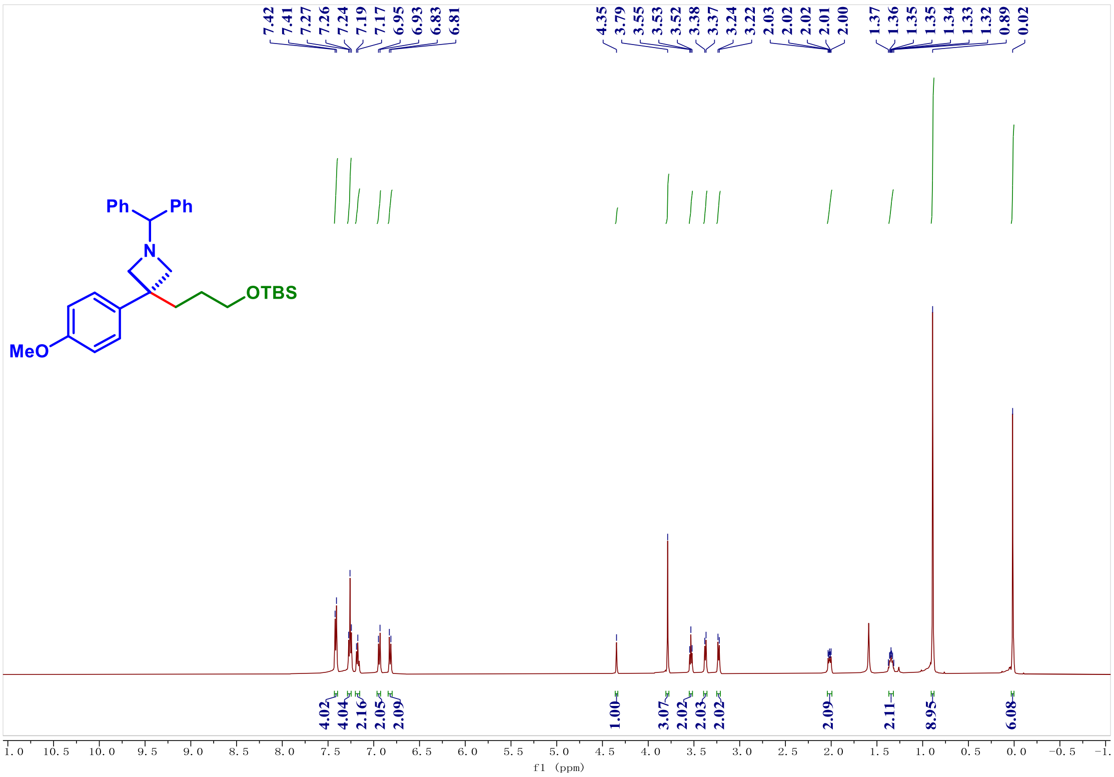

^{13}C NMR of Compound 13 (101 MHz, $CDCl_3$):

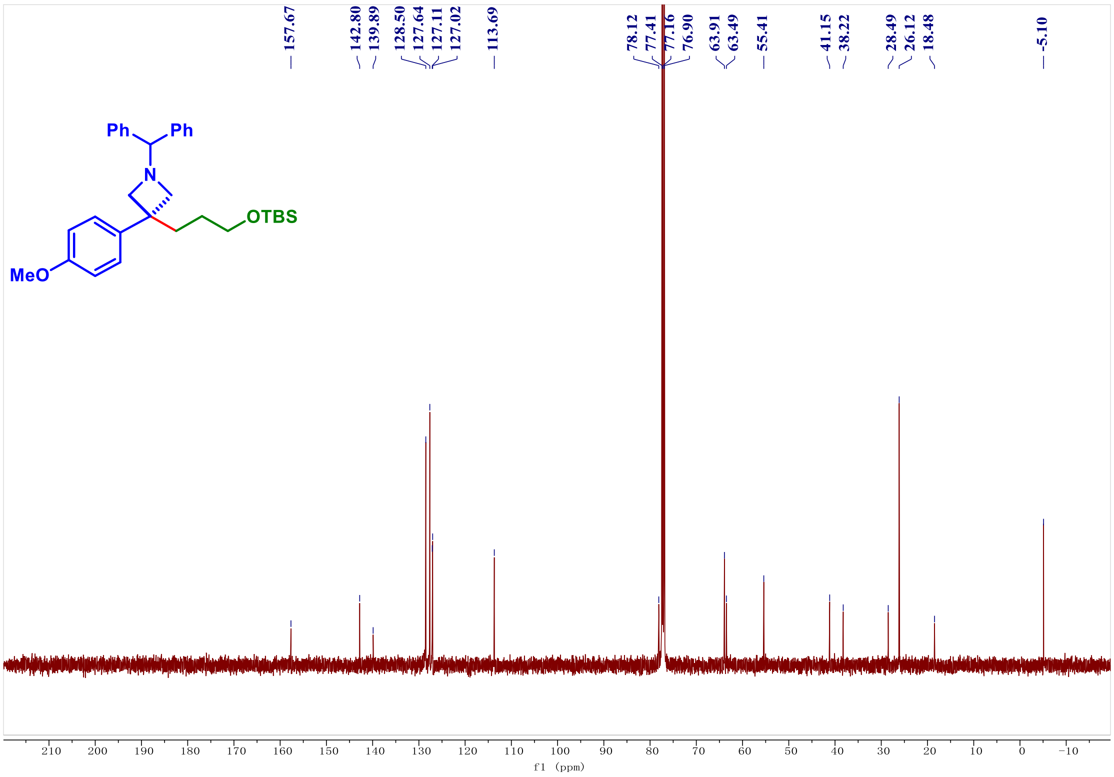

^{1}H NMR of Compound 14 (500 MHz, $CDCl_3$):

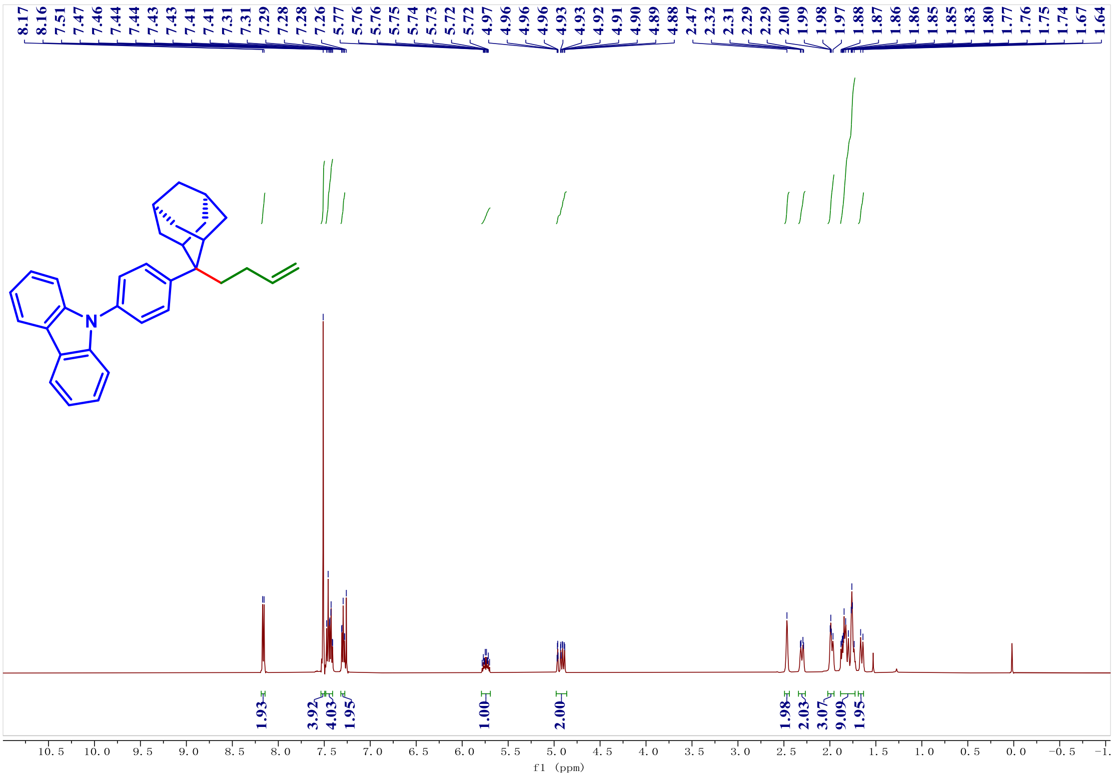

^{13}C NMR of Compound 14 (126 MHz, $CDCl_3$):

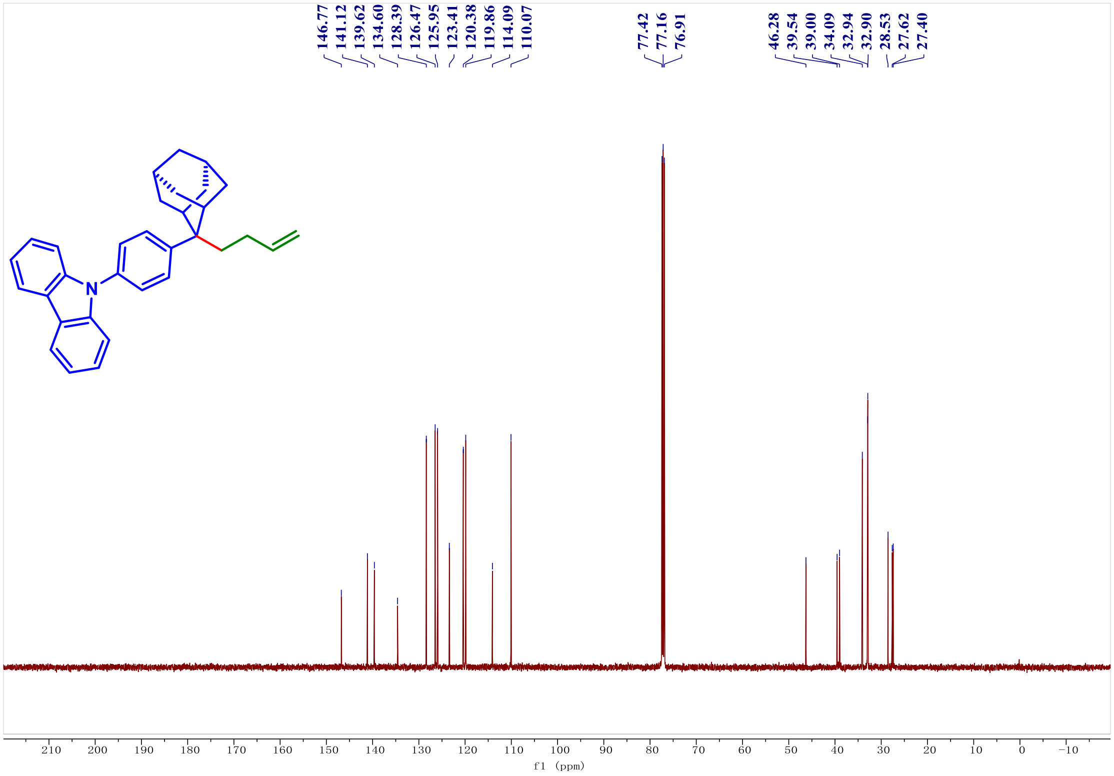

^{1}H NMR of Compound 15 (400 MHz, CDCl$_3$):

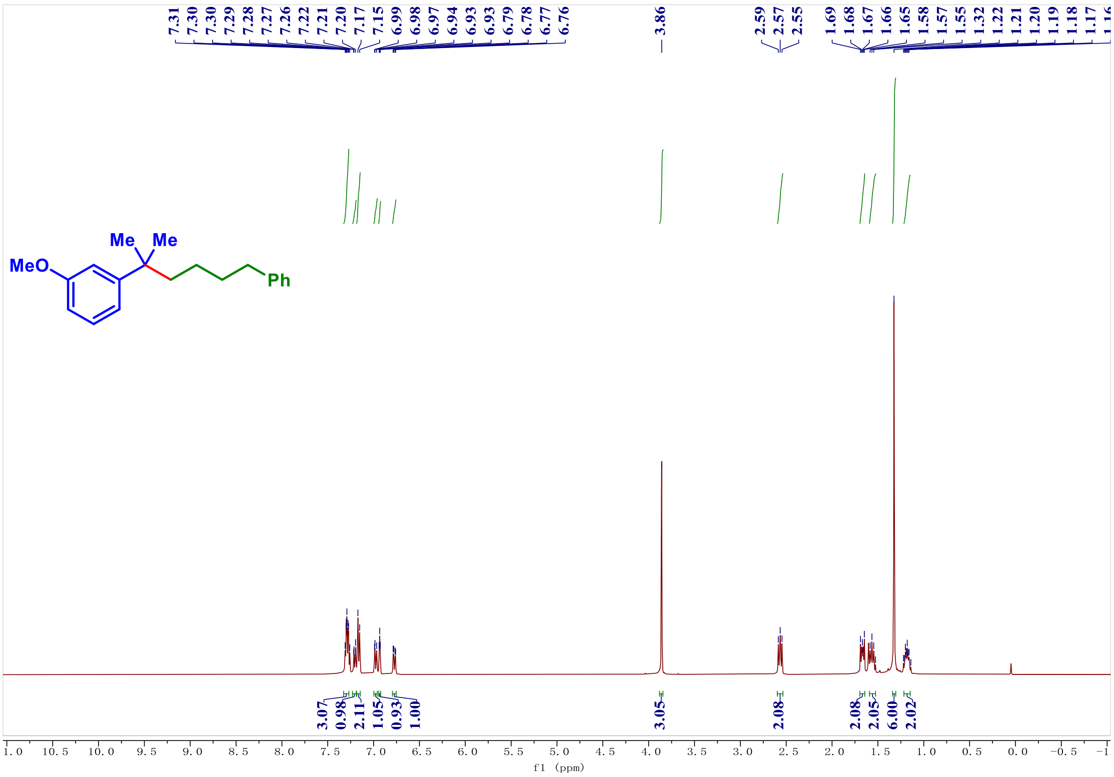

^{13}C NMR of Compound 15 (126 MHz, CDCl$_3$):

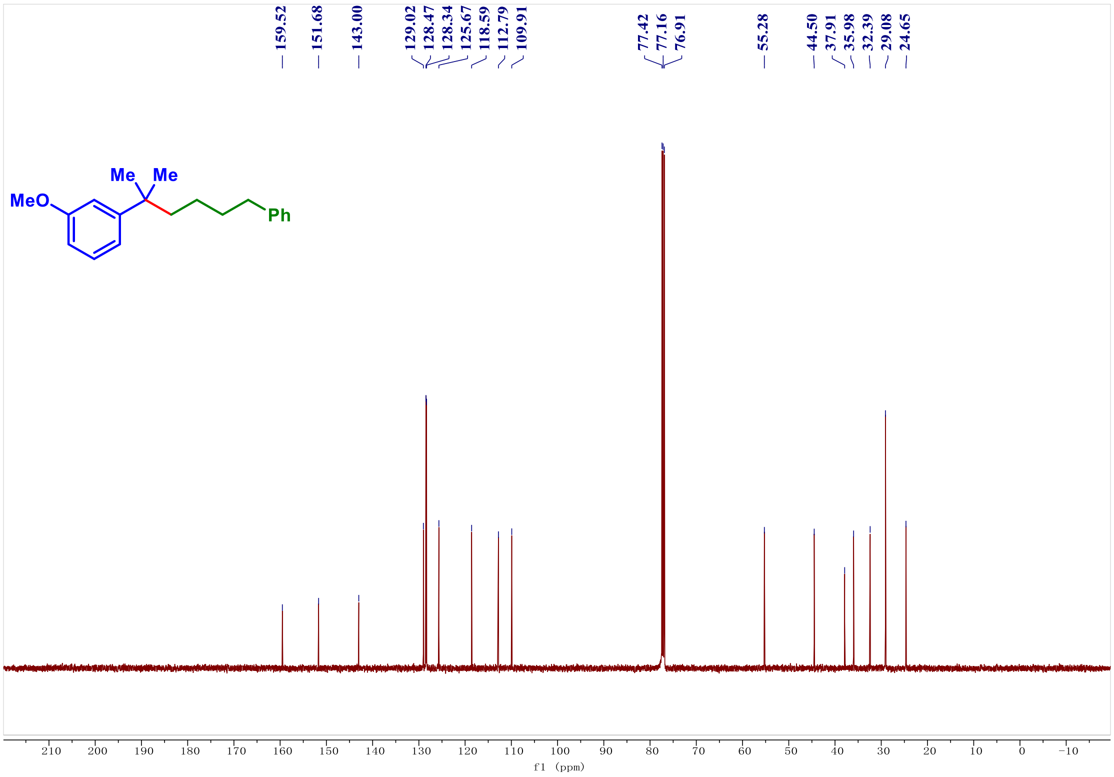

^{1}H NMR of Compound 16 (500 MHz, $CDCl_3$):

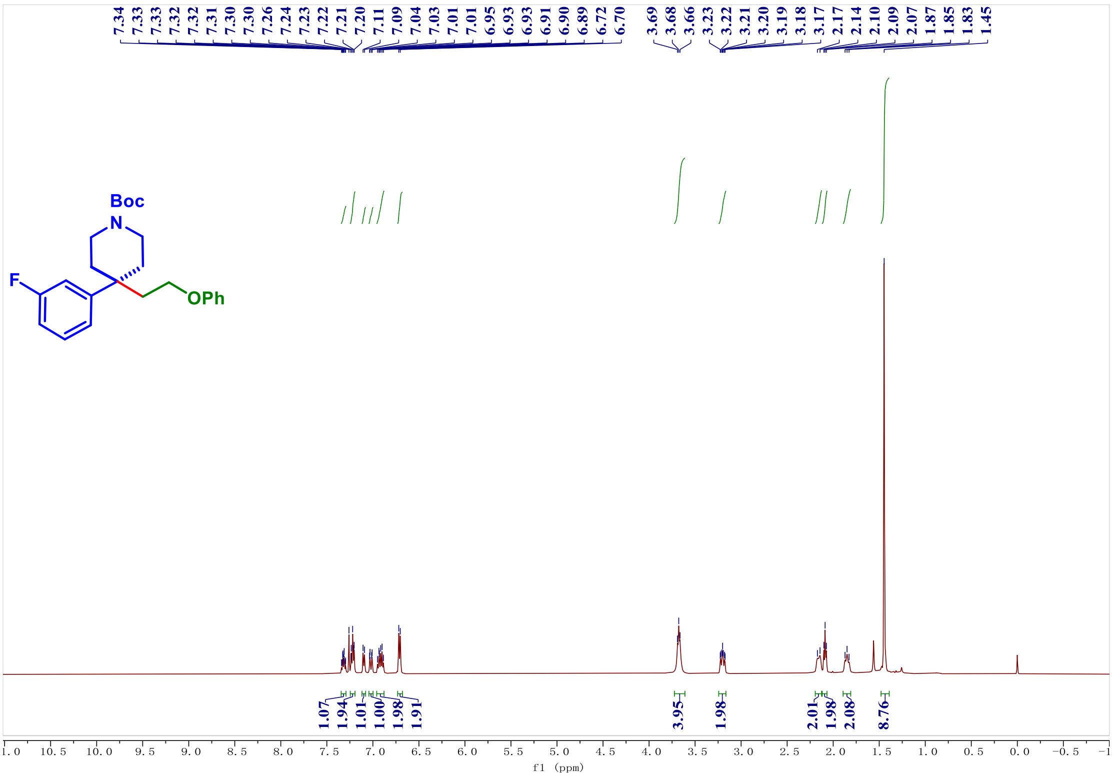

^{13}C NMR of Compound 16 (101 MHz, $CDCl_3$):

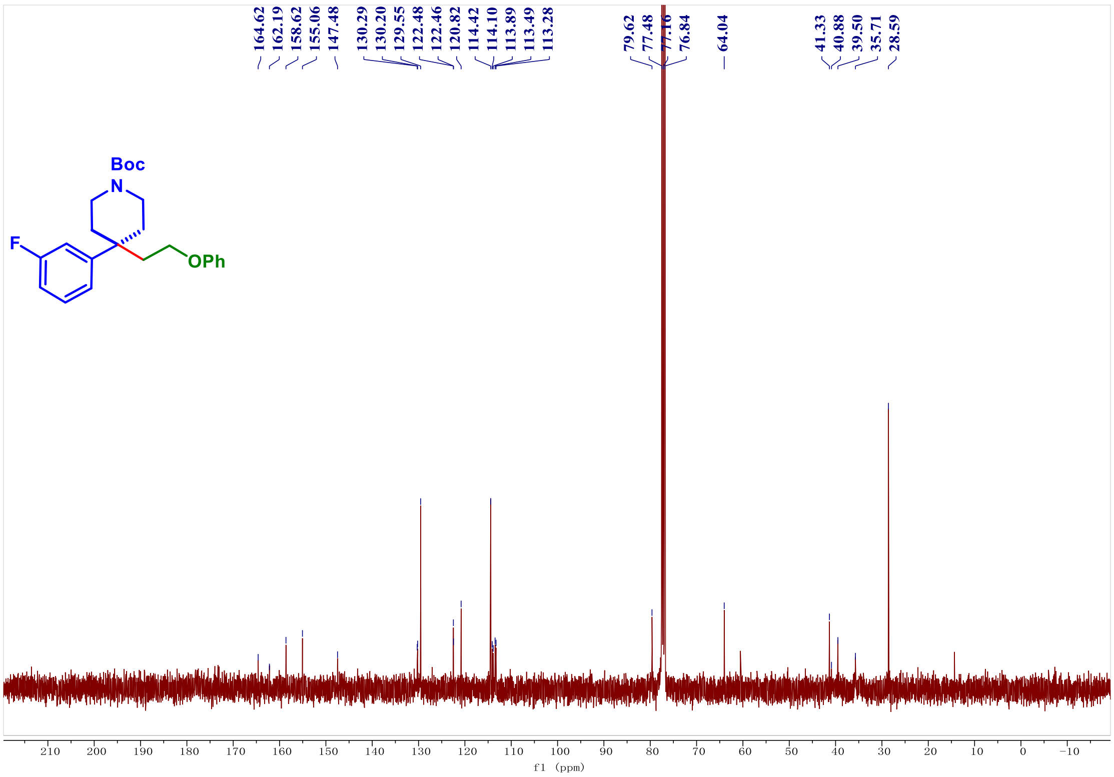

^{19}F NMR of Compound 16 (376 MHz, CDCl$_3$):

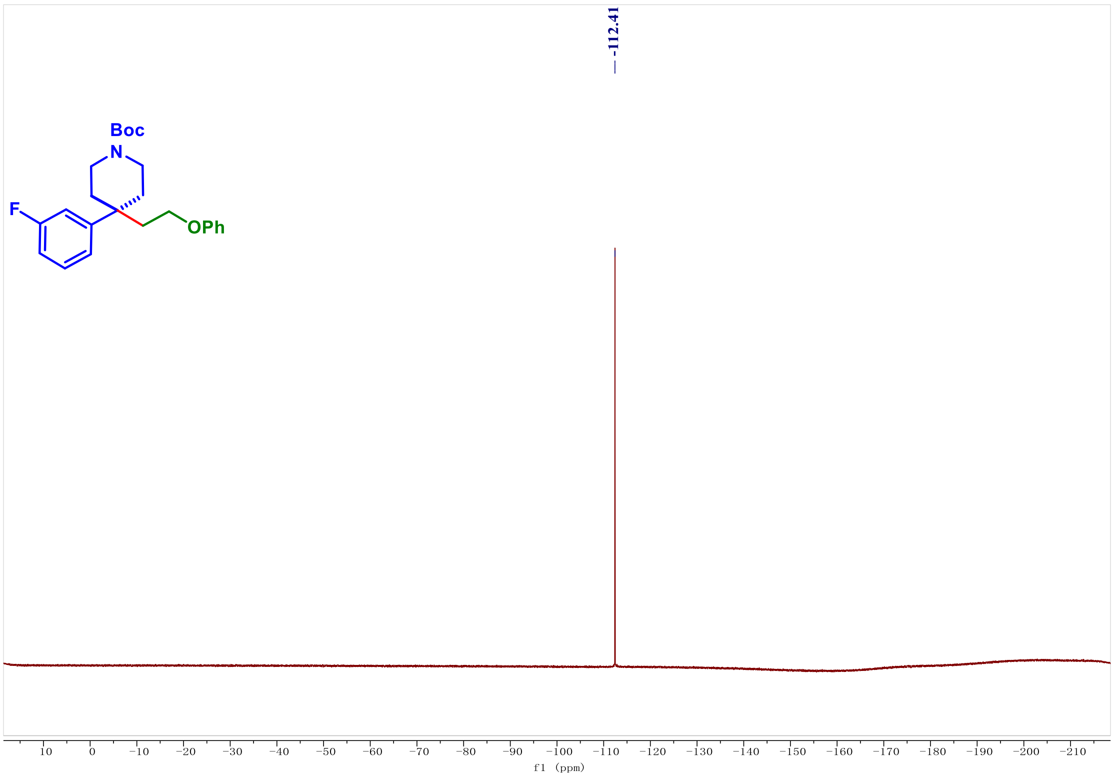

^{1}H NMR of Compound 17 (400 MHz, $CDCl_3$):

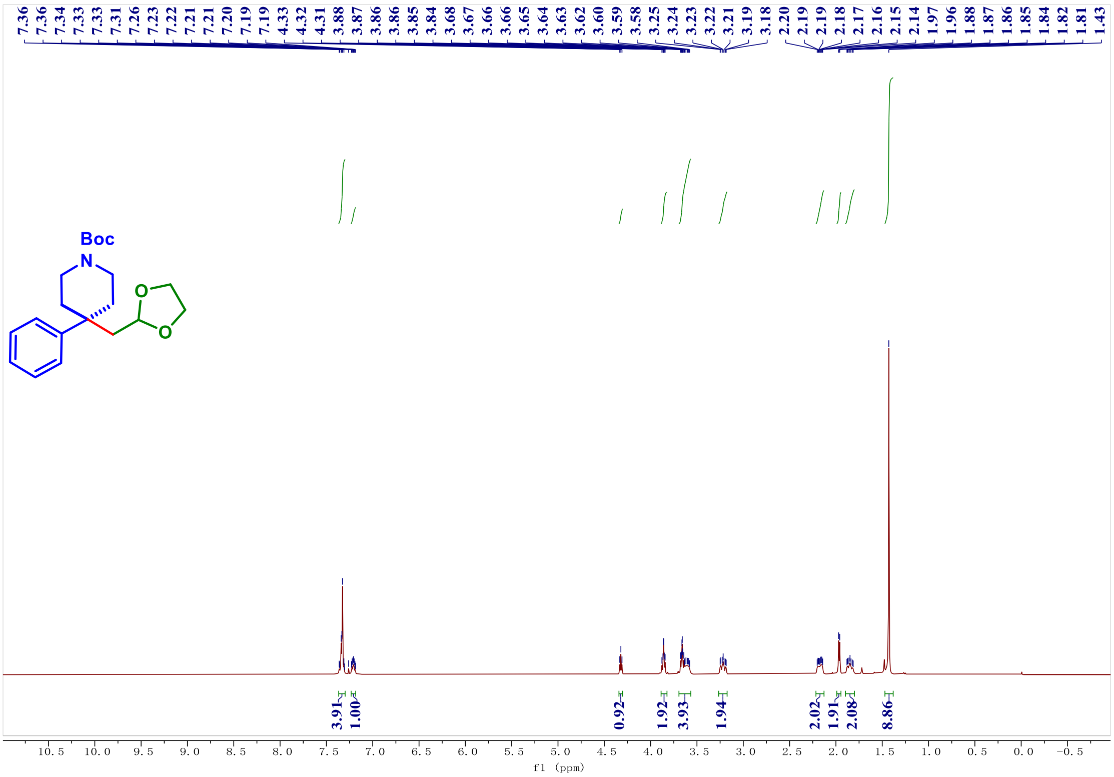

^{13}C NMR of Compound 17 (126 MHz, $CDCl_3$):

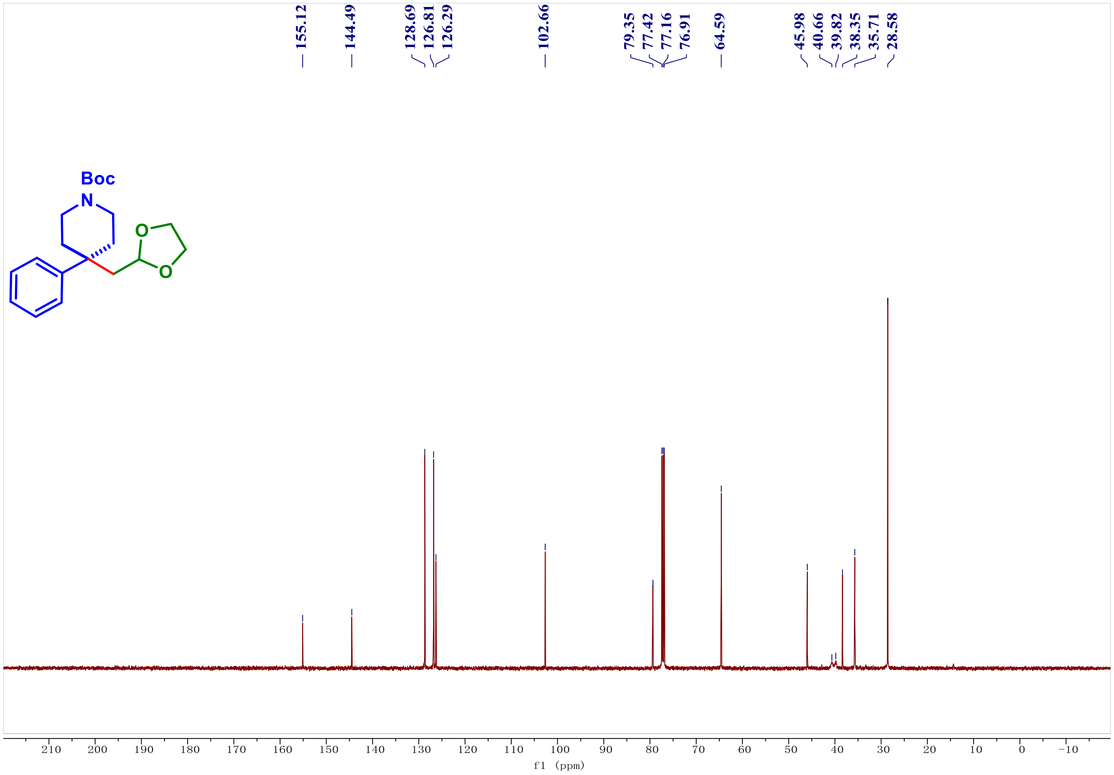

^{1}H NMR of Compound 18 (400 MHz, $CDCl_3$):

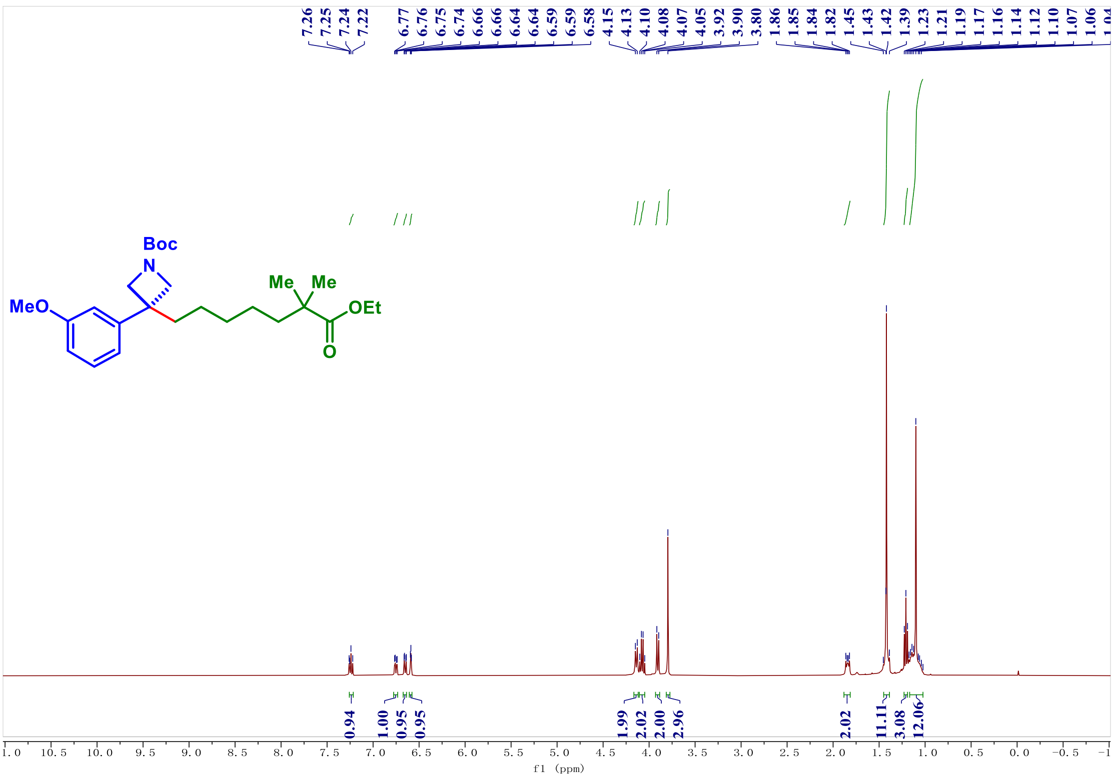

^{13}C NMR of Compound 18 (126 MHz, $CDCl_3$):

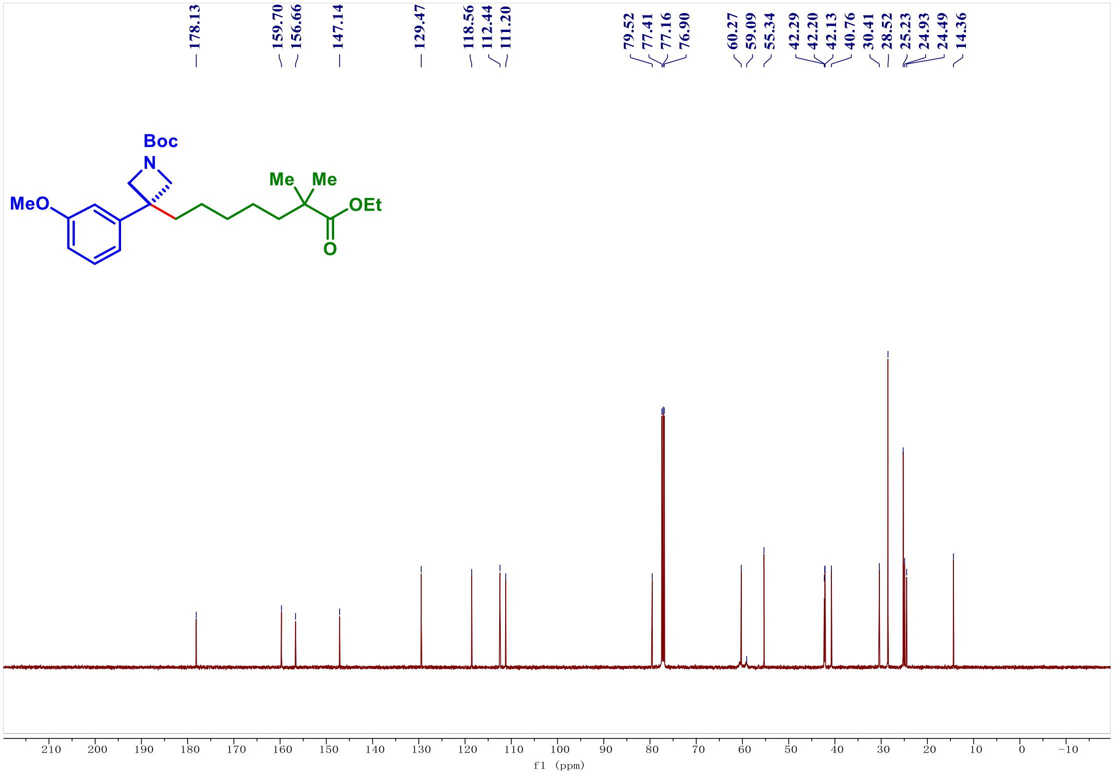

^{1}H NMR of Compound 19 (500 MHz, $CDCl_3$):

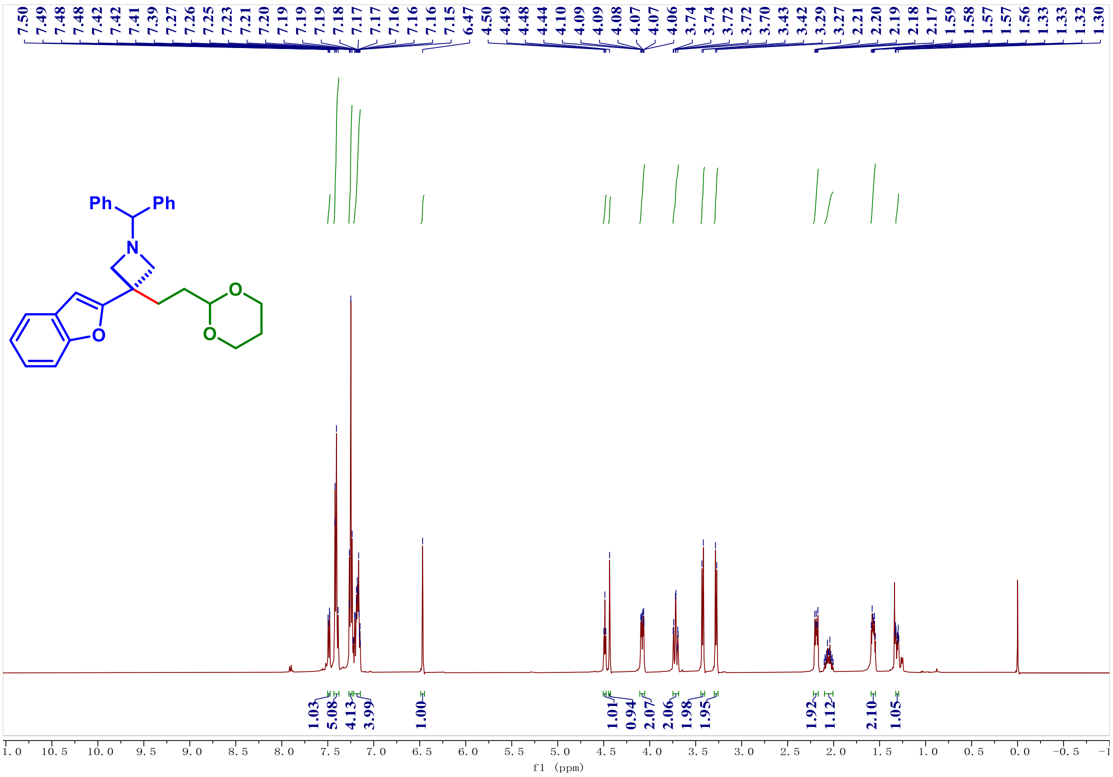

^{13}C NMR of Compound 19 (126 MHz, $CDCl_3$):

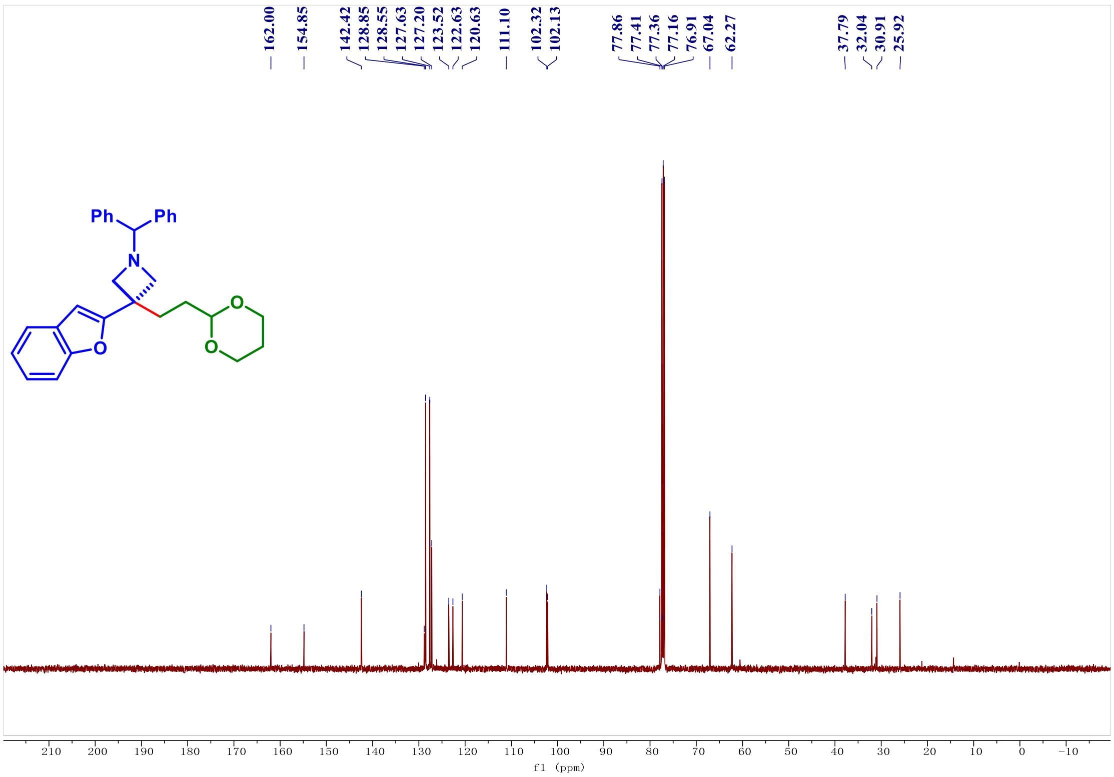

^{1}H NMR of Compound 20 (500 MHz, $CDCl_3$):

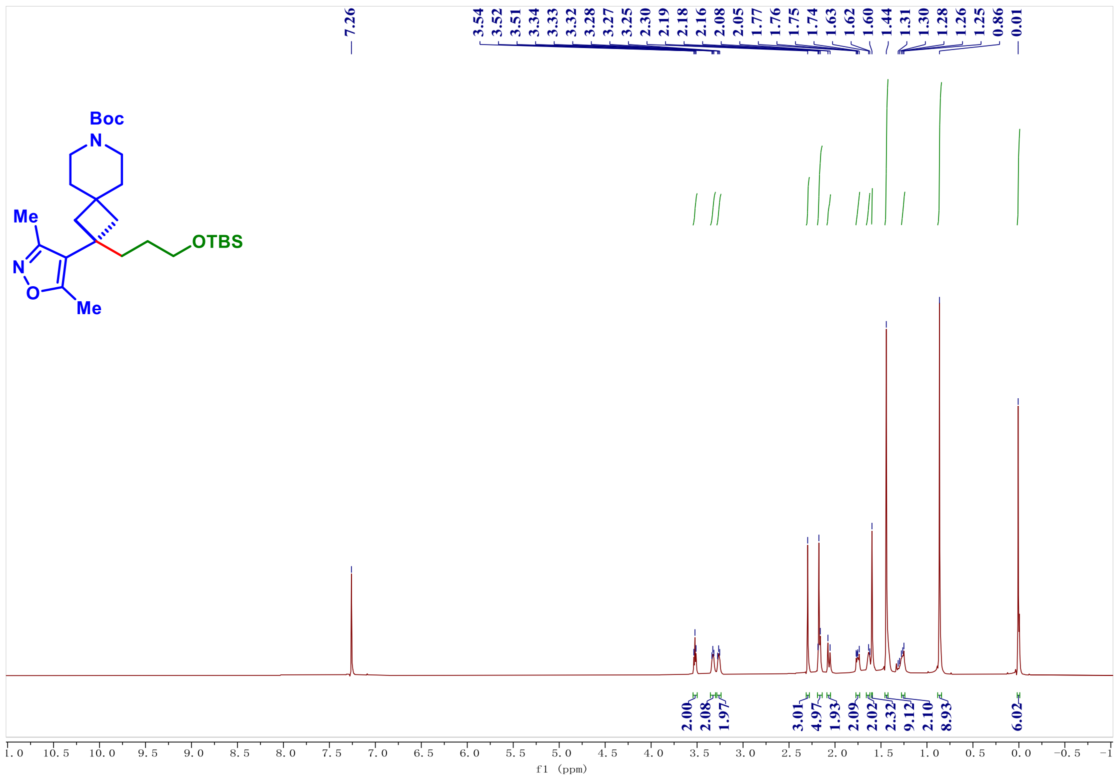

^{13}C NMR of Compound 20 (126 MHz, $CDCl_3$):

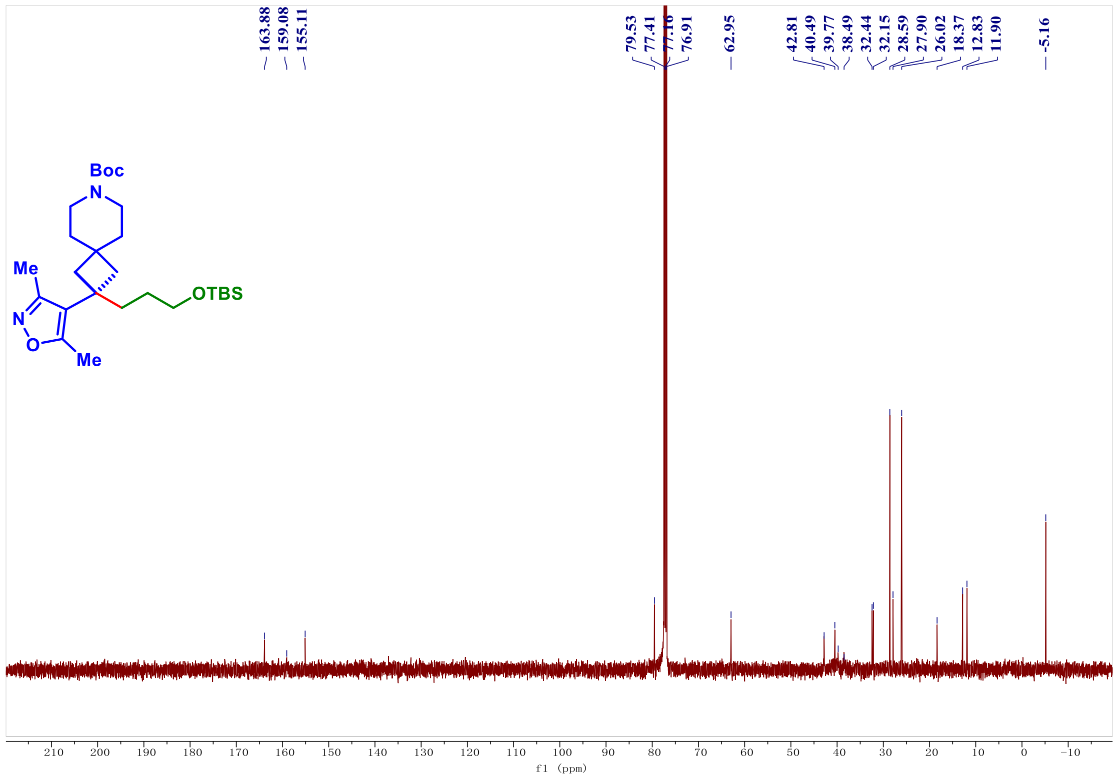

^{1}H NMR of Compound 21 (400 MHz, $CDCl_3$):

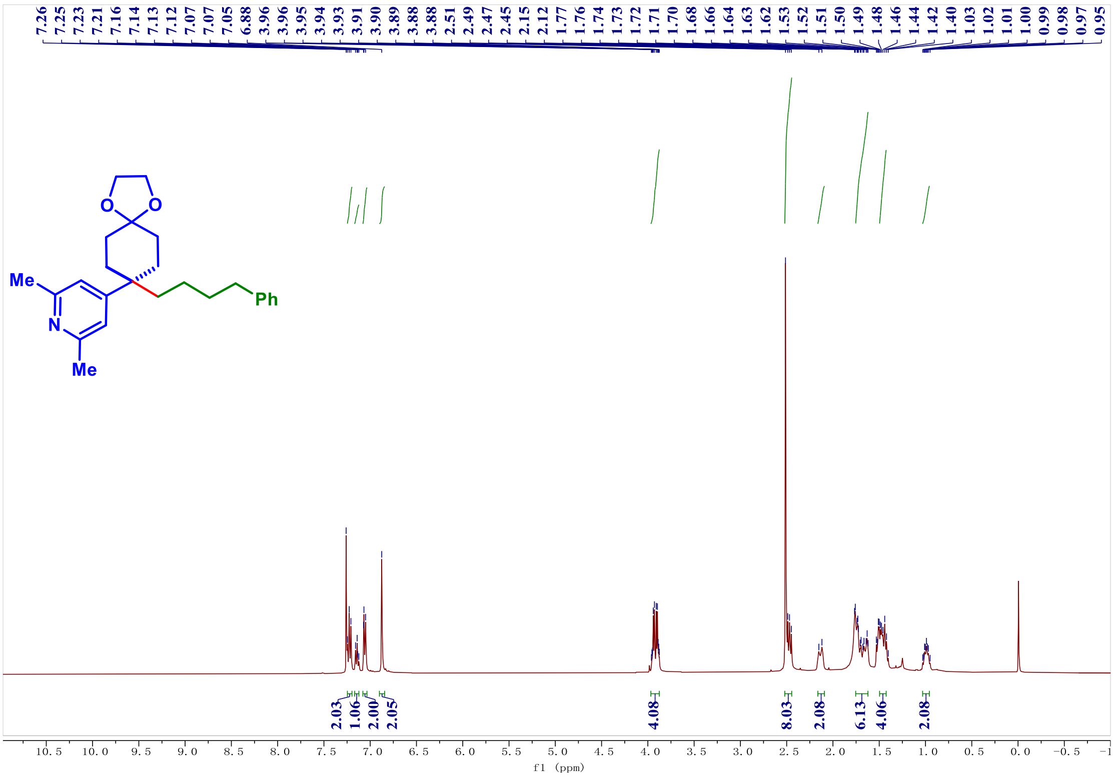

^{13}C NMR of Compound 21 (101 MHz, $CDCl_3$):

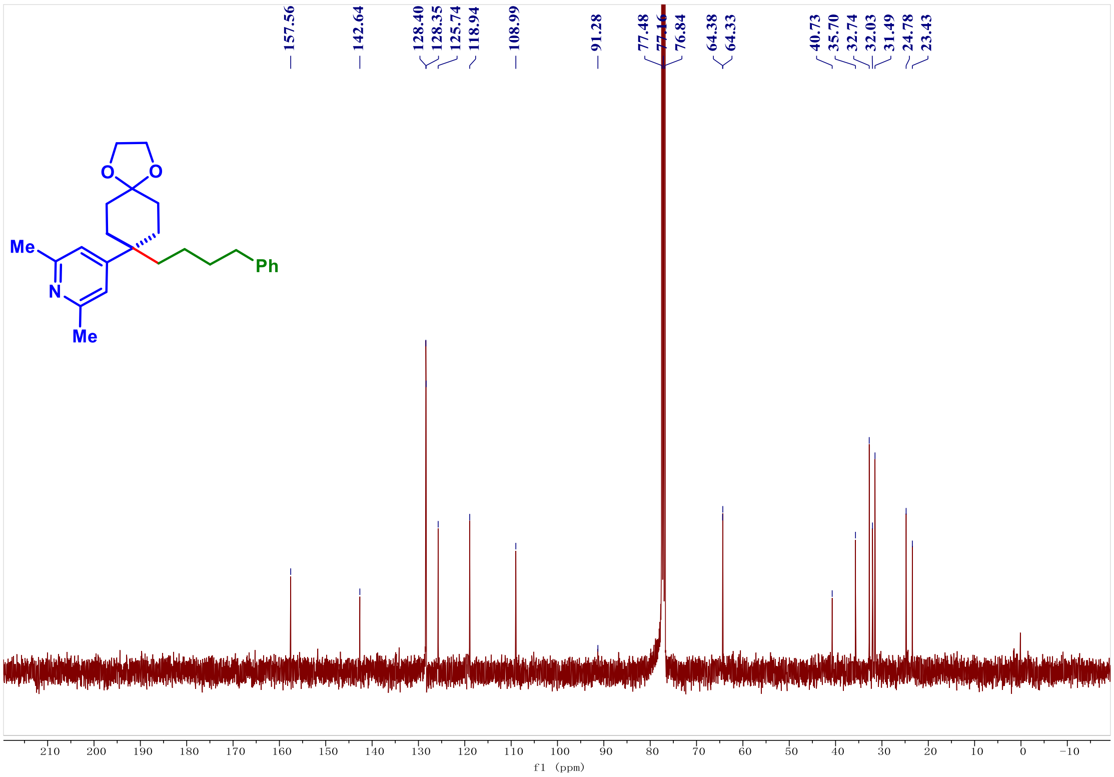

^{1}H NMR of Compound 22 (500 MHz, $CDCl_3$):

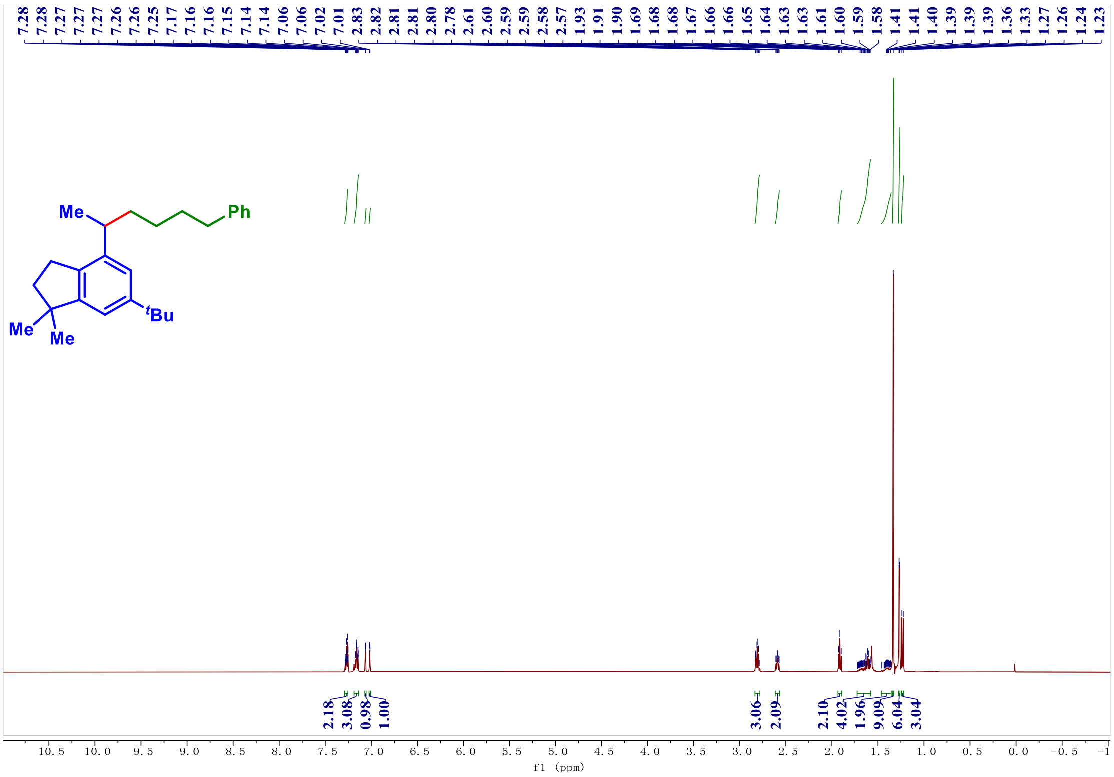

^{13}C NMR of Compound 22 (126 MHz, $CDCl_3$):

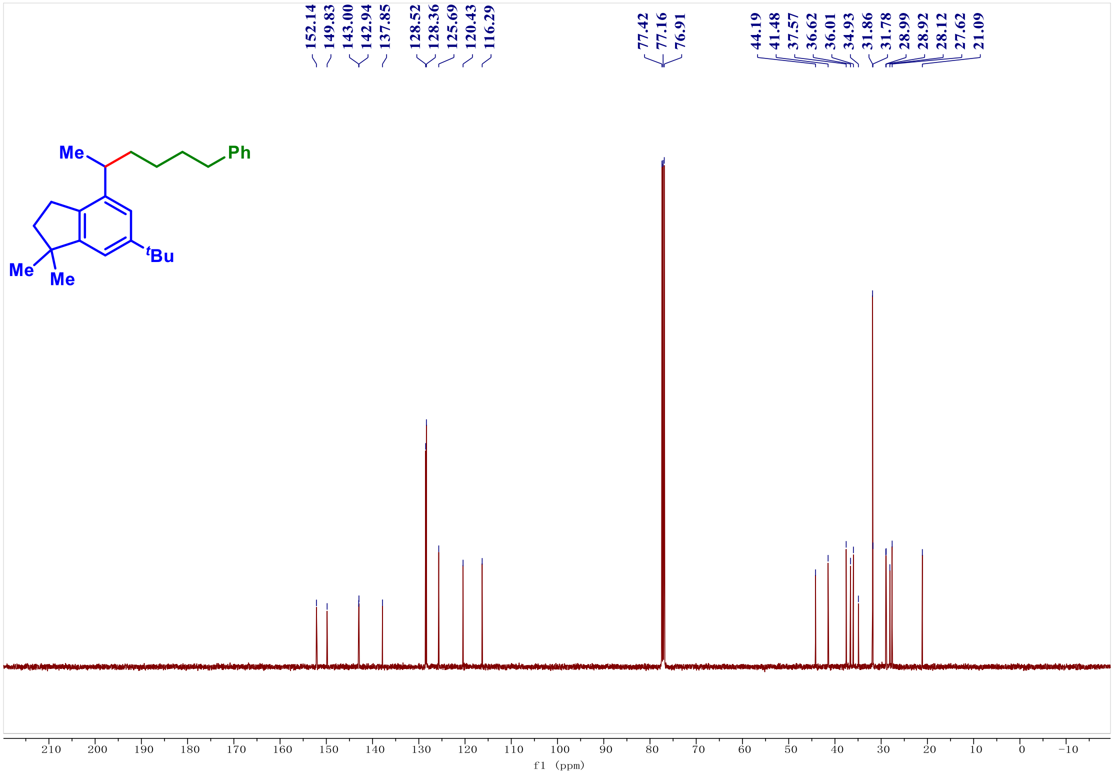

^{1}H NMR of Compound 23 (500 MHz, $CDCl_3$):

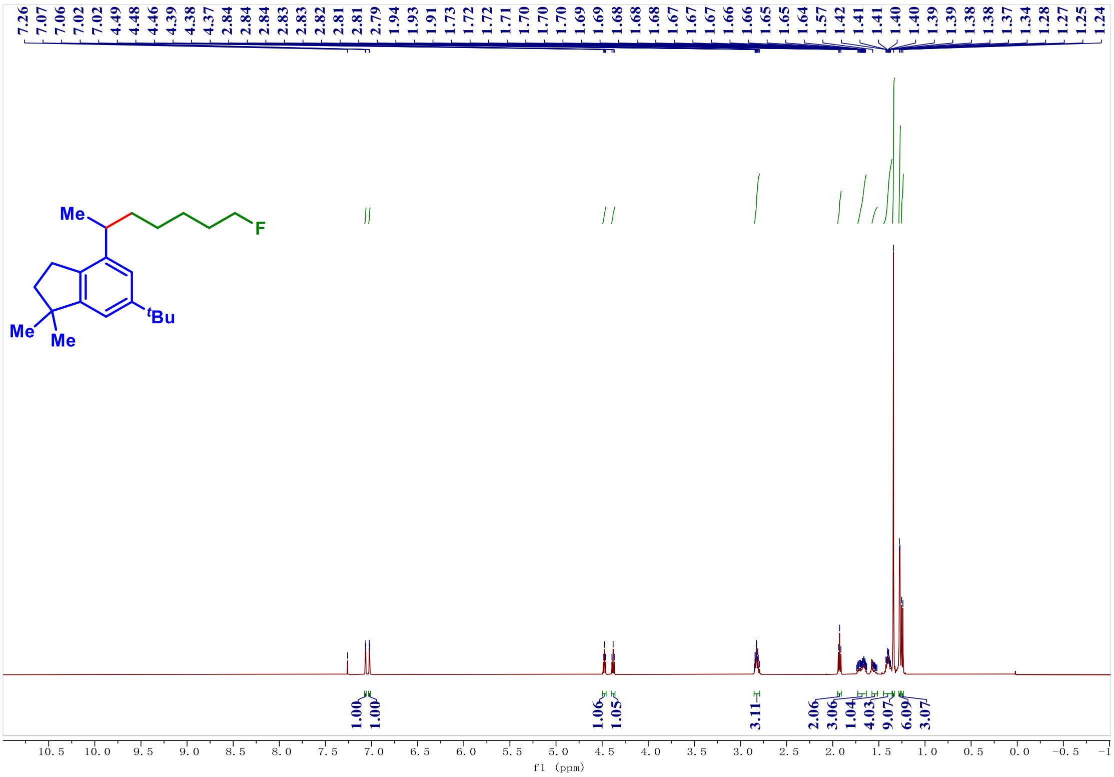

^{13}C NMR of Compound 23 (126 MHz, $CDCl_3$):

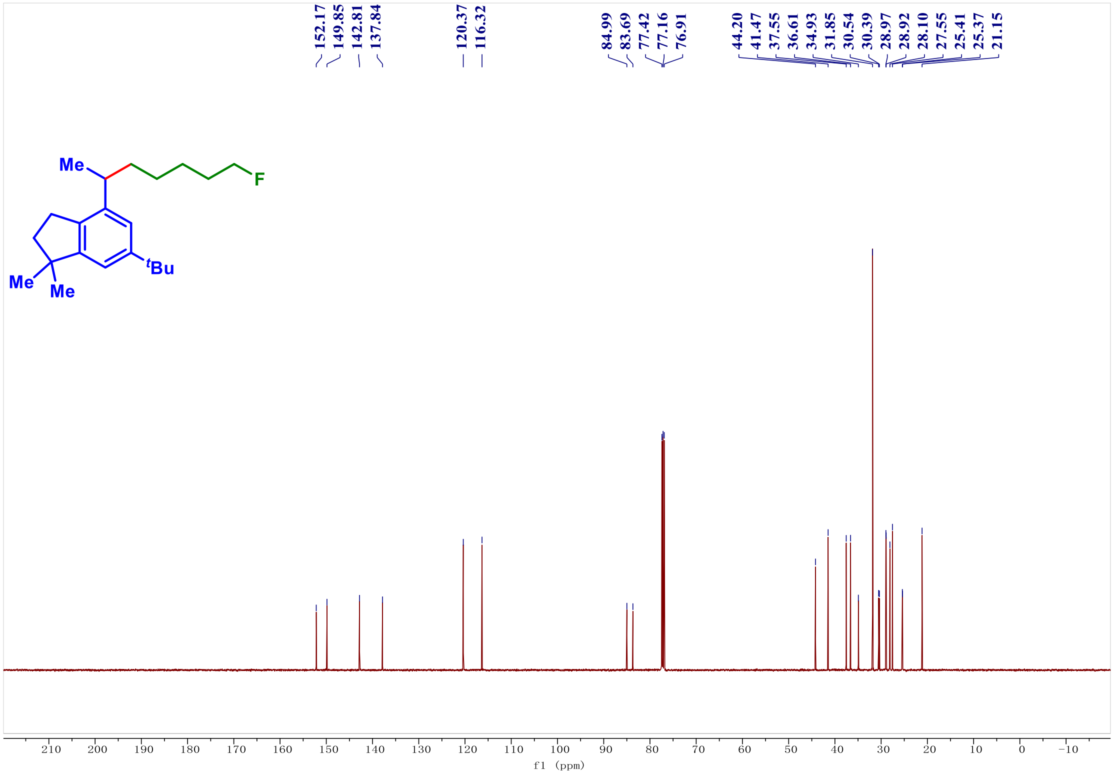

^{19}F NMR of Compound 23 (376 MHz, $CDCl_3$):

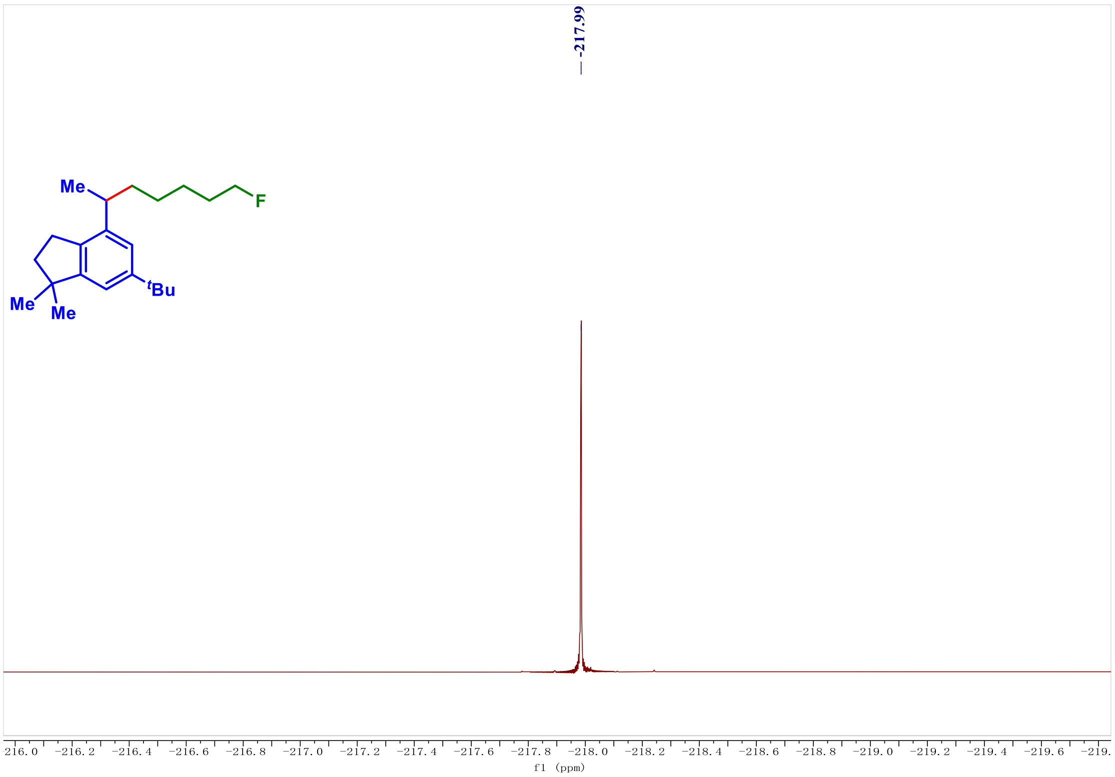

^{1}H NMR of Compound 24 (400 MHz, $CDCl_3$):

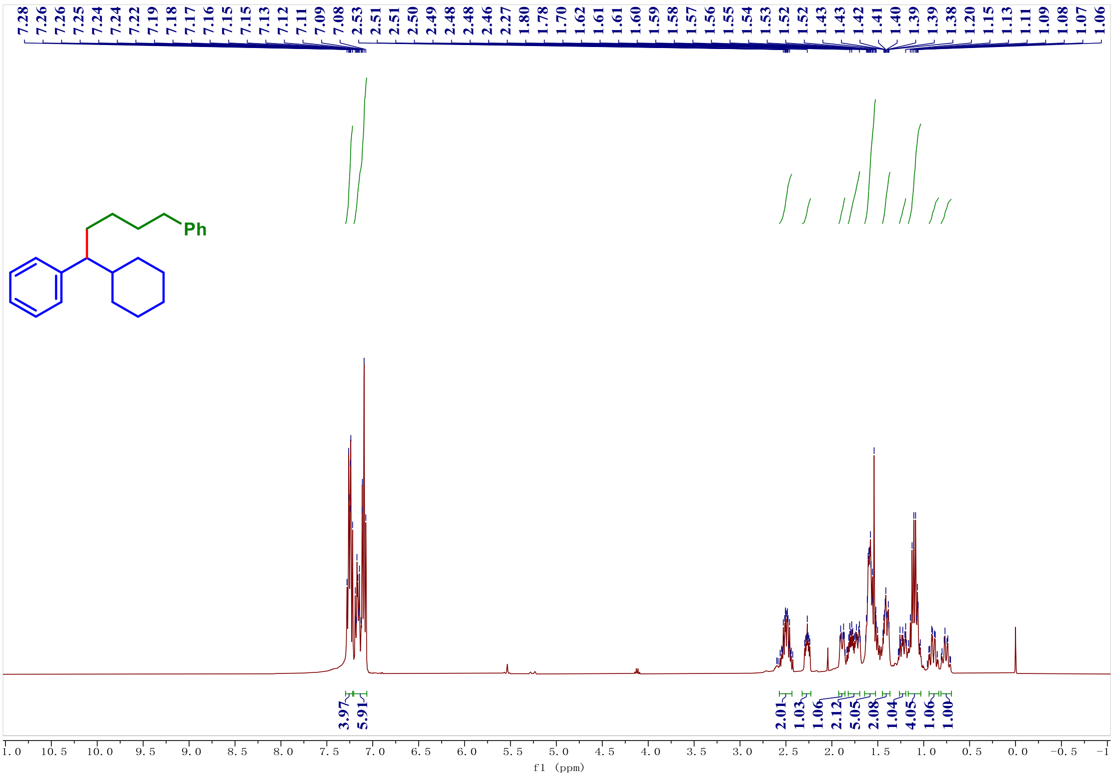

^{13}C NMR of Compound 24 (101 MHz, $CDCl_3$):

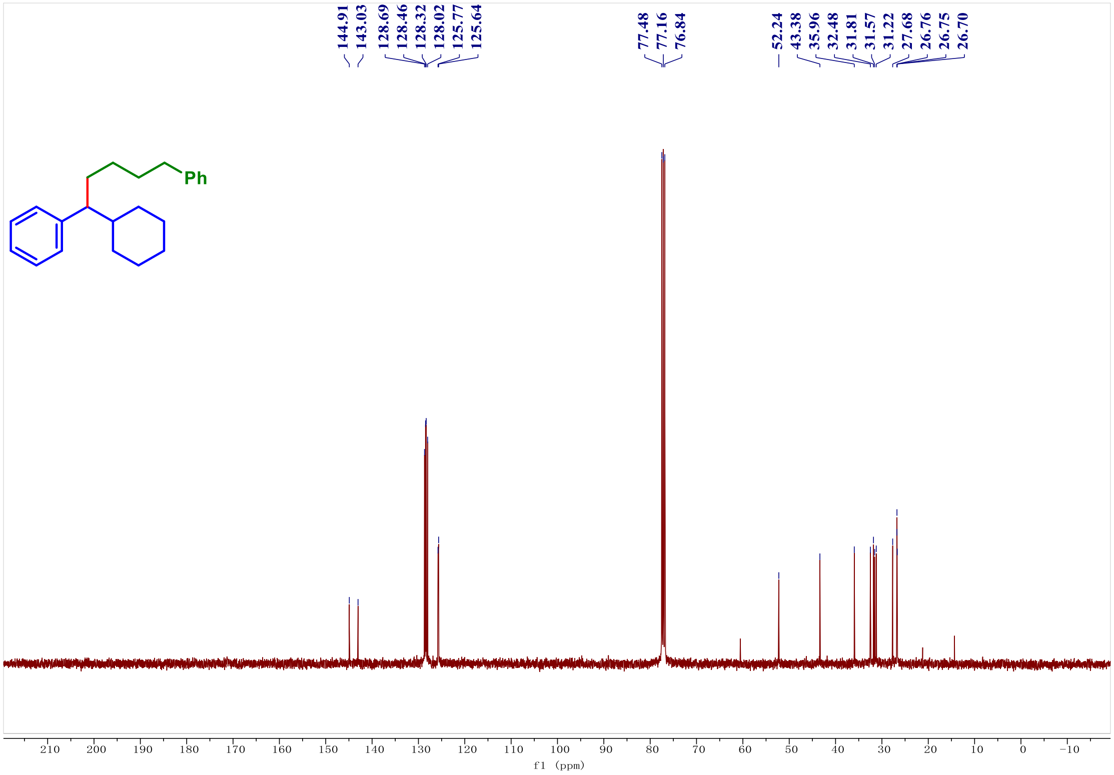

^{1}H NMR of Compound 25 (500 MHz, $CDCl_3$):

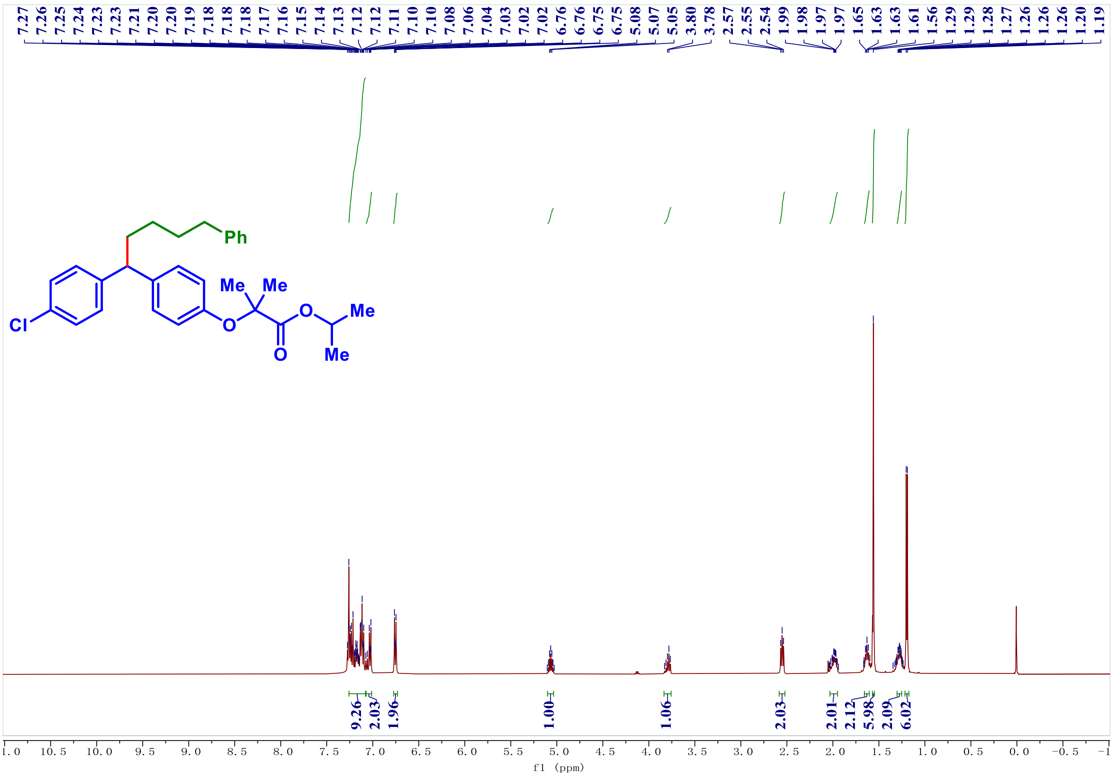

^{13}C NMR of Compound 25 (126 MHz, $CDCl_3$):

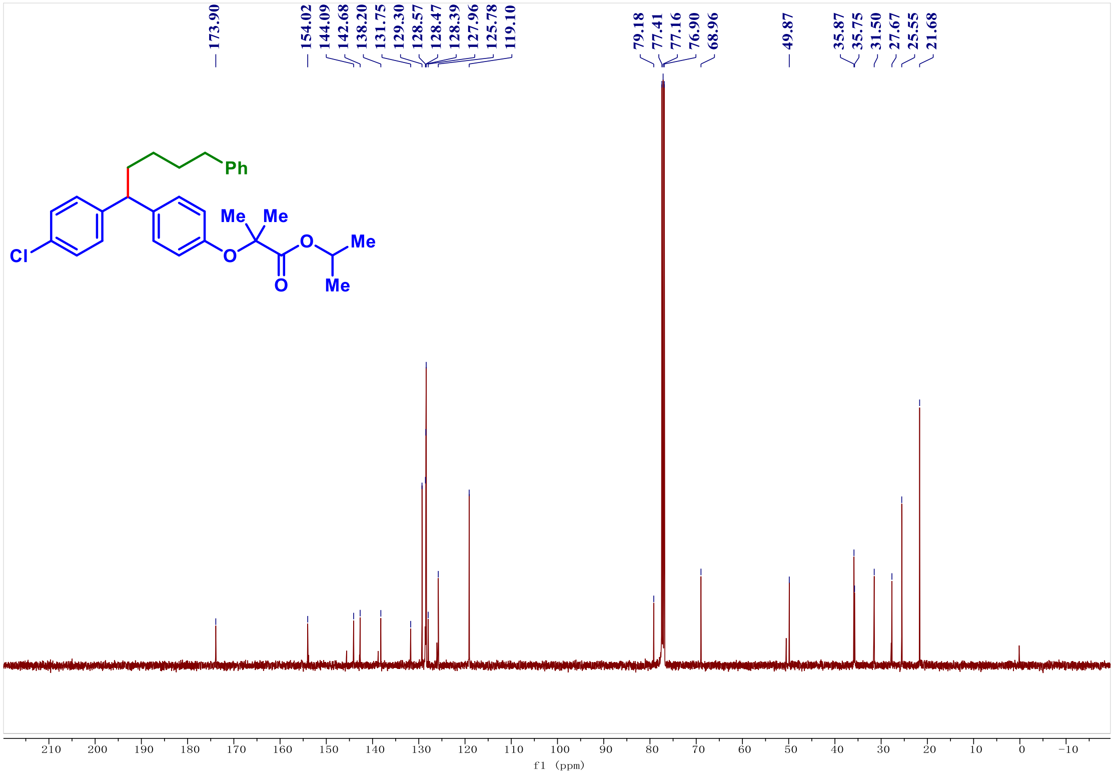

1H NMR of Compound 26 (400 MHz, $CDCl_3$):

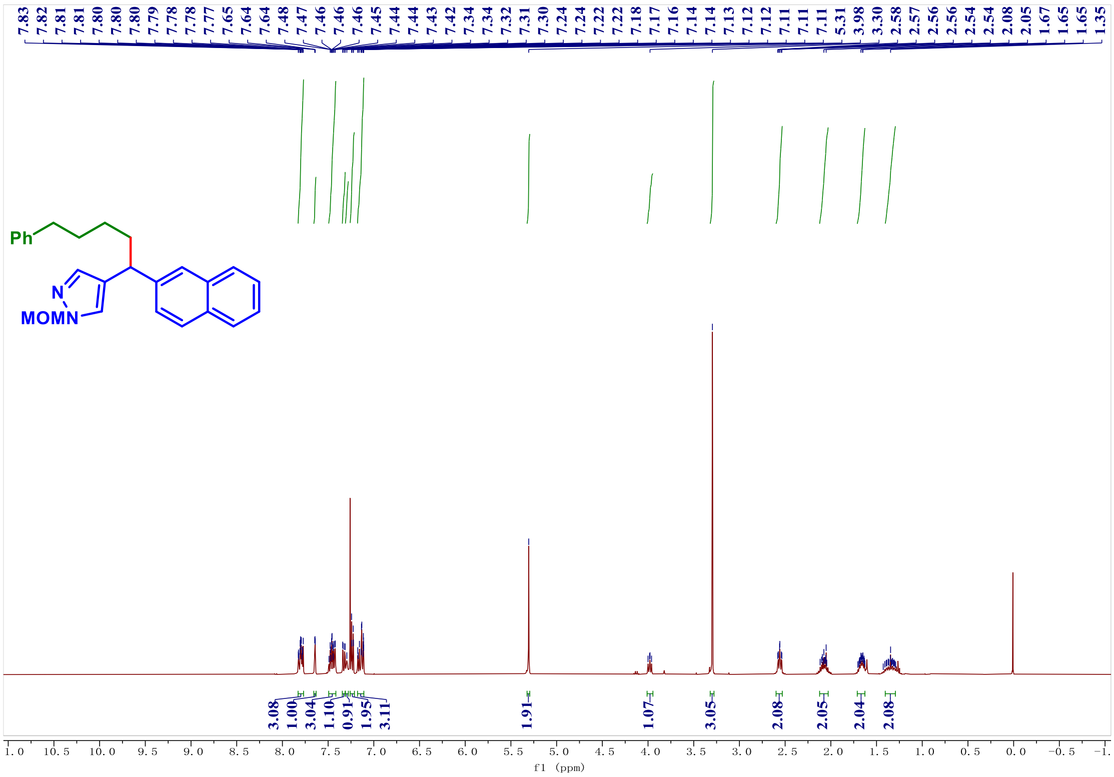

13C NMR of Compound 26 (101 MHz, $CDCl_3$):

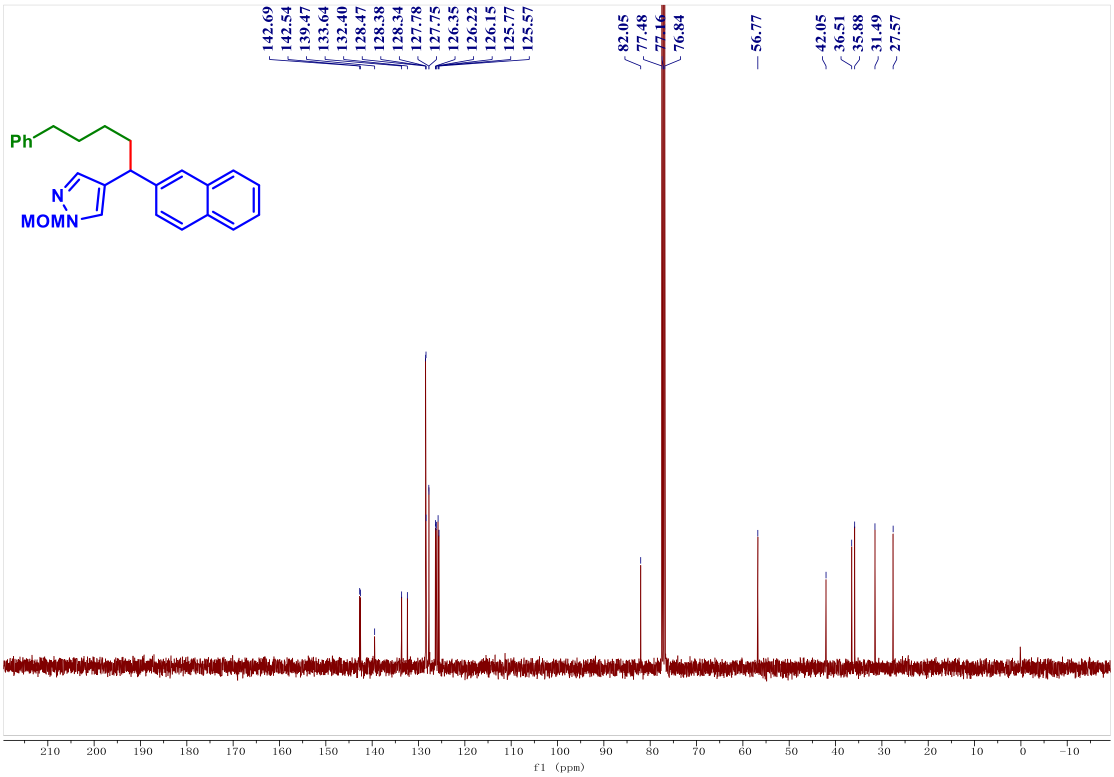

^{1}H NMR of Compound 27 (400 MHz, $CDCl_3$):

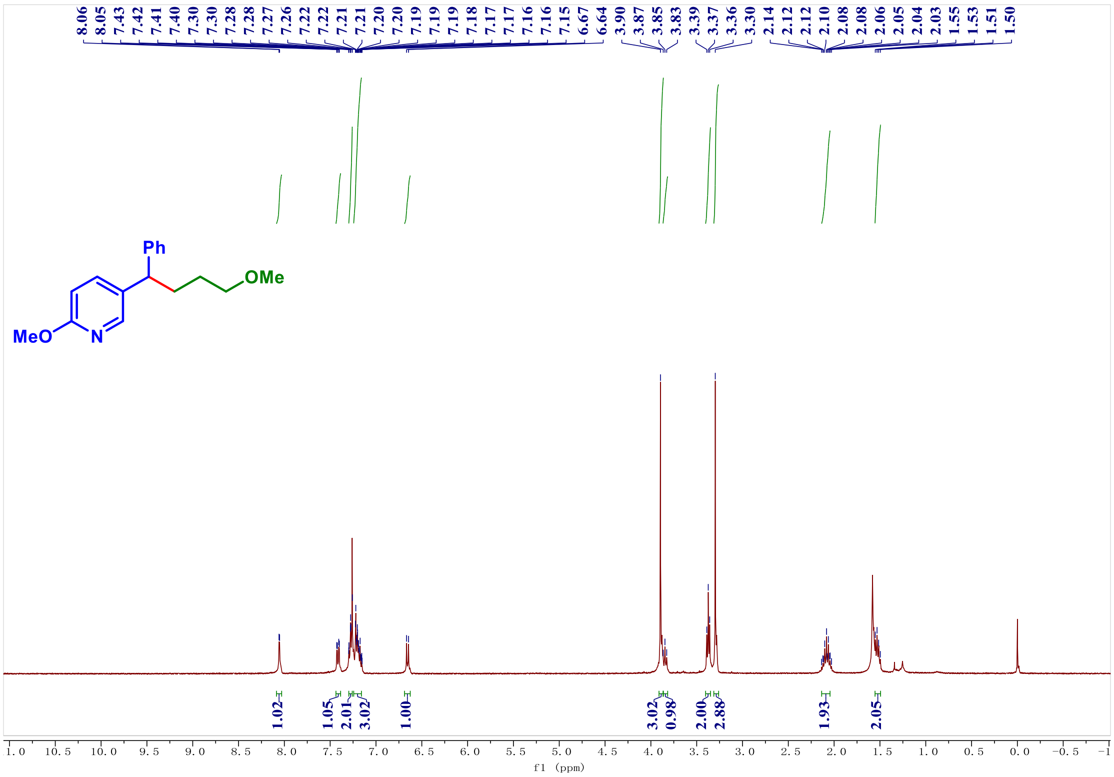

^{13}C NMR of Compound 27 (126 MHz, $CDCl_3$):

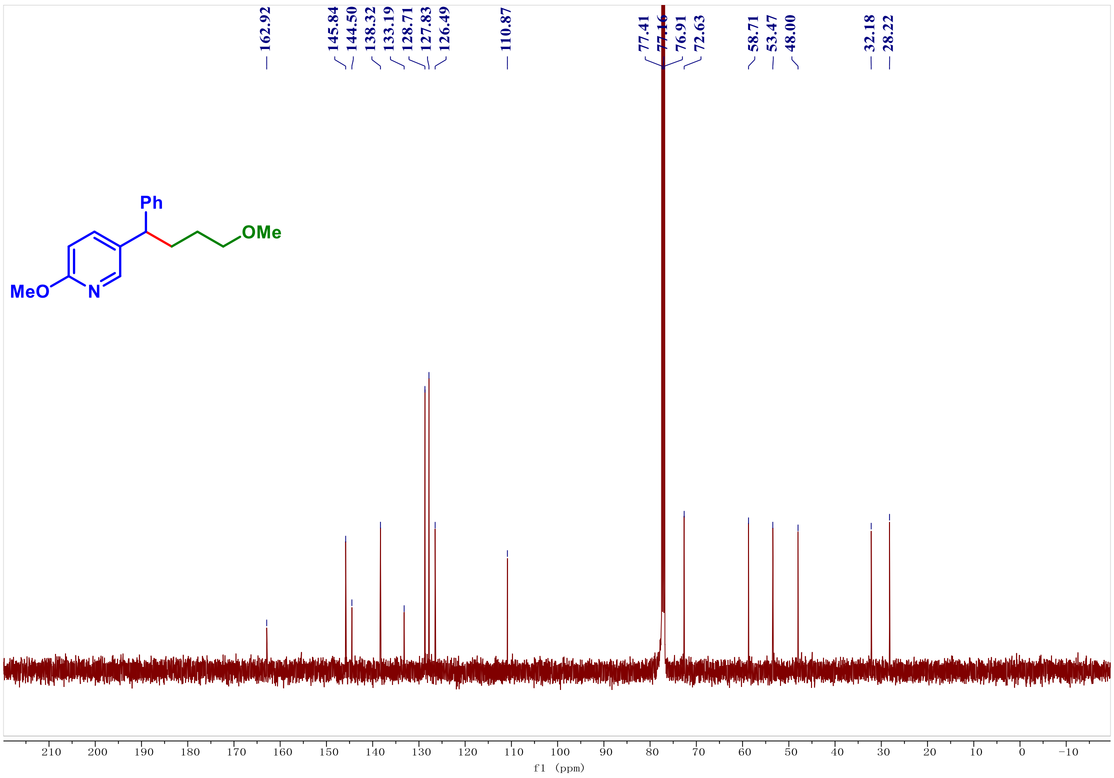

^{1}H NMR of Compound 28 (500 MHz, $CDCl_3$):

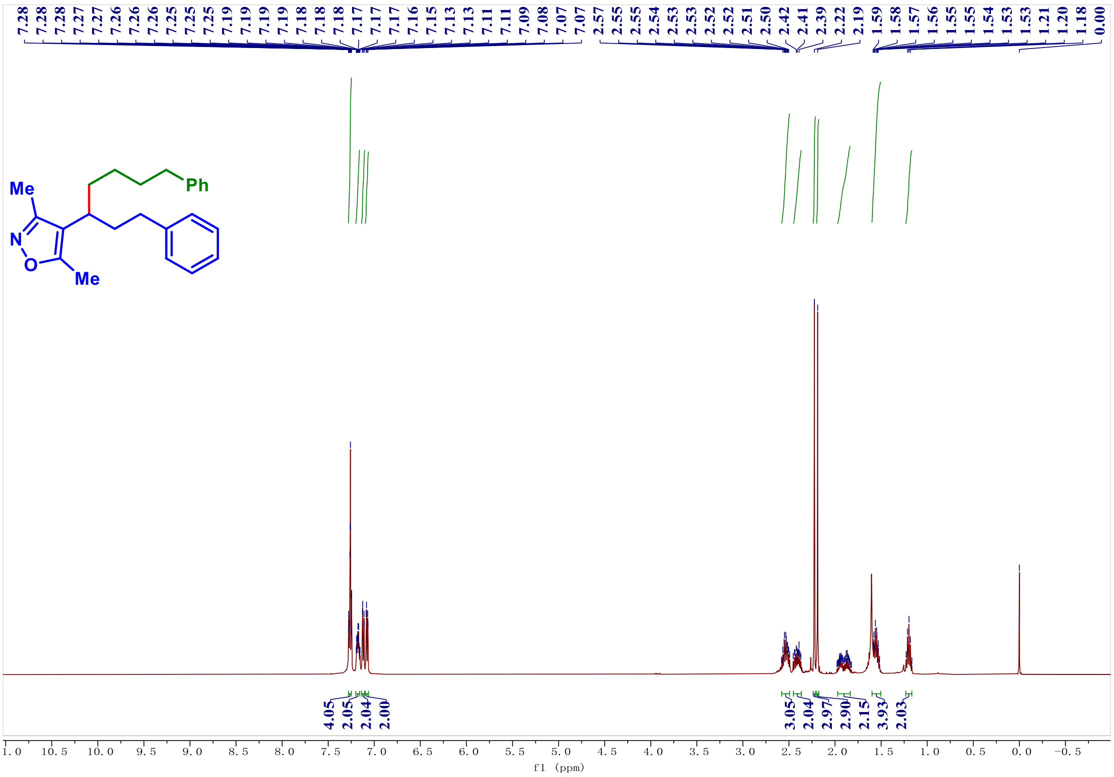

^{13}C NMR of Compound 28 (126 MHz, $CDCl_3$):

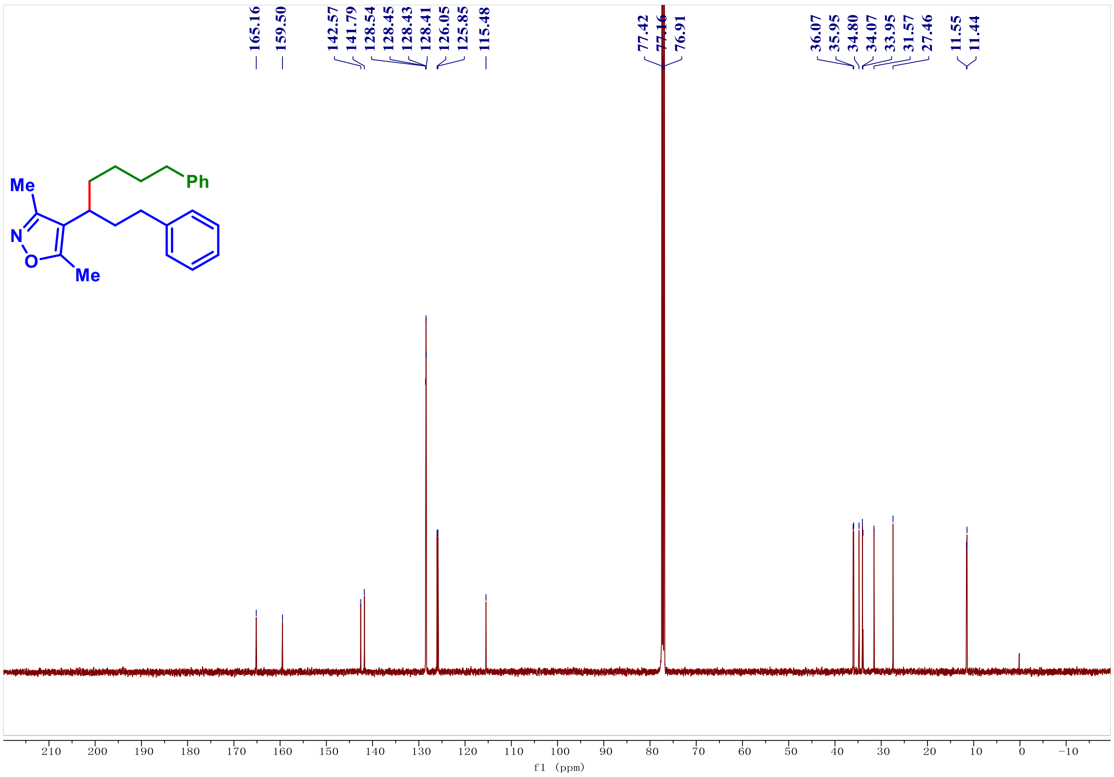

^{1}H NMR of Compound 29 (400 MHz, $CDCl_3$):

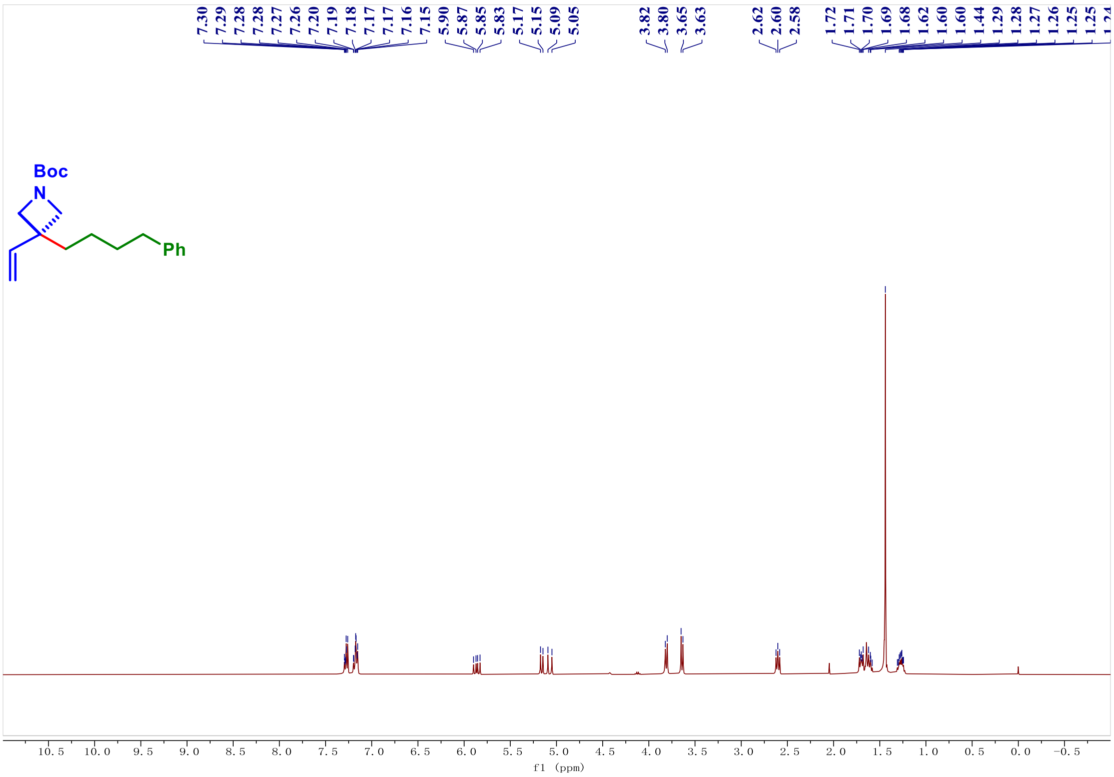

^{13}C NMR of Compound 29 (101 MHz, $CDCl_3$):

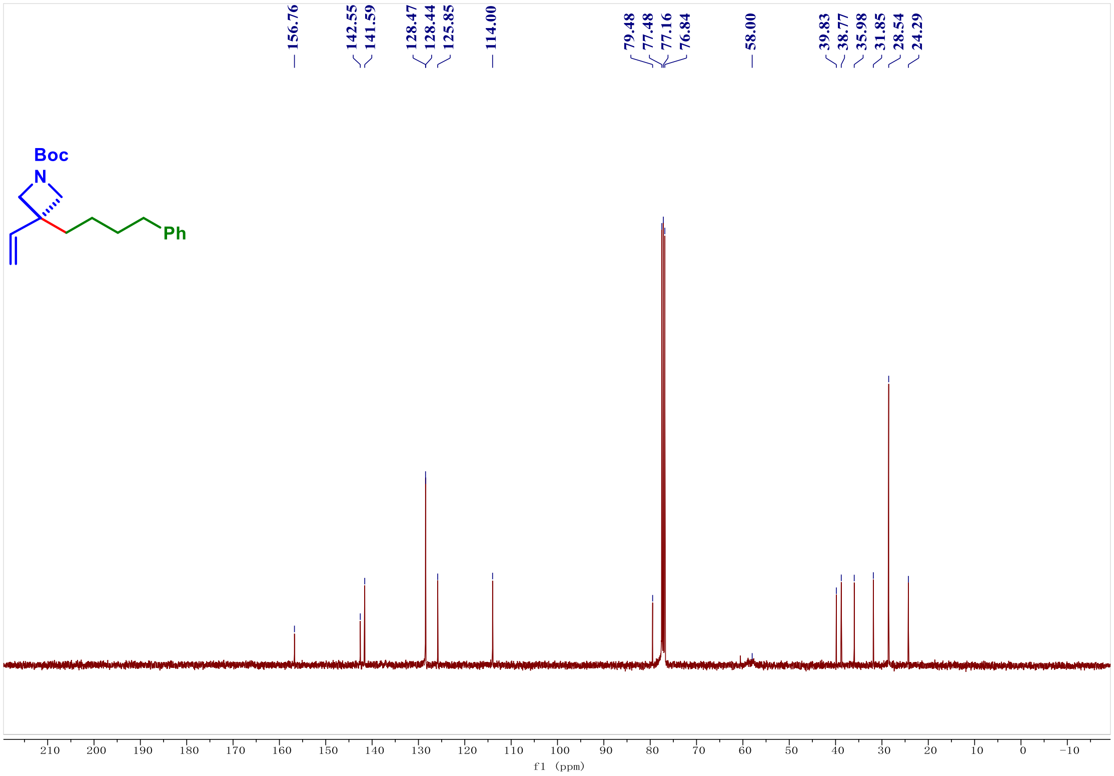

^{1}H NMR of Compound 30 (500 MHz, $CDCl_3$):

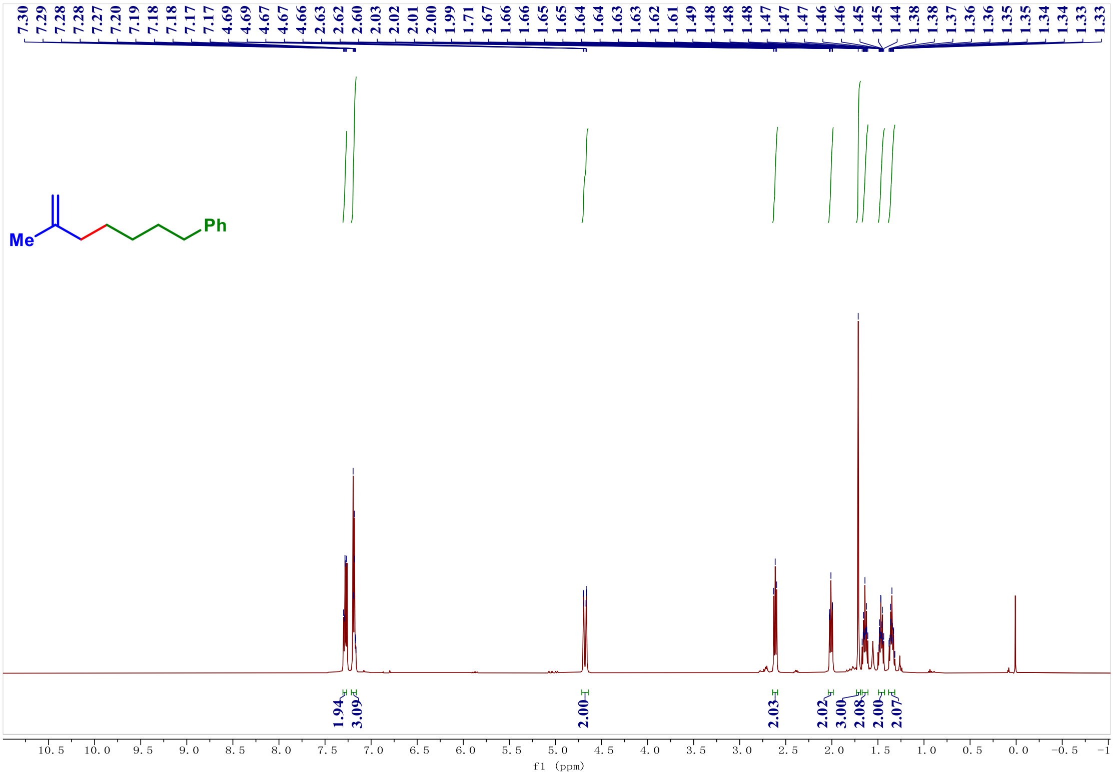

^{13}C NMR of Compound 30 (126 MHz, $CDCl_3$):

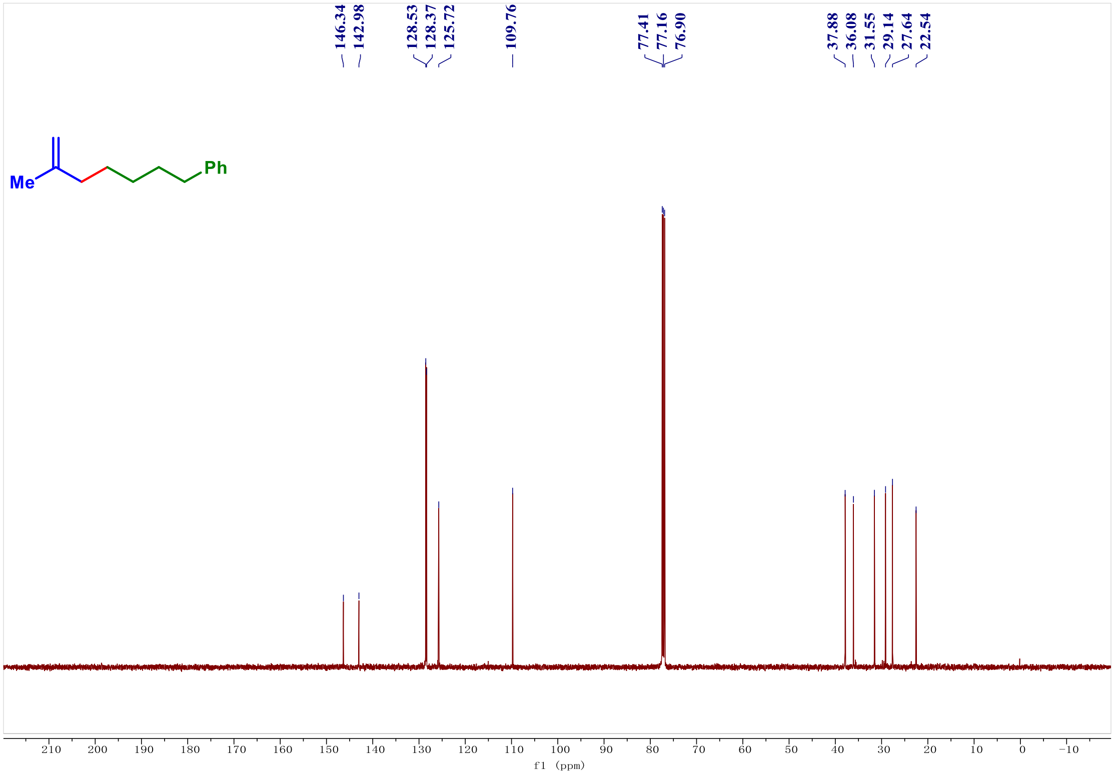

^{1}H NMR of Compound 31 (400 MHz, $CDCl_3$):

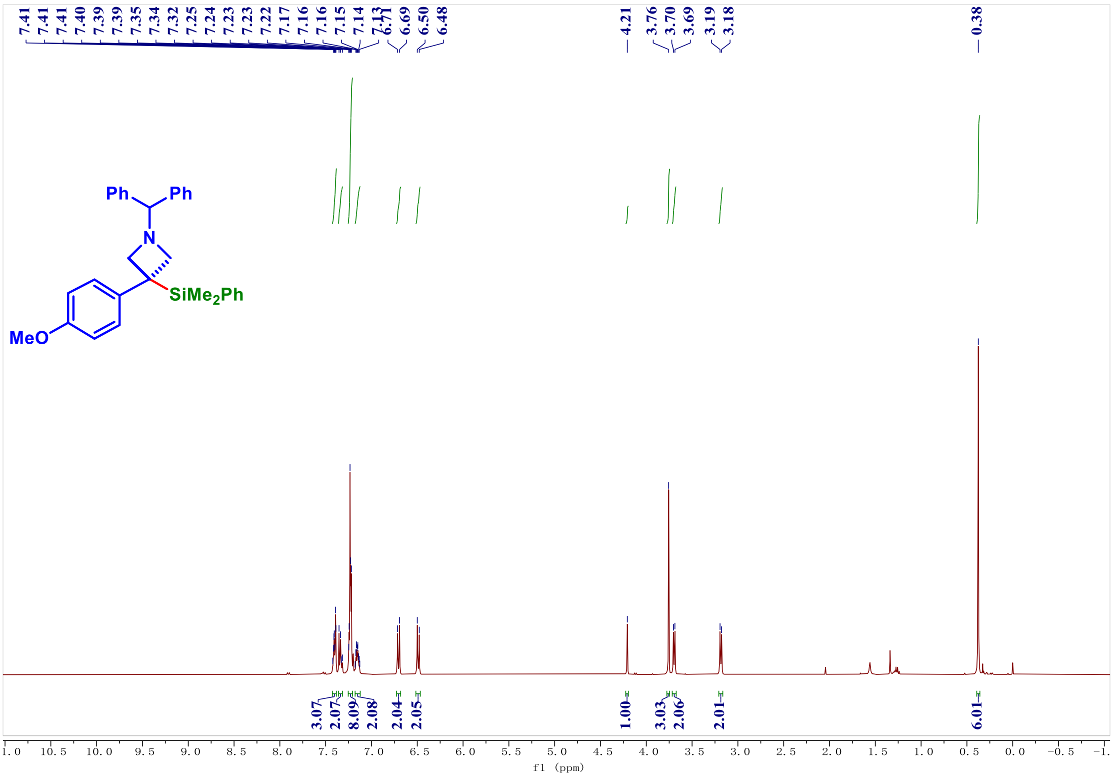

^{13}C NMR of Compound 31 (126 MHz, $CDCl_3$):

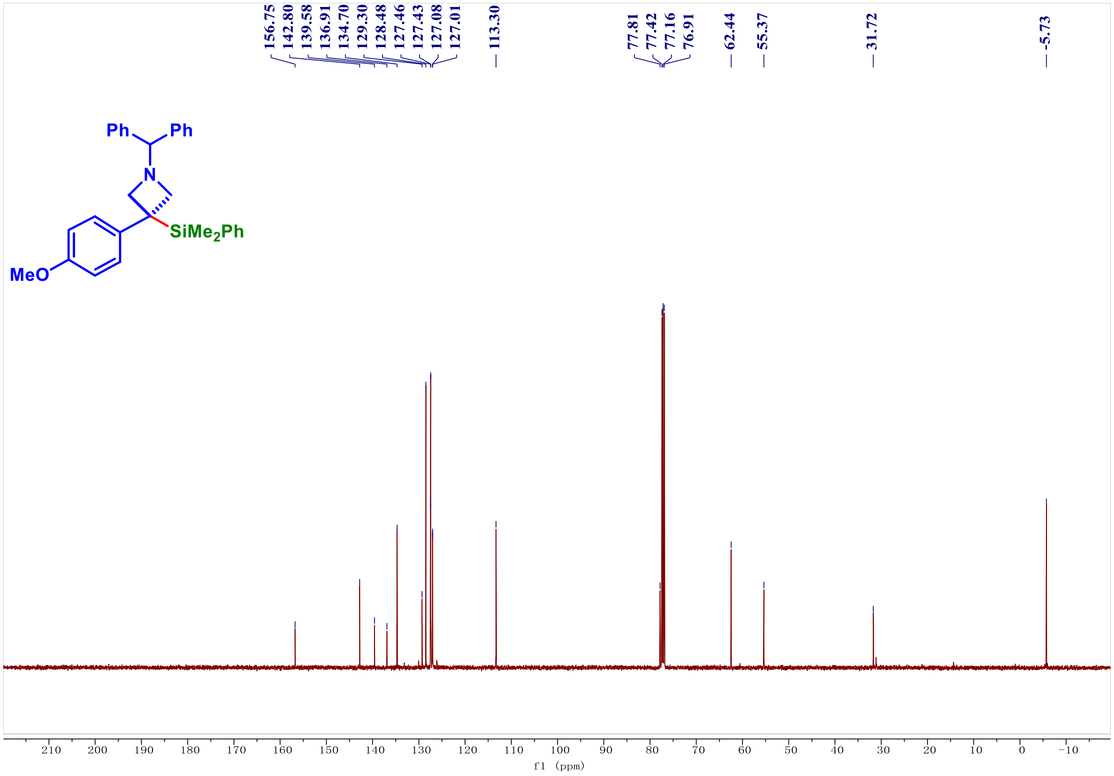

^{1}H NMR of Compound 32 (500 MHz, $CDCl_3$):

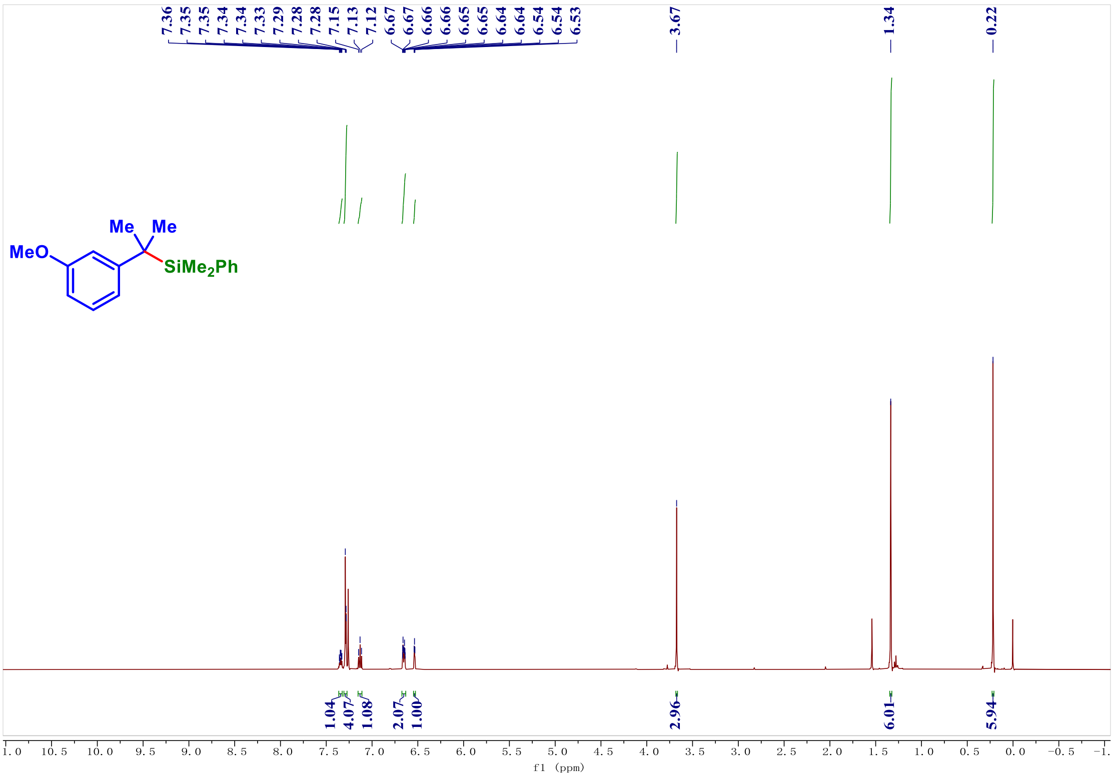

^{13}C NMR of Compound 32 (126 MHz, $CDCl_3$):

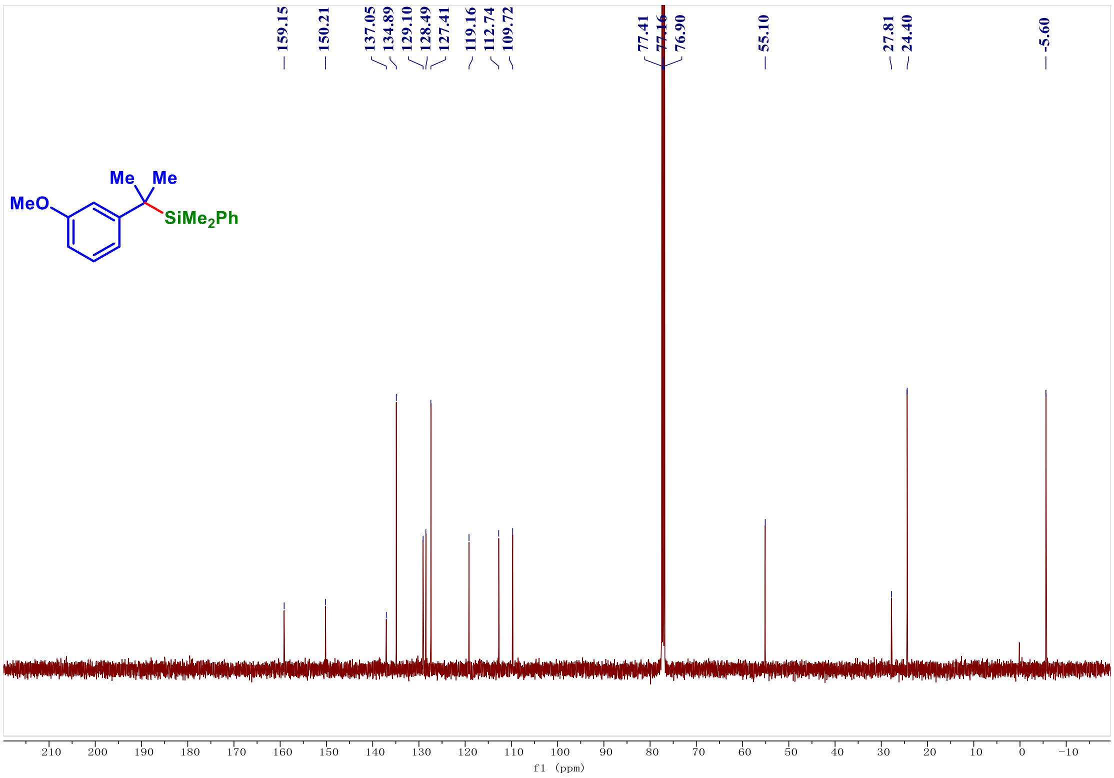

^{1}H NMR of Compound 33 (500 MHz, $CDCl_3$):

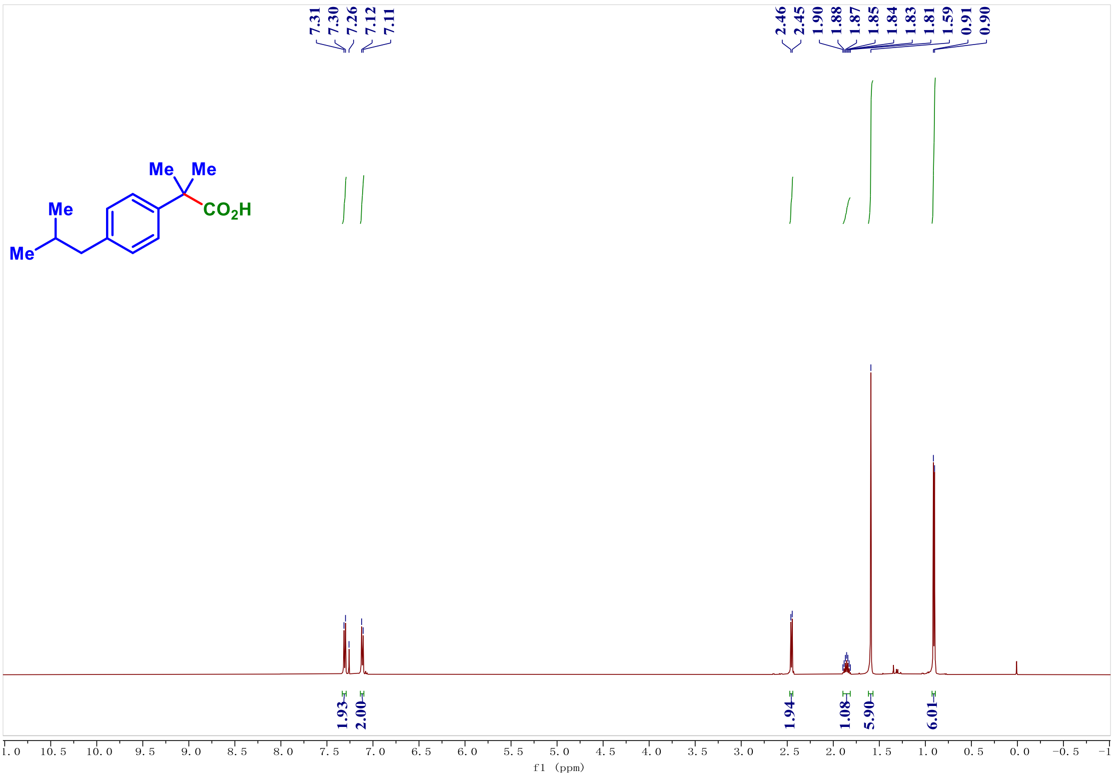

^{13}C NMR of Compound 33 (101 MHz, $CDCl_3$):

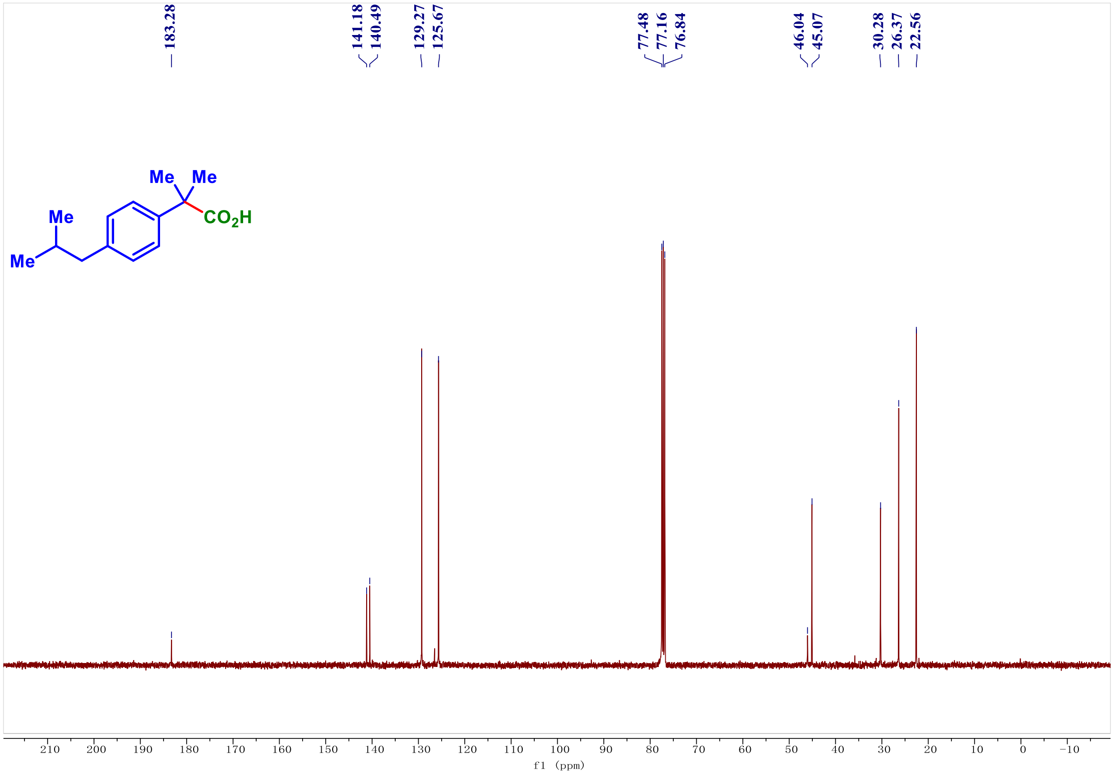

^{1}H NMR of Compound 34 (500 MHz, $CDCl_3$):

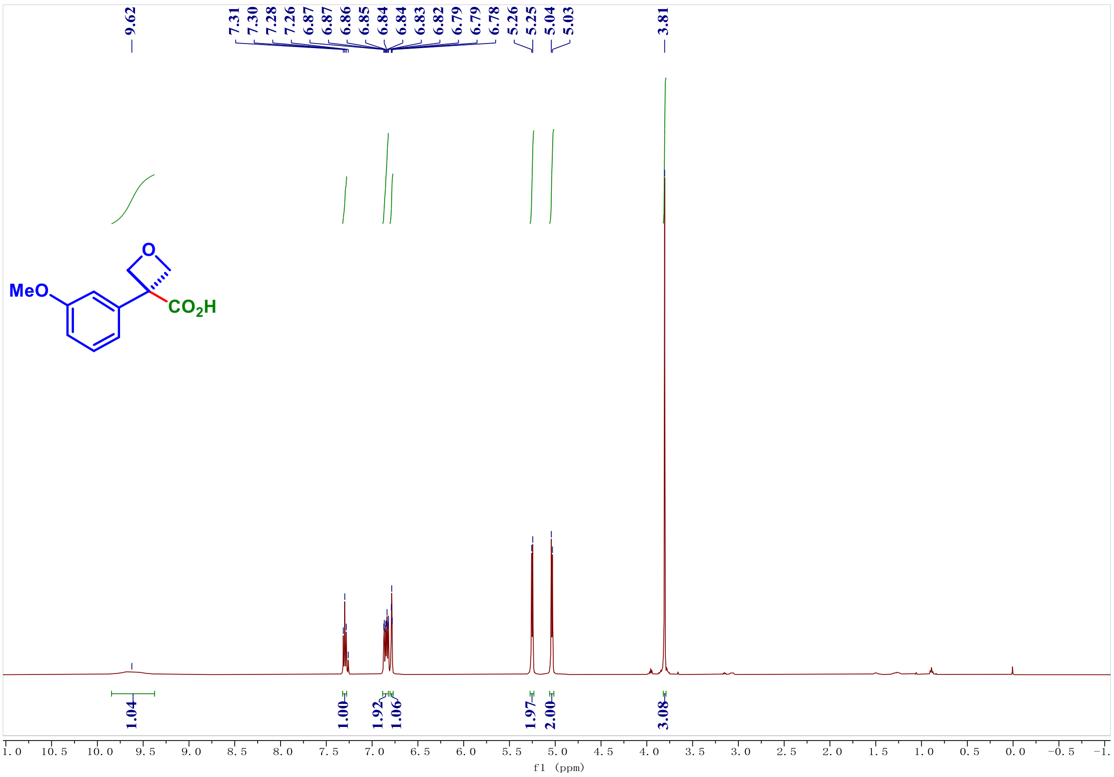

^{13}C NMR of Compound 34 (101 MHz, $CDCl_3$):

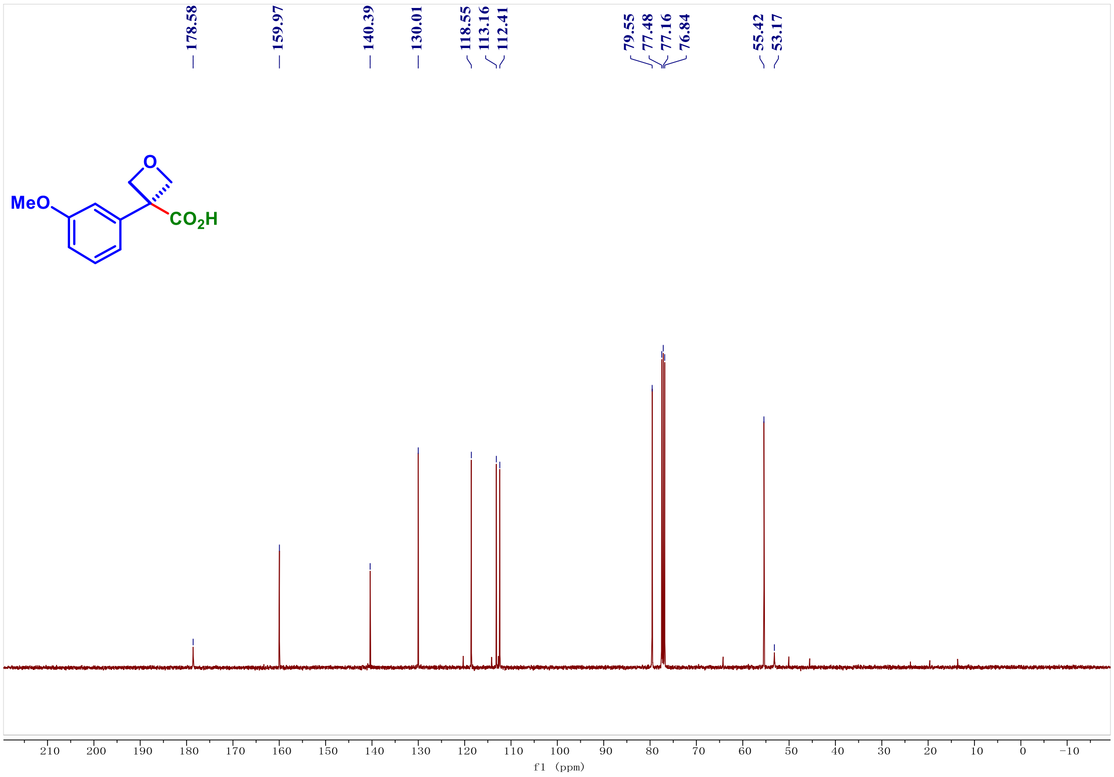

^{1}H NMR of Compound 35 (500 MHz, $CDCl_3$):

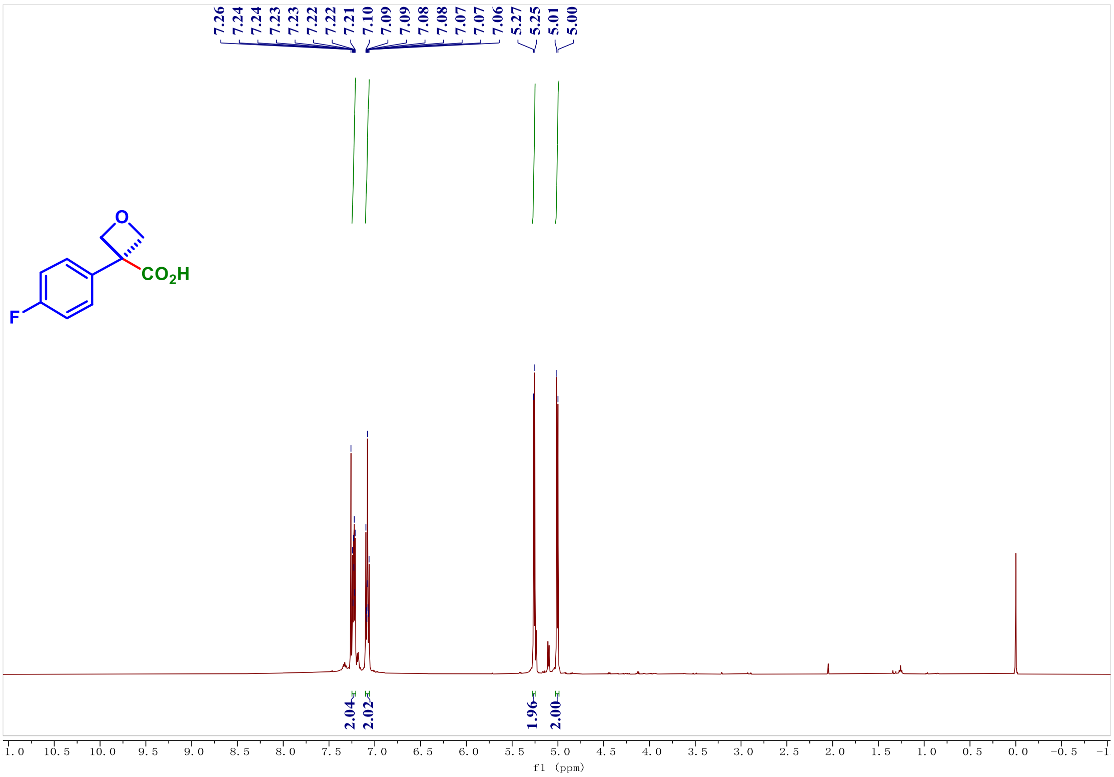

^{13}C NMR of Compound 35 (126 MHz, $CDCl_3$):

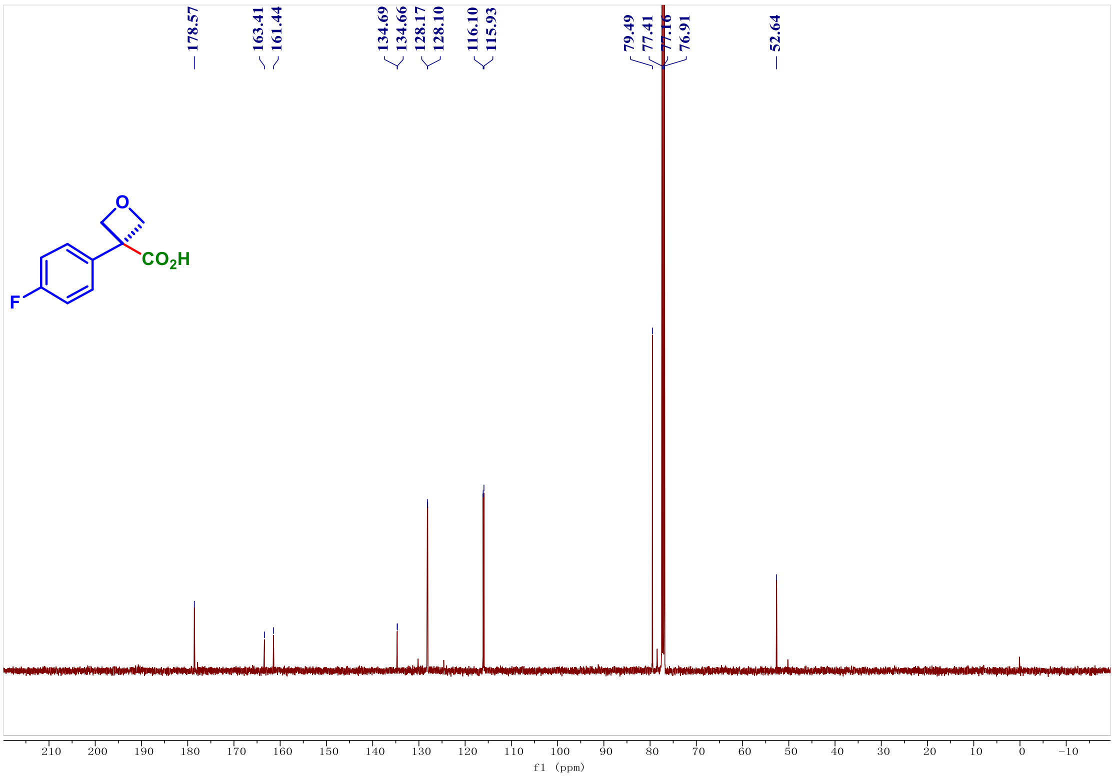

^{19}F NMR of Compound 35 (471 MHz, $CDCl_3$):

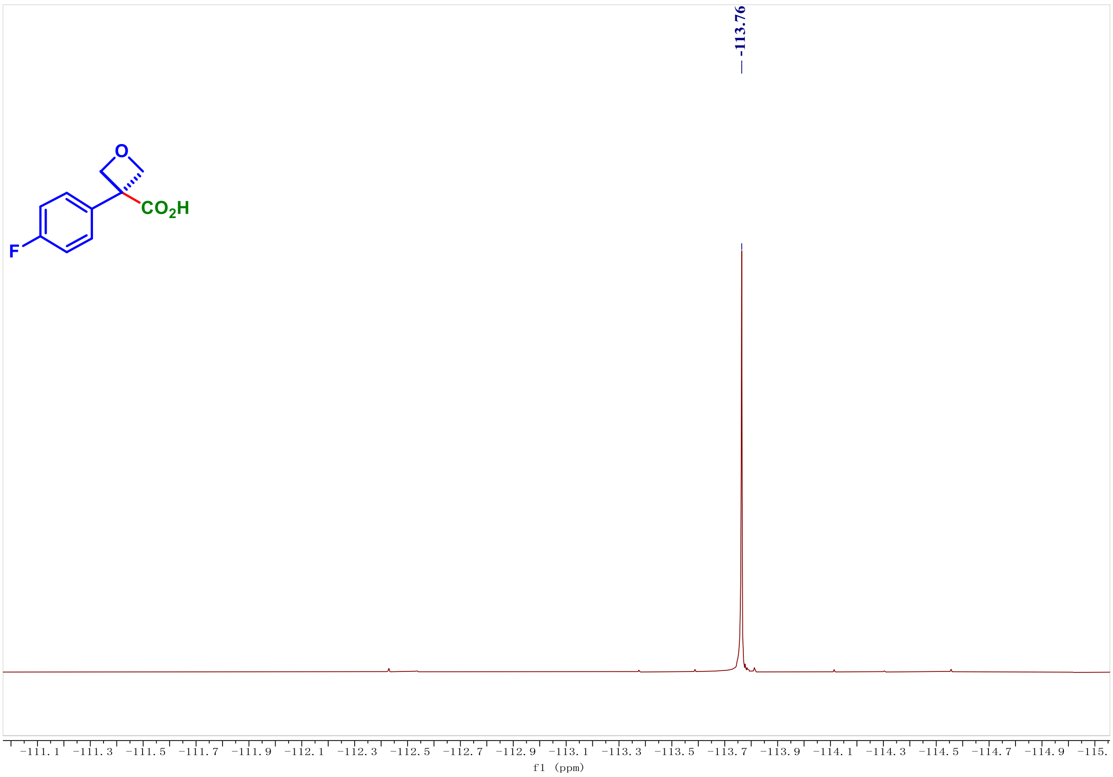

^{1}H NMR of Compound 38 (400 MHz, CDCl$_3$):

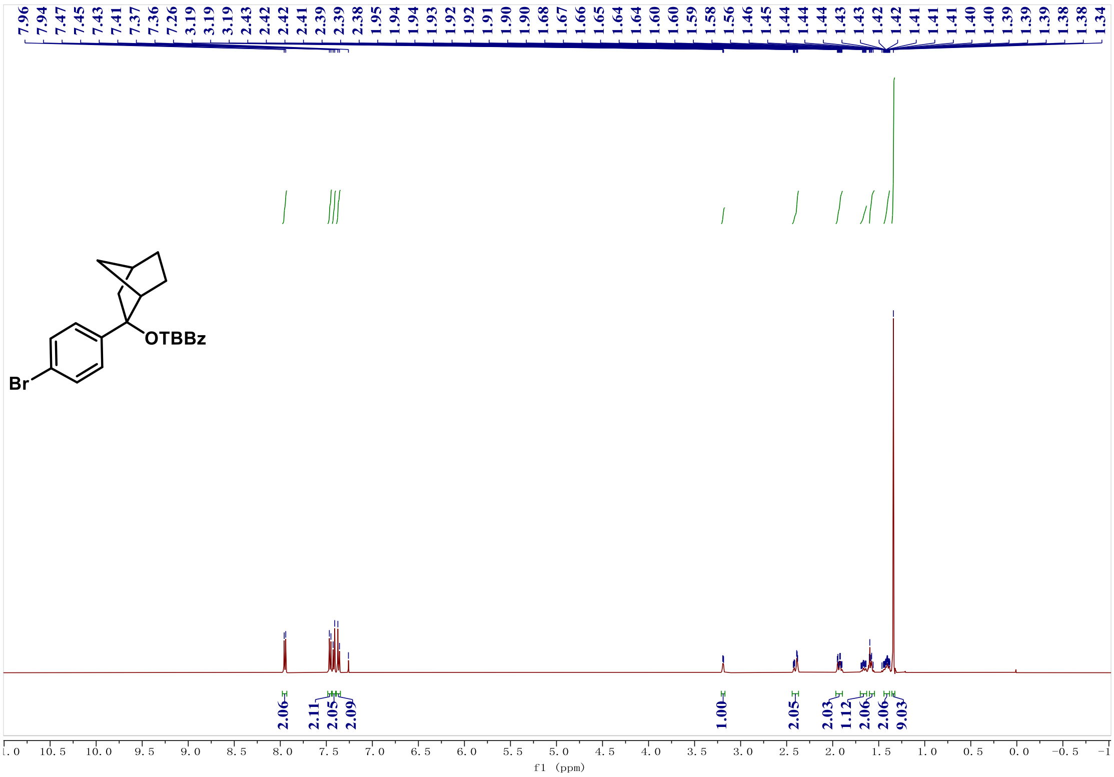

^{13}C NMR of Compound 38 (126 MHz, CDCl$_3$):

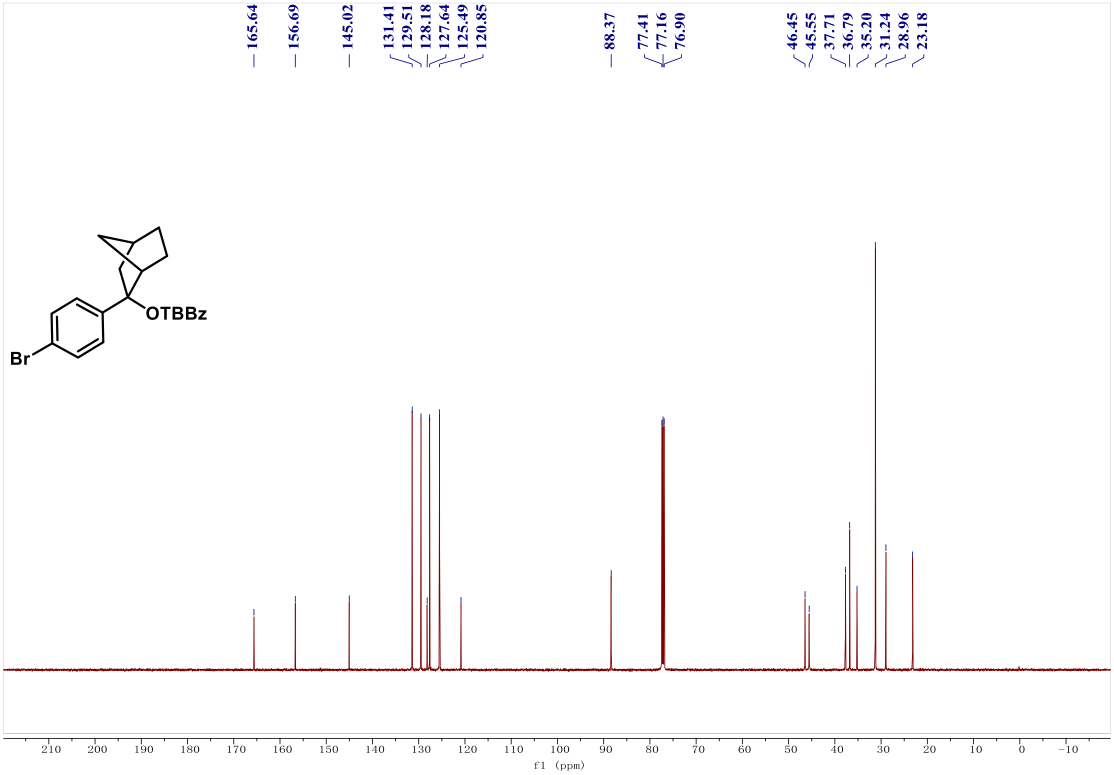

^{1}H NMR of Compound 39 (400 MHz, $CDCl_3$):

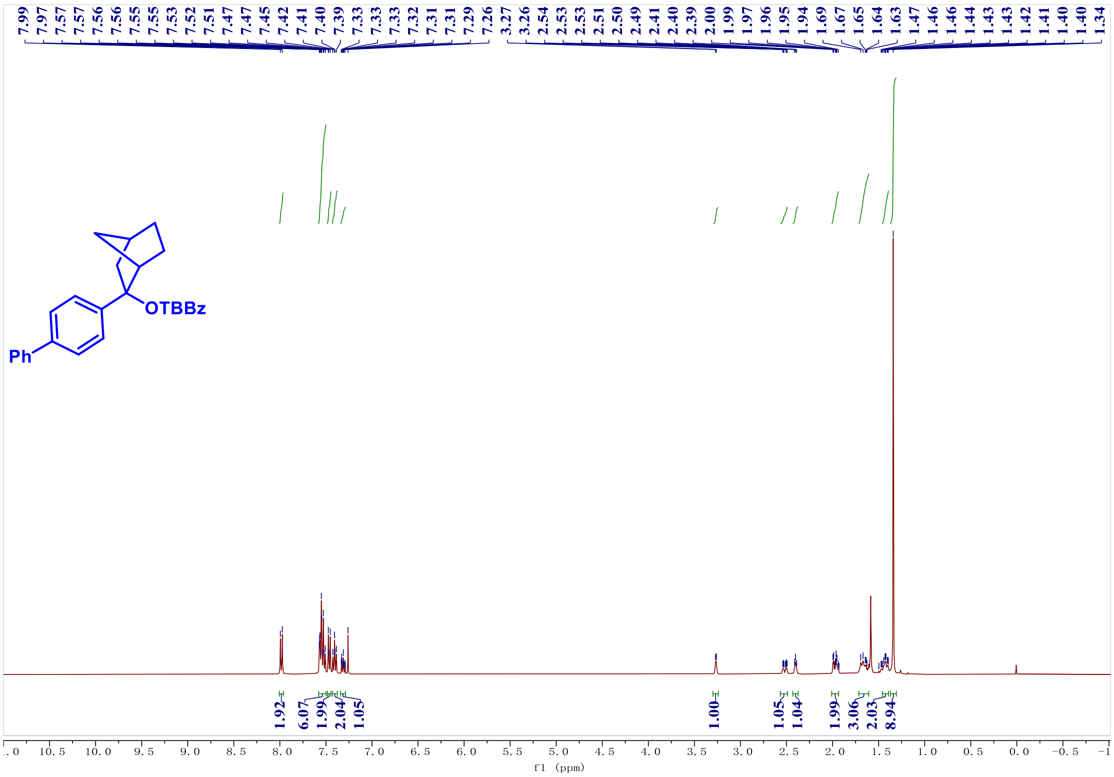

^{13}C NMR of Compound 39 (101 MHz, $CDCl_3$):

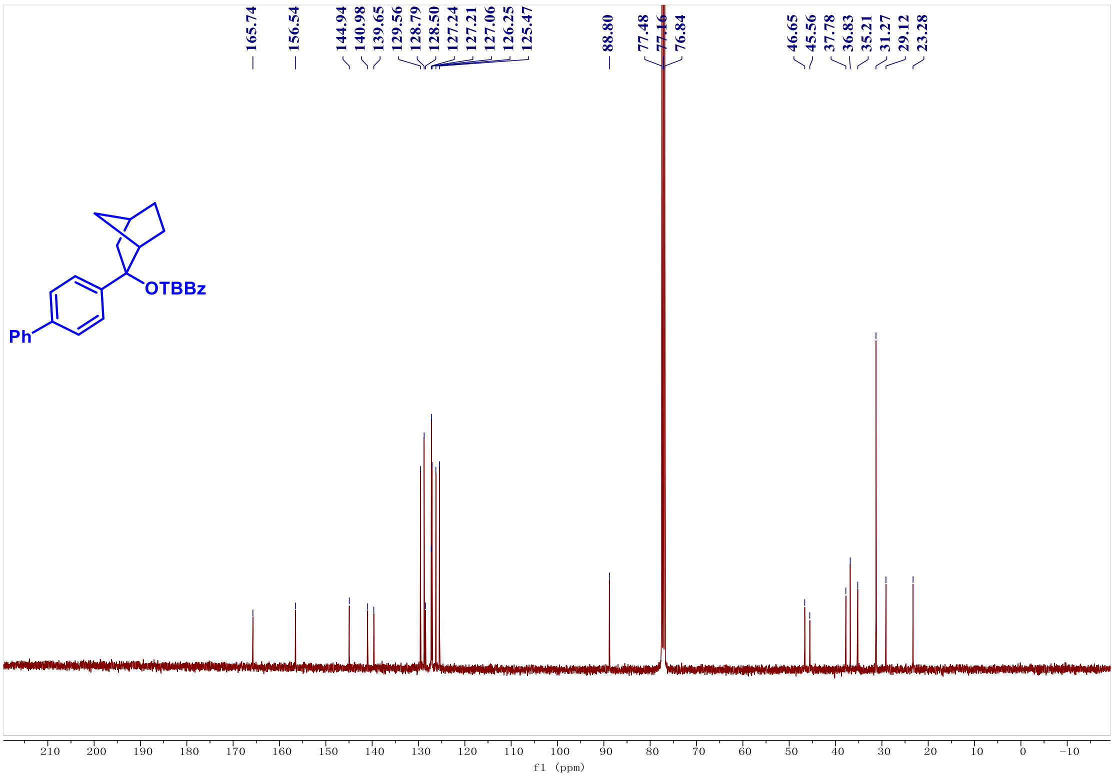

^{1}H NMR of Compound 40 (400 MHz, $CDCl_3$):

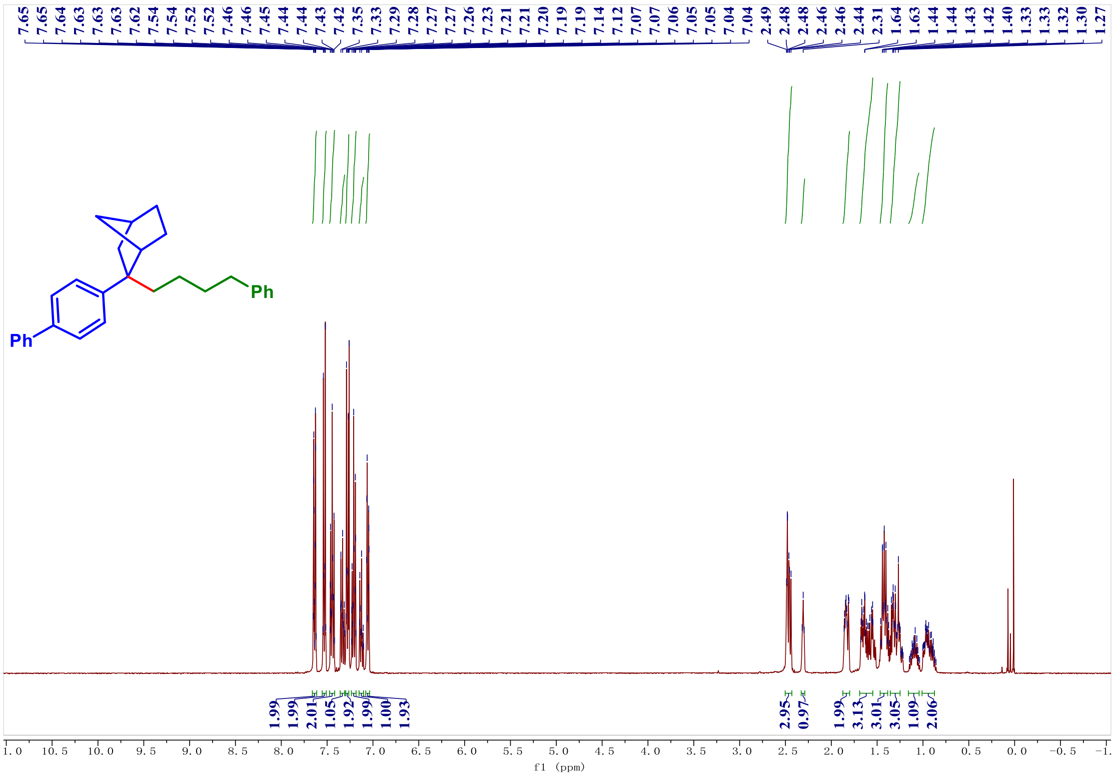

^{13}C NMR of Compound 40 (101 MHz, $CDCl_3$):

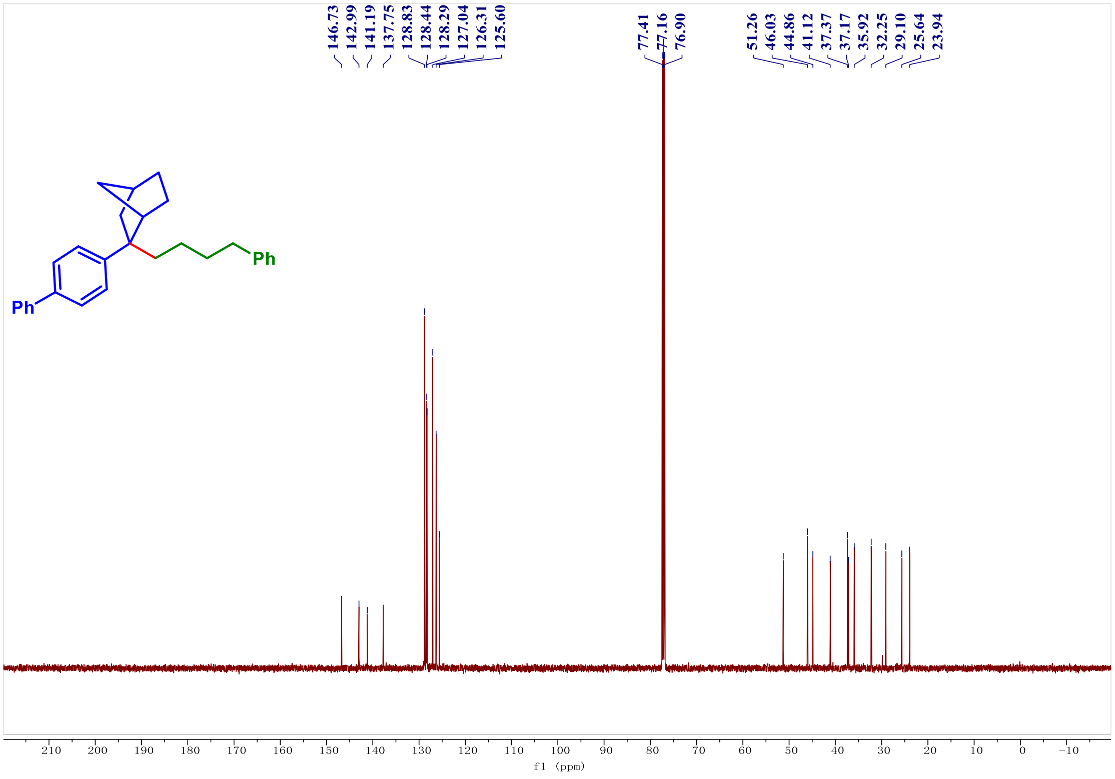

^{1}H NMR of Compound 41 (400 MHz, $CDCl_3$):

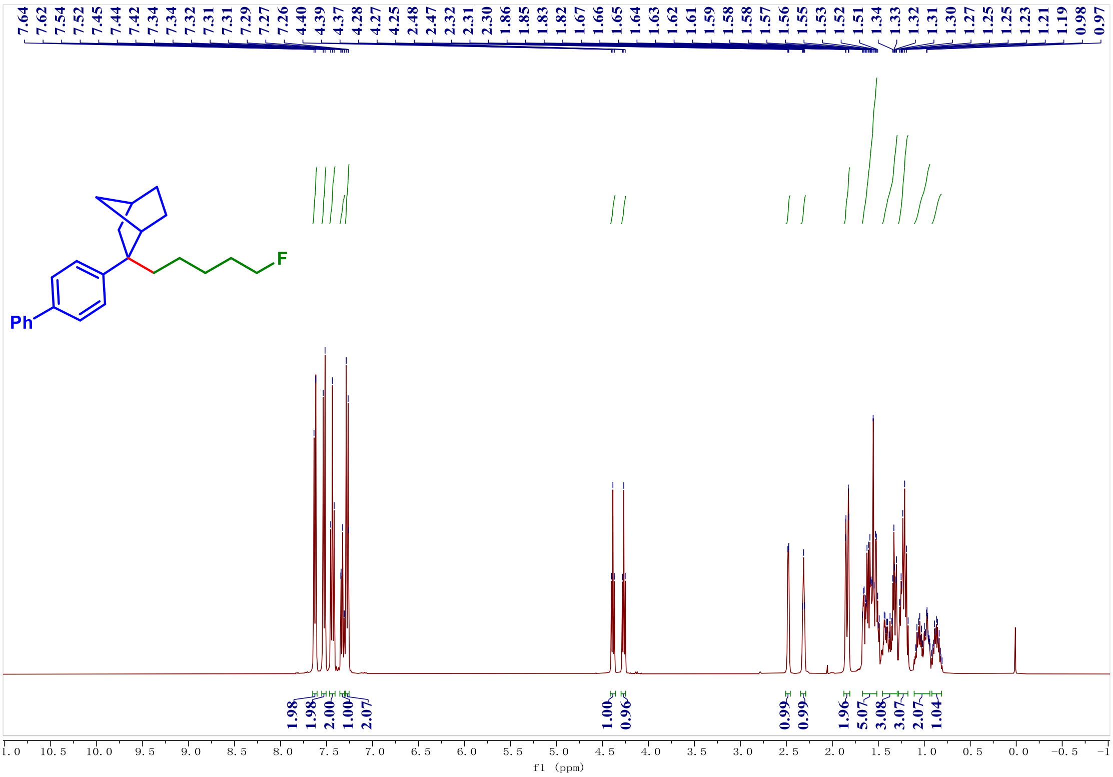

^{13}C NMR of Compound 41 (126 MHz, $CDCl_3$):

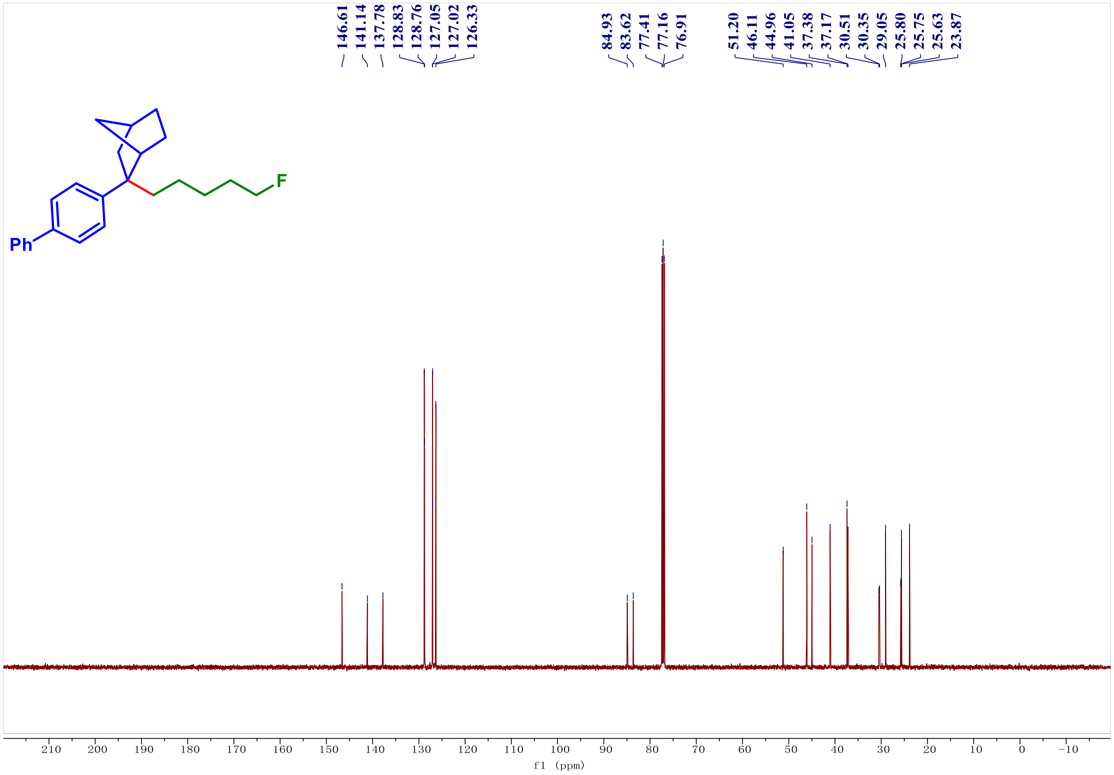

^{19}F NMR of Compound 41 (376 MHz, $CDCl_3$):

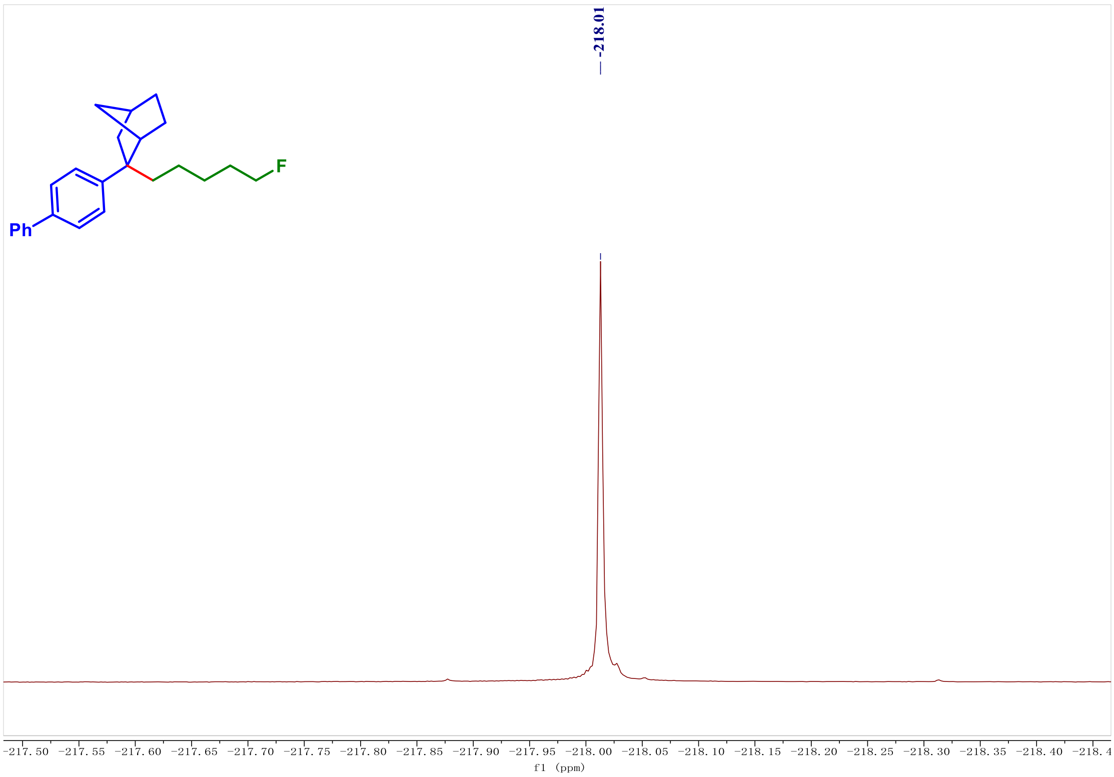

^{1}H NMR of Compound 43 (500 MHz, $CDCl_3$):

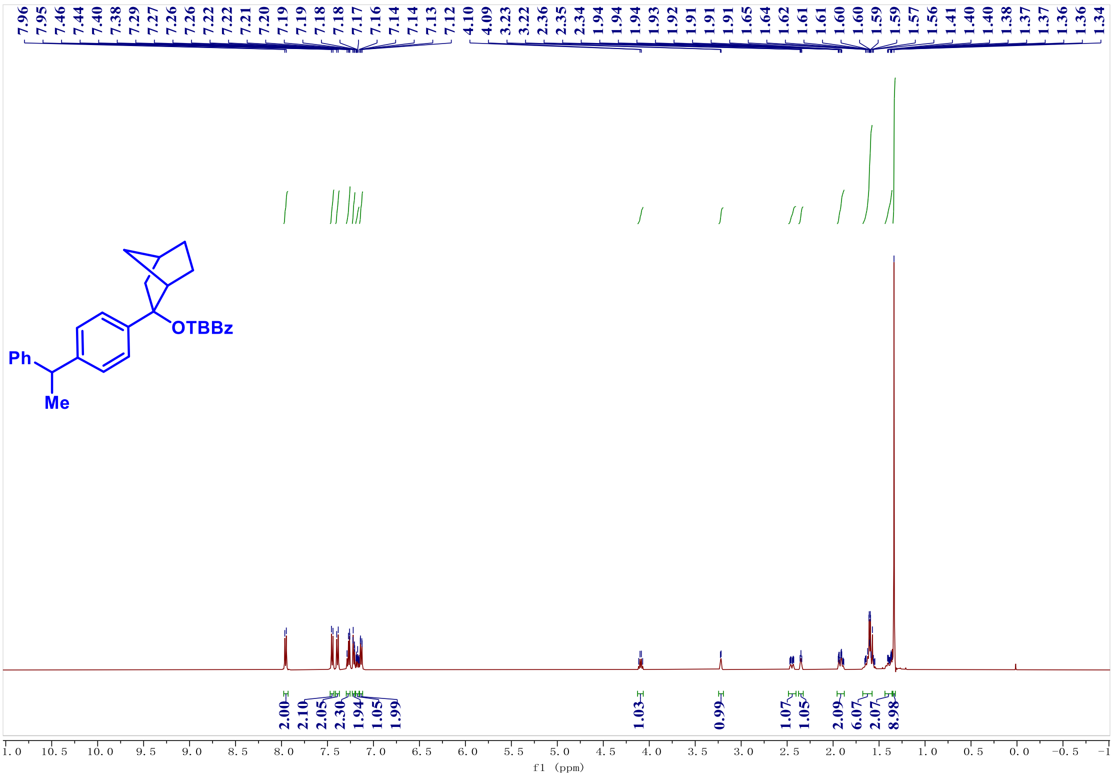

^{13}C NMR of Compound 43 (126 MHz, $CDCl_3$):

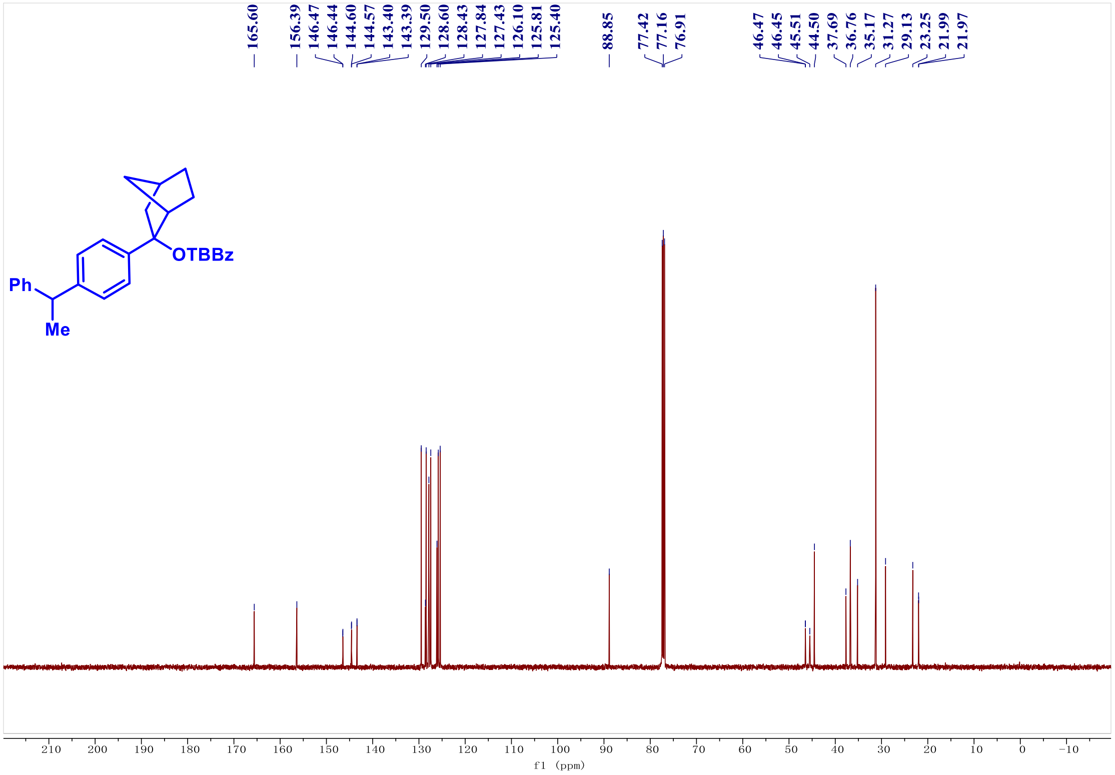

^{1}H NMR of Compound 44 (400 MHz, $CDCl_3$):

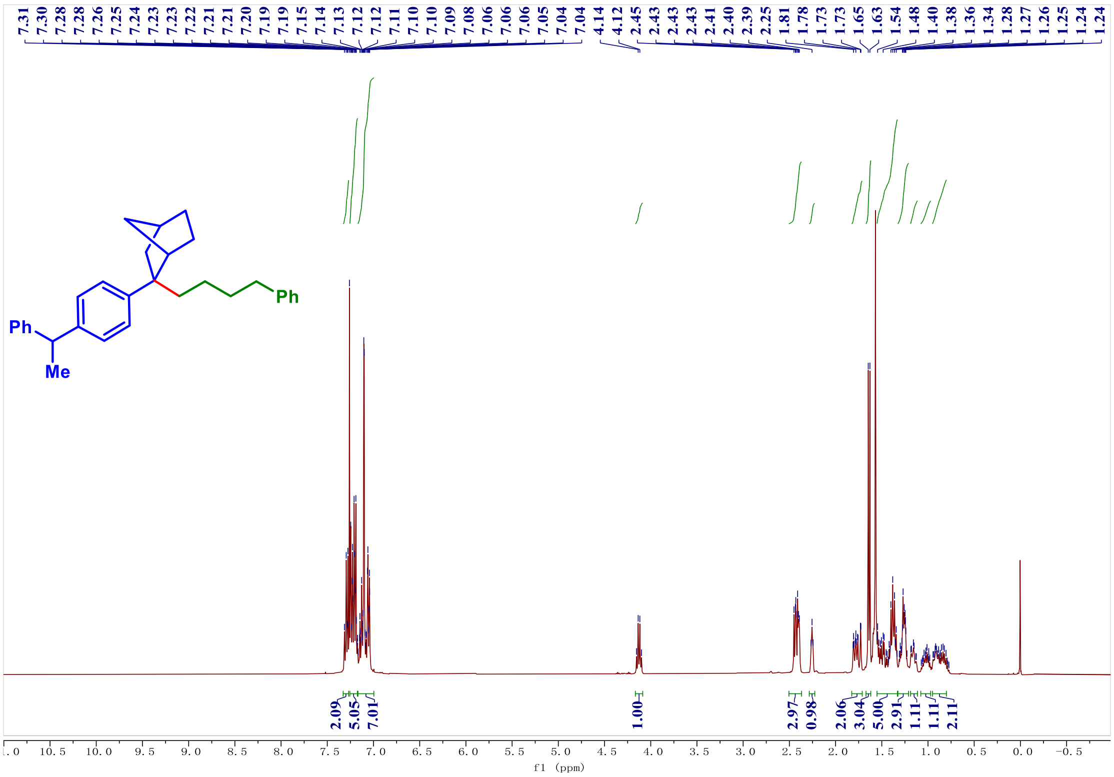

^{13}C NMR of Compound 44 (101 MHz, $CDCl_3$):

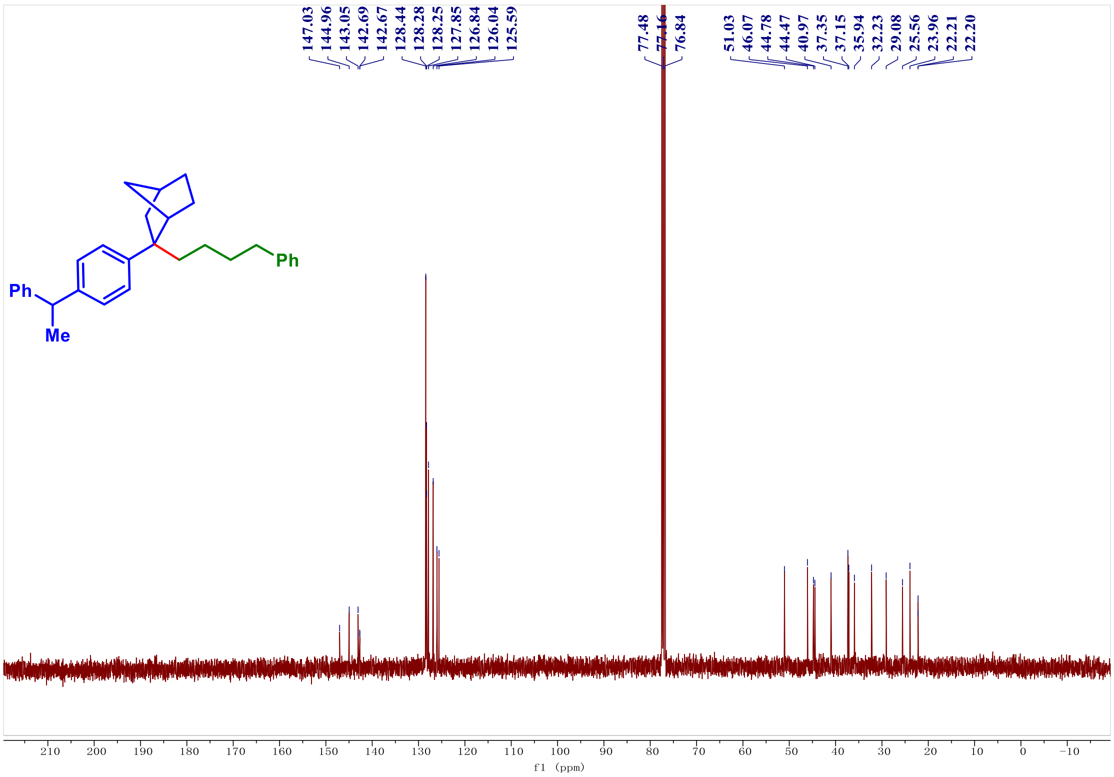

^{1}H NMR of Compound 45 (400 MHz, $CDCl_3$):

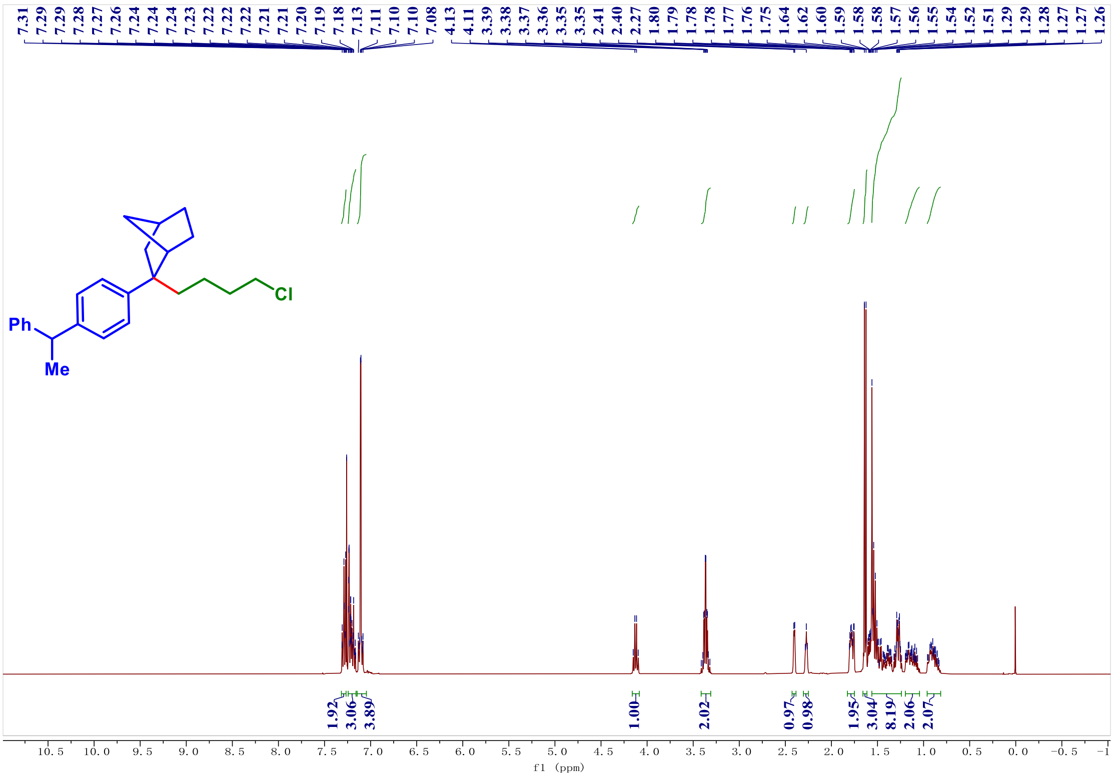

^{13}C NMR of Compound 45 (101 MHz, $CDCl_3$):

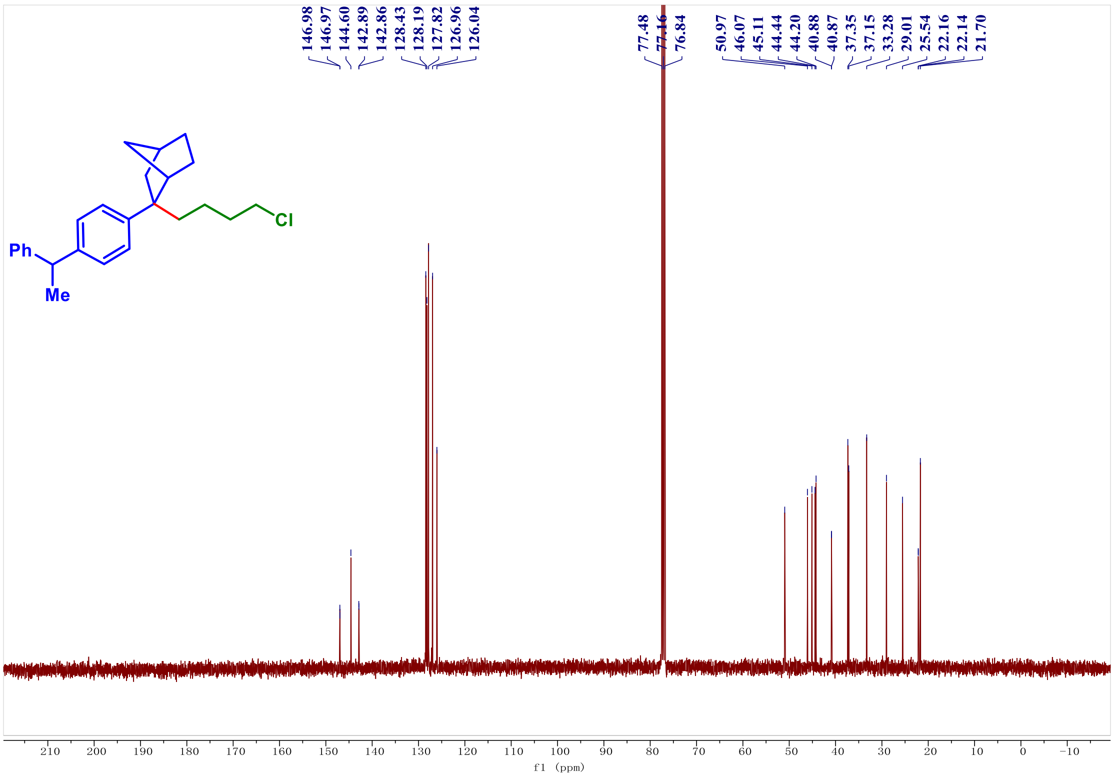

^{1}H NMR of Compound 48 (400 MHz, CDCl$_3$):

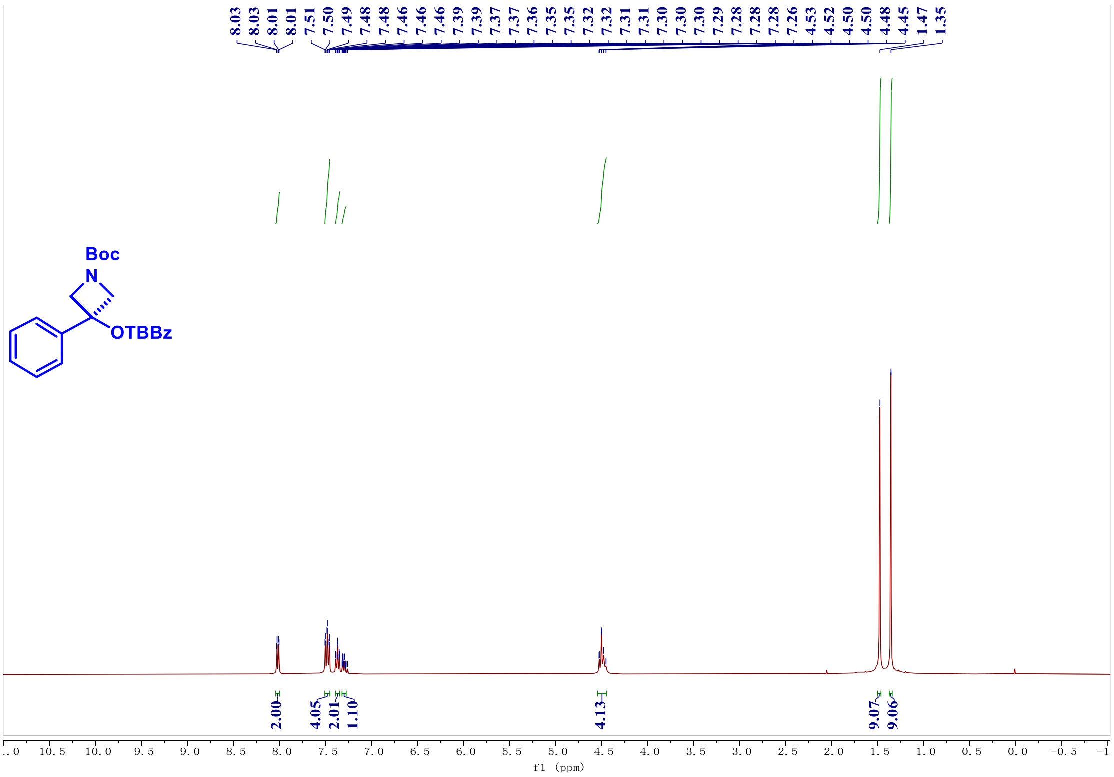

^{13}C NMR of Compound 48 (101 MHz, CDCl$_3$):

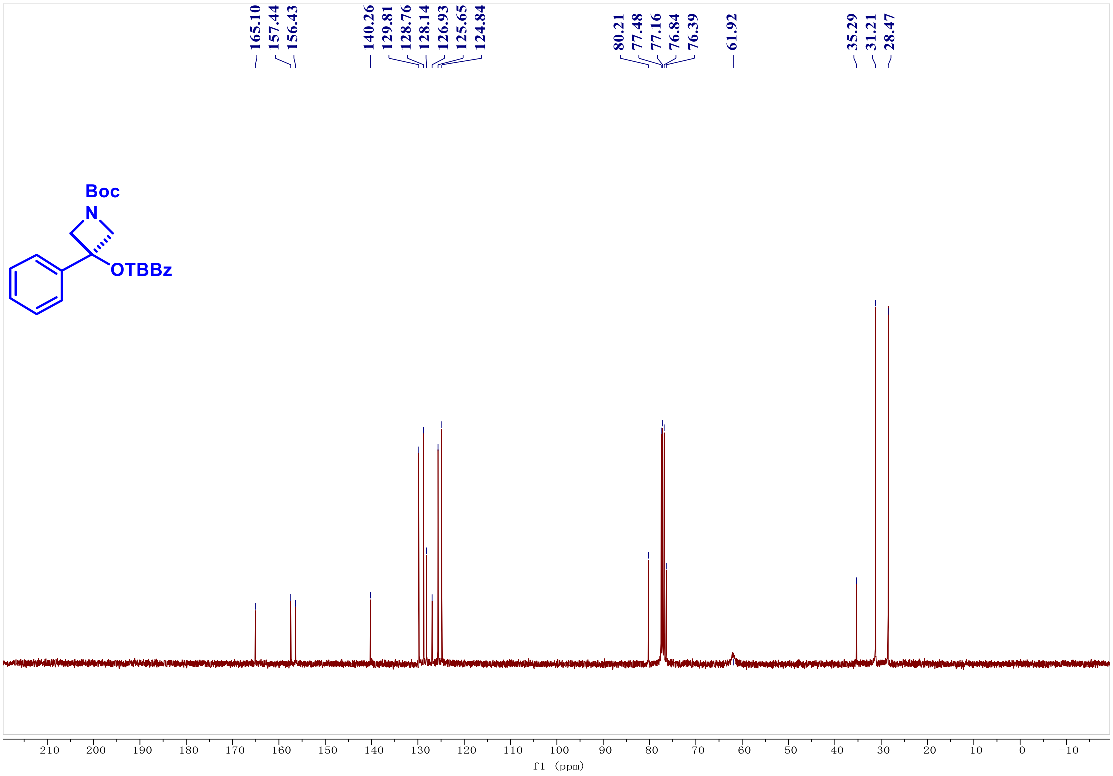

^{1}H NMR of Compound 50 (500 MHz, $CDCl_3$):

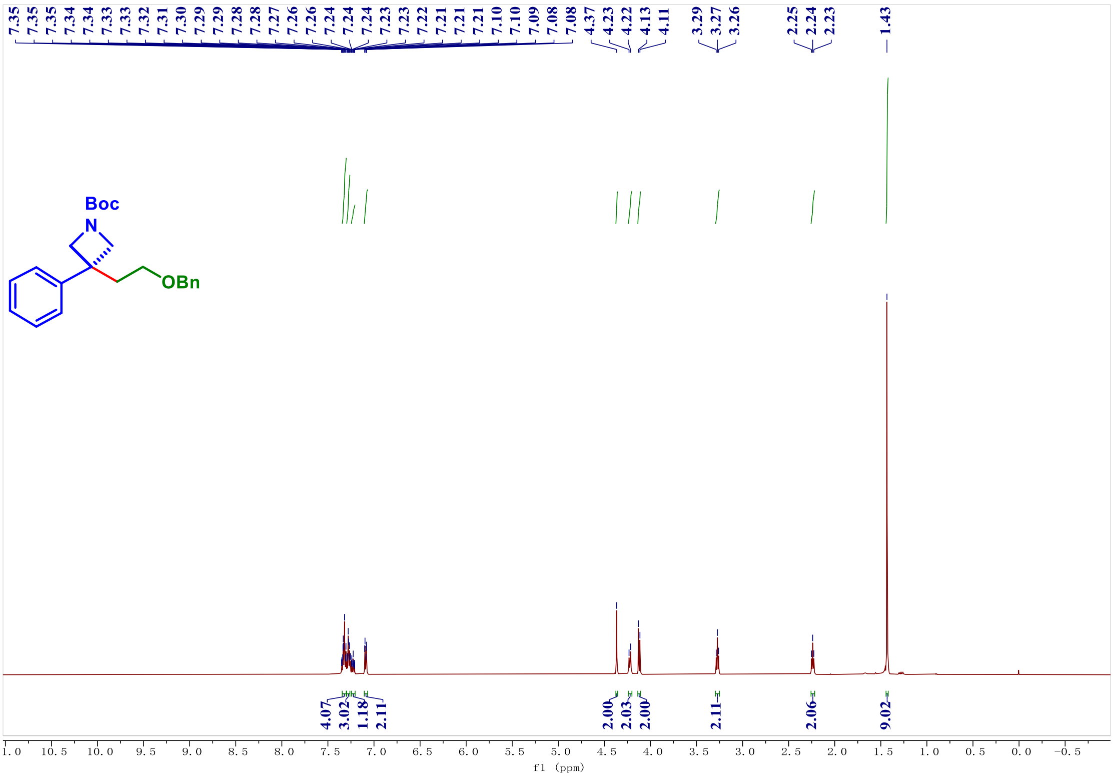

^{13}C NMR of Compound 50 (126 MHz, $CDCl_3$):

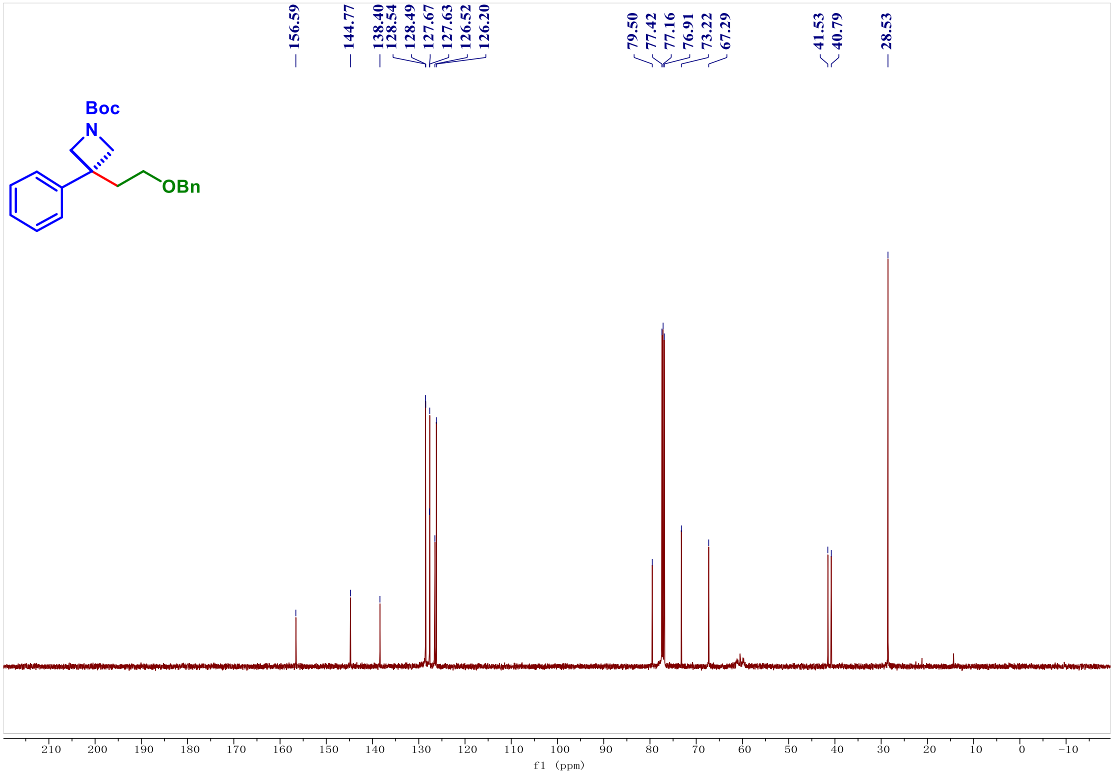

^{1}H NMR of Compound 53 (400 MHz, $CDCl_3$):

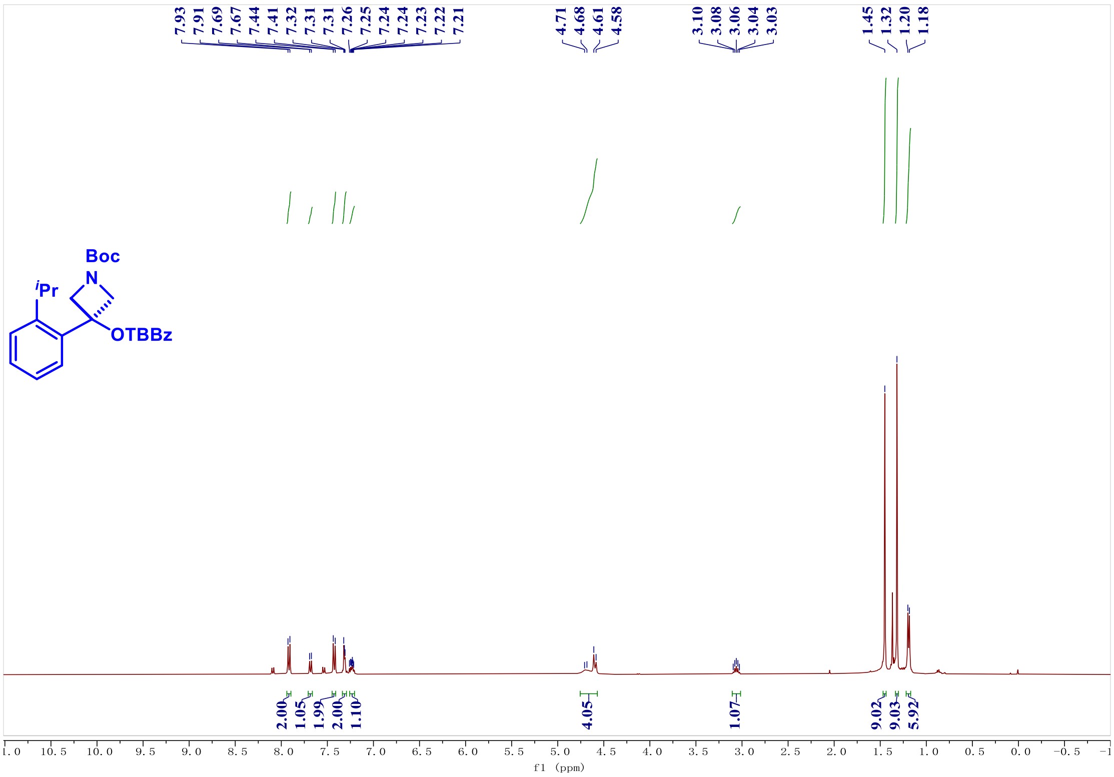

^{13}C NMR of Compound 53 (101 MHz, $CDCl_3$):

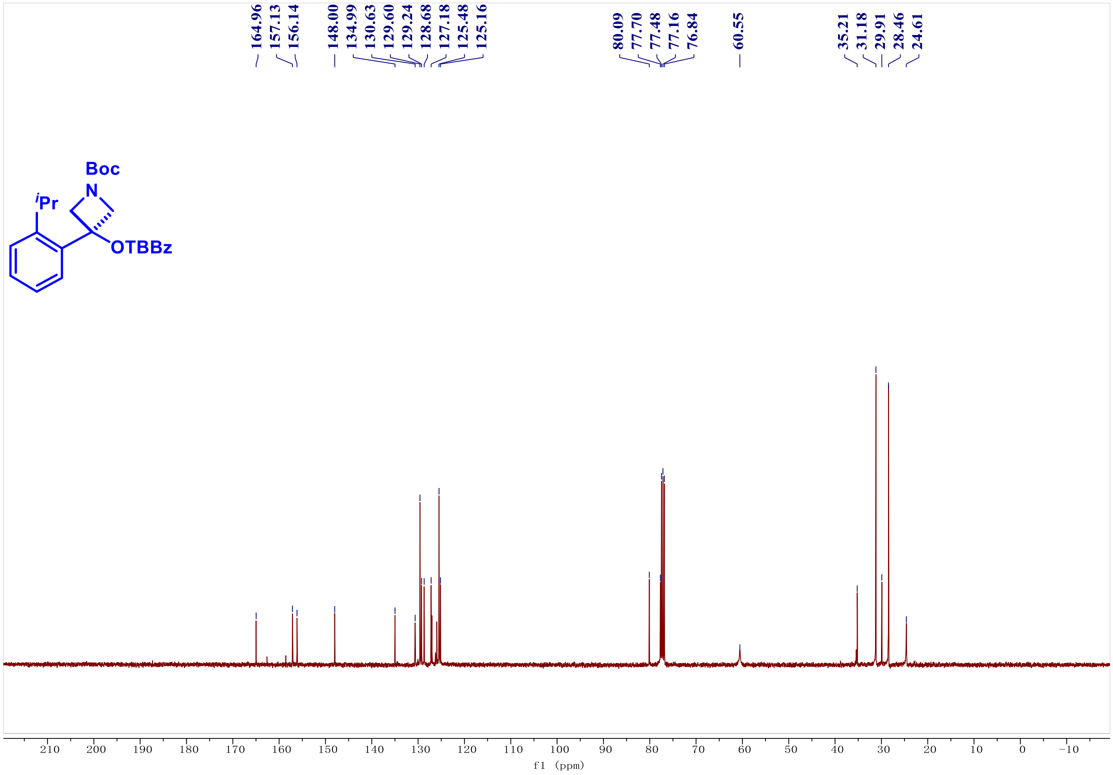

^{1}H NMR of Compound 54 (500 MHz, $CDCl_3$):

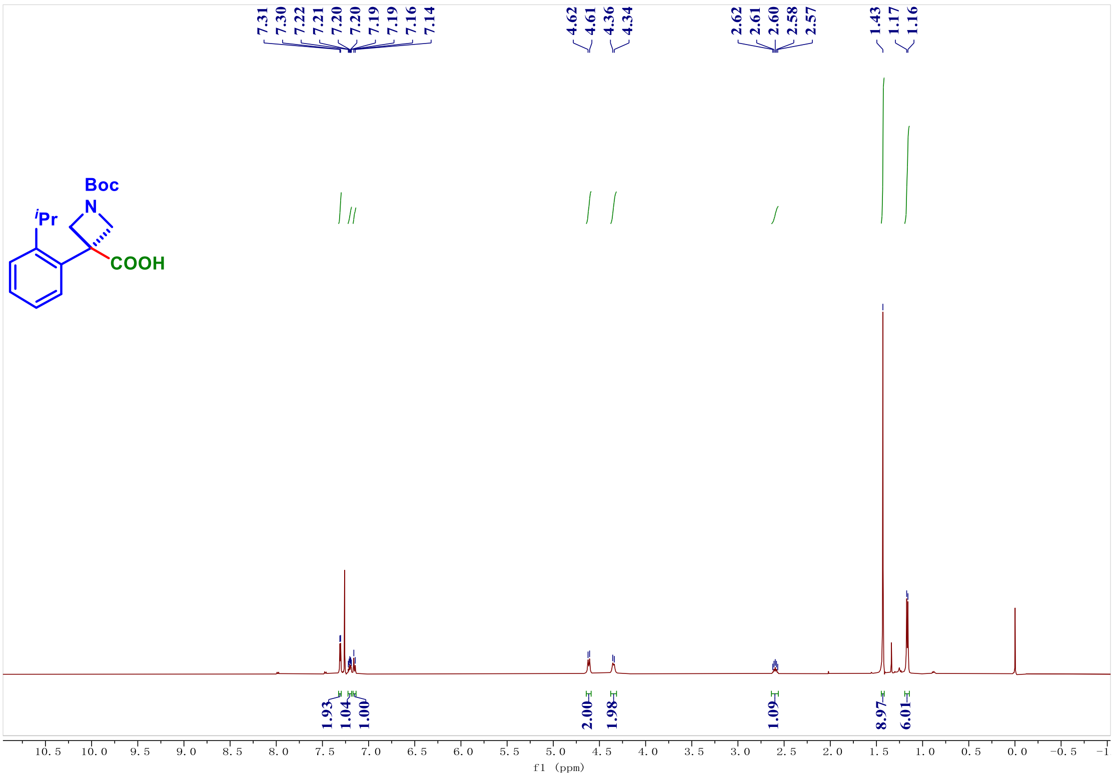

^{13}C NMR of Compound 54 (101 MHz, $CDCl_3$):

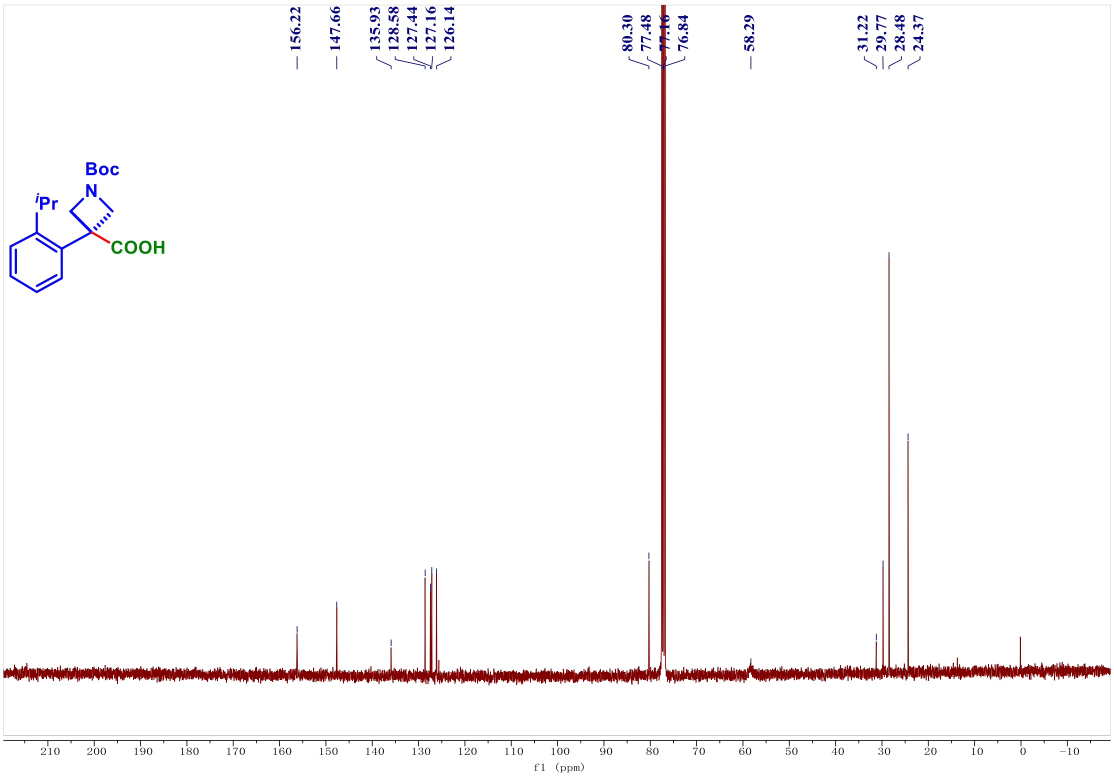

^{1}H NMR of Compound 57 (400 MHz, $CDCl_3$):

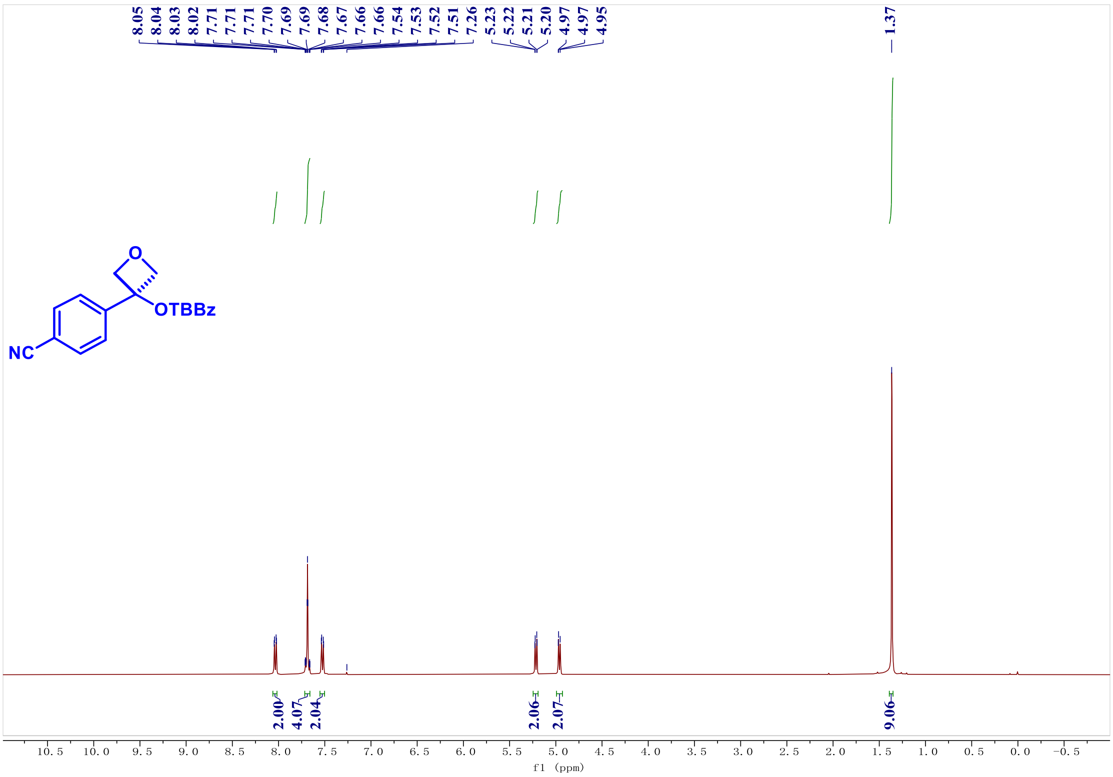

^{13}C NMR of Compound 57 (101 MHz, $CDCl_3$):

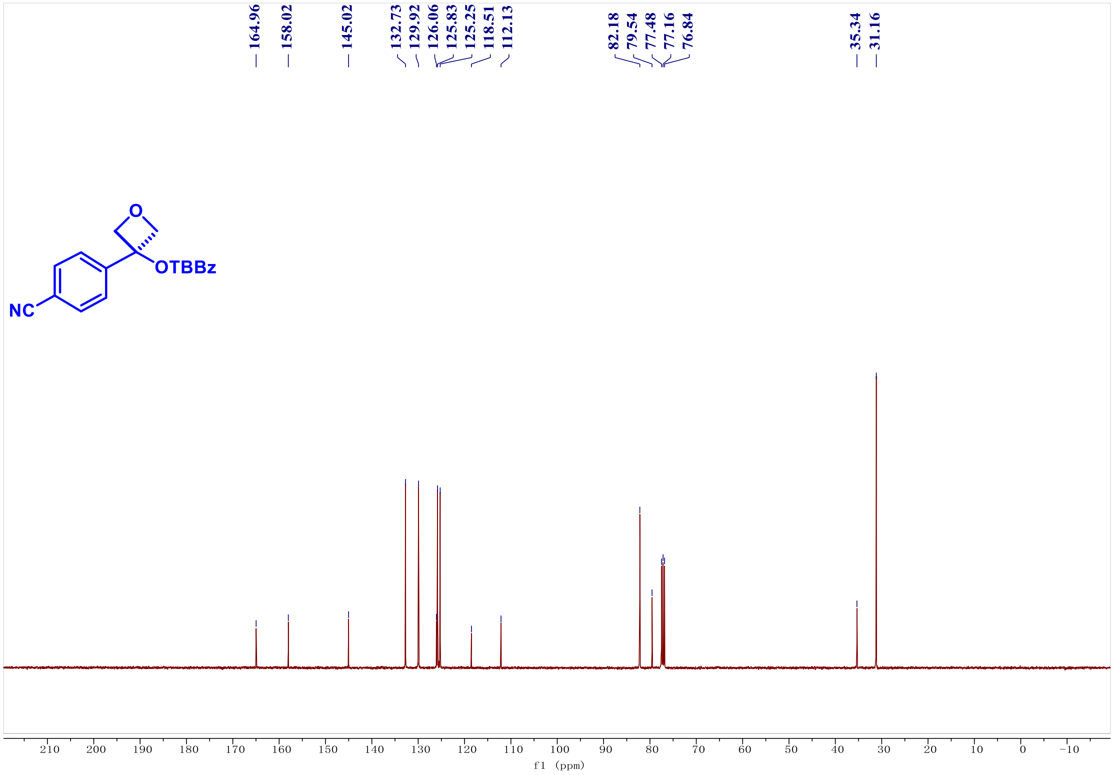

^{1}H NMR of Compound 58 (400 MHz, $CDCl_3$):

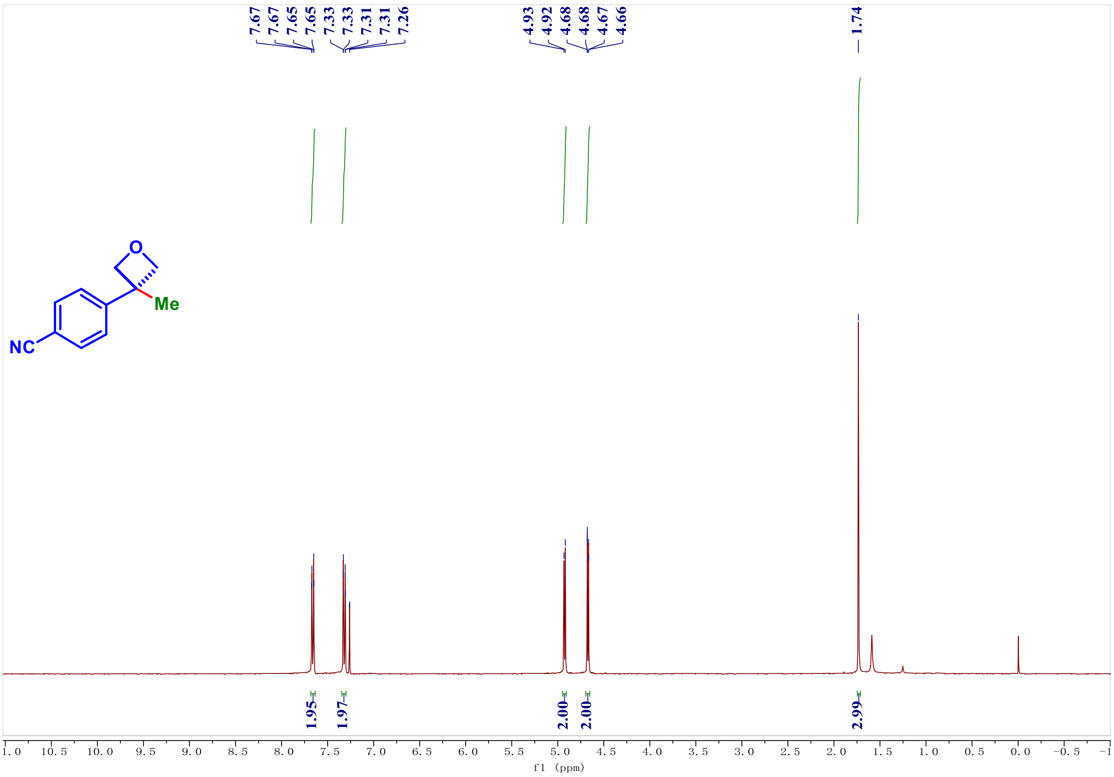

^{13}C NMR of Compound 58 (101 MHz, $CDCl_3$):

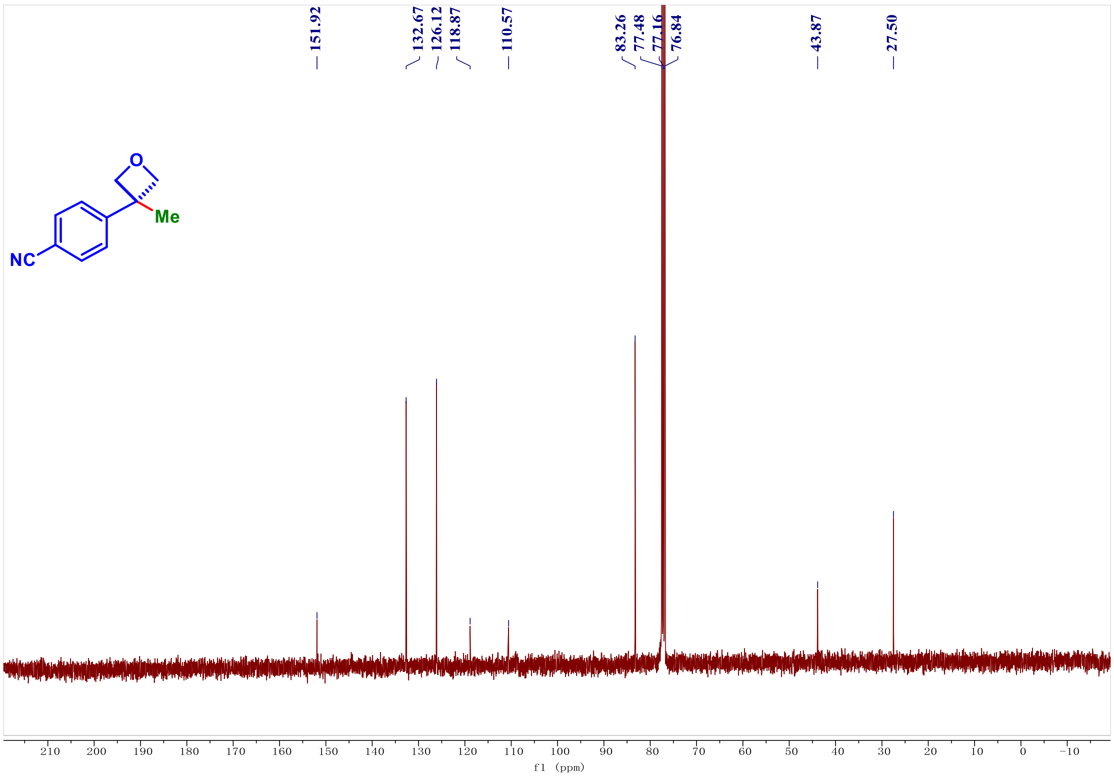

^{1}H NMR of Compound 59 (400 MHz, $CDCl_3$):

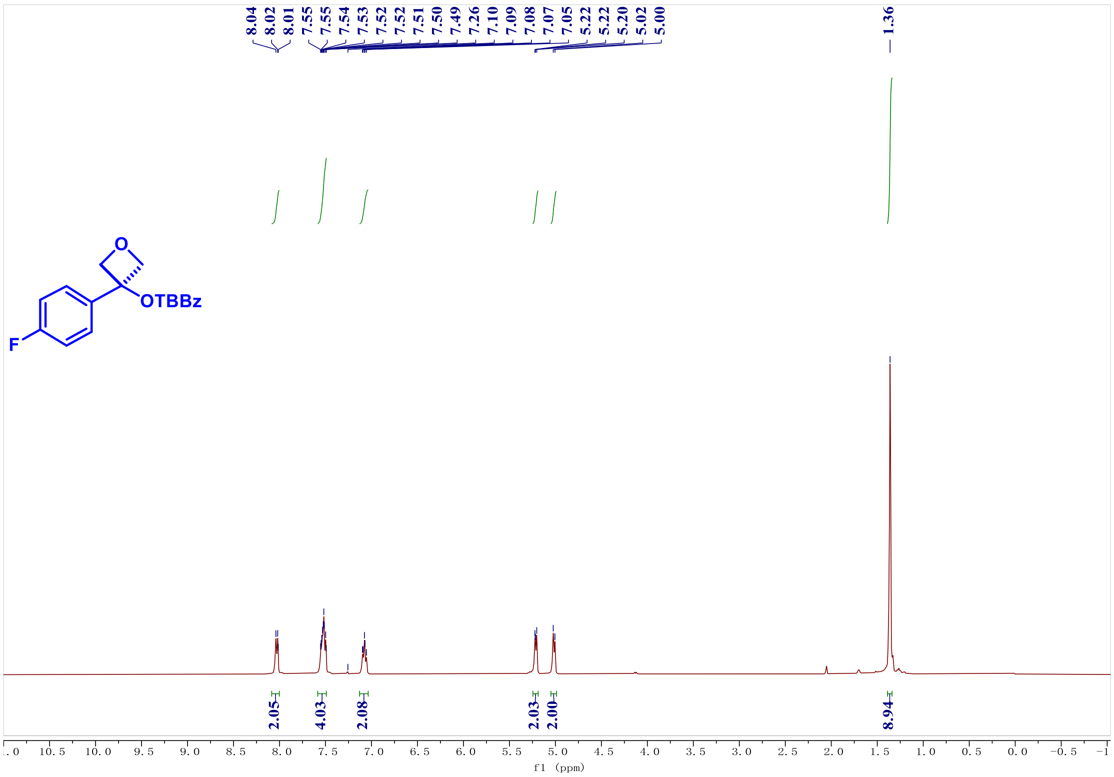

^{13}C NMR of Compound 59 (101 MHz, $CDCl_3$):

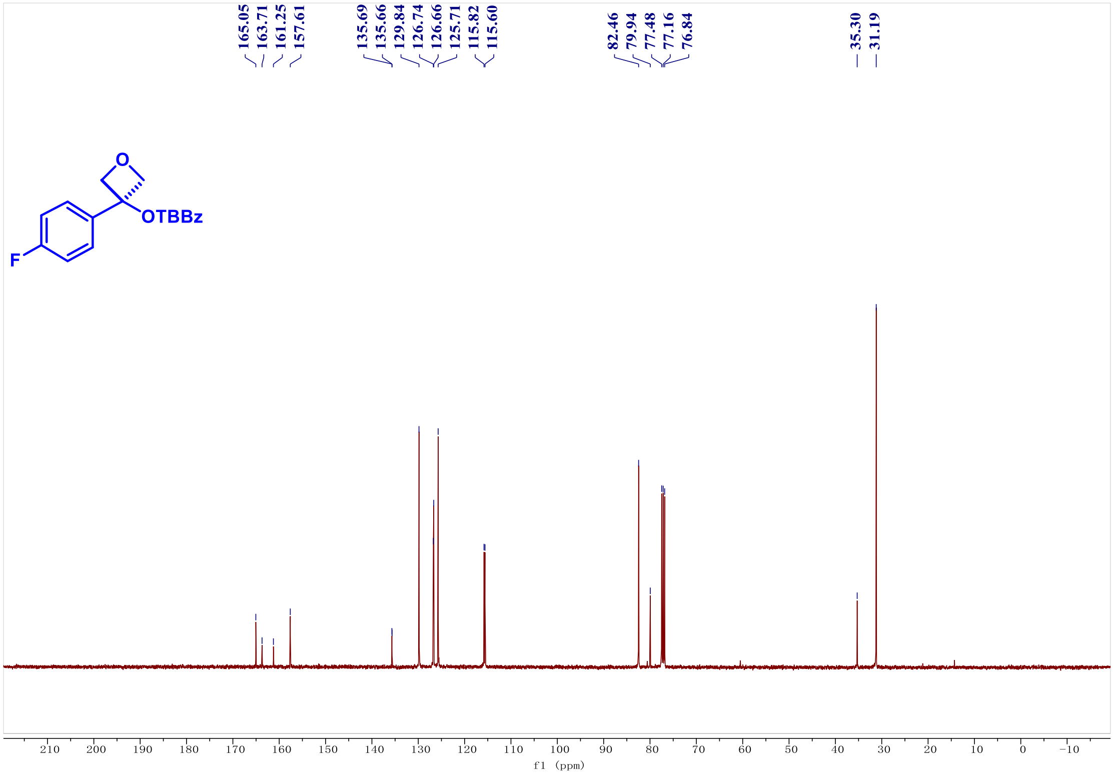

^{19}F NMR of Compound 59 (376 MHz, $CDCl_3$):

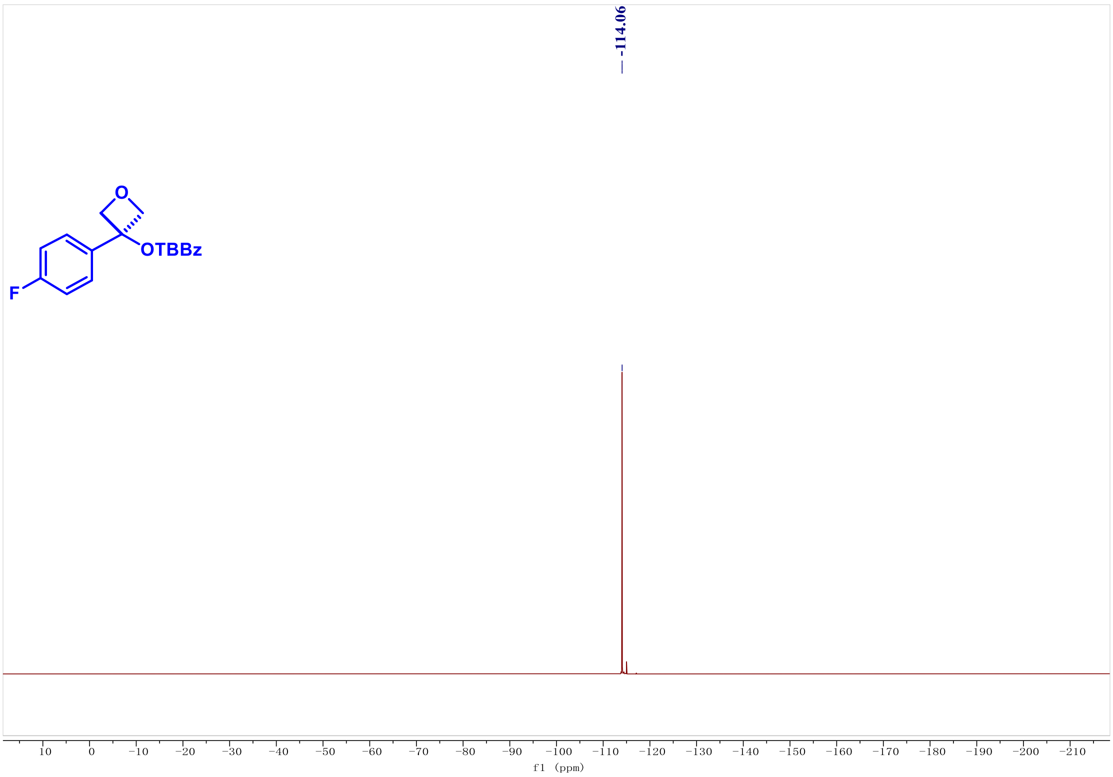

^{1}H NMR of Compound 61 (400 MHz, $CDCl_3$):

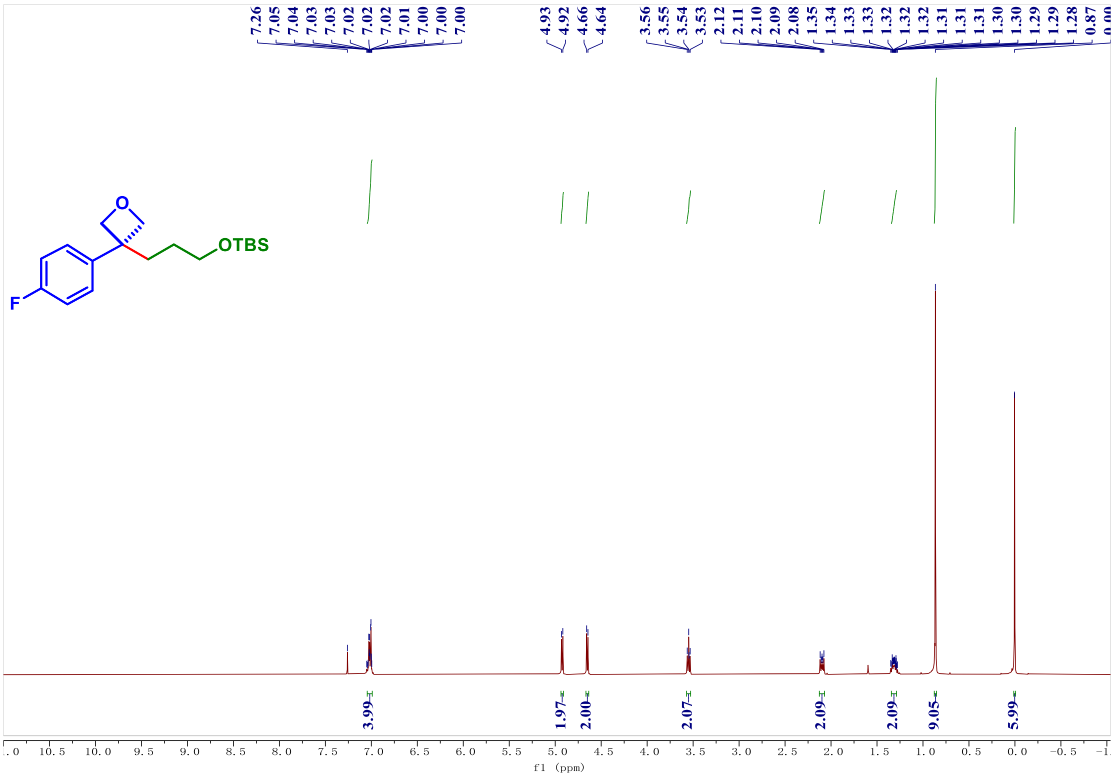

^{13}C NMR of Compound 61 (101 MHz, $CDCl_3$):

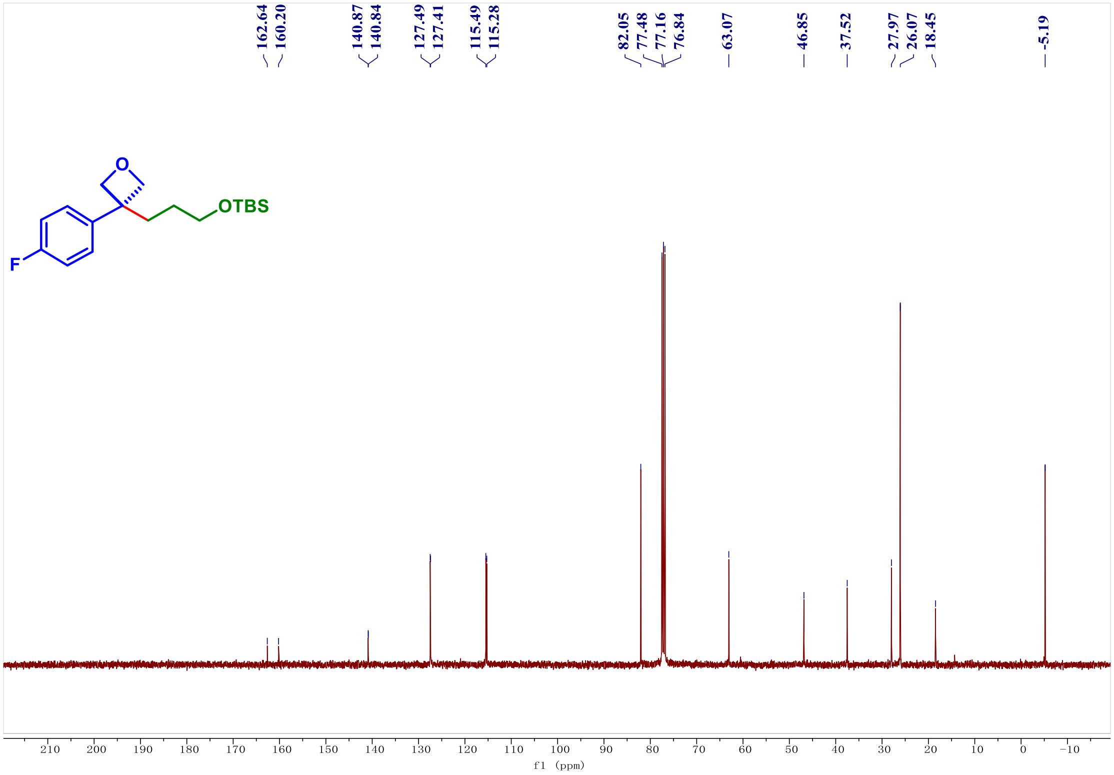

^{19}F NMR of Compound 61 (376 MHz, CDCl$_3$):

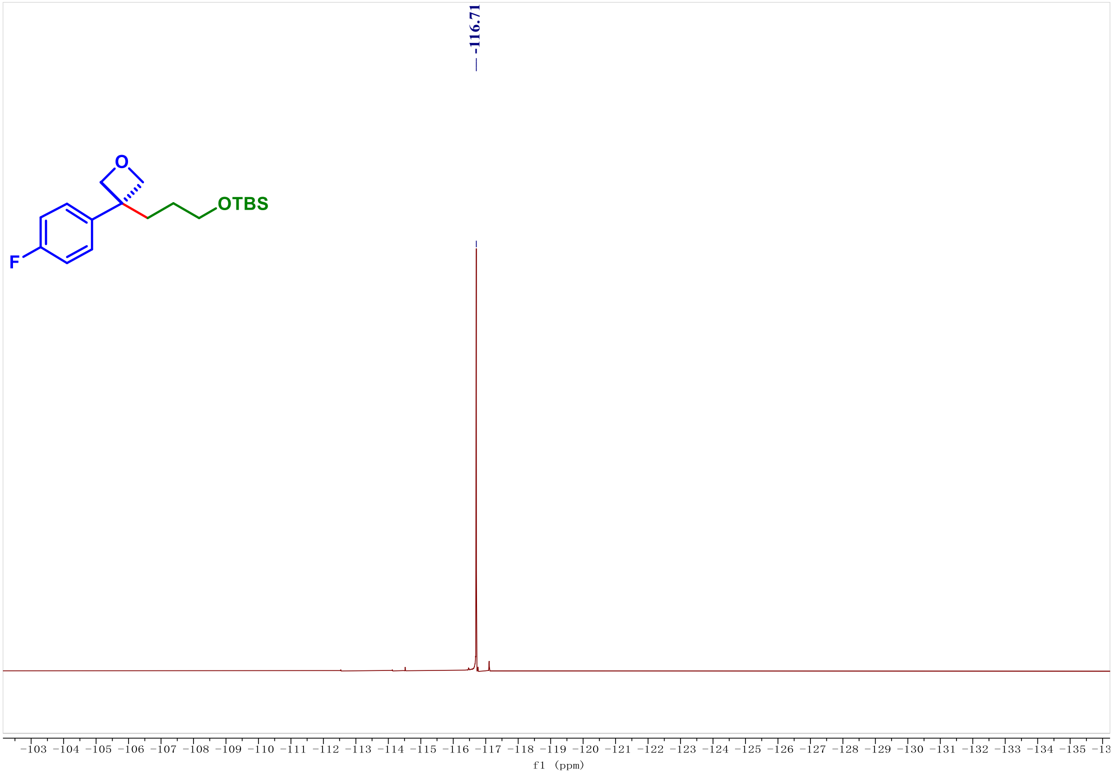

^{1}H NMR of Compound 63 (400 MHz, $CDCl_3$):

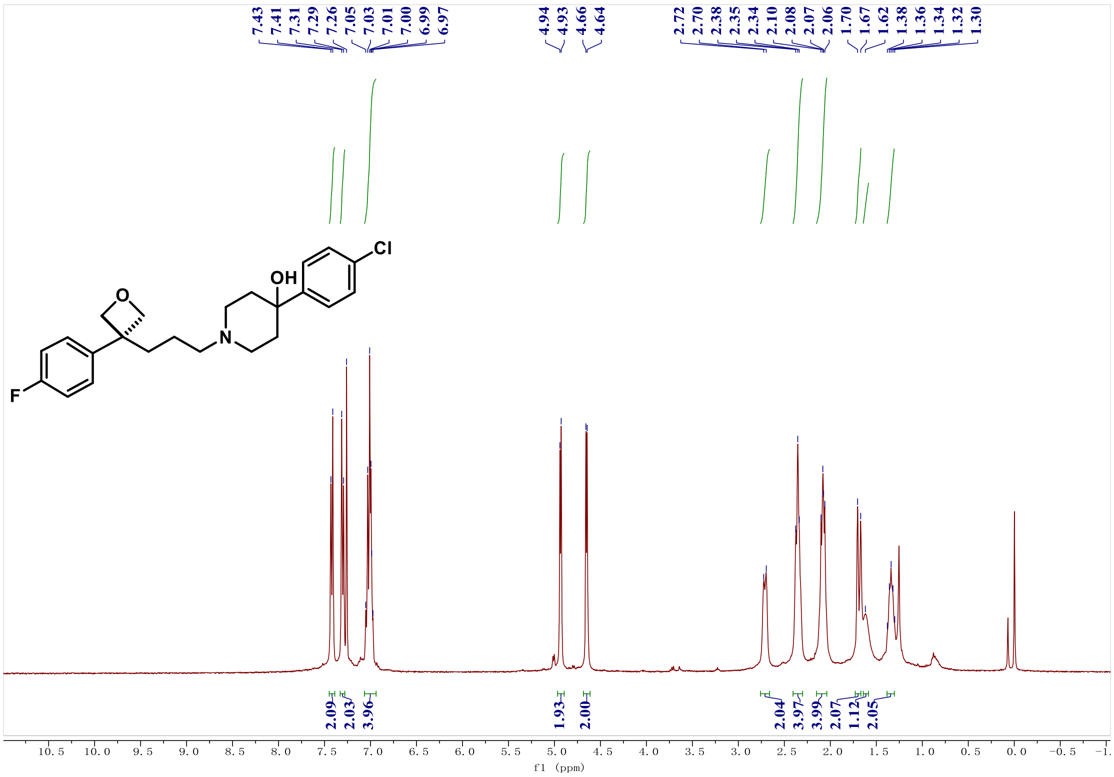

^{13}C NMR of Compound 63 (101 MHz, $CDCl_3$):

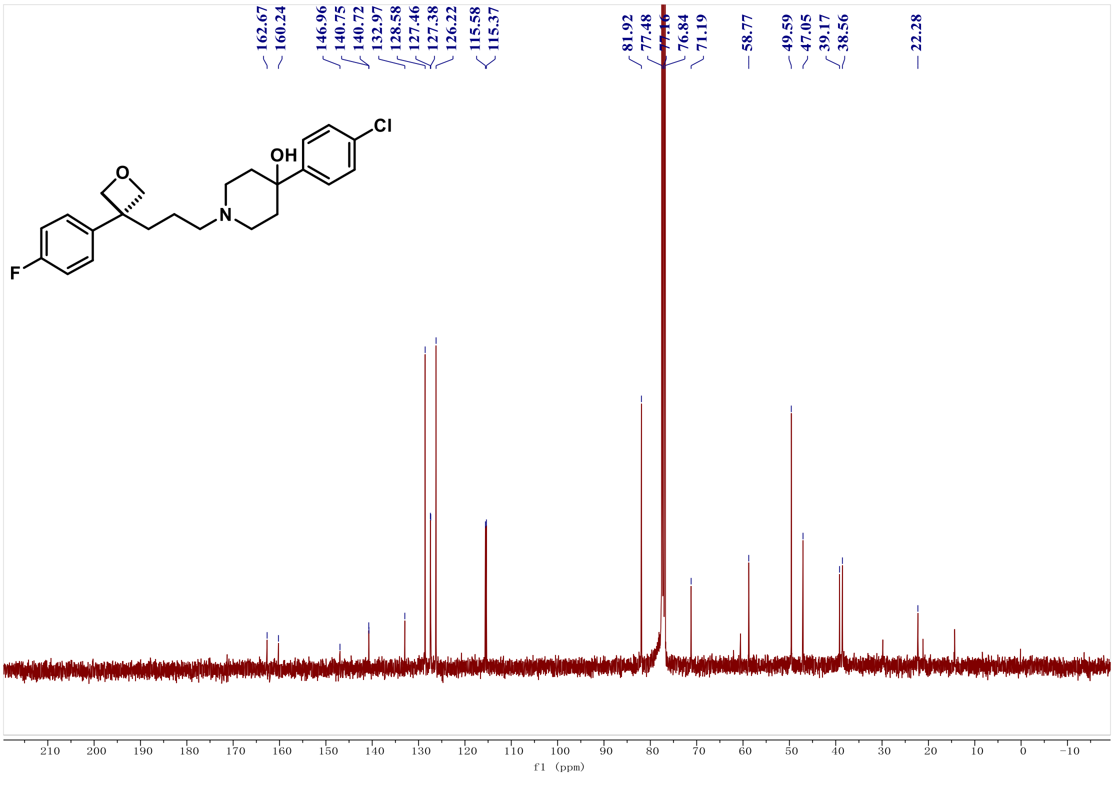

^{19}F NMR of Compound 63 (376 MHz, $CDCl_3$):

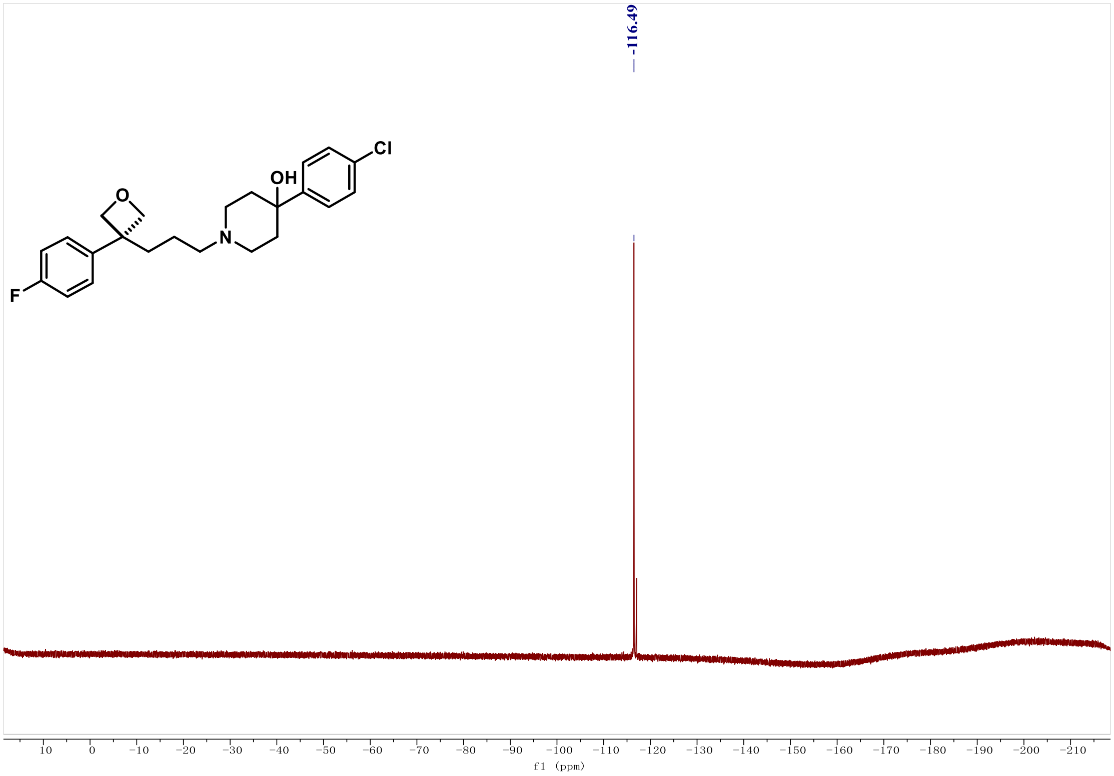

^{1}H NMR of Compound 65 (500 MHz, $CDCl_3$):

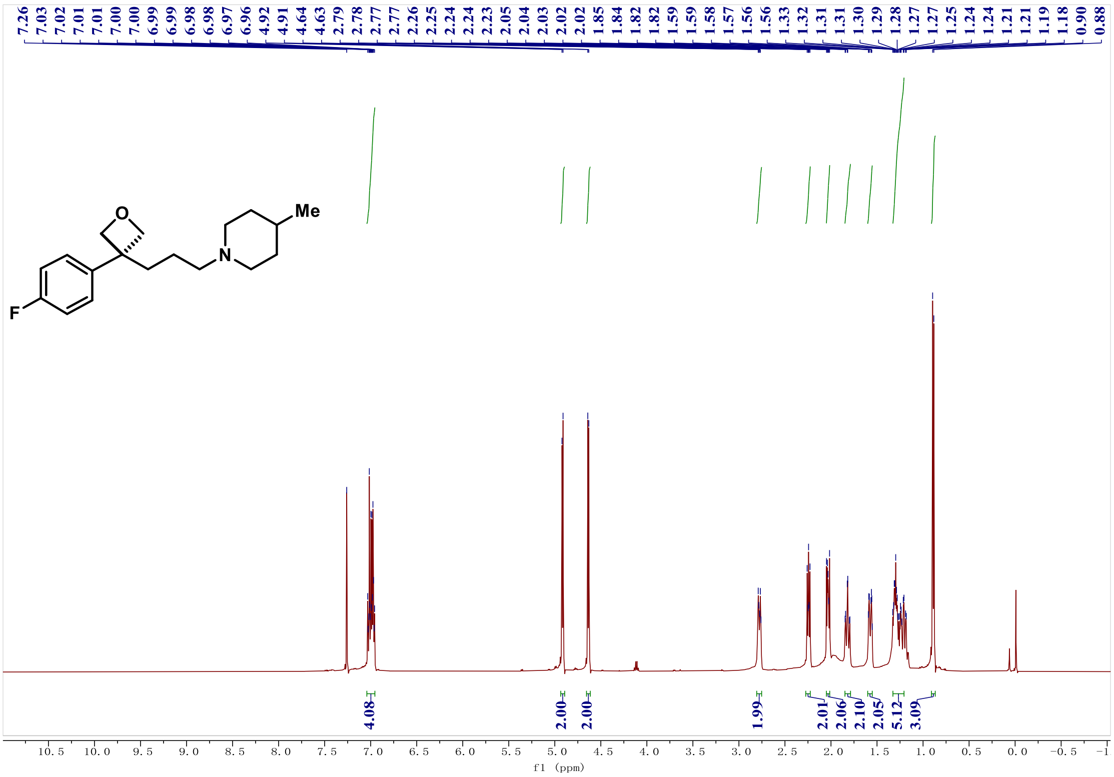

^{13}C NMR of Compound 65 (126 MHz, $CDCl_3$):

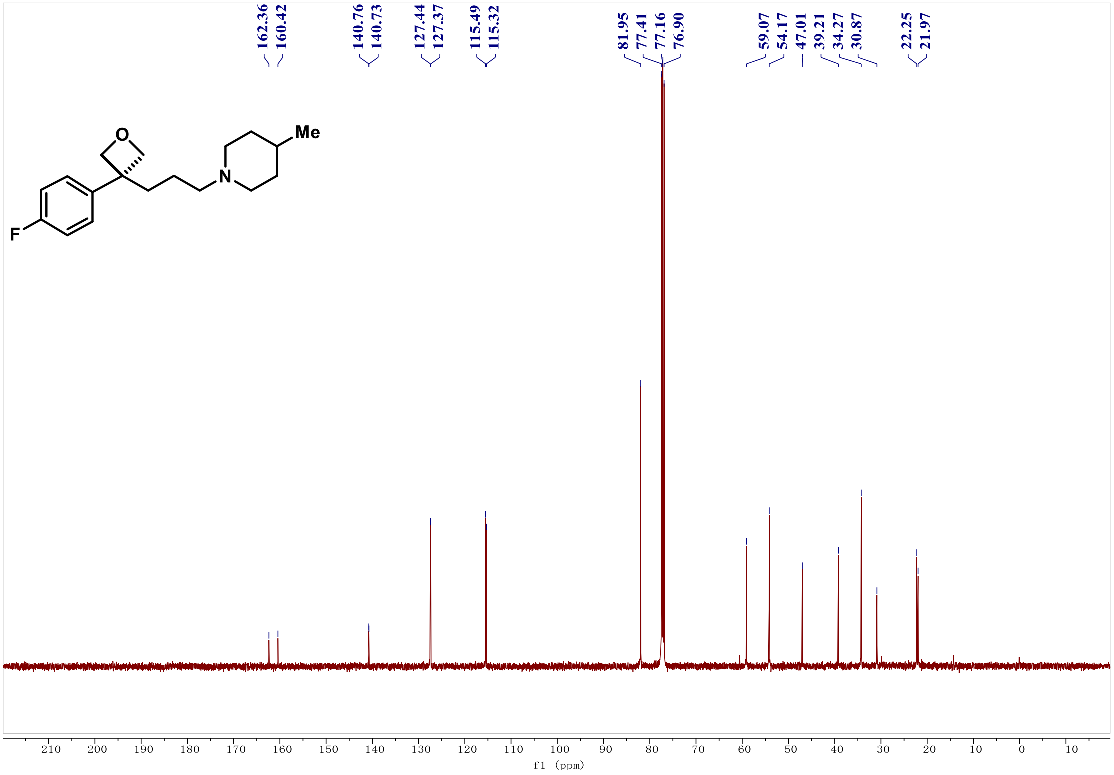

^{19}F NMR of Compound 65(471 MHz, $CDCl_3$):

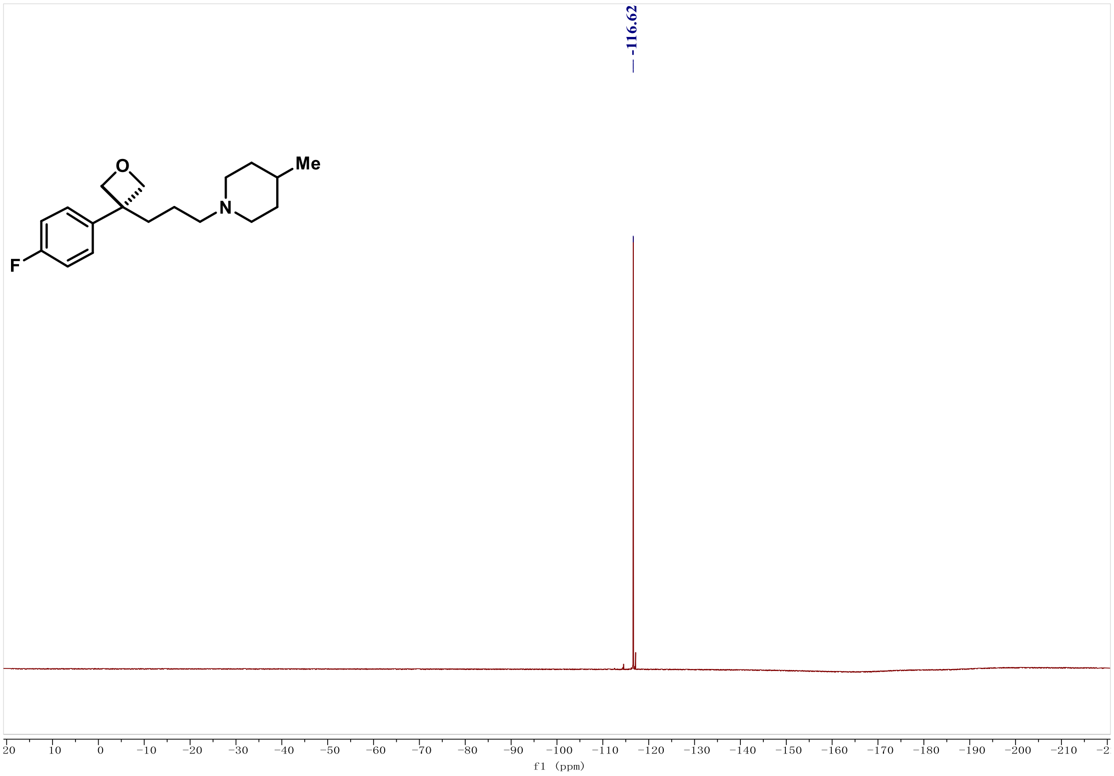

^{1}H NMR of Compound 66 (500 MHz, $CDCl_3$):

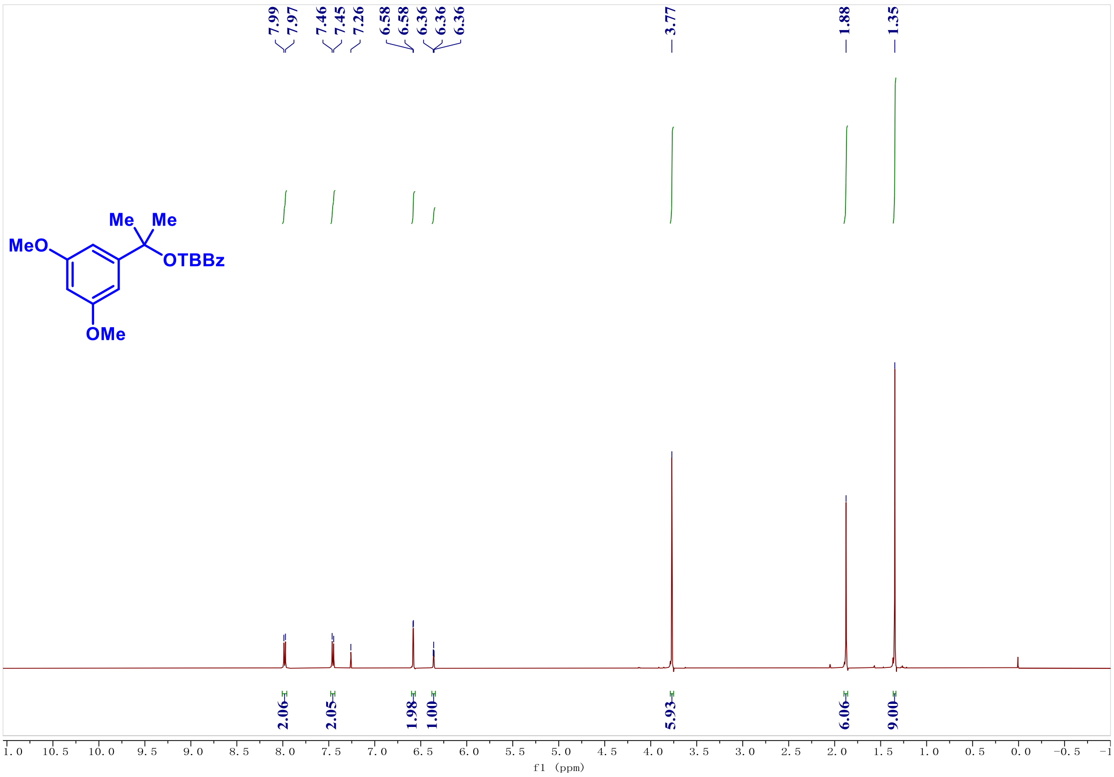

^{13}C NMR of Compound 66 (126 MHz, $CDCl_3$):

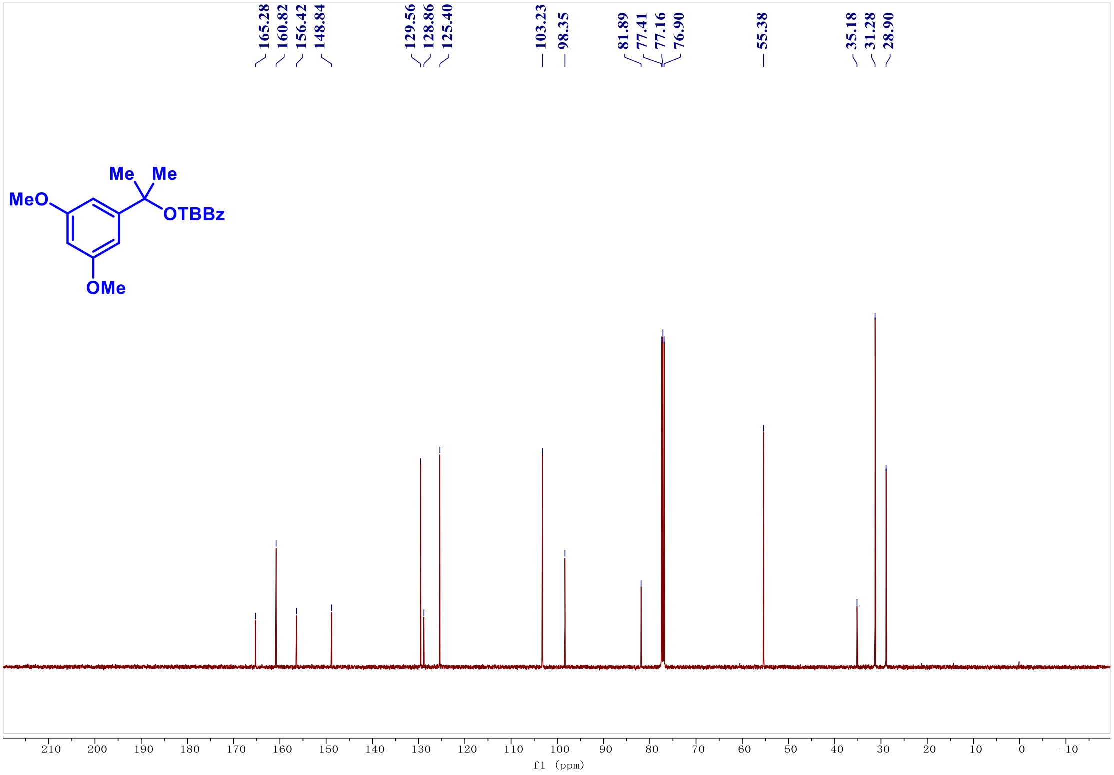

^{1}H NMR of Compound 68 (400 MHz, $CDCl_3$):

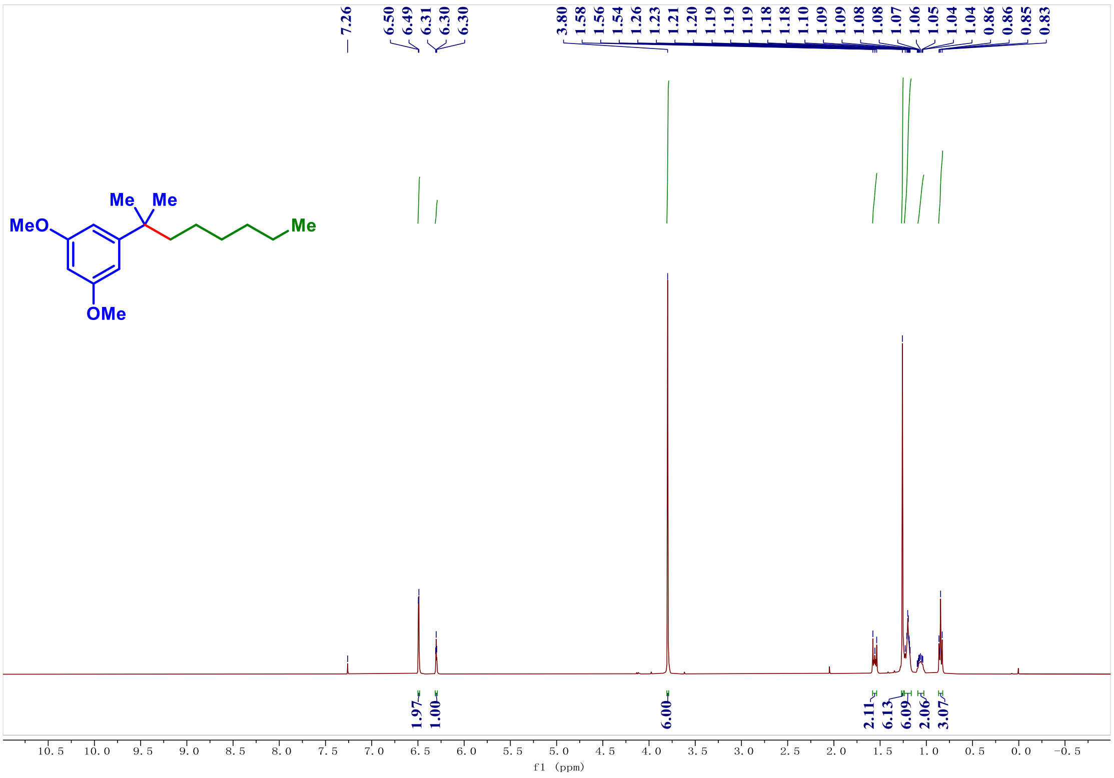

^{13}C NMR of Compound 68 (101 MHz, $CDCl_3$):

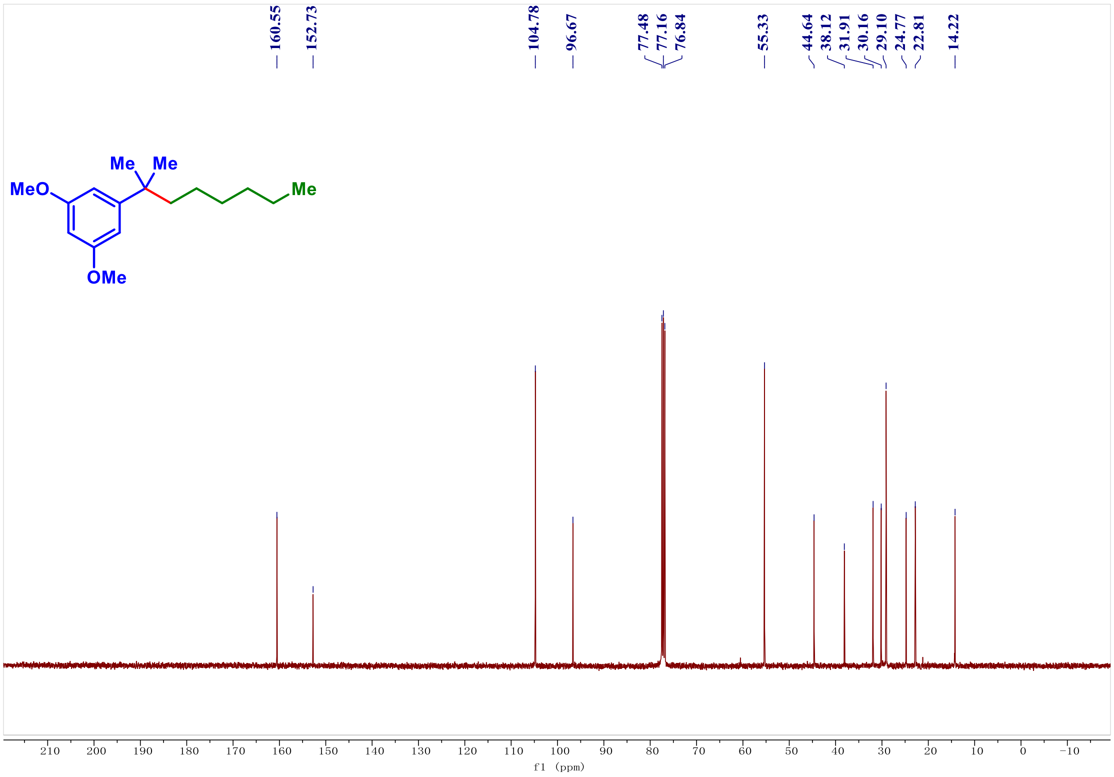

^{1}H NMR of Compound 69 (500 MHz, $CDCl_3$):

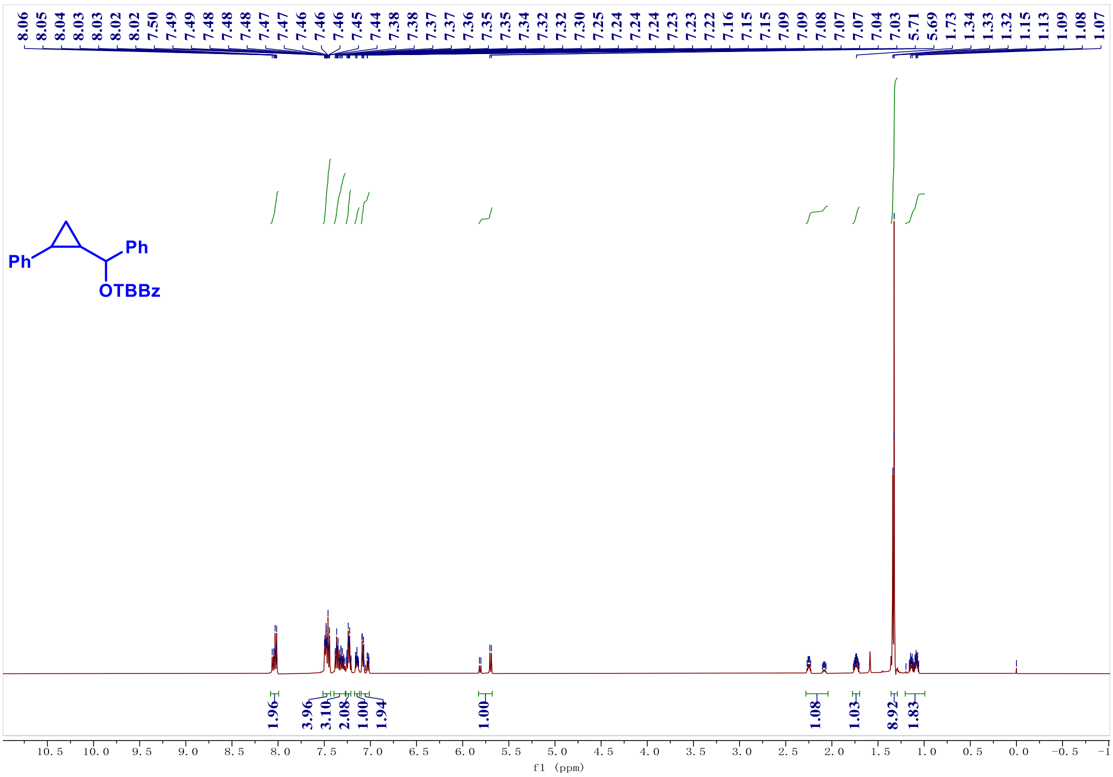

^{13}C NMR of Compound 69 (126 MHz, $CDCl_3$):

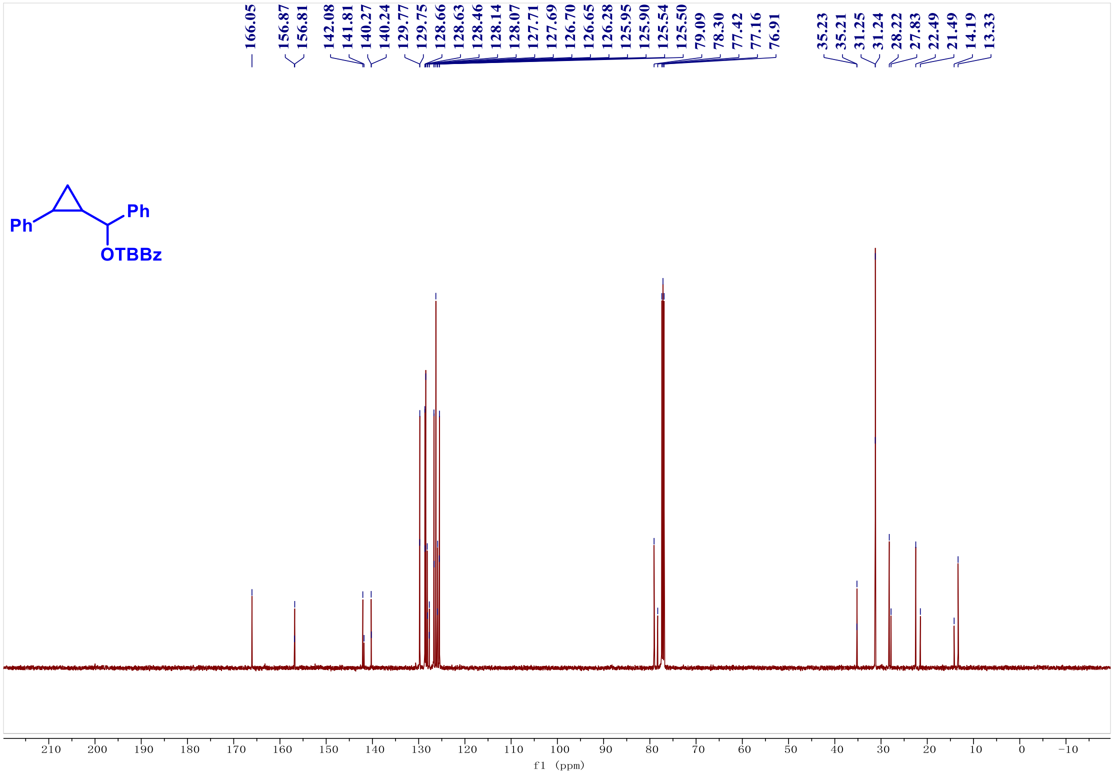

^{1}H NMR of Compound 71 (500 MHz, $CDCl_3$):

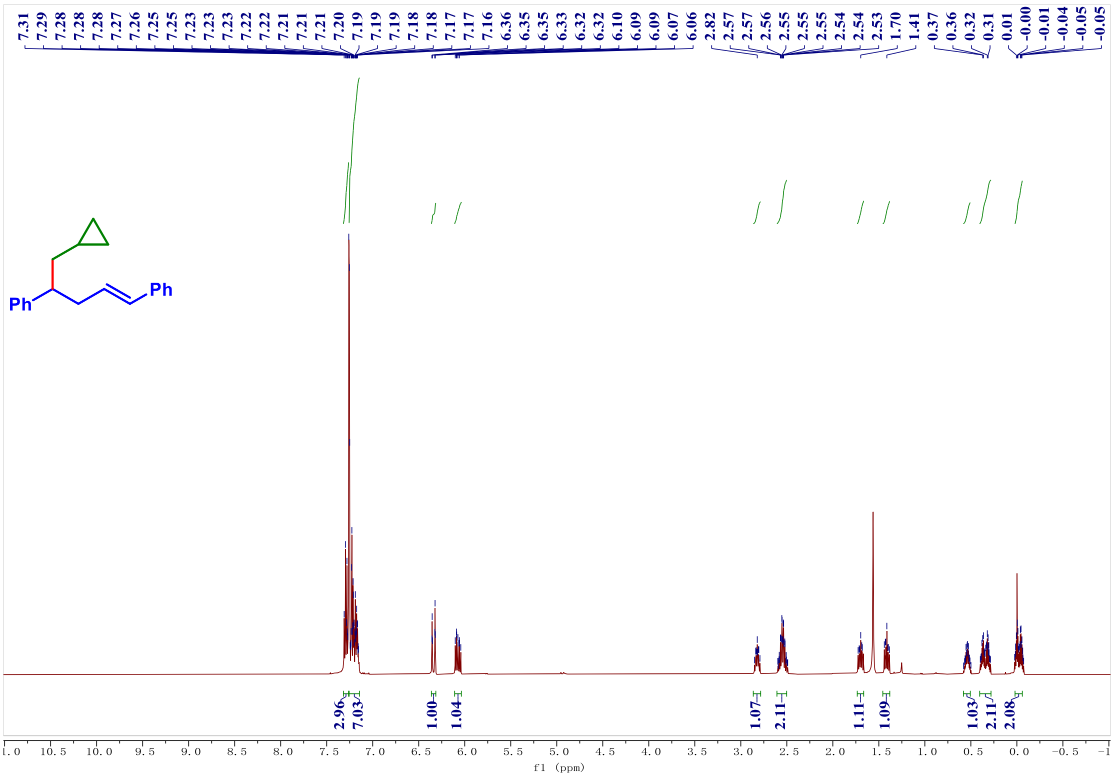

^{13}C NMR of Compound 71 (126 MHz, $CDCl_3$):

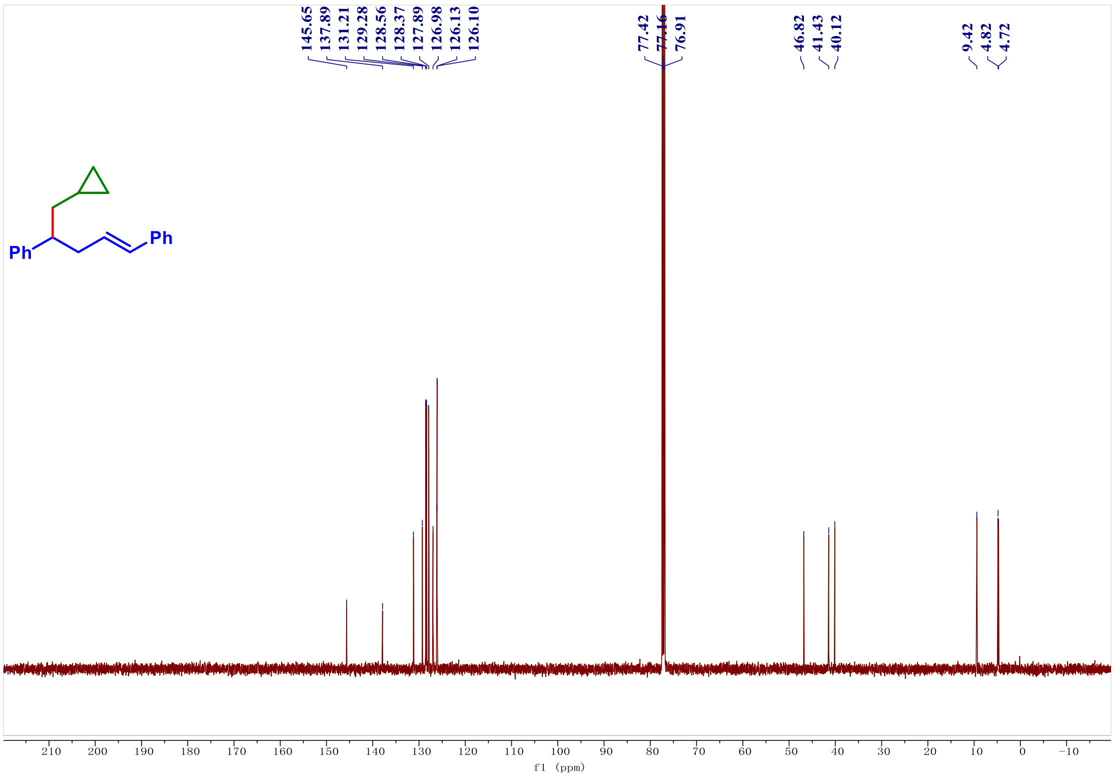

^{1}H NMR of Compound 73 (500 MHz, $CDCl_3$):

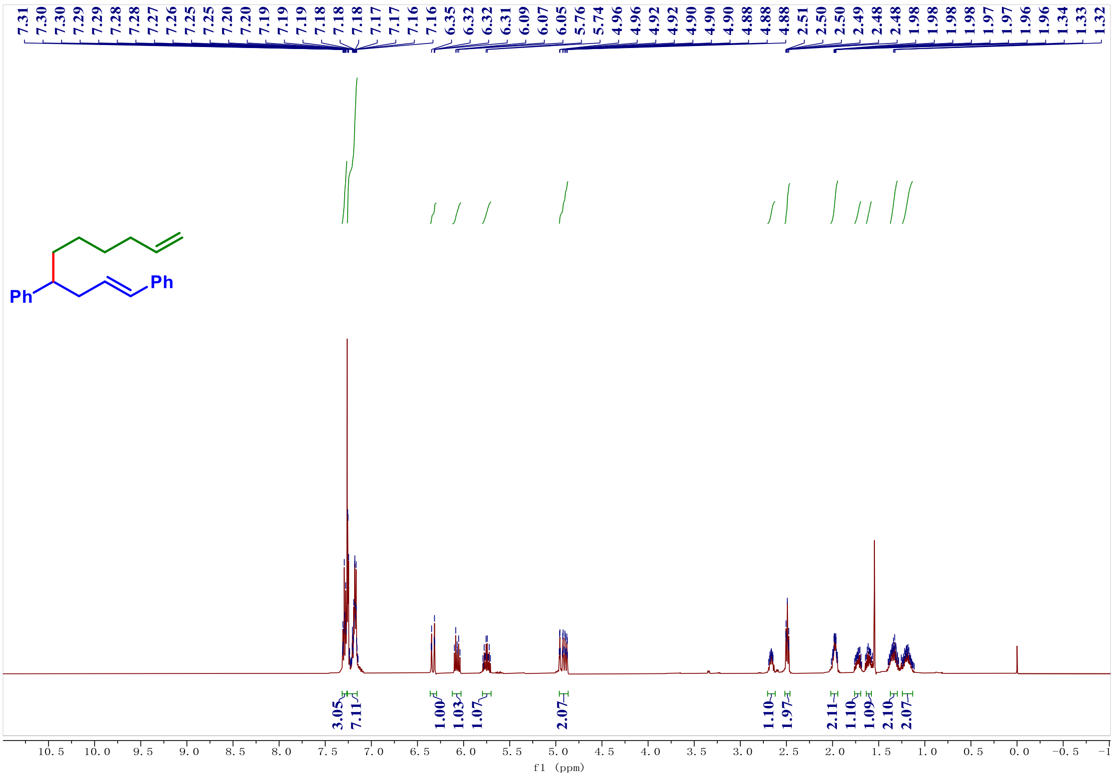

^{13}C NMR of Compound 73 (126 MHz, $CDCl_3$):

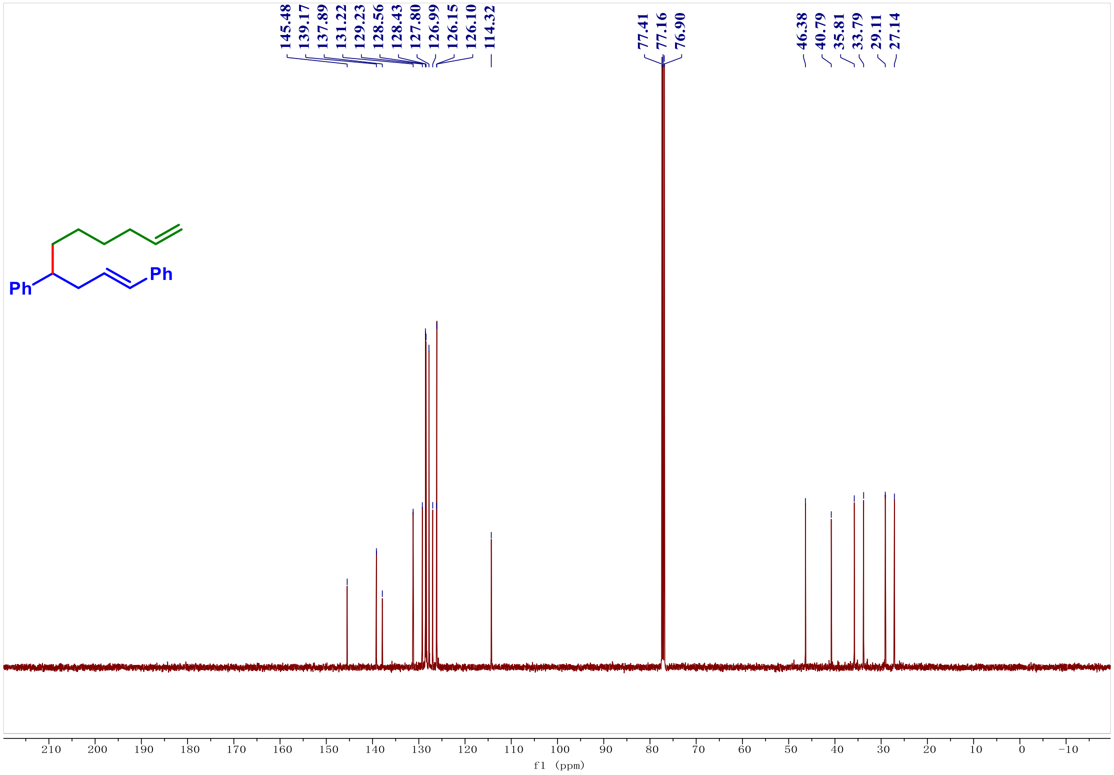

^{1}H NMR of Compound 7-*d* (500 MHz, $CDCl_3$):

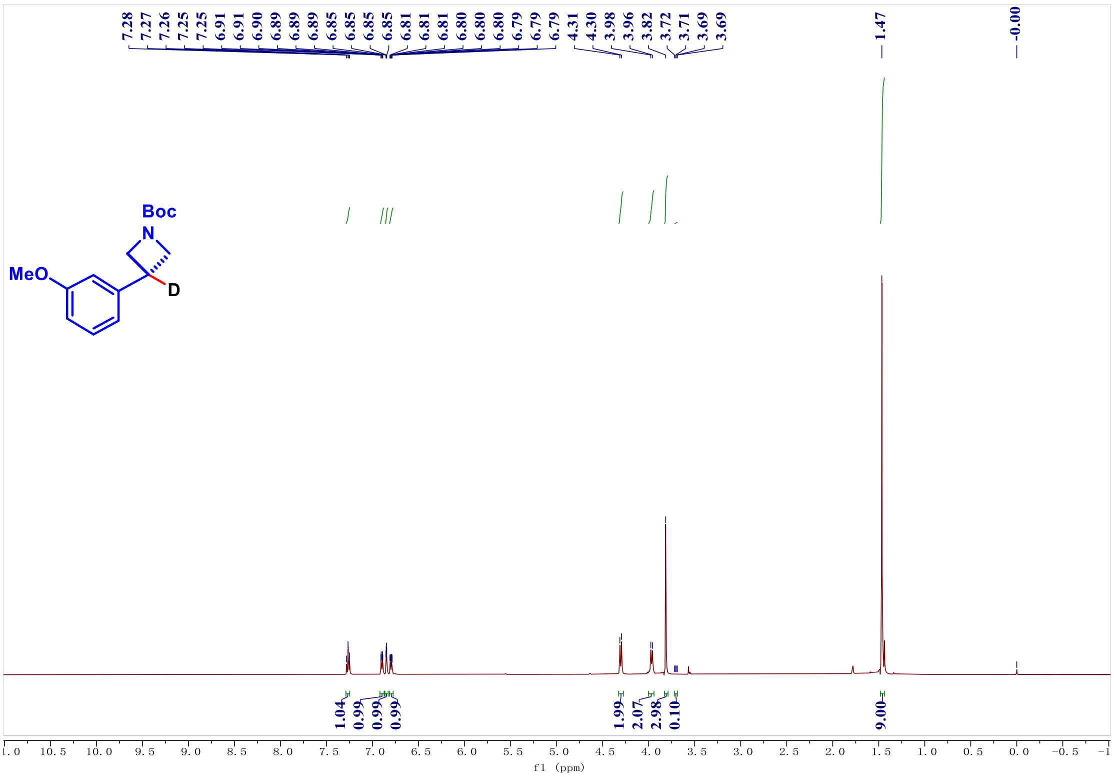

^{13}C NMR of Compound 7-*d* (101 MHz, $CDCl_3$):

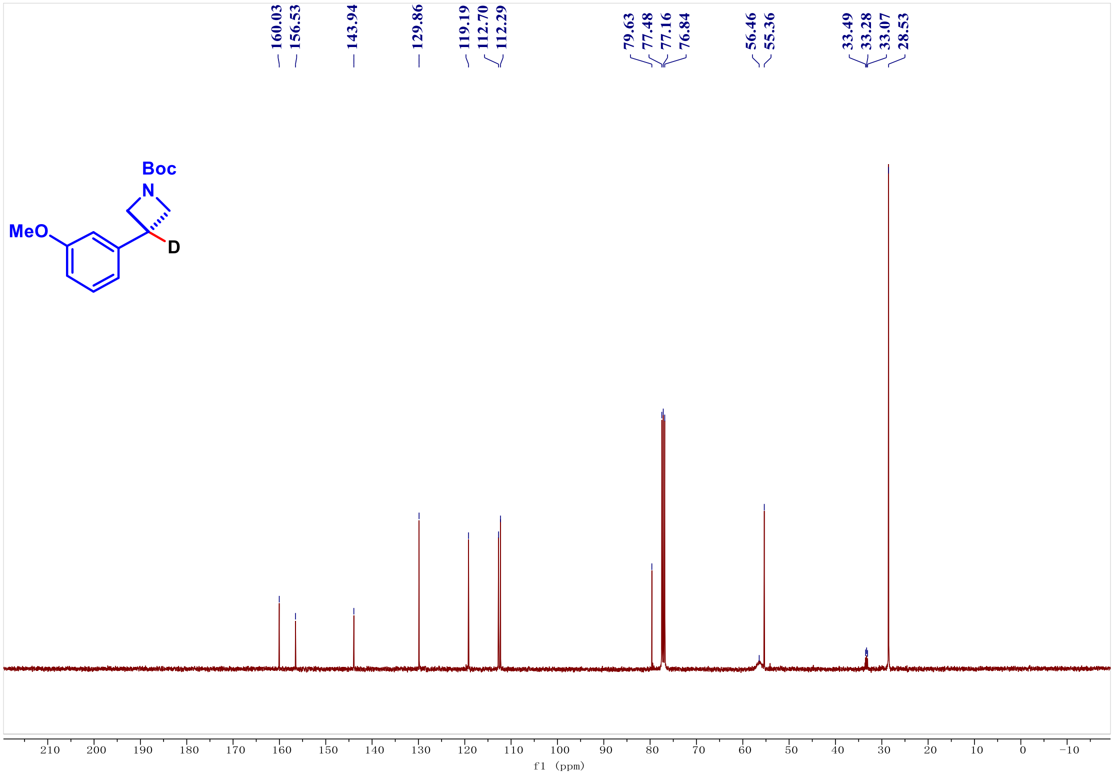

REFERENCES

1. J. Boström, D. G. Brown, R. J. Young, G. M. Keserü, Expanding the medicinal chemistry synthetic toolbox. *Nat. Rev. Drug Discov.* **17**, 709–727 (2018).

2. R. Jana, T. P. Pathak, M. S. Sigman, Advances in transition metal (Pd, Ni, Fe)-catalyzed cross-coupling reactions using alkyl-organometallics as reaction partners. *Chem. Rev.* **111**, 1417–1492 (2011).

3. D. A. Everson, D. J. Weix, Cross-electrophile coupling: Principles of reactivity and selectivity. *J. Org. Chem.* **79**, 4793–4798 (2014).

4. X. Wang, Y. Dai, H. Gong, Nickel-catalyzed reductive couplings. *Top. Curr. Chem.* **374**, 43 (2016).

5. L. E. Ehehalt, O. M. Beleh, I. C. Priest, J. M. Mouat, A. K. Olszewski, B. N. Ahern, A. R. Cruz, B. K. Chi, A. J. Castro, K. Kang, J. Wang, D. J. Weix, Cross-electrophile coupling: Principles, methods, and applications in synthesis. *Chem. Rev.* **124**, 13397–13569 (2024).

6. W. Zhang, L. Lu, W. Zhang, Y. Wang, S. D. Ware, J. Mondragon, J. Rein, N. Strotman, D. Lehnherr, K. A. See, S. Lin, Electrochemically driven cross-electrophile coupling of alkyl halides. *Nature* **604**, 292–297 (2022).

7. X. Tao, W. Lee, Z. Xu, H. Shu, Q. Wang, S. Ni, Y. Pan, S. Hong, Y. Wang, Reductive deaminative cross-coupling of alkyl bistriflimides enabled by electrocatalysis. *Sci. Adv.* **10**, eads5410 (2024).

8. H. Fu, J. Cao, T. Qiao, Y. Qi, S. J. Charnock, S. Garfinkle, T. K. Hyster, An asymmetric sp^3–sp^3 cross-electrophile coupling using ‘ene’-reductases. *Nature* **610**, 302–307 (2022).

9. S. Roediger, E. Le Saux, P. Boehm, B. Morandi, Coupling of unactivated alkyl electrophiles using frustrated ion pairs. *Nature* **636**, 108–114 (2024).

10. Y. Liu, P. Li, Y. Wang, Y. Qiu, Electroreductive cross-electrophile coupling (eXEC) reactions. *Angew. Chem. Int. Ed. Engl.* **62**, e202306679 (2023).

11. J. Lei, S. Yu, Z.-G. Xu, Cross-electrophile couplings (XECs) between similar electrophile reagents. *Chin. J. Chem.* **42**, 3518–3532 (2024).

12. W. Zhang, W. Guan, J. I. Martinez Alvarado, L. F. T. Novaes, S. Lin, Deep electroreductive chemistry: Harnessing carbon- and silicon-based reactive intermediates in organic synthesis. *ACS Catal.* **13**, 8038–8048 (2023).

13. L. Cheng, Q. Lin, Y. Chen, H. Gong, Recent progress on transition-metal-mediated reductive C(sp^3)–O bond radical addition and coupling reactions. *Synthesis* **54**, 4426–4446 (2022).

14. P. Villo, A. Shatskiy, M. D. Kärkäs, H. Lundberg, Electrosynthetic C−O bond activation in alcohols and alcohol derivatives. *Angew. Chem. Int. Ed. Engl.* **62**, e202211952 (2023).

15. Y. Wang, J. Xu, Y. Pan, Y. Wang, Recent advances in electrochemical deoxygenation reactions of organic compounds. *Org. Biomol. Chem.* **21**, 1121–1133 (2023).

16. X. Pang, X.-Z. Shu, Reductive deoxygenative functionalization of alcohols by first-row transition metal catalysis. *Chin. J. Chem.* **41**, 1637–1652 (2023).

17. T. Mandal, S. Mallick, M. Islam, S. D. Sarkar, Alcohols as alkyl synthons enabled by photoredox-catalyzed deoxygenative activation. *ACS Catal.* **14**, 13451–13496 (2024).

18. A. Cook, S. G. Newman, Alcohols as substrates in transition-metal-catalyzed arylation, alkylation, and related reactions. *Chem. Rev.* **124**, 6078–6144 (2024).

19. Y. Yuan, L. Liu, F. Zhang, Y.-N. Zhang, C. Huo, Recent advances in electrochemical deoxygenative functionalization of alcohols and their derivatives. *Tetrahedron Chem.* **14**, 100126 (2025).

20. A. J. Ressler, J. I. Martinez-Alvarado, R. Hariharan, W. Guan, S. Lin, Deoxygenative functionalization of alcohols and carbonyl compounds *via* electrochemical reduction. *Angew. Chem. Int. Ed. Engl.* **64**, e202510069 (2025).

21. P. Villo, M. Lill, Z. Fan, K. Breitwieser, J. White, S. P. Morente, M. Ahlquist, H. Lundberg, Electrochemical deoxygenative silylation of alcohols. *Angew. Chem. Int. Ed. Engl.* **64**, e202508697 (2025).

22. Y.-X. Zheng, Y.-X. Wu, L.-J. Su, P. Xiong, H.-C. Xu, Harnessing electrochemistry for direct deoxygenative silylation of alcohols and ketones. *Angew. Chem. Int. Ed. Engl.* **64**, e202509411 (2025).

23. W. Guan, Y. Chang, S. Lin, Electrochemically driven deoxygenative borylation of alcohols and carbonyl compounds. *J. Am. Chem. Soc.* **145**, 16966–16972 (2023).

24. P. Villo, M. Lill, Z. Alsaman, A. S. Kronberg, V. Chu, G. Ahumada, H. Agarwala, M. Ahlquist, H. Lundberg, Electroreductive deoxygenative C−H and C−C bond formation from non-derivatized alcohols fueled by anodic borohydride oxidation. *ChemElectroChem* **10**, e202300420 (2023).

25. H. Senboku, K. Yoneda, S. Hara, Electrochemical direct carboxylation of benzyl alcohols having an electron-withdrawing group on the phenyl ring: One-step formation of phenylacetic acids from benzyl alcohols under mild conditions. *Tetrahedron Lett.* **56**, 6772–6776 (2015).

26. M. Ohkoshi, J. Michinishi, S. Hara, H. Senboku, Electrochemical carboxylation of benzylic carbonates: Alternative method for efficient synthesis of arylacetic acids. *Tetrahedron* **66**, 7732–7737 (2010).

27. H. Senboku, K. Sakai, A. Fukui, Y. Sato, Y. Yamauchi, Efficient synthesis of mandel acetates by electrochemical carboxylation of benzal diacetates. *ChemElectroChem* **6**, 4158–4164 (2019).

28. H. Wang, Z. Wang, G. Zhao, V. Ramadoss, L. Tian, Y. Wang, Electrochemical deoxygenative Barbier-type reaction. *Org. Lett.* **24**, 3668–3673 (2022).

29. J. Kang, H. Cho, H. Kim, Electroreductive formylation of activated alcohols *via* radical–polar crossover. *Chem. Commun.* **59**, 5733–5736 (2023).

30. X. Tian, T. A. Karl, S. Reiter, S. Yakubov, R. de Vivie-Riedle, B. König, J. P. Barham, Electro-mediated photoredox catalysis for selective C(sp^3)–O cleavages of phosphinated alcohols to carbanions. *Angew. Chem. Int. Ed. Engl.* **60**, 20817–20825 (2021).

31. Z. Tan, H. Zhang, K. Xu, C. Zeng, Electrochemical radical–polar crossover: A radical approach to polar chemistry. *Sci. China Chem.* **67**, 450–470 (2024).

32. P. Li, Y. Qiu, Reductive electrophilic cross-coupling for constructing C(sp^3)–C(sp^3) bonds. *Synlett* **36**, 438–444 (2025).

33. D. Bishop, J. F. Cavadla, I. M. Lockhart, M. Wright, Analgetics based on the azetidine ring. *J. Med. Chem.* **11**, 466–470 (1968).

34. Y. Ye, H. Chen, J. L. Sessler, H. Gong, Zn-mediated fragmentation of tertiary alkyl oxalates enabling formation of alkylated and arylated quaternary carbon centers. *J. Am. Chem. Soc.* **141**, 820–824 (2019).

35. A. Joshi-Pangu, C.-Y. Wang, M. R. Biscoe, Nickel-catalyzed Kumada cross-coupling reactions of tertiary alkylmagnesium halides and aryl bromides/triflates. *J. Am. Chem. Soc.* **133**, 8478–8481 (2011).

36. C. Lohre, T. Dröge, C. Wang, F. Glorius, Nickel-catalyzed cross-coupling of aryl bromides with tertiary Grignard reagents utilizing donor-functionalized N-heterocyclic carbenes (NHCs). *Chem. A Eur. J.* **17**, 6052–6055 (2011).

37. S. L. Zultanski, G. C. Fu, Nickel-catalyzed carbon–carbon bond-forming reactions of unactivated tertiary alkyl halides: Suzuki arylations. *J. Am. Chem. Soc.* **135**, 624–627 (2013).

38. X. Wang, S. Wang, W. Xue, H. Gong, Nickel-catalyzed reductive coupling of aryl bromides with tertiary alkyl halides. *J. Am. Chem. Soc.* **137**, 11562–11565 (2015).

39. D. N. Primer, G. A. Molander, Enabling the cross-coupling of tertiary organoboron nucleophiles through radical-mediated alkyl transfer. *J. Am. Chem. Soc.* **139**, 9847–9850 (2017).

40. S. A. Green, S. Vásquez-Céspedes, R. A. Shenvi, Iron–nickel dual catalysis: A new engine for olefin functionalization and the formation of quaternary centers. *J. Am. Chem. Soc.* **140**, 11317–11324 (2018).

41. X. Wang, G. Ma, Y. Peng, C. E. Pitsch, B. J. Moll, T. D. Ly, X. Wang, H. Gong, Ni-catalyzed reductive coupling of electron-rich aryl iodides with tertiary alkyl halides. *J. Am. Chem. Soc.* **140**, 14490–14497 (2018).

42. T.-G. Chen, H. Zhang, P. K. Mykhailiuk, R. R. Merchant, C. A. Smith, T. Qin, P. S. Baran, Quaternary centers by nickel-catalyzed cross-coupling of tertiary carboxylic acids and (hetero)aryl zinc reagents. *Angew. Chem. Int. Ed. Engl.* **58**, 2454–2458 (2019).

43. M. Yuan, Z. Song, S. O. Badir, G. A. Molander, O. Gutierrez, On the nature of $C(sp^3)$–$C(sp^2)$ bond formation in nickel-catalyzed tertiary radical cross-couplings: A case study of Ni/photoredox catalytic cross-coupling of alkyl radicals and aryl halides. *J. Am. Chem. Soc.* **142**, 7225–7234 (2020).

44. J. G. Estrada, W. L. Williams, S. I. Ting, A. G. Doyle, Role of electron-deficient olefin ligands in a Ni-catalyzed aziridine cross-coupling to generate quaternary carbons. *J. Am. Chem. Soc.* **142**, 8928–8937 (2020).

45. D. C. Salgueiro, B. K. Chi, I. A. Guzei, P. García-Reynaga, D. J. Weix, Control of redox-active ester reactivity enables a general cross-electrophile approach to access arylated strained rings. *Angew. Chem. Int. Ed. Engl.* **61**, e202205673 (2022).

46. J. Guo, D. Norris, A. Ramirez, J. L. Sloane, E. M. Simmons, J. M. Ganley, M. S. Oderinde, T. G. Murali Dhar, G. H. M. Davies, T. C. Sherwood, Unlocking tertiary acids for metallaphotoredox $C(sp^2)$–$C(sp^3)$ decarboxylative cross-couplings. *ACS Catal.* **13**, 11910–11918 (2023).

47. X. Ying, Y. Li, L. Li, C. Li, Nickel-catalyzed C–I-selective $C(sp^2)$–$C(sp^3)$ cross-electrophile coupling of bromo(iodo)arenes with alkyl bromides. *Angew. Chem. Int. Ed. Engl.* **62**, e202304177 (2023).

48. G. Laudadio, P. Neigenfind, Á. Péter, C. Z. Rubel, M. A. Emmanuel, M. S. Oderinde, T. El-Hayek Ewing, M. D. Palkowitz, J. L. Sloane, K. W. Gillman, D. Ridge, M. D. Mandler, P. N. Bolduc, M. C. Nicastri, B. Zhang, S. Clementson, N. N. Petersen, P. Martin-Gago, P. Mykhailiuk, K. M. Engle, P. S. Baran, Nickel-electrocatalytic decarboxylative arylation to access quaternary centers. *Angew. Chem. Int. Ed. Engl.* **63**, e202314617 (2024).

49. J. R. Dorsheimer, M. A. Ashley, T. Rovis, Dual nickel/photoredox-catalyzed deaminative cross-coupling of sterically hindered primary amines. *J. Am. Chem. Soc.* **143**, 19294–19299 (2021).

50. T. T. Talele, Opportunities for tapping into three-dimensional chemical space through a quaternary carbon. *J. Med. Chem.* **63**, 13291–13315 (2020).

51. W. Xue, X. Jia, X. Wang, X. Tao, Z. Yin, H. Gong, Nickel-catalyzed formation of quaternary carbon centers using tertiary alkyl electrophiles. *Chem. Soc. Rev.* **50**, 4162–4184 (2021).

52. R. Chen, N. E. Intermaggio, J. Xie, J. A. Rossi-Ashton, C. A. Gould, R. T. Martin, J. Alcázar, D. W. C. MacMillan, Alcohol–alcohol cross-coupling enabled by S_H2 radical sorting. *Science* **383**, 1350–1357 (2024).

53. X.-B. Yan, C.-L. Li, W.-J. Jin, P. Guo, X.-Z. Shu, Reductive coupling of benzyl oxalates with highly functionalized alkyl bromides by nickel catalysis. *Chem. Sci.* **9**, 4529–4534 (2018).

54. K. Lam, I. E. Markó, Chemoselective chemical and electrochemical deprotections of aromatic esters. *Org. Lett.* **11**, 2752–2755 (2009).

55. K. Lam, I. E. Markó, Organic electrosynthesis using toluates as simple and versatile radical precursors. *Chem. Commun.* **1**, 95–97 (2009).

56. K. Lam, I. E. Markó, Using toluates as simple and versatile radical precursors. *Org. Lett.* **10**, 2773–2776 (2008).

57. C. E. Dahm, D. G. Peters, Electrochemical reduction of tetraalkylammonium tetrafluoroborates at carbon cathodes in dimethylformamide. *J. Electroanal. Chem.* **402**, 91–96 (1996).

58. F. Lovering, J. Bikker, C. Humblet, Escape from flatland: Increasing saturation as an approach to improving clinical success. *J. Med. Chem.* **52**, 6752–6756 (2009).

59. W. Wei, S. Cherukupalli, L. Jing, X. Liu, P. Zhan, Fsp^3: A new parameter for drug-likeness. *Drug Discov. Today* **25**, 1839–1845 (2020).

60. J. Tsien, C. Hu, R. R. Merchant, T. Qin, Three-dimensional saturated $C(sp^3)$-rich bioisosteres for benzene. *Nat. Rev. Chem.* **8**, 605–627 (2024).

61. R. Zhao, Z. Lin, I. Maksso, J. Struwe, L. Ackermann, Electrochemical cross-electrophile-coupling for transition metal-free allylic carboxylation with ambient CO_2. *ChemElectroChem* **9**, e202200989 (2022).

62. T. Nishikata, S. Ishida, R. Fujimoto, Site-selective tertiary alkyl–fluorine bond formation from α-bromoamides using a copper/CsF catalyst system. *Angew. Chem. Int. Ed. Engl.* **55**, 10008–10012 (2016).

63. Q. Qian, Z. Zang, S. Wang, Y. Chen, K. Lin, H. Gong, Nickel-catalyzed reductive cross-coupling of aryl halides. *Synlett* **24**, 619–624 (2013).

64. Q. Zhang, X. Wang, Q. Qian, H. Gong, Nickel-catalyzed reductive cross-coupling of benzyl halides with aryl halides. *Synthesis* **48**, 2829–2836 (2016).

65. D. R. Parmar, J. Y. Soni, R. Guduru, R. H. Rayani, R. V. Kusurkar, A. G. Vala, Azetidines of pharmacological interest. *Arch. Pharm.* **354**, e2100062 (2021).

66. J. J. Rojas, J. A. Bull, Oxetanes in drug discovery campaigns. *J. Med. Chem.* **66**, 12697–12709 (2023).

67. C.-M. Hsu, H.-B. Lin, X.-Z. Hou, R. V. P. T. Tapales, C.-K. Shih, S. Miñoza, Y.-S. Tsai, Z.-N. Tsai, C.-L. Chan, H.-H. Liao, Azetidines with all-carbon quaternary centers: Merging relay catalysis with strain release functionalization. *J. Am. Chem. Soc.* **145**, 19049–19059 (2023).

68. V. Jaiswal, S. Mondal, B. Singh, V. P. Singh, J. C. Saha, Cation-promoted strain-release-driven access to functionalized azetidines from azabicyclo[1.1.0]butanes. *Angew. Chem. Int. Ed. Engl.* **62**, e202304471 (2023).

69. L. Massaro, P. Neigenfind, A. Feng, G. Kuehn, F. C. Attard, A. DeSanti, M. R. Collins, M. Bravo, R. K. Twumasi, M. Nicastri, D. Chen, P. N. Bolduc, M. A. Emmanuel, M. S. Oderinde, M. D. Palkowitz, X. Zheng, A. C. Hunter, K. C. Harper, C. C. Tyrol, P. K. Mykhailiuk, Y. Kawamata, P. S. Baran, Triply convergent Ni-electrocatalytic assembly of 1,1-diaryl cyclobutanes, azetidines and oxetanes. *Nat. Chem.*, org/10.1038/s41557-025-01990-x (2025).

70. D. Tian, G. Chen, X. Wang, H.-J. Zhang, Modular access to functionalized oxetanes as benzoyl bioisosteres. *J. Am. Chem. Soc.* **146**, 18011–18018 (2024).

71. D. Tian, G. Chen, X. Xiao, X. Wang, H.-J. Zhang, Modular access to functionalized azetidines *via* electrophilic azetidinylation. *Org. Chem. Front.* **12**, 5226–5238 (2025).

72. R. C. Lemoine, A. C. Petersen, L. Setti, J. Wanner, A. Jekle, G. Heilek, A. deRosier, C. Ji, P. Berry, D. Rotstein, Evaluation of secondary amide replacements in a series of CCR5 antagonists as a means to increase intrinsic membrane permeability. Part 1: Optimization of gem-disubstituted azacycles. *Bioorg. Med. Chem. Lett.* **20**, 704–708 (2010).

73. M. Bolli, C. Brotschi, C. Lescop, Preparation of pyridin-3-yl derivatives as LPA1 receptor modulators. *Patent WO2020254408 A1* (2020).

74. M. Birker, C. Lescop, Combination of an azetidine LPA1 receptor antagonist with pirfenidone and/or nintedanib for use in the treatment of fibrotic diseases. *Patent WO2021110805 A1* (2021).

75. R. Davenport, F. Masse, A. Prud'homme, C. Bürki, P. Doerrwaechter, M. Künzli, F. D'Aiuto, M. H. Bolli, G. Schäfer, Large-scale synthesis of LPA1-receptor antagonist ACT-1016-0707. *Org. Process Res. Dev.* **28**, 577–587 (2024).

76. M. Härter, H. Beck, P. Ellinghaus, K. Berhörster, S. Greschat, K.-H. Thierauch, F. Süssmeier, Heterocyclically substituted aryl compounds as HIF inhibitors. *Patent US2013/196964 A1* (2013).

77. N. A. Meanwell, Applications of bioisosteres in the design of biologically active compounds. *J. Agric. Food Chem.* **71**, 18087–18122 (2023).

78. G. Wuitschik, M. Rogers-Evans, K. Müller, H. Fischer, B. Wagner, F. Schuler, L. Polonchuk, E. M. Carreira, Oxetanes as promising modules in drug discovery. *Angew. Chem. Int. Ed. Engl.* **45**, 7736–7739 (2006).

79. J. A. Burkhard, G. Wuitschik, M. Rogers-Evans, K. Müller, E. M. Carreira, Oxetanes as versatile elements in drug discovery and synthesis. *Angew. Chem. Int. Ed. Engl.* **49**, 9052–9067 (2010).

80. J. A. Bull, R. A. Croft, O. A. Davis, R. Doran, K. F. Morgan, Oxetanes: Recent advances in synthesis, reactivity, and medicinal chemistry. *Chem. Rev.* **116**, 12150–12233 (2016).

81. R. A. Naikoo, R. Painuli, Z. Akhter, P. P. Singh, Cannabinoid receptor 2 (CB2) modulators: A patent review (2016–2024). *Bioorg. Chem.* **153**, 107775 (2024).

82. P. Morales, M. Gómez-Cañas, G. Navarro, D. P. Hurst, F. J. Carrillo-Salinas, L. Lagartera, R. Pazos, P. Goya, P. H. Reggio, C. Guaza, R. Franco, J. Fernández-Ruiz, N. Jagerovic, Chromenopyrazole, a versatile cannabinoid scaffold with in vivo activity in a model of multiple sclerosis. *J. Med. Chem.* **59**, 6753–6771 (2016).

83. J.-M. Adam, P. Dott, M. Hohler, T. Burger, O. Tagliente, E. Mischler, Process for the preparation of HU-910 and crystalline structure thereof. *Patent WO2018087767A1* (2018).

84. N. Itagaki, T. Sugahara, Y. Iwabuchi, Expedient synthesis of potent cannabinoid receptor agonist (−)-CP55,940. *Org. Lett.* **7**, 4181–4183 (2005).

85. P. Li, C. Guo, S. Wang, D. Ma, T. Feng, Y. Wang, Y. Qiu, Facile and general electrochemical deuteration of unactivated alkyl halides. *Nat. Commun.* **13**, 3774 (2022).

86. D. Wood, S. Lin, Deuterodehalogenation under net reductive or redox-neutral conditions enabled by paired electrolysis. *Angew. Chem. Int. Ed. Engl.* **62**, e202218858 (2023).

87. L. Ge, Y. Li, H. Bao, Iron-catalyzed radical acyl-azidation of alkenes with aldehydes: Synthesis of unsymmetrical β-azido ketones. *Org. Lett.* **21**, 256–260 (2019).
